# Structural Diversity and Biological Activity of Cyanopeptolins Produced by *Nostoc edaphicum* CCNP1411

**DOI:** 10.3390/md21100508

**Published:** 2023-09-26

**Authors:** Robert Konkel, Marta Cegłowska, Karolina Szubert, Ewa Wieczerzak, Sofia Iliakopoulou, Triantafyllos Kaloudis, Hanna Mazur-Marzec

**Affiliations:** 1 Department of Marine Biology and Biotechnology, Faculty of Oceanography and Geography, University of Gdańsk, PL-81378 Gdynia, Poland; robert.konkel@phdstud.ug.edu.pl (R.K.); karolina.szubert@phdstud.ug.edu.pl (K.S.); 2 Institute of Oceanology, Polish Academy of Sciences, Powstańców Warszawy 55, PL-81712 Sopot, Poland; mceglowska@iopan.pl; 3 Department of Biomedical Chemistry, Faculty of Chemistry, University of Gdańsk, Wita Stwosza 63, PL-80308 Gdańsk, Poland; ewa.wieczerzak@ug.edu.pl; 4 Department of Sustainable Agriculture, University of Patras, GR-30131 Agrinio, Greece; chem3642@upnet.gr; 5 Institute of Nanoscience & Nanotechnology, NCSR Demokritos, GR-15310 Agia Paraskevi, Greece; t.kaloudis@inn.demokritos.gr; 6 Laboratory of Organic Micropollutants, Water Quality Control Department, EYDAP SA, Menidi, GR-13674 Athens, Greece

**Keywords:** cyanopeptolin, cyanobacteria, proteases inhibition, anticancer activity

## Abstract

Cyanopeptolins (CPs) are one of the most commonly occurring class of cyanobacterial nonribosomal peptides. For the majority of these compounds, protease inhibition has been reported. In the current work, the structural diversity of cyanopeptolins produced by *Nostoc edaphicum* CCNP1411 was explored. As a result, 93 CPs, including 79 new variants, were detected and structurally characterized based on their mass fragmentation spectra. CPs isolated in higher amounts were additionally characterized by NMR. To the best of our knowledge, this is the highest number of cyanopeptides found in one strain. The biological assays performed with the 34 isolated CPs confirmed the significance of the amino acid located between Thr and the unique 3-amino-6-hydroxy-2-piperidone (Ahp) on the activity of the compounds against serine protease and HeLa cancer cells.

## 1. Introduction

Cyanobacteria are widely recognized as a source of different classes of non-ribosomal peptides. Of these, microcystins, cyanopeptolins, anabaenopeptins, microginins, and aeruginosins have been most commonly studied [1,2]. Cyanopeptolins (CPs) belong to cyclic depsipeptides. They were first isolated from *Microcystis* sp. PCC 7806 [3] and identified in such cyanobacterial genera as *Caldora* [4], (Coleofasciculales), *Anabaena* [5], *Dichothrix* [6], *Microchaete* [7], *Nostoc* [8,9,10,11,12], *Scytonema* [13,14], *Stigonema* [15], (Nostocales), *Aphanocapsa* [16], *Leptolyngbya* [17], *Lyngbya* [18,19] (Synechococcales), *Microcystis* [3,20,21,22,23,24], *Oscillatoria/Planktothrix* [25,26,27,28], *Radiocystis* [29] (Chroococcales), and *Symploca* [30,31] (Oscillatoriales). There are also reports on the presence of CPs in the sea hare *Dolabella auricularia* [32], mucus bacteria *Chondromyces* [33], and *Streptomyces olivochromogenes* NBRC 3561 [34].

Similar to many other non-ribosomal peptides, cyanopeptolins belong to bioactive metabolites with some biotechnological potential. The peptides are primarily known for their ability to inhibit serine proteases such as trypsin [22,35,36,37,38], chymotrypsin [22,33,39,40,41], thrombin [36,42,43], elastase [4,28,44,45,46], plasmin [28,36,37,47,48], proteinase-3, cathepsin G [49], and kallikrein [35]. They also affect the activity of protein phosphatase 1 (PP1) and PP2 [11,47], cytosolic AP [21], aminopeptidase N (APN) [21,50], and factor XIa [35]. Less frequently, antimicrobial, anticancer [4,51,52,53], and antiviral activities [54] have been reported.

To date, 227 CPs and CP-like compounds have been described [1], 103 of which are produced by cyanobacteria of the *Microcystis* genus. They have been named aeruginopeptins [23,55], anabaenopeptolides [5], bouillomides [19], crocapeptins [33], dolastatin [32], hofmannolin [14], ichthyopeptins [54], insulapeptolides [49], jizanpeptins [56], kempopeptins [18,52], kyanamide [4], largamides [26], lyngbyastatins [44,57,58], loggerspeptins [52], microcysilide [59], micropeptins [60,61,62,63,64,65,66], molassamide [6,52], nostocyclin [11], nostopeptins [9], oscillapeptilides [27], oscillapeptins [27,28,46,67], planktopeptins [45], pompanopeptin [68], scyptolins [13,14], somamides [69], stigonemapeptin [15], streptopeptolin [34], symplocamide [31], and tasipeptins [30].

**Figure 1:**
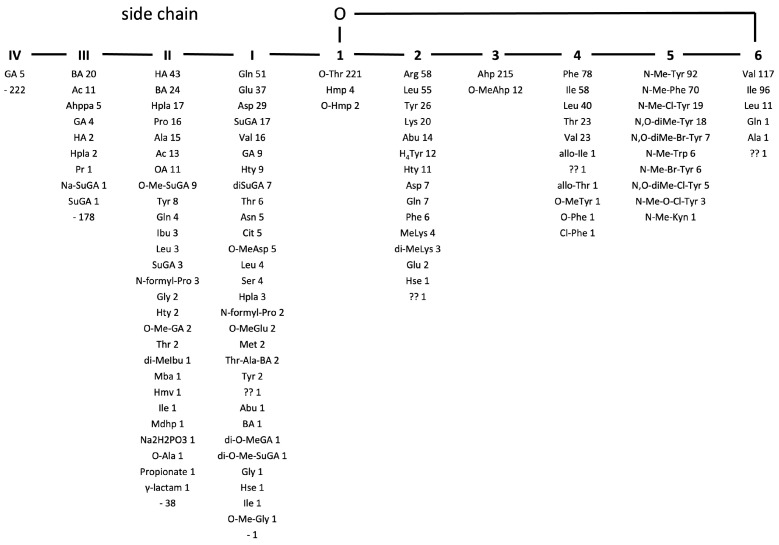
General structure of cyanopeptolin variants with residues occupying a given position (Abu—2-amino-2-butenoic acid; Ac—acetate; Ahp—3-amino-6-hydroxy-2-piperidone; Ahppa—2-amino-5-(4′-hydroxyphenyl)pentanoic acid; BA—Butanoic acid; Cit—Citrulline; GA—Glyceric acid; H_4_Tyr—Tetrahydrotyrosine; HA—Hexanoic acid; Hmp—3-hydroxy-4-methyl-proline; Hmv—2-hydroxy-3-methylvaleric acid; Hpla—Hydroxy-phenyl lactic acid; Hse—Homoserine; Hty—Homotyrosine; Ibu—Isobutyric acid; Kyn—Kynurenine; Mba—Methyl-2-butenoic acid; Mdhp—Methyl-dehydroproline; OA—Octanoic acid; Pr—Propanoyl; Su—Sulfo).

The structural diversity of these compounds is a consequence of their biosynthetic pathway. Biosynthesis is carried out on large enzymatic complexes called non-ribosomal peptide synthetases (NRPS). One cyanobacterial strain can produce a mixture of non-ribosomal peptides with modifications in different positions of their structure [70,71]. Typically, all CPs consist of a six-amino acid cyclic part closed by an ester bond between the *b*-hydroxy group of the residue in position 1 and the carboxy-group of the residue in position 6 (Figure 1, Table S1 [3,5,6,7,8,9,10,11,13,14,15,16,19,20,21,22,23,24,25,26,27,28,29,30,31,32,33,34,35,36,37,38,40,41,42,43,44,45,46,47,49,52,54,55,56,57,58,59,60,61,62,63,64,65,67,68,69,72,73,74,75,76,77,78,79,80,81,82,83,84,85,86,87,88,89,90]). Most CPs have a side-chain composed of 1–4 units attached to the residue in position 1, usually occupied by Thr (221/227), optionally, by 3-hydroxy-4-methyl-proline (Hmp) (4/227) [49] or *O*-Hmp (2/227) [9]. A common feature of all cyanopeptolins is the presence of 3-amino-6-hydroxy-2-piperidone (Ahp) or its methylated form in position 3 (Figure 1). Position 5 is occupied by methylated aromatic amino acids, mainly tyrosine or phenylalanine. In some cases, the aromatic amino acid is chlorinated or brominated [16,18,52,56,68,73]. Position 6 is quite conserved and mainly occupied by Val, Leu, or Ile. In one CP variant, this position is occupied by Gln, and in one by Ala. High structural variability of CP-like depsipeptides is also evident in a wide range of molecular masses of the compounds: from 770 Da for tasipeptin A [30] to 1181 Da for oscillapeptin B [28].

Structural elucidation of CPs and other natural products is mainly based on high-resolution mass spectrometry (HRMS), the accurate *m/z* measurements of pseudomolecular ions, and the analysis of fragmentation spectra. Accessible platforms such as GNPS (Global Natural Product Social Molecular Networking) facilitate the analysis and exchange of tandem mass spectrometry data (MS/MS) [91]. The Feature-Based Molecular Networking (FBMN) workflow in GNPS [92] builds on chromatographic feature detection and alignment and connects related molecules by their spectral similarity to ‘spectral families‘. In addition to automated search of the spectral library, visualization of the molecular network facilitates spectral annotation and dereplication [91,92,93,94,95,96].

In our previous studies, the production of 13 cyanopeptolins by *Nostoc edaphicum* CCNP1411 was reported [8]. The goal of the current work was to expand the existing knowledge about the structural diversity of CPs produced by CCNP1411 and to explore its effect on the biological activity of the peptides.

## 2. Results and Discussion

### 2.1. Identification of CP Structures

Cyanobacteria possess the ability to synthesize a wide array of natural products. The analyses of 185 cyanobacterial genomes led to the identification of 1817 natural products biosynthetic gene clusters (BGCs) [97]. In the same study, a positive correlation between the number of BGCs and the size of the genome was documented. Cyanobacteria of the order Nostocales are characterized by the largest genomes and are among those that pose the highest average number (11–25) of natural product BGCs [97]. Many of the synthesized compounds are biologically active, and their biotechnological and pharmaceutical potential is commonly explored. In CCNP1411, three classes of non-ribosomal peptides were identified. This includes: anabaenopeptins with four structural variants [98], nostocyclopeptides with six linear and five cyclic variants [99], and thirteen cyanopeptolins [8]. In the current study, the number of CPs variants detected in CCNP1411 increased to 93. However, when the cell extract of CCNP1411 was analyzed with LC-HRMS, only 67 CPs were detected. For these peptides, the exact masses were determined [Table S2]. The remaining peptides were detected in concentrated fractions collected during the separation process.

**Figure 2:**
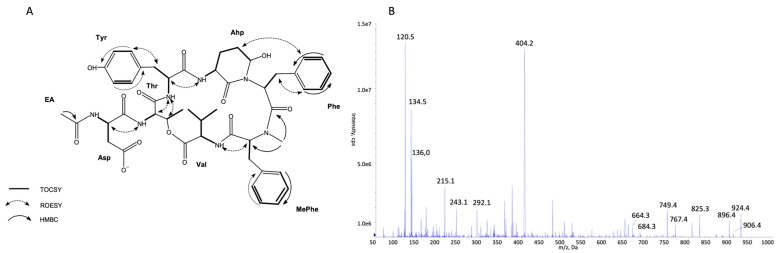
Key TOCSY, ROESY, and HMBC correlations (**A**) and enhanced product ion mass spectrum of the cyanopeptolin CP 941 with precursor ion [M + H − H_2_O]^+^ at *m/z* 924.4 (**B**).

**Figure 3:**
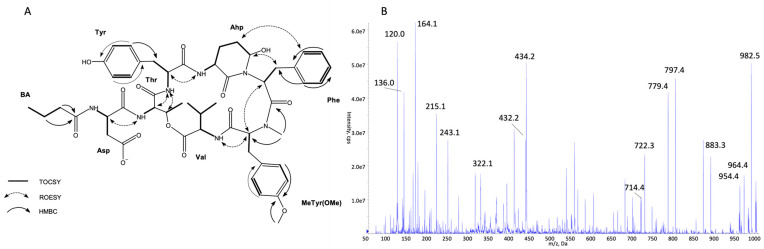
Key TOCSY, ROESY, and HMBC correlations (**A**) and enhanced product ion mass spectrum of the cyanopeptolin CP 999 with precursor ion [M + H − H_2_O]^+^ at *m/z* 982.5 (**B**).

**Figure 4:**
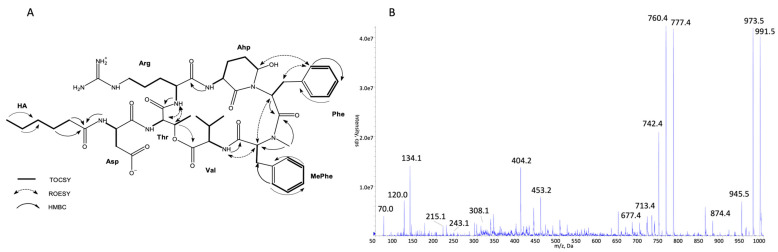
Key TOCSY, ROESY, and HMBC correlations (**A**) and enhanced product ion mass spectrum of the cyanopeptolin CP 990 with precursor ion [M + H]^+^ at *m/z* 991.5 (**B**).

**Figure 5:**
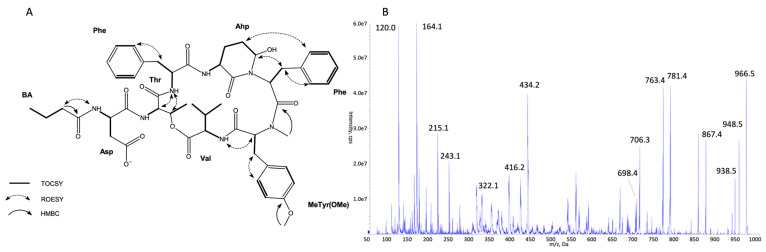
Key TOCSY, ROESY, and HMBC correlations (**A**) and enhanced product ion mass spectrum of the cyanopeptolin CP 983 with precursor ion [M + H − H_2_O]^+^ at *m/z* 966.5 (**B**).

**Figure 6:**
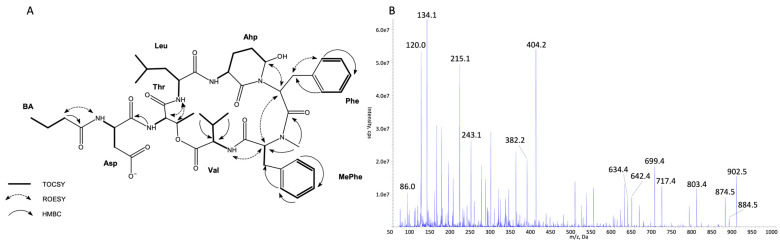
Key TOCSY, ROESY, and HMBC correlations (**A**) and enhanced product ion mass spectrum of the cyanopeptolin CP 919 with precursor ion [M + H − H_2_O]^+^ at *m/z* 902.5 (**B**).

**Figure 7:**
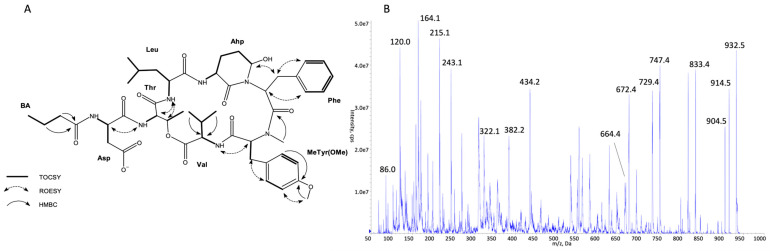
Key TOCSY, ROESY, and HMBC correlations (**A**) and enhanced product ion mass spectrum of the cyanopeptolin CP 949 with precursor ion [M + H − H_2_O]^+^ at *m/z* 932.5 (**B**).

**Figure 8:**
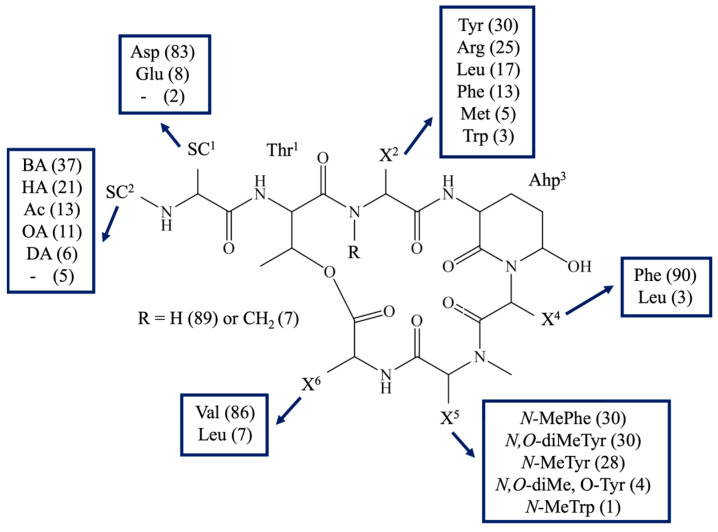
General structure of cyanopeptolins produced by *Nostoc edaphicum*. The number of variants with specific amino acids is given in brackets. SC indicates side-chain.

The structures of all detected peptides were identified based on their mass fragmentation spectra (Figure 2, Figure 3, Figure 4, Figure 5, Figure 6 and Figure 7 and Figures S1–S87). In the spectra of all CPs containing Phe in position 4, there were ion peaks at *m/z* 243 [Ahp + Phe + H − H_2_O]^+^ and 215 [Ahp + Phe + H − CO − H_2_O]^+^, while those containing Leu^4^ gave ion peaks at *m/z* 209 [Ahp + Leu + H − H_2_O]^+^ and 181 [Ahp + Leu + H − CO − H2O]^+^. Immonium ions at *m/z* 86 (Leu/Ile), 120 (Phe), 134 (MePhe), 150 (MeTyr), 159 (Trp), 173 (MeTrp), 164 (diMeTyr), and 180 (diMe,OTyr), and the peak at *m/z* 405, 420, 434, and 450 corresponding to fragments [Ahp^3^ + Phe^4^ + X^5^ + H − H_2_O] or at *m/z* 371, 386, 400, and 416 corresponding to fragments [Ahp^3^ + Leu^4^ + X^5^ + H − H_2_O] belong to the most important diagnostic ions. Other ions that supported the process of structure elucidation are listed in Table S3. For the dereplication process, the CPs identified in CCNP1411 were compared with the resources of the CyanoMetDB [1]. This is the most comprehensive and openly accessible database containing cyanobacterial metabolites. The updated versions of the database are available on the Zenodo and NORMAN Suspect List Exchange (No S075). Of the 93 CPs detected in CCNP1411 in this work, only 14 were included in the database. Generally, the presence and frequency of specific residues in the structure of CPs produced by CCNP1411 (Figure 8) were in line with the residues present in the previously identified CPs presented in Figure 1. Position 2 of the CPs is most diverse and occupied by Arg, Tyr, Phe, Leu, Met, Trp, as well as methylated Leu, Phe, and Tyr. Similarly to the spectra of Tyr^2^, H_4_Tyr^2^, and Leu^2^-containing aeruginopeptins 917S-A, -B, and -C [55], the Tyr^2^, Leu^2^, or Phe^2^-containing CPs identified in CCNP1411 gave a high intensity dehydrated [M + H − H_2_O]^+^ precursor ion peak.

Based on the mass fragmentation spectra, it is not possible to distinguish the isobaric residues (e.g., Ile/Leu). Therefore, for CPs isolated in the highest quantities, i.e., CP 941 and CP 999 (with Tyr^2^), CP 990 (with Arg^2^), CP 983 (with Phe^2^), CP 949 and CP 919 (with Leu^2^), NMR analyses were performed (Figure 2, Figure 3, Figure 4, Figure 5, Figure 6 and Figure 7, Figures S88–S123, Tables S4–S9). The obtained results were consistent with structure elucidation based on MS/MS and allowed the identification of Leu^2^ in CP 949 and CP 919. The NMR analyses also allowed us to verify the previously published structure of CP 999 [8]. It was revealed that position 5 in CP 999 is occupied by *N,O*-di-MeTyr, and not by MeHty, as suggested based on the MS/MS spectrum. Both residues give the same fragment ions, including the immonium ion at *m/z* 164. *N,O*-di-MeTyr^5^ was previously detected in cyanopeptolins produced by *Nostoc insulare* [49] and *Oscillatoria agardhii* [27,28,46,84]. This structure misinterpretation illustrates well the need for the application of at least two spectroscopic methods, e.g., NMR and MS/MS, to provide the correct information on chemical structure, especially when isomers are analyzed [100]. Unfortunately, in the case of natural products, which are biosynthesized in minute amounts, the isolation of sufficient amounts of pure compound (>1 mg) for NMR is impossible or difficult to achieve. Then, the structural analyses can be based on HRMS/MS, which allows the assignment of molecular formula and provides important information on the structural components of the analyte [101]. Other, more recently developed MS techniques (e.g., ion mobility MS) can additionally support the structure elucidation process [102].

Of the 25 CP-like peptides identified in cyanobacteria of the genus *Nostoc* and included in Table S1, more than half (13) were reported from CCNP1411 [8]. When all structural variants from this study are included in the database, *Nostoc* can be considered as rich source of cyanopeptolins as *Microcystis*.

### 2.2. Molecular Networking of Cyanopeptolins

To describe the structural diversity of CPs, molecular networking was performed using data from the HRMS/MS analysis of 10-mg dry biomass of CCNP1411 cell extract. A search of databases linked with the GNPS spectra library did not detect any CPs produced by *N. edaphicum*. Instead, it proposed 209 compounds structurally similar to CCNP1411 cyanopeptolins, including anabaenopeptilide 202A, cyanopeptolin 963A, lyngbyastatin 8, and micropeptin 103. The search also resulted in the detection of 27 compounds within the 195–532 *m/z* range.

The molecular network for *N. edaphicum* CCNP1411 showed the existence of 116 nodes connected into 9 clusters by 320 edges (Figure S124), including 3 clusters with CPs features (Figure S124A), 4 with nostocyclopeptides features (Figure S124B), and 2 clusters which did not match any of the above-mentioned groups of compounds (Figure S124C).

**Figure 9:**
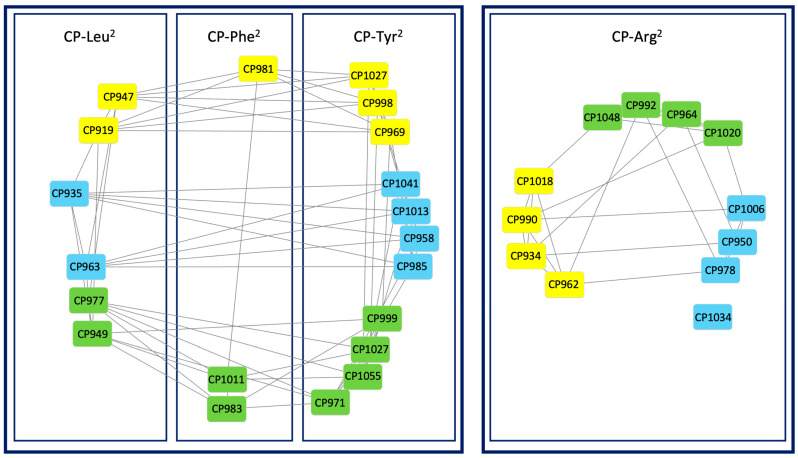
Molecular network generated from HRMS/MS spectra of *N. edaphicum* CCNP1411 extract using the FBMN tool in GNPS. Only nodes corresponding to the annotated cyanopeptolins are represented (green—di-MeTyr, blue-MeTyr, yellow—MePhe).

The 3 CP clusters were grouped into 62 nodes connected by 202 edges. We were able to assign 32 nodes to specific CPs variants identified in CCNP1411 (Figure S124). The *m/z* values of the remaining nodes did not match the compounds described in this work, or their weak spectra did not allow the features to be confidently assigned to specific CP variants. A visualization of the 32 annotated CPs is shown in (Figure 9).

These 32 CPs were grouped into two main clusters based on the similarity of fragmentation pattern profiles being a consequence of their specific structural traits (Figure 9). The Arg^2^-bearing CPs were distinctly separated from variants with Tyr^2^, Leu^2^, or Phe^2^, which showed higher similarity to each other. This grouping might result from the fact that, unlike CPs with Arg^2^, the three types of CPs gave dehydrated ions as parent ions in their spectra. In both clusters, the CPs with different amino acids in position 5 grouped separately. Visualization of the structural relationships between CPs using a molecular network yielded consistent results with manually performed structural analysis of MS/MS data.

### 2.3. Enzymatic Assay

Serine proteases play a significant role in major metabolic pathways. Therefore, inhibitors of these enzymes potentially constitute lead compounds in pharmaceutical research. In our study, 34 CPs were isolated as pure compounds (purity > 95%) (Table 1) and their in vitro activities against four serine proteases (trypsin, chymotrypsin, elastase, and thrombin), were determined. In line with our previous results [8], neither of the peptides were active against thrombin, even at the highest concentration applied in the assay (45 µg × mL^−1^). Our current work also confirmed the significance of the residue in position 2 for the inhibition of trypsin, chymotrypsin, and elastase. Peptides with Arg^2^ inhibited trypsin at IC_50_ values from 0.28 µM (CP 1018) to 7.25 µM (CP 1048) and showed weaker or no activity against chymotrypsin (from IC_50_ = 6.75 µM to nonactive) (Table 1). Similar effects of CPs with Arg^2^ on trypsin and no or weak effect against chymotrypsin were previously reported by other authors [22,28,37,38,47,74]. Opposite results were reported only for a CP-like peptide called symplocamide A [31]. The peptide inhibited trypsin at IC_50_ = 80.2 ± 0.7 μM and showed more potent activity against chymotrypsin (IC_50_ = 0.38 ± 0.08 μM). The authors suggested that the activity of symplocamide A can be modified by the *N,O*-dimethylbromotyrosine at position 5.

In line with previous findings [20,21,41,82], CPs with hydrophobic amino acid residues, i.e., Tyr^2^, Phe^2^ and Leu^2^, inhibited the activity of chymotrypsin (Table 1). The exception was CP1011. The lowest IC_50_ value was determined for CP958 (0.38 µM). The presence of Leu^2^ was additionally associated with the inhibition of elastase [4,14,49,52], however, compared to CP-like peptides such as kyanamide (IC_50_ = 0.13 nM) from *Caldora penicillate* [4] or loggerpeptins [52], the effects were moderate (minimum IC_50_ = 3.32 µM for CP949) (Table 1). In the case of known CP-like peptides, elastase inhibition was additionally enhanced by the presence of 2-amino-2-butenoic acid (Abu^2^). For Abu^2^-containing lyngbyastatins, symplostatins, and molassamide, the IC_50_ values were in a sub-micromolar range [52,58,103]. The molecular docking [52,103] and analyses of crystal structure [14] revealed that Abu^2^ and Leu^2^ occupy the S1–S4 elastase subunits and confirmed the significance of these amino acids for their interaction with the enzyme.

Although the amino acid in position 2 is belived to be critical for the interaction of CPs with serine proteases, variants with no activity have been reported [42,47,64]. This fact indicates that other components of the molecules are important for enzyme inhibition as well. Indeed, in the work by Salvadore et al. [103], symplostatins with *N*-MeTyr^5^ were found to be slightly stronger inhibitors of elastase than those with *N*-MePhe^5^. The effect of the side-chain on the activity of CPs was also postulated. Interestingly, the two Arg^2^-containing CPs from CCNP1411 that lack the side-chain (CP 809 and CP 778) were not active (Table 1). Thus far, the CP-like peptide composed of only the cyclic part was tested once [47]. Micropeptin MZ771, with Arg^2^ and without the side-chain, did not affect the activity of enzymes. In addition, CPs with the same cyclic part but differing in the side-chain structure (e.g., CP 1048 and CP 1020b) were shown to have different effects on the tested enzyme (7.25 and 0.39 µM, respectively).

### 2.4. MTT Assay

**Figure 10:**
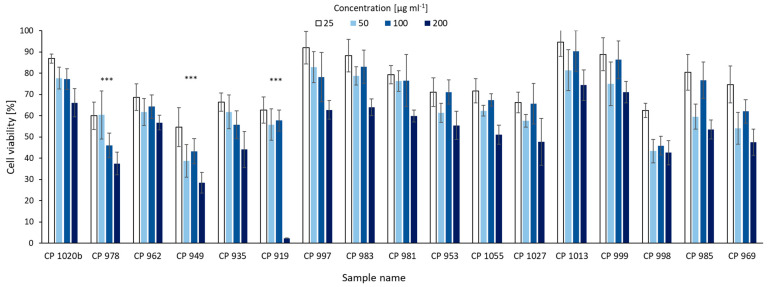
Effect of cyanopeptolins (tested at a range of concentration 25, 50, 100, and 200 µg × mL^−1^) on the proliferation of human cervical cancer (HeLa) cells. CPs that at the highest concentration reduced cell viability by more than 60% were marked with asterixis as significant.

The cytotoxic activity of two CPs produced by CCNP1411, CP 962 with Arg^2^, and CP 985 with Tyr^2^, was previously tested against a breast cancer cell line and no effects were observed, even at 500 µg × mL^−1^ [8]. In the current study, the activity of 17 isolated CPs against a human cervical cancer (HeLa) cell line was assayed. Only for one of the free Arg^2^-containing CPs, CP 978, was the concentration-dependent reduction in cell viability significant (Figure 10). At the highest concentration (200 µg × mL^−1^), the cell viability was 62.5% (SD = 5.35) lower than in the control. Significant effects were also observed for Leu^2^-containing CPs, especially CP 949 and CP 919, which at 200 µg × mL^−1^ reduced cell viability by 71.5% (SD = 4.92) and 97.6% (SD = 0.12). Other CPs had no effect on cancer cell proliferation. The cytotoxic effects of CP-like peptides have been rarely reported. Among the few examples there are: symplocamide A that affected H-460 lung cancer cells and neuro-2a neuroblastoma cells [31], tasipeptins A and B cytotoxic to KB human epithelial carcinoma cells [30], molassamide inhibiting the elastase-mediated migration of breast cancer cells [52], and kyanamide which was moderately cytotoxic to HeLa S3 cells [4]. The majority of the cytotoxic CP-like peptides belong to Leu^2^ or Abu^2^ bearing analogues and elastase inhibitors, suggesting that these amino acids are critical for activity against both targets.

## 3. Materials and Methods

### 3.1. Extraction and Isolation of Cyanopeptolins

*N. edaphicum* CCNP1411 (GenBank accession number KJ161445), isolated from the Gulf of Gdańsk, was grown for biomass as previously described by Fidor et al. [99]. The freeze-dried material (100 g) was extracted with 75% methanol (MeOH) in MilliQ water (1000 mL × 5) by vortexing (15 min × 5) and bath sonication (10 min × 3) followed by centrifugation (10,000× *g*; 15 min; 4 °C). The separation of the material was performed with the application of the Shimadzu HPLC system (Shimadzu Corporation, Kyoto, Japan). First, the sample was loaded onto a Biotage^®^Sfär C18 D flash chromatography column (120 g, 100 Å, 30 μm) (Biotage, Uppsala, Sweden) and a step gradient elution (12 mL × min^−1^) with water: methanol mixture was applied. The fractions containing CPs were evaporated in a centrifugal vacuum concentrator (MiVac, SP Scientific, Ipswich, UK) and subjected to further separation in Jupiter Proteo C12 preparative and analytical columns (250 × 21.2 mm, 4 μm, 90 Å, 12 mL × min^−1^; 250 × 10.0 mm, 4 μm, 90 Å, 5 mL × min^−1^; 150 × 4.6 mm, 4 μm, 90 Å, 0.5 mL × min^−1^) (Phenomenex, Aschaffenburg, Germany). The mobile phase was a mixture of 5% acetonitrile in MilliQ water (phase A) and 100% acetonitrile (phase B), both with the addition of 0.1% formic acid. The collected fractions were analyzed with the application of LC-MS/MS. If needed, the fractionation process was repeated.

### 3.2. LC-MS/MS Analysis

The LC-MS/MS system composed of Agilent 1200 HPLC (Agilent Technologies, Waldbronn, Germany) and a QTRAP5500 tandem mass spectrometer (Sciex, Toronto, Canada) was used. Compounds were separated in a Jupiter Proteo C12 column (150 × 4.6 mm, 4 μm, 90 Å) (Phenomenex, Aschaffenburg, Germany), using water: acetonitrile mixture (both solvents with 0.1% formic acid). The turbo ion spray operated in positive ionisation, at 550 °C; voltage, 5.5 kV; nebuliser gas pressure, 60 psi; curtain gas pressure, 20 psi. To determine the content of the samples, an IDA (information-dependent acquisition) mode was used, and ions within the *m/z* range 500–1250 and intensity greater than 5 × 10^5^ cps were fragmented. The collision energy was 60 eV, and the dwell time was 100 msec.

### 3.3. LC-HRMS Analysis

The analysis of CPs present in the cell extract was performed with theapplication of an Elute HPG1300 HPLC system (Bruker Daltonics, Bremen, Germany) coupled with an Impact II high-resolution time of flight tandem mass spectrometer (QToF-HRMS) (Bruker Daltonics, Bremen, Germany). Chromatographic separation was performed in an Atlantis T3 C18 column (100 Å, 3 µm, 2.1 mm × 100 mm, Waters) with a VanGuard cartridge precolumn (Waters). The mobile phases were water (A) and acetonitrile (B) both acidified with 0.1% formic acid. A gradient elution program from 25 to 100% B was used with a constant flow of 0.2 mL × min^−1^. The ESI conditions were: positive ionization mode, capillary voltage 3100 V, nebulizer gas 1.0 bar, dry gas 6.0 L × min^−1^, dry gas temperature 220 ℃, hexapole 100 Vpp and pre-pulse storage 5 μs. Stepping mode was activated as follows: collision RF from 200 Vpp to 700 Vpp (50–50% of the timing), transfer time from 20 μs to 80 μs (50–50% of the timing) and collision energy from 8.4 eV to 10.5 eV (25–7 5% of the timing). Full scan accurate mass spectra were obtained in the range 50–1300 *m/z* in Auto MS (Data Dependent Analysis, DDA) with dynamic exclusion. Calibration was carried out in every sample run using the sodium formate cluster ions (10 mM). Bruker’s HyStar and Data Analysis software was utilized for data acquisition, calibration, and raw data conversion to the .mzXML format before further processing.

### 3.4. Molecular Networking

A molecular network was created with the Feature-Based Molecular Networking (FBMN) workflow [92] on GNPS (https://gnps.ucsd.edu, accessed on 10 August 2023) [93]. The mass spectrometry data were first processed with MZmine3 [104] and the results were exported to GNPS for FBMN analysis. Data were filtered by removing all MS/MS fragment ions within ±17 Da of the precursor *m/z*. MS/MS spectra were window filtered by choosing only the top 6 fragment ions in the ±50 Da window throughout the spectrum. The precursor ion mass tolerance was set to 0.05 Da and the MS/MS fragment ion tolerance to 0.05 Da. A molecular network was then created where the edges were filtered to have a cosine score greater than 0.7 and more than 6 matched peaks. Further, edges between two nodes were kept in the network if, and only if, each of the nodes appeared in each others respective top 10 most similar nodes. Finally, the maximum size of a molecular family was set to 100, and the lowest scoring edges were removed from the molecular families until the molecular family size was below this threshold. The spectra in the network were then searched against the GNPS spectral libraries [93]. The library spectra were filtered in the same manner as the input data. All matches kept between network spectra and library spectra were required to have a score greater than 0.7 and at least 6 matched peaks. The DEREPLICATOR was used to annotate MS/MS spectra [105]. The molecular networks were visualized using Cytoscape software [106].

### 3.5. NMR Analysis

The ^1^H NMR and 2D homo- and heteronuclear NMR (COSY, TOCSY, ROESY, HSQC, and HMBC) were acquired with the application of a Bruker Avance III spectrometers at 500 MHz (1D NMR) and 700 MHz (2D NMR). The spectra were recorded in dimethyl sulfoxide-d6 (DMSO-d6). The NMR data were processed and analyzed by TopSpin (Bruker, Billerica, MA, USA) and POKY software [107].

### 3.6. Enzymatic Assays

The enzyme inhibitory activity of cyanopeptolins was assayed against trypsin [108], chymotrypsin, thrombin [109], and elastase [57] as described before [98]. In brief, the samples were prepared and serially diluted (1 mg, 1:1—1:10,000 times) in 1% DMSO; the standard inhibitors: aprotinin (trypsin and chymotrypsin) AEBSF 4-(2-aminoethyl)benzenesulfonyl fluoride hydrochloride (thrombin), elastatinal (elastase) (all from Sigma Aldrich; St. Louis, MO, USA) were also prepared in 1% DMSO. DMSO (1%) without the addition of the enzyme served as a negative control. All tests were carried out in triplicates. After the incubation of the assay mixtures, the absorbances were measured at 405 nm (Varioskan Flash Thermo Fisher Scientific OY, Vantaa, Finland). The reduction in enzyme activity greater than 50% was considered as significant.

### 3.7. MTT Assays

The cytotoxic activity of the selected 17 CPs against the HeLa cervical cancer cell line (Merck KGaA, Darmstadt, Germany) was assessed with the MTT (3-(4,5-dimethylthiazole-2-yl)-2,5-diphenyltetrazolium bromide) assay according to Felczykowska et al. [110]. HeLa cells were seeded at 4 × 10^3^ cells per well in DMEM medium (Merck KGaA) supplemented with 10% (*v*/*v*) fetal bovine serum (Merck KGaA) and penicillin-streptomycin solution (1% *v/v*, stock solution 50 u and 0.05 mg × mL^−1^, respectively; Merck KGaA). Cells were allowed to attach overnight, and then the tested compounds prepared in 1% DMSO were added. After incubation (24 h), 25 µL of MTT solution (4 mg × mL^−1^; Merck KGaA) were added and then the samples were incubated for another 4 h. The formazan crystals were dissolved with 100% DMSO and absorbance was measured with Spectramax i3 (Molecular Deviecs, LLC San Jose, CA, USA). The reduction in cell viability greater than 60% was considered as significant.

## 4. Conclusions

Analysis of concentrated samples obtained from higher biomass of *Nostoc edaphicum* CCNP1411 resulted in the identification of 93 cyanopeptolins, including 79 new variants. To the best of our knowledge, this is the highest number of cyanopeptides ever recorded in one strain. The tests performed with the application of 34 isolated CPs of diverse structure confirmed the role of the residue located between Thr^1^ and Ahp^3^ on the activity of the compounds. Arg^2^-containing CPs were most active against trypsin, CPs with hydrophobic amino acid in position 2 inhibited chymotrypsin, while only CPs with Leu^2^ inhibited elastase and showed the most potent cytotoxic effect on human cervical cancer (HeLa) cells. The enzymatic assays also indicated the significance of the CP side-chain for the interactions with serine proteases. With the cytotoxic activity against cancer cells and the activity against enzymes implicated in a number of human diseases, CPs can be classified as lead compounds for further studies on their pharmaceutical potential.

## Figures and Tables

**Table 1:** Enzymatic activity of cyanopeptolins against serine proteases (trypsin (TRY), chymotrypsin (CHY), elastase (E), and thrombin) (TRB)). (*) results published in [8]. (−, not active (inhibition below 50%); +/−, low activity (inhibition between 50 and 60%); +, medium activity (up to 60%).

Name	Structure	TRY	CHY	E	TRB
		[µM]			
CP 1048	[Thr + Arg + Ahp + Phe + diMeTyr + Val]Asp + OA	7.25	+/−	−	−
CP 1034	[Thr + Arg + Ahp + Phe + MeTyr + Val]Asp + OA	5.51	−	−	−
CP 1020b	[Thr + Arg + Ahp + Phe + diMeTyr + Val]Asp + HA	0.39 (0.25 *)	3.6 (3.1 *)	−	−
CP 1018	[Thr + Arg + Ahp + Phe + MePhe + Val]Asp + OA	0.28 (0.24 *)	−	−	−
CP 992	[Thr + Arg + Ahp + Phe + diMeTyr + Val]Asp + BA	0.31 (0.24 *)	3.32 (3.5 *)	−	−
CP 990	[Thr + Arg + Ahp + Phe + MePhe + Val]Asp + HA	3.73	−	−	−
CP 978	[Thr + Arg + Ahp + Phe + MeTyr + Val]Asp + BA	0.29 (0.26 *)	4.2 (3.8 *)	−	−
CP 962	[Thr + Arg + Ahp + Phe + MePhe + Val]Asp + BA	3.18	−	−	−
CP 950	[Thr + Arg + Ahp + Phe + MeTyr + Val]Asp + Ac	0.66	−	−	−
CP 934	[Thr + Arg + Ahp + Phe + MePhe + Val]Asp + Ac	0.42	6.75	−	−
CP 809	[Thr + Arg + Ahp + Phe + diMeTyr + Val]	−	−	−	−
CP 778	[Thr + Arg + Ahp + Phe + MePhe + Val]	−	−	−	−
CP 1055	[Thr + Tyr + Ahp + Phe + diMeTyr + Val]Asp + OA	−	3.69	−	−
CP 1027	[Thr + Tyr + Ahp + Phe + diMeTyr + Val]Asp + HA	−	0.38 (0.26 *)	−	−
CP 1025	[Thr + Tyr + Ahp + Phe + MePhe + Val]Asp + OA	−	+/−	−	−
CP 1013	[Thr + Tyr + Ahp + Phe + MeTyr + Val]Asp + HA	−	3.97	−	−
CP 999	[Thr + Tyr + Ahp + Phe + diMeTyr + Val]Asp + BA	−	+	−	−
CP 997b	[Thr + Tyr + Ahp + Phe + MePhe + Val]Asp + HA	−	7.10	−	−
CP 985	[Thr + Tyr + Ahp + Phe + MeTyr + Val]Asp + BA	−	0.49 (0.26 *)	−	−
CP 972	[Thr + Tyr + Ahp + Phe + diMeTyr + Val]Asp + Ac	−	5.19	−	−
CP 969	[Thr + Tyr + Ahp + Phe + MePhe + Val]Asp + BA	−	1.94	−	−
CP 958	[Thr + Tyr + Ahp + Phe + MeTyr + Val]Asp + Ac	−	0.38	−	−
CP 941	[Thr + Tyr + Ahp + Phe + MePhe + Val]Asp + Ac	−	0.7	−	−
CP 983b	[Thr + Tyr + Ahp + Phe + MePhe + Leu]Asp + BA	−	2.49	−	−
CP 949	[Thr + Leu + Ahp + Phe + diMeTyr + Val]Asp + BA	−	1.59	3.32	−
CP 935	[Thr + Leu + Ahp + Phe + MeTyr + Val]Asp + BA	−	4.92	−	−
CP 919	[Thr + Leu + Ahp + Phe + MePhe + Val]Asp + BA	−	1.45	5.71	−
CP 1011	[Thr + Phe + Ahp + Phe + diMeTyr + Val]Asp + HA	−	−	−	−
CP 997	[Thr + Phe + Ahp + Phe + MeTyr + Val]Asp + HA	−	4.64	−	−
CP 981	[Thr + Phe + Ahp + Phe + MePhe + Val]Asp + HA	−	3.92	−	−
CP 983	[Thr + Phe + Ahp + Phe + diMeTyr + Val]Asp + BA	−	0.99	−	−
CP 969b	[Thr + Phe + Ahp + Phe + MeTyr + Val]Asp + BA	−	4.78	−	−
CP 953	[Thr + Phe + Ahp + Phe + MePhe + Val]Asp + BA	−	5.95	−	−
CP 925	[Thr + Phe + Ahp + Phe + MePhe + Val]Asp + Ac	−	5.45	−	−

## Data Availability

The structural results of cyanopeptolins described in this work will be added to the CyanoMetDB and GNPS databases.

## Supplementary Materials

The following supporting information can be downloaded at: https://www.mdpi.com/article/10.3390/md21100508/s1, Table S1. Cyanopeptolin variants described so far.; Table S2. Cyanopeptolin variants produced by the *Nostoc edaphicum* CCNP1411.; Figure S1. Structure and enhanced product ion mass spectrum of the cyanopeptolin CP 777.; Figure S2. Structure and enhanced product ion mass spectrum of the cyanopeptolin CP 807.; Figure S3. Structure and enhanced product ion mass spectrum of the cyanopeptolin CP 892.; Figure S4. Structure and enhanced product ion mass spectrum of the cyanopeptolin CP 922.; Figure S5. Structure and enhanced product ion mass spectrum of the cyanopeptolin CP 934.; Figure S6. Structure and enhanced product ion mass spectrum of the cyanopeptolin CP 944.; Figure S7. Structure and enhanced product ion mass spectrum of the cyanopeptolin CP 950.; Figure S8. Structure and enhanced product ion mass spectrum of the cyanopeptolin CP 962 [8].; Figure S9. Structure and enhanced product ion mass spectrum of the cyanopeptolin CP 964.; Figure S10. Structure and enhanced product ion mass spectrum of the cyanopeptolin CP 976.; Figure S11. Structure and enhanced product ion mass spectrum of the cyanopeptolin CP 978 [8].; Figure S12. Structure and enhanced product ion mass spectrum of the cyanopeptolin CP 992 [8].; Figure S13. Structure and enhanced product ion mass spectrum of the cyanopeptolin CP 992b.; Figure S14. Structure and enhanced product ion mass spectrum of the cyanopeptolin CP992c.; Figure S15. Structure and enhanced product ion mass spectrum of the cyanopeptolin CP 1006 [8].; Figure S16. Structure and enhanced product ion mass spectrum of the cyanopeptolin CP 1008.; Figure S17. Structure and enhanced product ion mass spectrum of the cyanopeptolin CP 1018 [8].; Figure S18. Structure and enhanced product ion mass spectrum of the cyanopeptolin CP 1020b [8].; Figure S19. Structure and enhanced product ion mass spectrum of the cyanopeptolin CP 1020.; Figure S20. Structure and enhanced product ion mass spectrum of the cyanopeptolin CP 1034.; Figure S21. Structure and enhanced product ion mass spectrum of the cyanopeptolin CP 1036.; Figure S22. Structure and enhanced product ion mass spectrum of the cyanopeptolin CP 1046.; Figure S23. Structure and enhanced product ion mass spectrum of the cyanopeptolin CP 1048 [8].; Figure S24. Structure and enhanced product ion mass spectrum of the cyanopeptolin CP 1076.; Figure S25. Structure and enhanced product ion mass spectrum of the cyanopeptolin CP 891.; Figure S26. Structure and enhanced product ion mass spectrum of the cyanopeptolin CP 907.; Figure S27. Structure and enhanced product ion mass spectrum of the cyanopeptolin CP 921.; Figure S28. Structure and enhanced product ion mass spectrum of the cyanopeptolin CP 933.; Figure S29. Structure and enhanced product ion mass spectrum of the cyanopeptolin CP 935.; Figure S30. Structure and enhanced product ion mass spectrum of the cyanopeptolin CP 947.; Figure S31. Structure and enhanced product ion mass spectrum of the cyanopeptolin CP 963.; Figure S32. Structure and enhanced product ion mass spectrum of the cyanopeptolin CP 963b.; Figure S33. Structure and enhanced product ion mass spectrum of the cyanopeptolin CP 965b.; Figure S34. Structure and enhanced product ion mass spectrum of the cyanopeptolin CP 975.; Figure S35. Structure and enhanced product ion mass spectrum of the cyanopeptolin CP 977.; Figure S36. Structure and enhanced product ion mass spectrum of the cyanopeptolin CP 991.; Figure S37. Structure and enhanced product ion mass spectrum of the cyanopeptolin CP 1005.; Figure S38. Structure and enhanced product ion mass spectrum of the cyanopeptolin CP 933b.; Figure S39. Structure and enhanced product ion mass spectrum of the cyanopeptolin CP 949b.; Figure S40. Structure and enhanced product ion mass spectrum of the cyanopeptolin CP 939b.; Figure S41. Structure and enhanced product ion mass spectrum of the cyanopeptolin CP 967b.; Figure S42. Structure and enhanced product ion mass spectrum of the cyanopeptolin CP 1011c.; Figure S43. Structure and enhanced product ion mass spectrum of the cyanopeptolin CP 937.; Figure S44. Structure and enhanced product ion mass spectrum of the cyanopeptolin CP 939.; Figure S45. Structure and enhanced product ion mass spectrum of the cyanopeptolin CP 953b.; Figure S46. Structure and enhanced product ion mass spectrum of the cyanopeptolin CP 995.; Figure S47. Structure and enhanced product ion mass spectrum of the cyanopeptolin CP 1023.; Figure S48. Structure and enhanced product ion mass spectrum of the cyanopeptolin CP 999d.; Figure S49. Structure and enhanced product ion mass spectrum of the cyanopeptolin CP 1014.; Figure S50. Structure and enhanced product ion mass spectrum of the cyanopeptolin CP 925.; Figure S51. Structure and enhanced product ion mass spectrum of the cyanopeptolin CP 953.; Figure S52. Structure and enhanced product ion mass spectrum of the cyanopeptolin CP 955.; Figure S53. Structure and enhanced product ion mass spectrum of the cyanopeptolin CP 967.; Figure S54. Structure and enhanced product ion mass spectrum of the cyanopeptolin CP 969b.; Figure S55. Structure and enhanced product ion mass spectrum of the cyanopeptolin CP 981.; Figure S56. Structure and enhanced product ion mass spectrum of the cyanopeptolin CP 997.; Figure S57. Structure and enhanced product ion mass spectrum of the cyanopeptolin CP 1011.; Figure S58. Structure and enhanced product ion mass spectrum of the cyanopeptolin CP 1025b.; Figure S59. Structure and enhanced product ion mass spectrum of the cyanopeptolin CP 992d. Figure S60. Structure and enhanced product ion mass spectrum of the cyanopeptolin CP 1036c.; Figure S61. Structure and enhanced product ion mass spectrum of the cyanopeptolin CP 1050. Figure S62. Structure and enhanced product ion mass spectrum of the cyanopeptolin CP 929.; Figure S63. Structure and enhanced product ion mass spectrum of the cyanopeptolin CP 957.; Figure S64. Structure and enhanced product ion mass spectrum of the cyanopeptolin CP 965.; Figure S65. Structure and enhanced product ion mass spectrum of the cyanopeptolin CP 969 [8].; Figure S66. Structure and enhanced product ion mass spectrum of the cyanopeptolin CP 971.; Figure S67. Structure and enhanced product ion mass spectrum of the cyanopeptolin CP 983b.; Figure S68. Structure and enhanced product ion mass spectrum of the cyanopeptolin CP 983c.; Figure S69. Structure and enhanced product ion mass spectrum of the cyanopeptolin CP 985 [8].; Figure S70. Structure and enhanced product ion mass spectrum of the cyanopeptolin CP 993. Figure S71. Structure and enhanced product ion mass spectrum of the cyanopeptolin CP 997b.; Figure S72. Structure and enhanced product ion mass spectrum of the cyanopeptolin CP 999b.; Figure S73. Structure and enhanced product ion mass spectrum of the cyanopeptolin CP 999c.; Figure S74. Structure and enhanced product ion mass spectrum of the cyanopeptolin CP 1011b. Figure S75. Structure and enhanced product ion mass spectrum of the cyanopeptolin CP 1013 [8].; Figure S76. Structure and enhanced product ion mass spectrum of the cyanopeptolin CP 1013b.; Figure S77. Structure and enhanced product ion mass spectrum of the cyanopeptolin CP 1013c.; Figure S78. Structure and enhanced product ion mass spectrum of the cyanopeptolin CP 1016.; Figure S79. Structure and enhanced product ion mass spectrum of the cyanopeptolin CP 1025.; Figure S80. Structure and enhanced product ion mass spectrum of the cyanopeptolin CP 1027 [8].; Figure S81. Structure and enhanced product ion mass spectrum of the cyanopeptolin CP 1036b.; Figure S82. Structure and enhanced product ion mass spectrum of the cyanopeptolin CP 1041.; Figure S83. Structure and enhanced product ion mass spectrum of the cyanopeptolin CP 1053.; Figure S84. Structure and enhanced product ion mass spectrum of the cyanopeptolin CP 1055.; Figure S85. Structure and enhanced product ion mass spectrum of the cyanopeptolin CP 1069.; Figure S86. Structure and enhanced product ion mass spectrum of the cyanopeptolin CP 1083.; Figure S87. Structure and enhanced product ion mass spectrum of the cyanopeptolin CP 1097.; Table S3. Diagnostic ions for cyanopeptolins produced by *Nostoc edaphicum* CCNP1411.; Table S4. NMR spectroscopic data for cyanopeptolin CP 941—Ac-Asp-[Thr-Tyr-Ahp-Phe-MePhe-Val].; Figure S88. 1H NMR spectrum of cyanopeptolin CP 941 in DMSO-d6.; Figure S89. DQF-COSY spectrum of cyanopeptolin CP 941 in DMSO-d6.; Figure S90. TOCSY spectrum of cyanopeptolin CP 941 in DMSO-d6.; Figure S91. ROESY spectrum of cyanopeptolin CP 941 in DMSO-d6.; Figure S92. HSQC spectrum of cyanopeptolin CP 941 in DMSO-d6.; Figure S93. HMBC spectrum of cyanopeptolin CP941 in DMSO-d6.; Table S5. NMR spectroscopic data for cyanopeptolin CP 999—BA-Asp-[Thr-Tyr-Ahp-Phe-MeTyr(OMe)-Val].; Figure S94. 1H NMR spectrum of cyanopeptolin CP 999 in DMSO-d6. Figure S95. DQF-COSY spectrum of cyanopeptolin CP 999 in DMSO-d6.; Figure S96. TOCSY spectrum of cyanopeptolin CP 999 in DMSO-d6.; Figure S97. ROESY spectrum of cyanopeptolin CP 999 in DMSO-d6.; Figure S98. HSQC spectrum of cyanopeptolin CP 999 in DMSO-d6.; Figure S99. HMBC spectrum of cyanopeptolin CP 999 in DMSO-d6.; Table S6. NMR spectroscopic data for cyanopeptolin CP 990—HA-Asp-[Thr-Arg-Ahp-Phe-MePhe-Val].; Figure S100. 1H NMR spectrum of cyanopeptolin CP 990 in DMSO-d6.; Figure S101. DQF-COSY spectrum of cyanopeptolin CP 990 in DMSO-d6.; Figure S102. TOCSY spectrum of cyanopeptolin CP 990 in DMSO-d6.; Figure S103. ROESY spectrum of cyanopeptolin CP 990 in DMSO-d6.; Figure S104. HSQC spectrum of cyanopeptolin CP 990 in DMSO-d6.; Figure S105. HMBC spectrum of cyanopeptolin CP 990 in DMSO-d6.; Table S7. NMR spectroscopic data for cyanopeptolin CP 983—BA-Asp-[Thr-Phe-Ahp-Phe-MeTyr(OMe)-Val].; Figure S106. 1H NMR spectrum of cyanopeptolin CP 983 in DMSO-d6.; Figure S107. DQF-COSY spectrum of cyanopeptolin CP 983 in DMSO-d6.; Figure S108. TOCSY spectrum of cyanopeptolin CP 983 in DMSO-d6.; Figure S109. ROESY spectrum of cyanopeptolin CP983 in DMSO-d6.; Figure S110. HSQC spectrum of cyanopeptolin CP 983 in DMSO-d6.; Figure S111. HMBC spectrum of cyanopeptolin CP 983 in DMSO-d6.; Table S8. NMR spectroscopic data for cyanopeptolin CP 949—BA-Asp-[Thr-Leu-Ahp-Phe-MeTyr(OMe)-Val].; Figure S112. 1H NMR spectrum of cyanopeptolin CP 949 in DMSO-d6.; Figure S113. DQF-COSY spectrum of cyanopeptolin CP949 in DMSO-d6.; Figure S114. TOCSY spectrum of cyanopeptolin CP 949 in DMSO-d6.; Figure S115. ROESY spectrum of cyanopeptolin CP 949 in DMSO-d6.; Figure S116. HSQC spectrum of cyanopeptolin CP 949 in DMSO-d6.; Figure S117. HMBC spectrum of cyanopeptolin CP 949 in DMSO-d6.; Table S9. NMR spectroscopic data for cyanopeptolin CP 919—BA-Asp-[Thr-Leu-Ahp-Phe-MePhe-Val].; Figure S118. 1H NMR spectrum of cyanopeptolin CP 919 in DMSO-d6.; Figure S119. DQF-COSY spectrum of cyanopeptolin CP 919 in DMSO-d6.; Figure S120. TOCSY spectrum of cyanopeptolin CP 919 in DMSO-d6.; Figure S121. ROESY spectrum of cyanopeptolin CP 919 in DMSO-d6.; Figure S122. HSQC spectrum of cyanopeptolin CP 919 in DMSO-d6.; Figure S123. HMBC spectrum of cyanopeptolin CP 919 in DMSO-d6.; Figure S124. A CCNP1411 clusters formed by the GNPS analysis based on the HRMS/MS fragmentation spectra obtained from *Nostoc edaphicum* CCNP1411 extract. Clusters are separated as: A—nodes containing CPs features; B—nodes containing Nostocyclopeptides features, and C—nodes containing unknown features.
