# Supplementary material for: Structural Diversity and Biological Activity of Cyanopeptolins Produced by Nostoc edaphicum CCNP1411
Source: Mar Drugs. 2023 Sep 26;21(10):508. doi: 10.3390/md21100508 (PMC10608790; doi:10.3390/md21100508)
Supplement: Supplementary file 1 [file marinedrugs-21-00508-s001.zip › marinedrugs-2595626-supplementary.pdf]

# Supplementary Material: Structural diversity and biological activity of cyanopeptolins produced by *Nostoc edaphicum* CCNP1411

**Robert Konkel [1], Marta Cegłowska [2], Karolina Szubert [1], Ewa Wieczerzak [3], Sofia Iliakopoulou [4], Triantafyllos Kaloudis [5,6], and Hanna Mazur-Marzec [1,*]**

[1] Department of Marine Biology and Biotechnology, Faculty of Oceanography and Geography, University of Gdańsk, Gdynia, Poland; robert.konkel@phdstud.ug.edu.pl; karolina.szubert@phdstud.ug.edu.pl; hanna.mazur-marzec@ug.edu.pl
[2] Institute of Oceanology, Polish Academy of Sciences, Powstańców Warszawy 55, PL-81712 Sopot, Poland; mceglowska@iopan.pl
[3] Department of Biomedical Chemistry, Faculty of Chemistry, University of Gdańsk, Gdańsk, Poland; ewa.wieczerzak@ug.edu.pl
[4] Department of Sustainable Agriculture, University of Patras, Agrinio, Greece; s.iliakopoulou@inn.demokritos.gr
[5] Institute of Nanoscience & Nanotechnology, NCSR Demokritos, Agia Paraskevi, Greece;
[6] Laboratory of Organic Micropollutants, Water Quality Control Department, EYDAP SA, Menidi, Athens, Greece; kaloudis@eydap.gr

* Correspondence: hanna.mazur-marzec@ug.edu.pl;

Table S1. Cyanopeptolin variants described so far.

| Name | Mass | Cyanobacterium | strain | 1 | 2 | 3 | 4 | 5 | 6 | Side chain | References |
|---|---|---|---|---|---|---|---|---|---|---|---|
| Tasipeptin B | 770.5 | *Symploca* | NIH304 | O-Thr | Leu | Ahp | Leu | N-MePhe | Val | BA | [30] |
| Micropeptin MZ771 | 771.5 | *Microcystis* | TAU IL-361 | O-Thr | Arg | O-MeAhp | Ile | N-MePhe | Ile | | [47] |
| Micropeptin MM836 | 836.4 | *Microcystis* | TAU IL-36 | O-Thr | Leu | Ahp | Phe | N-MePhe | Ile | GA | [42] |
| Planktopeptin BL843 | 843.4 | *Oscillatoria/ Planktothrix rubescens* | *n.a.* | O-Thr | Leu | Ahp | Thr | N-MeTyr | Ile | GA-gamma-lactam | [45] |
| Micropeptin MZ845 | 845.5 | *Microcystis* | TAU IL-361 | O-Thr | Arg | Ahp | Ile | N-MePhe | Ile | GA | [47, 72] |
| Micropeptin MM850 | 850.4 | *Microcystis* | TAU IL-36 | O-Thr | Leu | Ahp | Phe | N-MePhe | Ile | GA | [42] |
| Micropeptin MZ859 | 859.5 | *Microcystis* | TAU IL-361 | O-Thr | Arg | O-MeAhp | Ile | N-MePhe | Ile | GA | [47] |
| Tasipeptin A | 869.5 | *Symploca* | NIH304 | O-Thr | Leu | Ahp | Leu | N-MePhe | Val | Val-BA | [30] |
| Cyanopeptolin 880 | 880.5 | *Oscillatoria/ Planktothrix agardhii* | CYA 126/8 | O-Thr | Hty | Ahp | Ile | N-MePhe | Ile | 2-O-MeGa | [25] |
| Micropeptin 88A | 883.5 | *Microcystis aeruginosa* | NIES-88 | O-Thr | H4Tyr | Ahp | Val | N-MePhe | Ile | Glu | [63] |
| Micropeptin HU895B | 895.4 | *Microcystis* | TAU IL-342 | O-Thr | Arg | Ahp | Ile | N-MeTyr | Ile | GA | [73] |
| Micropeptin HU895A | 895.4 | *Microcystis* | TAU IL-342 | O-Thr | Arg | Ahp | Ile | N,O-diMe-Cl-Tyr | Val | GA | [73] |
| Dolastatin 13 | 905.5 | *Dolabella auricularia* | *n.a.* | O-Thr | Dhb | Ahp | Phe | N-MePhe | Val | Val-O-Me_Ga | [32] |
| Micropeptin HU909 | 909.4 | *Microcystis* | TAU IL-342 | O-Thr | Arg | O-MeAhp | Ile | N-Me-Cl-Tyr | Val | GA | [73] |
| Micropeptin SF909 | 909.4 | *n.a.* | *n.a.* | O-Thr | Gln | Ahp | Leu | N-MeTyr | Ile | Hpla | [21, 74] |
| Micropeptin LH911B | 911.4 | *Microcystis* | TAU IL-37 | O-Thr | Arg | Ahp | Val | N-MePhe | Ile | SuGA | [75] |
| Micropeptin LH911A | 911.4 | *Microcystis* | TAU IL-37 | O-Thr | Arg | Ahp | Ile | N-MePhe | Val | SuGA | [72, 75] |
| Cyanopeptolin 911 | 911.4 | *n.a.* | *n.a.* | O-Thr | Arg | Ahp | Leu | N-MeTyr | Val | SuGA | [76] |
| Micropeptin LH911C | 911.4 | *Microcystis* | TAU IL-37 | O-Thr | Me-Lys | Ahp | Ile | N-MePhe | Ile | SuGA | [75] |
| Micropeptin MM916 | 916.4 | *Microcystis* | TAU IL-36 | O-Thr | Leu | Ahp | Phe | N-MePhe | Ile | SuGA | [42] |
| Somamide B | 918.4 | *n.a.* | *n.a.* | O-Thr | Dhb | Ahp | Phe | N-MeTyr | Val | Gln-BA | [69] |
| Nostopeptin BN920 | 920.5 | *Nostoc/Microcystis aeruginosa* | TAU IL-235/NIVA Cya 43 | O-Thr | Leu | Ahp | Phe | N-MeTyr | Val | GlnAc | [10, 40] |
| Micropeptin LH920 | 920.5 | *Microcystis* | TAU IL-37 | O-Thr | Gln | Ahp | Phe | N-MeTyr | Val | Gly-HA | [75] |
| Cyanopeptolin 920 | 920.5 | *n.a.* | *n.a.* | O-Thr | Lys | Ahp | Phe | N-MeTyr | Val | Leu-Ac | [76] |
| Micropeptin MZ925 | 925.4 | *Microcystis* | TAU IL-361 | O-Thr | Arg | Ahp | Ile | N-MePhe | Ile | SuGA | [47, 72] |
| Cyanopeptolin S | 925.4 | *n.a.* | *n.a.* | O-Thr | Arg | Ahp | Ile | N-MeTyr | Val | SuGa | [42, 77] |
| Micropeptin LH925 | 925.4 | *Microcystis* | TAU IL-37 | O-Thr | di-Me-Lys | Ahp | Ile | N-MePhe | Ile | SuGA | [75] |
| Nostopeptin B | 926.5 | *Nostoc minutum* | NIES-26 | O-Hmp | Leu | Ahp | Ile | N-MeTyr | Ile | Gln-AC | [9] |
| Micropeptin KB928 | 928.5 | *Microcystis* | TAU IL-381 | O-Thr | Asp | Ahp | Val | N-MePhe | Ile | Asp-BA | [22] |

| | | | | | | | | | | | |
|---|---|---|---|---|---|---|---|---|---|---|---|
| Cyanopeptolin B | 928.5 | *Microcystis aeruginosa* | PCC7806 | O-Thr | Lys | Ahp | Leu | N-MePhe | Val | Asp-HA | [3] |
| Cyanopeptolin 930 | 930.5 | *Microcystis* | | O-Thr | Lys | Ahp | Leu | N-MeTyr | Val | Glu-BA | [72] |
| Micropeptin MM932 | 932.4 | *Microcystis* | TAU IL-36 | O-Thr | Leu | Ahp | Phe | N-MeTyr | Ile | SuGA | [42] |
| Lyngbyastatin 8 | 932.5 | Lyngbya semiplena | | O-Thr | Abu | Ahp | Phe | N-MeTyr | Val | Val-Ac | [57] |
| precrocapeptin A2 | 933.5 | *Chondromycs crocatus* | Cm c5 | O-Thr | Leu | Ahp | Phe | N-MeTyr | Val | Gln-Ibu | [33] |
| crocapeptin A1 | 935.5 | *Chondromycs crocatus* | Cm c5 | O-Thr | Leu | Ahp | Phe | N-MeTyr | Val | Gln-Propionate | [33] |
| Micropeptin MZ939B | 939.4 | *Microcystis* | TAU IL-361 | O-Thr | Arg | O-MeAhp | Ile | N-MePhe | Ile | 2-SuGA | [47] |
| Micropeptin MZ939A | 939.4 | *Microcystis* | TAU IL-361 | O-Thr | Arg | O-MeAhp | Ile | N-MePhe | Ile | 1-SuGA | [47] |
| Insulapeptolide A | 941.5 | *Nostoc insulare* | SAG 54.79 | O-Hmp | Leu | Ahp | Ile | N-MeTyr | Val | Ac-Cit | [49] |
| Cyanopeptolin C | 942.5 | *Microcystis aeruginosa* | PCC7806 | O-Thr | Me-Lys | Ahp | Leu | N-MePhe | Val | Asp-HA | [3] |
| Micropeptin SD944 | 944.5 | *Microcystis aeruginosa* | TAU IL-215 | O-Thr | Lys | Ahp | Ile | N-MeTyr | Val | Asp-HA | [78] |
| Micropeptin KT946 | 946.4 | *Microcystis* | MB-K | O-Thr | Hty | Ahp | Ile | N-MePhe | Ile | Su-GA | [24] |
| Lyngbyastatin 7 | 946.5 | *Lyngbya semiplena* | *n.a.* | O-Thr | Abu | Ahp | Phe | N-MeTyr | Val | Gln-Ha | [58] |
| Streptopeptolin | 947.4 | Streptomyces olivochromogenes | NBRC 3561 | O-Thr | Gln | Ahp | Phe | N-MeTyr | Ala | Gln-Mba | [34] |
| Kempopeptin C | 948.5 | *n.a.* | *n.a.* | O-Thr | Lys | Ahp | Leu | N,O-diMe-Cl-Tyr | Val | Val-BA | [52] |
| crocapeptin A2 | 949.5 | *Chondromycs crocatus* | Cm c5 | O-Thr | Leu | Ahp | Phe | N-MeTyr | Val | Gln-Ibu | [33] |
| Anabaenopeptilide 90A | 952.5 | *Anabaena* | strain 90 | O-Thr | Hty | Ahp | Thr | N,O-diMeTyr | Ile | N-formyl-Gln | [5] |
| Cyanopeptolin 954 | 954.4 | *Microcystis aeruginosa* | NIVA Cya 43 | O-Thr | Leu | Ahp | Phe | N-Me-Cl-Tyr | Val | GlnAc | [40] |
| Nostopeptin A | 954.5 | *Nostoc minutum* | NIES-26 | O-Hmp | Leu | Ahp | Ile | N-MeTyr | Ile | Gln-BA | [9] |
| Insulapeptolide B | 955.5 | *Nostoc insulare* | SAG 54.79 | O-Hmp | Leu | Ahp | Ile | N-MeTyr | Leu | Ac-Cit | [49] |
| Insulapeptolide C | 955.5 | *Nostoc insulare* | SAG 54.79 | O-Hmp | Leu | Ahp | Ile | N,O-diMeTyr | Val | Ac-Cit | [49] |
| Micropeptin KB956 | 956.5 | *Microcystis* | TAU IL-381 | O-Thr | Asp | O-MeAhp | Val | N-MePhe | Ile | O-Me-Asp-BA | [22] |
| Cyanopeptolin A | 956.5 | *Microcystis aeruginosa* | PCC7806 | O-Thr | Arg | Ahp | Leu | N-MePhe | Val | Asp-HA | [3] |
| Cyanopeptolin D | 956.6 | *Microcystis aeruginosa* | PCC7806 | O-Thr | di-Me-Lys | Ahp | Leu | N-MePhe | Val | Asp-HA | [3] |
| Cyanopeptolin 958 | 958.5 | *Microcystis* | | O-Thr | Arg | Ahp | Leu | N-MeTyr | Val | Glu-BA | [72] |
| Micropeptin B | 958.5 | *Microcystis aeruginosa* | NIES-100 | O-Thr | Lys | Ahp | Leu | N-MeTyr | Val | Glu-HA | [37] |
| Cyanopeptolin 959 | 959.4 | *n.a.* | *n.a.* | O-Thr | Arg | Ahp | Phe | N-MeTyr | Ile | SuGA | [76] |

| | | | | | | | | | | | |
|---|---|---|---|---|---|---|---|---|---|---|---|
| Cyanopeptolin 960 | 960.4 | *Oscillatoria/ Planktothrix agardhii* | CYA 126/8 | O-Thr | Hty | Ahp | Ile | N-MePhe | Ile | 2-O-MeSuGa | [25] |
| Lyngbyastatin 9 | 960.5 | Lyngbya semiplena | | O-Thr | Abu | Ahp | Phe | N-MeTyr | Val | Val-BA | [57] |
| Bouillomide A | 960.5 | *Lyngbya bouillonii* | *n.a.* | O-Thr | Dhb | Ahp | Phe | N-MeTyr | Val | Leu-Ala-BA | [19] |
| Micropeptin HH960 | 960.5 | *Microcystis aeruginosa* | TAU IL-347 | O-Thr | Leu | Ahp | Leu | N-MePhe | Val | Asn-Hpla | [79] |
| Micropeptin 90 | 961.4 | *Microcystis aeruginosa* | NIES-90 | O-Thr | Arg | Ahp | Phe | N-MeTyr | Val | SuGA | [61] |
| Molassamide | 962.5 | *Leptolyngbya sp./Dichothrix utahensis* | DRTO-73 | O-Thr | Abu | Ahp | Phe | N-MeTyr | Val | Thr-Ala-BA | [6, 52] |
| Cyanopeptolin CP962 | 962.5 | *Nostoc edaphicum* | CCNP1411 | O-Thr | Arg | Ahp | Phe | N-MePhe | Val | Asp-BA | [8] |
| Cyanopeptolin 963A | 963.5 | *Microcystis aeruginosa* | PCC 7806 | O-Thr | Tyr | Ahp | Leu | N-MePhe | Val | Glu-HA | [80] |
| crocapeptin A3 | 963.5 | *Chondromycs crocatus* | Cm c5 | O-Thr | Leu | Ahp | Phe | N-MeTyr | Val | Gln-2-Me-Ibu | [33] |
| Micropeptin EI964 | 964.5 | *Microcystis aeruginosa* | TAU IL-217; TAU IL-231 | O-Thr | Arg | Ahp | Phe | N-MeTyr | Ile | Asp-AC | [10] |
| Somamide A | 965.5 | *n.a.* | *n.a.* | O-Thr | Dhb | Ahp | Phe | N-MeTyr | Val | Met-O-HA | [69] |
| Cyanopeptolin CP969 | 969.4 | *Nostoc edaphicum* | CCNP1411 | O-Thr | Tyr | Ahp | Phe | N-MePhe | Val | Asp-BA | [8] |
| Micropeptin KB970B | 970.5 | *Microcystis* | TAU IL-381 | O-Thr | Asp | O-MeAhp | Val | N-MePhe | Ile | Asp-HA | [22] |
| Micropeptin KB970A | 970.5 | *Microcystis* | TAU IL-381 | O-Thr | Asp | Ahp | Val | N-MePhe | Ile | O-Me-Asp-HA | [22] |
| Micropeptin KB970C | 970.5 | *Microcystis* | TAU IL-381 | O-Thr | Asp | O-MeAhp | Val | N-MePhe | Val | O-Me-Asp-HA | [22] |
| Anabaenopeptilide 90B | 972.4 | *Anabaena* | strain 90 | O-Thr | Hty | Ahp | Thr | N-Me-Cl-Tyr | Ile | N-formyl-Gln | [5] |
| Micropeptin 973 | 972.5 | *Microcystis panniformis* | MIRS-04 | O-Thr | Arg | Ahp | Ile | N-MePhe | Val | Gln-BA | [65] |
| Cyanopeptolin 972 | 972.5 | *n.a.* | *n.a.* | O-Thr | Arg | Ahp | Leu | N-MeTyr | Val | Asp-HA | [72, 81] |
| Stigonemapeptin | 973.5 | *Stigonema* | | O-Thr | Abu | Ahp | Phe | N-MeTyr | Val | Gln-N-formyl-Pro | [15] |
| Loggerpeptin C | 974.5 | *Leptolyngbya* | DRTO-73 | O-Thr | Leu | Ahp | Phe | N-MeTyr | Val | Abu-Ala-BA | [52] |
| Micropeptin 478A | 975.4 | *Microcystis aeruginosa* | NIES-478 | O-Thr | Arg | Ahp | Ile | N-Me-Cl-Tyr | Ile | SuGA | [62] |
| Micropeptin HU975 | 975.4 | *Microcystis* | TAU IL-342 | O-Thr | Arg | Ahp | Ile | N-Me-Cl-Tyr | Ile | SuGA | [73] |
| Insulapeptolide D | 976.6 | *Nostoc insulare* | SAG 54.79 | O-Hmp | Leu | Ahp | Ile | N,O-diMeTyr | Leu | Ac-Cit | [49] |
| Cyanopeptolin CP978 | 977.5 | *Nostoc edaphicum* | CCNP1411 | O-Thr | Arg | Ahp | Phe | N-MeTyr | Val | Asp-BA | [8] |
| Micropeptin MM978 | 978.5 | *Microcystis* | TAU IL-36 | O-Thr | Gln | Ahp | Phe | N-MeTyr | Ile | Glu-HA | [42] |
| Micropeptin HM978 | 978.5 | *Microcystis* | TAU IL-33 | O-Thr | Leu | Ahp | Ile | N-MePhe | Val | Asn-Hpla | [82] |
| Micropeptin HH978 | 978.5 | *Microcystis aeruginosa* | TAU IL-347 | O-Thr | Leu | Ahp | Leu | N-MePhe | Val | Asn-Hpla | [79] |
| Micropeptin SD979 | 979.5 | *Microcystis aeruginosa* | TAU IL-215 | O-Thr | Tyr | Ahp | Ile | N-MeTyr | Val | Asp-HA | [78] |

| | | | | | | | | | | | |
|---|---|---|---|---|---|---|---|---|---|---|---|
| Micropeptin GH979 | 979.5 | *Microcystis* | TAU IL-33 | O-Thr | Leu | Ahp | Ile | N-MePhe | Val | Asp-Hpla | [82] |
| Scyptolin A | 980.5 | *Scytonema hofmannii* | PCC 7110 | O-Thr | Leu | Ahp | Thr | N-Me-Cl-Tyr | Val | Thr-Ala-BA | [13] |
| Cyanopeptolin 984 | 983.4 | *Microcystis wesenbergii* | NIVA-CYA 172/5 | O-Thr | Leu | Ahp | Phe | N-Me-Cl-Tyr | Gln | GlnAC | [83] |
| Micropeptin HA983 | 983.4 | *Microcystis* | TAU IL-33 | O-Thr | Glu | Ahp | Phe | N-Me-Cl-Tyr | Ile | GlnAC | [82] |
| Cyanopeptolin 983 | 983.5 | *n.a.* | *n.a.* | O-Thr | Leu | Ahp | Val | N,O-diMeTyr | Val | Gln-ProAc | [84] |
| Micropeptin KB984 | 984.6 | *Microcystis* | TAU IL-381 | O-Thr | Asp | O-MeAhp | Val | N-MePhe | Ile | O-Me-Asp-HA | [22] |
| Cyanopeptolin CP985 | 985.4 | *Nostoc edaphicum* | CCNP1411 | O-Thr | Tyr | Ahp | Phe | N-MeTyr | Val | Asp-BA | [8] |
| Micropeptin K139 | 986.5 | *Radiocystis feernandoi* | *n.a.* | O-Thr | Arg | Ahp | Ile | N-MeTyr | Ile | Asp-HA | [29] |
| Cyanopeptolin 986 | 986.5 | *n.a.* | *n.a.* | O-Thr | Arg | Ahp | Leu | N-MeTyr | Val | Glu-HA | [72] |
| Micropeptin A | 986.6 | *Microcystis aeruginosa* | NIES-100 | O-Thr | Lys | Ahp | Leu | N-MeTyr | Val | Glu-OA | [37, 72] |
| Micropeptin HU989 | 989.4 | *Microcystis* | TAU IL-342 | O-Thr | Arg | Ahp | Ile | N,O-diMe-Cl-Tyr | Ile | SuGA | [73] |
| Insulapeptolide G | 990.5 | *Nostoc insulare* | SAG 54.79 | O-Thr | Phe | Ahp | Thr | N-MePhe | Val | Ser-Pro-BA | [49] |
| Kempopeptin A | 990.5 | *Lyngbya* | *n.a.* | O-Thr | Leu | Ahp | Phe | N-MeTyr | Val | Thr-Pro | [28] |
| Cyanopeptolin CP990 | 990.5 | *Nostoc edaphicum* | CCNP1411 | O-Thr | Arg | Ahp | Phe | N-MePhe | Val | Asp-HA | [8] |
| crocapeptin B | 990.5 | *Chondromycs crocatus* | Cm c5 | O-Thr | Leu | Ahp | Phe | N-MeTyr | Ile | Cit-Ibu | [33] |
| Cyanopeptolin 991 | 991.4 | *Microcystis* | | O-Thr | Arg | Ahp | Leu | N-MePhe | Val | diSuGA | [72] |
| Kempopeptin B | 992.5 | *Lyngbya* | *n.a.* | O-Thr | Lys | Ahp | Ile | N,O-diMe-Br-Tyr | Val | Val-BA | [28] |
| Micropeptin EI992 | 992.5 | *Microcystis aeruginosa* | TAU IL-217; TAU IL-231 | O-Thr | Arg | Ahp | Phe | N-MeTyr | Ile | Asp-BA | [10] |
| Cyanopeptolin 992 | 992.5 | *Microcystis* | | O-Thr | Lys | Ahp | Phe | N-MeTyr | Val | Glu-HA | [72] |
| Cyanopeptolin CP992 | 992.5 | *Nostoc edaphicum* | CCNP1411 | O-Thr | Arg | Ahp | Phe | N,O-diMeTyr | Val | Asp-BA | [8] |
| Loggerpeptin A | 992.5 | *Leptolyngbya* | DRTO-73 | O-Thr | Leu | Ahp | Phe | N-MeTyr | Val | Thr-Ala-BA | [52] |
| Micropeptin KB992 | 992.5 | *Microcystis* | TAU IL-381 | O-Thr | Leu | O-MeAhp | Ile | N-MePhe | Val | Asn-Hpla | [22] |
| Micropeptin HH992 | 992.5 | *Microcystis aeruginosa* | TAU IL-347 | O-Thr | Leu | Ahp | Leu | N-MePhe | Val | Asn-Hpla | [79] |
| Micropeptin E | 993.5 | *Microcystis aeruginosa* | NIES-100 | O-Thr | Tyr | Ahp | Leu | N-MeTyr | Val | Glu-HA | [20, 72] |
| Micropeptin SF995 | 995.5 | *n.a.* | *n.a.* | O-Thr | Arg | Ahp | Ile | N-MeTrp | Val | Asp-HA | [74] |
| Micropeptin 996 | 996.5 | *Microcystis aeruginosa* | UTEX LB2386 | O-Thr | Hty | Ahp | Phe | N-MePhe | Val | Gln-BA | 66 |
| Cyanopeptolin 997 B | 997.5 | *n.a.* | *n.a.* | O-Thr | Leu | Ahp | Val | N,O-diMeTyr | Leu | Gln-ProAc | [84] |
| Cyanopeptolin 997 A | 997.5 | *n.a.* | *n.a.* | O-Thr | Leu | Ahp | Leu | N-MeTyr | Leu | Gln-ProAc | [84] |
| Cyanopeptolin 997 C | 997.5 | *n.a.* | *n.a.* | O-Thr | Leu | Ahp | Leu | N,O-diMeTyr | Val | Gln-ProAc | [84] |
| Micropeptin KR998 | 998.5 | *Microcystis aeruginosa* | TAU IL-40 | O-Thr | Tyr | Ahp | Phe | N-MeTyr | Val | Gln-BA | [85] |
| Cyanopeptolin CP999 | 999.5 | *Nostoc edaphicum* | CCNP1411 | O-Thr | Tyr | Ahp | Phe | N,O-diMeTyr | Val | Asp-BA | [8] |

| | | | | | | | | | | | |
|---|---|---|---|---|---|---|---|---|---|---|---|
| Micropeptin SD999 | 999.5 | *Microcystis aeruginosa* | TAU IL-215 | O-Thr | Arg | Ahp | Ile | N-Me-Kyn | Val | Asp-HA | [78] |
| Kyanamide | 1000.2 | *Caldora penicillata* | | O-Thr | Leu | Ahp | Phe | N-MeTrp | Val | Gln-HA | [4] |
| Cyanopeptolin 1000A | 1000.6 | *n.a.* | *n.a.* | O-Thr | Lys | Ahp | Leu | N-MeTyr | Ile | Glu-OA | [76] |
| Cyanopeptolin 1001 | 1001.5 | *n.a.* | *n.a.* | O-Thr | Leu | Ahp | Leu | N-Me-O-Cl-Tyr | Leu | Gln-Mdhp | [84] |
| Micropeptin KR 1002 | 1002.5 | *Microcystis aeruginosa* | TAU IL-40 | O-Thr | H4Tyr | Ahp | Phe | N-MeTyr | Val | Gln-BA | [85] |
| Micropeptin SD1002 | 1002.5 | *Microcystis aeruginosa* | TAU IL-215 | O-Thr | Tyr | Ahp | Ile | N-MeTrp | Val | Asp-HA | [78] |
| Cyanopeptolin 1003 | 1003.5 | *n.a.* | *n.a.* | O-Thr | Leu | Ahp | Val | N-Me-O-Cl-Tyr | Val | Gln-ProAc | [84] |
| Insulapeptolide H | 1004.5 | *Nostoc insulare* | SAG 54.79 | O-Thr | Phe | Ahp | Thr | N-MePhe | Leu | Ser-Pro-BA | [49] |
| Cyanopeptolin SS | 1005.4 | *n.a.* | *n.a.* | O-Thr | *??* | Ahp | ?? | N-MeTyr | ?? | ?? | [42, 49] |
| Insulapeptolide F | 1006.5 | *Nostoc insulare* | SAG 54.79 | O-Thr | Phe | Ahp | Thr | N-MeTyr | Val | Ser-Pro-BA | [49] |
| Cyanopeptolin 1007 MB2 | 1006.5 | *Microcystis* | | O-Thr | Lys | Ahp | Leu | N-Me-Cl-Tyr | Ile | Glu-HA | [86] |
| Cyanopeptolin CP1006 | 1006.5 | *n.a./Nostoc edaphicum* | n.a./CCNP1411 | O-Thr | Arg | Ahp | Phe | N-MeTyr | Val | Asp-HA | [8, 72, 81] |
| Cyanopeptolin 1006D | 1006.5 | *n.a.* | *n.a.* | O-Thr | Lys | Ahp | Phe | N-MeTyr | Ile | Glu-HA | [76] |
| Micropeptin DR1006 | 1006.5 | *Microcystis aeruginosa* | TAU IL-237 and IL-23 | O-Thr | Leu | Ahp | Leu | N-MePhe | Ile | Gln-Hpla | [21] |
| Cyanopeptolin 1009 | 1009.4 | *n.a.* | *n.a.* | O-Thr | Arg | Ahp | Cl-Phe | N-MeTyr | Ile | SuGA | [81] |
| Micropeptin T20 | 1010.3 | *Microcystis aeruginosa* | *n.a.* | O-Thr | Phe | Ahp | Phe | N-MeTyr | Ile | GA-Na2H2PO3 | [39] |
| Cyanopeptolin 1011 | 1011.6 | *n.a.* | *n.a.* | O-Thr | Leu | Ahp | Leu | N,O-diMeTyr | Leu | Gln-ProAc | [84] |
| Cl-Cyanopeptolin W | 1012.4 | *n.a.* | *n.a.* | O-Thr | Arg | Ahp | Phe | N-Me-Cl-Tyr | Ile | GluAc | [81] |
| Cyanopeptolin CP1013 | 1013.5 | *Nostoc edaphicum* | CCNP1411 | O-Thr | Tyr | Ahp | Phe | N-MeTyr | Val | Asp-HA | [8] |
| Cyanopeptolin 1014 | 1014.6 | *n.a.* | *n.a.* | O-Thr | Arg | Ahp | Leu | N-MeTyr | Val | Glu-OA | [72] |
| Micropeptin T2 | 1015.5 | *n.a.* | *n.a.* | O-Thr | Lys | Ahp | Phe | N-MeTrp | Val | Glu-HA | [38] |
| Cyanopeptolin 1017 | 1017.5 | *n.a.* | *n.a.* | O-Thr | Leu | Ahp | Val | N-Me-O-Cl-Tyr | Leu | Gln-ProAc | [84] |
| Cyanopeptolin CP1018 | 1018.5 | *Nostoc edaphicum* | CCNP1411 | O-Thr | Arg | Ahp | Phe | N-MePhe | Val | Asp-OA | [8] |
| Micropeptin MZ1019 | 1019.4 | *Microcystis* | TAU IL-361 | O-Thr | Arg | O-MeAhp | Ile | N-MePhe | Ile | 1,2-SuGA | 47 |
| Cyanopeptolin 1020B | 1020.5 | *Microcystis* | | O-Thr | Lys | Ahp | Leu | N-MeTyr | Val | Glu-OA | [72] |
| Insulapeptolide E | 1020.5 | *Nostoc insulare* | SAG 54.79 | O-Thr | Phe | Ahp | Thr | N-MeTyr | Leu | Ser-Pro-BA | [49] |
| Cyanopeptolin 1020 | 1020.5 | *Microcystis aeruginosa* | UV006 | O-Thr | Arg | Ahp | Phe | N-MeTyr | Val | Glu-HA | [35, 72] |
| Cyanopeptolin CP1020 | 1020.5 | *Nostoc edaphicum* | CCNP1411 | O-Thr | Arg | Ahp | Phe | N,O-diMeTyr | Val | Asp-HA | [8] |
| Micropeptin HU1021 | 1021.4 | *Microcystis* | TAU IL-342 | O-Thr | Arg | Ahp | Ile | N-MeTyr | Ile | diSuGA | [73] |
| Micropeptin LH1021 | 1021.5 | *Microcystis* | TAU IL-37 | O-Thr | Gln | Ahp | Phe | N-MeTyr | Val | Thr-Gly-HA | [75] |
| Micropeptin F | 1021.5 | *Microcystis aeruginosa* | NIES-100 | O-Thr | Tyr | Ahp | Leu | N-MeTyr | Val | Glu-OA | [20, 72] |
| Aeruginopeptin 917SC | 1022.5 | *n.a.* | *n.a.* | O-Thr | Leu | Ahp | Leu | N-MeTyr | Ile | Hpla-Gln | [55] |

| | | | | | | | | | | | |
|---|---|---|---|---|---|---|---|---|---|---|---|
| Cyanopeptolin 1025 | 1025.6 | *n.a.* | *n.a.* | O-Thr | Leu | Ahp | Leu | N,O-diMeTyr | Leu | Gln-PrPro | [84] |
| Cyanopeptolin CP1027 | 1027.5 | *Nostoc edaphicum* | CCNP1411 | O-Thr | Tyr | Ahp | Phe | N,O-diMeTyr | Val | Asp-HA | [8] |
| Micropeptin C | 1027.5 | *Microcystis aeruginosa* | NIES-100 | O-Thr | Tyr | Ahp | Phe | N-MeTyr | Val | Glu-HA | [20] |
| Micropeptin KR1030 | 1030.5 | *Microcystis aeruginosa* | TAU IL-40 | O-Thr | H4Tyr | Ahp | Phe | N-MeTyr | Val | Gln-HA | [85] |
| Cyanopeptolin 1032 | 1031.5 | *n.a.* | *n.a.* | O-Thr | Leu | Ahp | Leu | N,O-diMeTyr | Leu | Gln-PrPro | [84] |
| Oscillapeptilide 97B | 1031.5 | *Oscillatoria/ Planktothrix agardhii* | strain 97 | O-Thr | Leu | Ahp | Phe | N-MeTyr | Ile | Gln-N-AcPro | [27] |
| Cyanopeptolin CB071 | 1034.5 | *Aphanocapsa* | 1001 | O-Thr | Arg | Ahp | Ile | N,O-diMe-Cl-Tyr | Val | Glu-HA | [16] |
| Cyanopeptolin 1034 | 1034.6 | *n.a.* | *n.a.* | O-Thr | Lys | Ahp | Phe | N-MeTyr | Ile | Glu-OA | [76] |
| Lyngbyastatin 10 | 1038.4 | Lyngbya semiplena | | O-Thr | Abu | Ahp | Phe | N-Me-Br-Tyr | Val | Val-BA | [57] |
| Bouillomide B | 10384 | *Lyngbya bouillonii* | *n.a.* | O-Thr | Dhb | Ahp | Phe | N-Me-Br-Tyr | Val | Leu-Ala-BA | [19] |
| Cyanopeptolin 1041 | 1040.5 | *n.a.* | *n.a.* | O-Thr | Arg | Ahp | Phe | N-MeTyr | Val | Glu-HA | [87] |
| A90720A | 1040.5 | *Microchaete loktakensis* | IC-39-2 | O-Thr | Arg | Ahp | Leu | N-MeTyr | Val | Leu-SuGA | [7, 36] |
| Micropeptin HU1041 | 1041.3 | *Microcystis* | TAU IL-342 | O-Thr | Arg | Ahp | Ile | N-Me-Cl-Tyr | Val | diSuGA | [73] |
| Ichthyopeptin A | 1042.5 | *Microcystis ichthyoblade* | BM Mi/13 | O-Thr | Tyr | Ahp | Val | N-MePhe | Ile | Gln-Hpla | [54] |
| Micropeptin KT1042 | 1042.5 | *Microcystis aeruginosa* | TAU IL-347 | O-Thr | Tyr | Ahp | allo-Ile | N-MePhe | Val | Gln-Hpla | [41] |
| Aeruginopeptin 228A | 1044.5 | *Microcystis aeruginosa* | TAC 954; M2285 | O-Thr | H4Tyr | Ahp | Thr | N-MePhe | Ile | Gln-Hpla | [23] |
| Micropeptin 103 | 1044.5 | *Microcystis viridis* | NIES-103 | O-Thr | Gln | Ahp | Phe | N-MeTrp | Val | Thr-Gly-HA | [43] |
| Oscillapeptilide 97A | 1045.5 | *Oscillatoria/ Planktothrix agardhii* | strain 97 | O-Thr | Leu | Ahp | Phe | N,O-diMeTyr | Ile | Gln-N-AcPro | [27] |
| Micropeptin KB1046 | 1046.5 | *Microcystis* | TAU IL-381 | O-Thr | H4Tyr | Ahp | Val | N-MePhe | Ile | Gln-Hpla | [22] |
| Aeruginopeptin 228B | 1048.5 | *Microcystis aeruginosa* | TAC 954; M2285 | O-Thr | H4Tyr | Ahp | Thr | N-MePhe | Ile | Gln-Hpla | [23] |
| Micropeptin KB1048 | 1048.5 | *Microcystis* | TAU IL-381 | O-Thr | Asp | O-MeAhp | Ile | N-Me-Cl-Tyr | Ile | O-Me-Asp-HA | [22] |
| Cyanopeptolin CP1048 | 1048.6 | *Nostoc edaphicum* | CCNP1411 | O-Thr | Arg | Ahp | Phe | N,O-diMeTyr | Val | Asp-OA | [8] |
| Micropeptin LH1048 | 1048.6 | *Microcystis* | TAU IL-37 | O-Thr | Arg | Ahp | Phe | N-MeTyr | Val | Glu-OA | [72, 75] |
| Anabaenopeptilide 202A | 1049.5 | *Anabaena* | strain 202A2 | O-Thr | Hty | Ahp | Thr | N-MeTyr | Ile | Gln-N-formyl-Pro | [5] |
| Symplocamide A | 1050.4 | *Symploca* | PNG-12/15/03-5 | O-Thr | Arg | Ahp | Ile | N,O-diMeTyr | Val | Gln-BA | [31] |
| Micropeptin T1 | 1050.5 | *n.a.* | *n.a.* | O-Thr | Tyr | Ahp | Phe | N-MeTrp | Val | Glu-HA | [38] |
| Cyanopeptolin 1054 MB1 | 1054.5 | *n.a.* | | O-Thr | Arg | Ahp | Phe | N-Me-Cl-Tyr | Ile | Glu-HA | [86] |

| | | | | | | | | | | | |
|---|---|---|---|---|---|---|---|---|---|---|---|
| Cyanopeptolin 1054 MB2 | 1054.5 | *n.a.* | | O-Thr | Me-Lys | Ahp | Phe | N-Me-Cl-Tyr | Ile | Glu-HA | [86] |
| Micropeptin 478B | 1055.3 | *Microcystis aeruginosa* | NIES-478 | O-Thr | Arg | Ahp | Ile | N-Me-Cl-Tyr | Ile | diSuGA | [62] |
| Micropeptin D | 1055.5 | *Microcystis aeruginosa* | NIES-100 | O-Thr | Tyr | Ahp | Phe | N-MeTyr | Val | Glu-OA | [20] |
| Lyngbyastatin 5 | 1056.5 | *Lyngbya* | *n.a.* | O-Thr | Abu | Ahp | Phe | N-MeTyr | Val | Hty-Ala-Ga | [58] |
| Micropeptin DR1056 | 1056.5 | *Microcystis aeruginosa* | TAU IL-237 and IL-23 | O-Thr | Tyr | Ahp | Leu | N-MePhe | Ile | Gln-Hpla | [21] |
| Hofmannolin | 1057.5 | *Scytonema hofmannii* | PCC 7110 | O-Thr | Phe | Ahp | O-MeTyr | N-MeTyr | Val | Glu-Hmv | [14] |
| Micropeptin TR1058 | 1058.5 | *Microcystis* | TAU IL-428 | O-Thr | Gln | Ahp | Ile | N-MePhe | Val | Tyr-Hpla | [88] |
| Micropeptin DR1060 | 1060.5 | *Microcystis aeruginosa* | TAU IL-237 and IL-23 | O-Thr | H4Tyr | Ahp | Leu | N-MePhe | Ile | Gln-Hpla | [21] |
| Planktopeptin BL1061 | 1061.6 | *Oscillatoria/ Planktothrix rubescens* | *n.a.* | O-Thr | Leu | Ahp | Thr | N-MeTyr | Ile | Gln-Leu-GA | [45] |
| Oscillapeptin C | 1061.6 | *Oscillatoria/ Planktothrix agardhii* | NIES-205 | O-Thr | H4Tyr | Ahp | Ile | N-MePhe | Ile | Hty-OMeGa | [28] |
| Micropeptin 88N | 1062.6 | *Microcystis aeruginosa* | NIES-88 | O-Thr | Tyr | Ahp | Val | N-MePhe | Ile | Glu-Leu-BA | [64] |
| Micropeptin 88E | 1062.6 | *Microcystis aeruginosa* | NIES-88 | O-Thr | Leu | Ahp | Val | N-MePhe | Ile | Glu-Tyr-BA | [63] |
| Micropeptin LH1062 | 1062.6 | *Microcystis* | TAU IL-37 | O-Thr | Arg | Ahp | Phe | N-MeTyr | Val | MeO-Glu-OA | [75] |
| Cyanopeptolin 1063 | 1063.6 | *n.a.* | *n.a.* | O-Thr | Tyr | Ahp | Ile | N-MeTyr | Ile | Gln-LeuAc | [76] |
| Pompanopeptin A | 1068.4 | *Lyngbya confervoides* | *n.a.* | O-Thr | Arg | Ahp | Ile | N,O-diMe-Br-Tyr | Val | Met(O)-Ba | [68] |
| Cyanopeptolin 1068 | 1068.5 | *Microcystis aeruginosa* | *n.a.* | O-Thr | di-Me-Lys | Ahp | Phe | N-Me-Cl-Tyr | Ile | Glu-HA | [86] |
| Micropeptin HU1069 | 1069.4 | *Microcystis* | TAU IL-342 | O-Thr | Arg | Ahp | Ile | N,O-diMe-Cl-Tyr | Ile | diSuGA | [73] |
| Anabaenopeptilide 202B | 1069.5 | *Anabaena* | strain 202A2 | O-Thr | Hty | Ahp | Thr | N-Me-Cl-Tyr | Ile | Gln-N-formyl-Pro | [5] |
| Aeruginopeptin 917SA | 1072.5 | *Microcystis* | n.a. | O-Thr | Tyr | Ahp | Leu | N-MeTyr | IIe | Gln-Hpla | [55] |
| Microcystilide A | 1072.5 | *Microcystis aeruginosa* | NO-15-1840 | O-Thr | Tyr | Ahp | Leu | N-MeTyr | Ile | Gln-Hpla | [59] |
| Aeruginopeptin 917SB | 1076.5 | *n.a.* | *n.a.* | O-Thr | *H4Tyr* | *Ahp* | Leu | N-MeTyr | Ile | Hpla-Gln | [55] |
| Micropeptin 88B | 1078.5 | *Microcystis aeruginosa* | NIES-88 | O-Thr | Glu | Ahp | Val | N-MePhe | Ile | Glu-Tyr-BA | [63] |
| Micropeptin 88Y | 1084.5 | *Microcystis aeruginosa* | NIES-88 | O-Thr | Tyr | Ahp | Val | N-MePhe | Ile | Glu-TyrAc | [64] |

| | | | | | | | | | | | |
|---|---|---|---|---|---|---|---|---|---|---|---|
| Oscillapeptin F | 1088.5 | *Oscillatoria/ Planktothrix agardhii* | NIES-596 | O-Thr | Lys | Ahp | Ile | N-MePhe | Ile | Hty-O-Me-SuGA | [28] |
| Oscillapeptin J | 1092.4 | *Oscillatoria/ Planktothrix rubescens* | | O-Thr | Arg | Ahp | Thr | N-MeTyr | Ile | Tyr-SuGA | [67] |
| Jizanpeptin A | 1104.4 | *n.a.* | *n.a.* | O-Thr | Lys | Ahp | Ile | N,O-diMe-Br-Tyr | Ile | Val-SuGA | [56] |
| Micropeptin 1106 | 1105.6 | *Microcystis aeruginosa* | *n.a.* | O-Thr | Arg | Ahp | Val | N-MeTyr | Ile | Glu-Tyr-BA | [60] |
| Oscillapeptin G | 1111.5 | *Oscillatoria/ Planktothrix agardhii* | | O-Thr | Leu | Ahp | Thr | N-MeTyr | Ile | Gln-Hty-GA | [35] |
| Micropeptin 88C | 1112.5 | *Microcystis aeruginosa* | NIES-88 | O-Thr | Tyr | Ahp | Val | N-MePhe | Ile | Glu-Tyr-BA | [63] |
| Micropeptin 88F | 1112.5 | *Microcystis aeruginosa* | NIES-88 | O-Thr | Tyr | Ahp | Val | N-MePhe | Ile | O-Me-Glu-Tyr-BA | [63] |
| Nostocyclin | 1116.5 | *Nostoc* | DUN901 | O-Thr | Hse | Ahp | Phe | N-MePhe | Val | Hse-Ile-Hpla | [11] |
| Micropeptin 88D | 1116.6 | *Microcystis aeruginosa* | NIES-88 | O-Thr | H4Tyr | Ahp | Val | N-MePhe | Ile | Glu-Tyr-BA | [63] |
| Jizanpeptin B | 1118.4 | *n.a.* | *n.a.* | O-Thr | Lys | Ahp | Ile | N,O-diMe-Br-Tyr | Ile | (R)Val-O-Me-SuGA | [56] |
| Jizanpeptin C | 1118.4 | *n.a.* | *n.a.* | O-Thr | Lys | Ahp | Ile | N,O-diMe-Br-Tyr | Ile | (S)Val-O-Me-SuGA | [56] |
| Micropeptin 1120 | 1119.6 | *Microcystis aeruginosa* | *n.a.* | O-Thr | Arg | Ahp | Val | N-MeTyr | Ile | OMeGlu-Tyr-BA | [60] |
| Scyptolin B | 1121.5 | *Scytonema hofmannii* | PCC 7110 | O-Thr | Leu | Ahp | Thr | N-Me-Cl-Tyr | Val | Ala-BA-Thr-Ala-BA | [13] |
| Planktopeptin BL1125 | 1125.6 | *Oscillatoria/ Planktothrix rubescens* | *n.a.* | O-Thr | Leu | Ahp | Thr | N-MeTyr | Ile | Gln-Hty-GA | [45] |
| Oscillapeptin D | 1127.5 | *Oscillatoria/ Planktothrix agardhii* | NIES-205 | O-Thr | Hty | Ahp | Ile | N-MePhe | Ile | Hty-O-Me-SuGA | [28] |
| Jizanpeptin D | 1132.4 | *n.a.* | *n.a.* | O-Thr | Lys | Ahp | Ile | N,O-diMe-Br-Tyr | Ile | Ile-O-Me-SuGA | [56] |
| Loggerpeptin B | 1133.6 | *Leptolyngbya* | DRTO-73 | O-Thr | Leu | Ahp | Phe | N-MeTyr | Val | Thr-Ala-BA-O-Ala-BA | [52] |
| Lyngbyastatin 4 | 1136.4 | *Lyngbya confervoides* | *n.a.* | O-Thr | Abu | Ahp | Phe | N-Me-Tyr | Val | Htyr-Ala-SuGa | [44] |
| Oscillapeptin E | 1137.5 | *Oscillatoria/ Planktothrix agardhii* | NIES-205 | O-Thr | H4Tyr | Ahp | Ile | N-MePhe | Ile | Hty-O-Me-SuGA | [28, 89] |
| Aeruginopeptin 95A | 1145.5 | *Microcystis aeruginosa* | TAC 954; M2285 | O-Thr | Tyr | Ahp | Thr | N-MePhe | Ile | Gln-Thr-Hpla | [23] |
| Jizanpeptin E | 1146.4 | *n.a.* | *n.a.* | O-Thr | Arg | Ahp | Ile | N,O-diMe-Br-Tyr | Ile | Val-O-Me-SuGA | [56] |

| | | | | | | | | | | | |
|---|---|---|---|---|---|---|---|---|---|---|---|
| Aeruginopeptin 95B | 1149.6 | *Microcystis aeruginosa* | TAC 954; M2285 | O-Thr | H4Tyr | Ahp | Thr | N-MePhe | Ile | Gln-Thr-Hpla | [23] |
| Lyngbyastatin 6 | 1150.5 | *Lyngbya* | *n.a.* | O-Thr | Abu | Ahp | Phe | N-MeTyr | Val | Hty-Ala-Na-SuGa | [58] |
| Oscillapeptin A | 1167.5 | *Oscillatoria/ Planktothrix agardhii* | NIES-204 | O-Thr | Hty | Ahp | Ile | N,O-diMeTyr | Ile | Hty-OMeSuGa | [46] |
| Oscillapeptin B | 1181.5 | *Oscillatoria/ Planktothrix agardhii* | NIES-204 | O-Thr | Me-Tyr | Ahp | Ile | N,O-diMeTyr | Ile | Hty-O-Me-SuGA | [28] |
| Largamide E | 1187.6 | *Oscillatoria/ Planktothrix* | FL31-01 | O-Thr | Tyr | Ahp | Thr | N-Me-Cl-Tyr | Val | Val-Ala-Ahppa-GA | [26] |
| Largamide D oxazolidine | 1213.5 | *Lyngbya confervoides* | *n.a.* | O-Thr | Leu | Ahp | allo-Thr | N-Me-Br-Tyr | Val | Val-Ala-Ahppa-GA | [90] |
| Largamide D | 1231.5 | *Oscillatoria/ Planktothrix* | FL31-01 | O-Thr | Hty | Ahp | Thr | N-Me-Br-Tyr | Val | Val-Ala-Ahppa-GA | [26] |
| Largamide F | 1281.5 | *Oscillatoria/ Planktothrix* | FL31-01 | O-Thr | Leu | Ahp | Thr | N-Me-Br-Tyr | Val | Val-Ala-Ahppa-GA | [26] |
| Largamide G | 1295.5 | *Oscillatoria/ Planktothrix* | FL31-01 | O-Thr | Leu | Ahp | Thr | N-Me-Br-Tyr | Val | Val-Ala-Ahppa-GA | [26] |

Table S2. Cyanopeptolin variants produced by the *Nostoc edaphicum* CCNP1411.

| Group | Name | Exact mass | *(m/z)* calc | *(m/z)* $[M+H]^+$ | Error mass | Retention time | Structure | Mass of isolated CP [mg] | References |
|---|---|---|---|---|---|---|---|---|---|
| CP-$Arg^2$ | CP 777 | 777.4174 | 778.4252 | 778.43 | | | [Thr+Arg+Ahp+Phe+MePhe+Val] | 0.3 | |
| CP-$Arg^2$ | CP 807 | 807.4279 | 808.4357 | 808.44 | | | [Thr+Arg+Ahp+Phe+diMeTyr+Val] | 0..5 | |
| CP-$Arg^2$ | CP 892 | 892.4443 | 893.4521 | 893.4521 | 0.0000 | 5.45 | [Thr+Arg+Ahp+Phe+MePhe+Val]Asp | | |
| CP-$Arg^2$ | CP 922 | 922.4549 | 923.4627 | 923.4635 | 0.0008 | 5.02 | [Thr+Arg+Ahp+Phe+diMeTyr+Val]Asp | | |
| CP-$Arg^2$ | CP 934 | 934.4549 | 935.4627 | 935.4626 | 0.0001 | 6.84 | [Thr+Arg+Ahp+Phe+MePhe+Val]Asp+Ac | 0.3 | |
| CP-$Arg^2$ | CP 944 | 944.4967 | 945.5046 | 945.5050 | 0.0005 | 5.81 | [Thr+Arg+Ahp+Leu+MeTyr+Val]Asp+BA | | |
| CP-$Arg^2$ | CP 950 | 950.4498 | 951.4576 | 951.4577 | 0.0001 | 4.40 | [Thr+Arg+Ahp+Phe+MeTyr+Val]Asp+Ac | 0.4 | |
| CP-$Arg^2$ | CP 962 | 962.4862 | 963.4940 | 963.4939 | 0.0001 | 7.54 | [Thr+Arg+Ahp+Phe+MePhe+Val]Asp+BA | 1.2 | [8] |
| CP-$Arg^2$ | CP 964 | 964.4654 | 965.4733 | 965.4731 | 0.0001 | 6.69 | [Thr+Arg+Ahp+Phe+diMeTyr+Val]Asp+Ac | | |
| CP-$Arg^2$ | CP 976 | 976.5018 | 977.5096 | 977.5074 | 0.0022 | 8.06 | [Thr+Arg+Ahp+Phe+MePhe+Leu]Asp+BA | | |
| CP-$Arg^2$ | CP 978 | 978.4811 | 979.4889 | 979.4889 | 0.0000 | 6.04 | [Thr+Arg+Ahp+Phe+MeTyr+Val]Asp+BA | 4.4 | [8] |
| CP-$Arg^2$ | CP 990 | 990.5175 | 991.5253 | 991.5246 | 0.0007 | 8.51 | [Thr+Arg+Ahp+Phe+MePhe+Val]Asp+HA | 3.9 | [8] |
| CP-$Arg^2$ | CP 992 | 992.4967 | 993.5046 | 993.5043 | 0.0003 | 7.43 | [Thr+Arg+Ahp+Phe+diMeTyr+Val]Asp+BA | 0.3 | [8] |
| CP-$Arg^2$ | CP 992b | 992.4967 | 993.5046 | 993.5047 | 0.0001 | 6.70 | [Thr+Arg+Ahp+Phe+MeTyr+Leu]Asp+BA | | |
| CP-$Arg^2$ | CP 992c | 992.4967 | 993.5046 | 993.50 | | | [Thr+Arg+Ahp+Phe+MeTyr+Val]Glu+BA | | |
| CP-$Arg^2$ | CP 1006 | 1006.5124 | 1007.5202 | 1007.5198 | 0.0004 | 7.33 | [Thr+Arg+Ahp+Phe+MeTyr+Val]Asp+HA | | [76, 8] |
| CP-$Arg^2$ | CP 1008 | 1008.4917 | 1009.4995 | 1009.50 | | | [Thr+Arg+Ahp+Phe+diMe,OTyr+Val]Asp+BA | | |
| CP-$Arg^2$ | CP 1018 | 1018.5488 | 1019.5566 | 1019.5568 | 0.0002 | 9.58 | [Thr+Arg+Ahp+Phe+MePhe+Val]Asp+OA | 1.7 | [8] |
| CP-$Arg^2$ | CP 1020b | 1020.5280 | 1021.5359 | 1021.5350 | 0.0009 | 8.41 | [Thr+Arg+Ahp+Phe+diMeTyr+Val]Asp+HA | 1.2 | [8] |
| CP-$Arg^2$ | CP 1020 | 1020.5280 | 1021.5359 | 1021.54 | | | [Thr+Arg+Ahp+Phe+MeTyr+Val]Glu+HA | | [35] |
| CP-$Arg^2$ | CP 1034 | 1034.5437 | 1035.5515 | 1035.5509 | 0.0006 | 8.51 | [Thr+Arg+Ahp+Phe+MeTyr+Val]Asp+OA | 0.5 | |
| CP-$Arg^2$ | CP 1036 | 1036.5230 | 1037.5308 | 1037.5279 | 0.0029 | 7.48 | [Thr+Arg+Ahp+Phe+diMe,OTyr+Val]Asp+HA | | |
| CP-$Arg^2$ | CP 1046 | 1046.5801 | 1047.5879 | 1047.5870 | 0.0009 | 10.69 | [Thr+Arg+Ahp+Phe+MePhe+Val]Asp+DA | 0.2 | |
| CP-$Arg^2$ | CP 1048 | 1048.5593 | 1049.5672 | 1049.5671 | 0.0001 | 9.47 | [Thr+Arg+Ahp+Phe+diMeTyr+Val]Asp+OA | 1.1 | [8] |
| CP-$Arg^2$ | CP 1076 | 1076.5906 | 1077.5985 | 1077.5988 | 0.0004 | 10.60 | [Thr+Arg+Ahp+Phe+diMeTyr+Val]Asp+DA | | |

| Group | Name | Exact mass | (*m*/*z*) calc | (*m*/*z*) [M+H-$H_2O$]$^+$ | Error mass | Retention time | Structure | Mass of isolated CP [mg] | References |
|---|---|---|---|---|---|---|---|---|---|
| CP-Leu$^{2}$ | CP 891 | 891.4378 | 874.4351 | 874.4342 | 0.0009 | 10.24 | [Thr+Leu+Ahp+Phe+MePhe+Val]Asp+Ac | | |
| CP-Leu$^{2}$ | CP 907 | 907.4327 | 890.4300 | 890.4299 | 0.0001 | 8.26 | [Thr+Leu+Ahp+Phe+MeTyr+Val]Asp+Ac | | |
| CP-Leu$^{2}$ | CP 919 | 919.4691 | 902.4664 | 902.4663 | 0.0001 | 11.35 | [Thr+Leu+Ahp+Phe+MePhe+Val]Asp+BA | 5.1 | |
| CP-Leu$^{2}$ | CP 921 | 921.4484 | 904.4457 | 904.4441 | 0.0015 | 10.00 | [Thr+Leu+Ahp+Phe+diMeTyr+Val]Asp+Ac | | |
| CP-Leu$^{2}$ | CP 933 | 933.4848 | 916.4820 | 916.48 | | | [Thr+Leu+Ahp+Phe+MePhe+Val]Glu+BA | | |
| CP-Leu$^{2}$ | CP 935 | 935.4640 | 918.4613 | 918.4610 | 0.0003 | 9.40 | [Thr+Leu+Ahp+Phe+MeTyr+Val]Asp+BA | 2.1 | |
| CP-Leu$^{2}$ | CP 947 | 947.5004 | 930.4977 | 930.4974 | 0.0003 | 12.69 | [Thr+Leu+Ahp+Phe+MePhe+Val]Asp+HA | | |
| CP-Leu$^{2}$ | CP 949 | 949.4797 | 932.4770 | 932.4764 | 0.0005 | 11.11 | [Thr+Leu+Ahp+Phe+diMeTyr+Val]Asp+BA | 4.6 | |
| CP-Leu$^{2}$ | CP 963b | 963.4953 | 946.4926 | 946.4920 | 0.0006 | 11.79 | [Thr+Leu+Ahp+Phe+diMeTyr+Val]Glu+BA | | |
| CP-Leu$^{2}$ | CP 963 | 963.4953 | 946.4926 | 946.4916 | 0.0010 | 10.85 | [Thr+Leu+Ahp+Phe+MeTyr+Val]Asp+HA | | |
| CP-Leu$^{2}$ | CP 965b | 965.4746 | 948.4719 | 948.47 | | | [Thr+Leu+Ahp+Phe+diMe,OTyr+Val]Asp+BA | | |
| CP-Leu$^{2}$ | CP 975 | 975.5317 | 958.5290 | 958.5276 | 0.0014 | 14.08 | [Thr+Leu+Ahp+Phe+MePhe+Val]Asp+OA | | |
| CP-Leu$^{2}$ | CP 977 | 977.5110 | 960.5083 | 960.5083 | 0.0000 | 12.45 | [Thr+Leu+Ahp+Phe+diMeTyr+Val]Asp+HA | | |
| CP-Leu$^{2}$ | CP 991 | 991.5266 | 974.5239 | 974.5215 | 0.0024 | 12.29 | [Thr+Leu+Ahp+Phe+MeTyr+Val]Asp+OA | | |
| CP-Leu$^{2}$ | CP 1005 | 1005.5423 | 988.5396 | 988.5394 | 0.0001 | 13.84 | [Thr+Leu+Ahp+Phe+diMeTyr+Val]Asp+OA | | |
| CP-MeLeu$^{2}$ | CP 933b | 933.4848 | 916.4820 | 916.48 | | | [Thr+MeLeu+Ahp+Phe+MePhe+Val]Asp+BA | | |
| CP-MeLeu$^{2}$ | CP 949b | 949.4797 | 932.4770 | 932.48 | | | [Thr+MeLeu+Ahp+Phe+MeTyr+Val]Asp+BA | | |
| CP-MePhe$^{2}$ | CP 939b | 939.4378 | 922.4351 | 922.44 | | | [Thr+MePhe+Ahp+Phe+MePhe+Val]Asp+Ac | | |
| CP-MePhe$^{2}$ | CP 967b | 967.4691 | 950.4664 | 950.47 | | | [Thr+MePhe+Ahp+Phe+MePhe+Val]Asp+BA | | |
| CP-MePhe$^{2}$ | CP 1011c | 1011.4953 | 994.4926 | 994.49 | | | [Thr+MePhe+Ahp+Phe+MeTyr+Val]Asp+HA | | |
| CP-Met$^{2}$ | CP 937 | 937.4255 | 920.4228 | 920.42 | | | [Thr+Met+Ahp+Phe+MePhe+Val]Asp+BA | | |
| CP-Met$^{2}$ | CP 939 | 939.4048 | 922.4021 | 922.40 | | | [Thr+Met+Ahp+Phe+diMeTyr+Val]Asp+Ac | | |
| CP-Met$^{2}$ | CP 953b | 953.4205 | 936.4177 | 936.42 | | | [Thr+Met+Ahp+Phe+MeTyr+Val]Asp+BA | | |
| CP-Met$^{2}$ | CP 995 | 995.4674 | 978.4647 | 978.47 | | | [Thr+Met+Ahp+Phe+diMeTyr+Val]Asp+HA | | |
| CP-Met$^{2}$ | CP 1023 | 1023.4987 | 1006.4960 | 1006.50 | | | [Thr+Met+Ahp+Phe+diMeTyr+Val]Asp+OA | | |
| CP-MeTyr$^{2}$ | CP 999d | 999.4590 | 982.4562 | 982.46 | | | [Thr+MeTyr+Ahp+Phe+MeTyr+Val]Asp+BA | | |

| | | | | | | | | | |
|---|---|---|---|---|---|---|---|---|---|
| CP-MeTyr$^2$ | CP 1014 | 1014.1269 | 997.1241 | 997.1572 | 0.0331 | 16.64 | [Thr+MeTyr+Ahp+Phe+diMeTyr+Val]Asp+BA | | |
| CP-Phe$^2$ | CP 925 | 925.4222 | 908.4194 | 908.4189 | 0.0005 | 10.58 | [Thr+Phe+Ahp+Phe+MePhe+Val]Asp+Ac | 0.5 | |
| CP-Phe$^2$ | CP 953 | 953.4535 | 936.4507 | 936.4500 | 0.0007 | 11.61 | [Thr+Phe+Ahp+Phe+MePhe+Val]Asp+BA | 0.8 | |
| CP-Phe$^2$ | CP 955 | 955.4327 | 938.4300 | 938.4276 | 0.0024 | 10.35 | [Thr+Phe+Ahp+Phe+diMeTyr+Val]Asp+Ac | 0.7 | |
| CP-Phe$^2$ | CP 967 | 967.4691 | 950.4664 | 950.4644 | 0.0020 | 12.16 | [Thr+Phe+Ahp+Phe+MePhe+Leu]Asp+BA | | |
| CP-Phe$^2$ | CP 969b | 969.4484 | 952.4457 | 952.4449 | 0.0008 | 10.60 | [Thr+Phe+Ahp+Phe+MeTyr+Val]Asp+BA | 0.8 | |
| CP-Phe$^2$ | CP 981 | 981.4848 | 964.4820 | 964.4816 | 0.0004 | 12.89 | [Thr+Phe+Ahp+Phe+MePhe+Val]Asp+HA | 1.5 | |
| CP-Phe$^2$ | CP 983 | 983.4640 | 966.4613 | 966.4612 | 0.0001 | 11.35 | [Thr+Phe+Ahp+Phe+diMeTyr+Val]Asp+BA | 4.6 | |
| CP-Phe$^2$ | CP 997 | 997.4797 | 980.4770 | 980.4766 | 0.0004 | 11.08 | [Thr+Phe+Ahp+Phe+MeTyr+Val]Asp+HA | 1.1 | |
| CP-Phe$^2$ | CP 1011 | 1011.4953 | 994.4926 | 994.4933 | 0.0007 | 12.64 | [Thr+Phe+Ahp+Phe+diMeTyr+Val]Asp+HA | 0.3 | |
| CP-Phe$^2$ | CP 1025b | 1025.5110 | 1008.5083 | 1998.51 | | | [Thr+Phe+Ahp+Phe+MeTyr+Val]Asp+OA | | |
| CP-Trp$^2$ | CP 992d | 992.4644 | 975.4616 | 975.4594 | 0.0022 | 11.88 | [Thr+Trp+Ahp+Phe+MePhe+Val]Asp+BA | | |
| CP-Trp$^2$ | CP 1036c | 1036.4906 | 1019.4878 | 1019.49 | | | [Thr+Trp+Ahp+Phe+MeTyr+Val]Asp+HA | | |
| CP-Trp$^2$ | CP 1050 | 1050.5062 | 1033.5035 | 1033.50 | | | [Thr+Trp+Ahp+Phe+diMeTyr+Val]Asp+HA | | |
| CP-Tyr$^2$ | CP 929 | 929.4171 | 912.4144 | 912.4266 | 0.0122 | 4.84 | [Thr+Tyr+Ahp+Phe+diMeTyr+Val]Asp | | |
| CP-Tyr$^2$ | CP 941 | 941.4171 | 924.4144 | 924.4140 | 0.0004 | 9.68 | [Thr+Tyr+Ahp+Phe+MePhe+Val]Asp+Ac | 3.4 | |
| CP-Tyr$^2$ | CP 957 | 957.4120 | 940.4093 | 940.4080 | 0.0013 | 7.63 | [Thr+Tyr+Ahp+Phe+MeTyr+Val]Asp+Ac | 0.6 | |
| CP-Tyr$^2$ | CP 965 | 965.4746 | 948.4719 | 948.4693 | 0.0026 | 2.56 | [Thr+Tyr+Ahp+Leu+diMeTyr+Val]Asp+BA | | |
| CP-Tyr$^2$ | CP 969 | 969.4484 | 952.4457 | 952.4449 | 0.0008 | 10.60 | [Thr+Tyr+Ahp+Phe+MePhe+Val]Asp+BA | 1.6 | [8] |
| CP-Tyr$^2$ | CP 971 | 971.4277 | 954.4249 | 954.4252 | 0.0003 | 9.44 | [Thr+Tyr+Ahp+Phe+diMeTyr+Val]Asp+Ac | 0.7 | |
| CP-Tyr$^2$ | CP 983b | 983.4640 | 966.4613 | 966.46 | | | [Thr+Tyr+Ahp+Phe+MePhe+Leu]Asp+BA | | |
| CP-Tyr$^2$ | CP 983c | 983.4640 | 966.4613 | 966.46 | | | [Thr+Tyr+Ahp+Phe+MePhe+Val]Glu+BA | | |
| CP-Tyr$^2$ | CP 985 | 985.4433 | 968.4406 | 968.4396 | 0.0010 | 8.65 | [Thr+Tyr+Ahp+Phe+MeTyr+Val]Asp+BA | 3.1 | [8] |
| CP-Tyr$^2$ | CP 993 | 993.5059 | 976.5032 | 976.50 | | | [Thr+Tyr+Ahp+Leu+diMeTyr+Val]Asp+HA | | |
| CP-Tyr$^2$ | CP 997b | 997.4797 | 980.4770 | 980.4764 | 0.0005 | 11.84 | [Thr+Tyr+Ahp+Phe+MePhe+Val]Asp+HA | 2.6 | |
| CP-Tyr$^2$ | CP 999 | 999.4590 | 982.4562 | 982.4555 | 0.0007 | 10.36 | [Thr+Tyr+Ahp+Phe+diMeTyr+Val]Asp+BA | 8.1 | [8] |
| CP-Tyr$^2$ | CP 999b | 999.4590 | 982.4562 | 982.4537 | 0.0025 | 9.22 | [Thr+Tyr+Ahp+Phe+MeTyr+Leu]Asp+BA | | |
| CP-Tyr$^2$ | CP 999c | 999.4590 | 982.4562 | 982.46 | | | [Thr+Tyr+Ahp+Phe+MeTyr+Val]Glu+HA | | |
| CP-Tyr$^2$ | CP 1011b | 1011.4953 | 994.4926 | 994.4917 | 0.0009 | 7.30 | [Thr+Tyr+Ahp+Phe+MePhe+Leu]Asp+HA | | |
| CP-Tyr$^2$ | CP 1013b | 1013.3746 | 996.3719 | 996.37 | | | [Thr+Tyr+Ahp+Phe+diMeTyr+Leu]Asp+BA | | |
| CP-Tyr$^2$ | CP 1013 | 1013.4746 | 996.4719 | 996.4699 | 0.0020 | 10.04 | [Thr+Tyr+Ahp+Phe+MeTyr+Val]Asp+HA | 2.8 | [8] |
| CP-Tyr$^2$ | CP 1013c | 1013.4746 | 996.4719 | 996.4702 | 0.0017 | 11.35 | [Thr+Tyr+Ahp+Phe+diMeTyr+Val]Glu+BA | | |
| CP-Tyr$^2$ | CP 1016 | 1016.0100 | 999.0072 | 999.0346 | 0.0274 | 12.72 | [Thr+Tyr+Ahp+Phe+diMe,OTyr+Val]Asp+BA | | |

| | | | | | | | | | |
|---|---|---|---|---|---|---|---|---|---|
| CP-Tyr$^{2}$ | CP 1025 | 1025.5110 | 1008.5083 | 1008.5080 | 0.0002 | 13.24 | [Thr+Tyr+Ahp+Phe+MePhe+Val]Asp+OA | 0.2 | |
| CP-Tyr$^{2}$ | CP 1027 | 1027.4903 | 1010.4875 | 1010.4869 | 0.0006 | 11.61 | [Thr+Tyr+Ahp+Phe+diMeTyr+Val]Asp+HA | 1.3 | [8] |
| CP-Tyr$^{2}$ | CP 1036b | 1036.4906 | 1019.4878 | 1019.4445 | 0.0433 | 8.64 | [Thr+Tyr+Ahp+Phe+MeTrp+Val]Asp+HA | | |
| CP-Tyr$^{2}$ | CP 1041 | 1041.5059 | 1024.5032 | 1024.5038 | 0.0006 | 11.53 | [Thr+Tyr+Ahp+Phe+MeTyr+Val]Asp+OA | | |
| CP-Tyr$^{2}$ | CP 1053 | 1053.5423 | 1036.5396 | 1036.54 | | | [Thr+Tyr+Ahp+Phe+MePhe+Val]Asp+DA | | |
| CP-Tyr$^{2}$ | CP 1055 | 1055.5216 | 1038.5188 | 1038.5183 | 0.0005 | 12.99 | [Thr+Tyr+Ahp+Phe+diMeTyr+Val]Asp+OA | 1.4 | |
| CP-Tyr$^{2}$ | CP 1069 | 1069.5372 | 1052.5345 | 1052.5350 | 0.0005 | 13.09 | [Thr+Tyr+Ahp+Phe+MeTyr+Val]Asp+DA | | |
| CP-Tyr$^{2}$ | CP 1083 | 1083.5529 | 1066.5501 | 1066.5454 | 0.0047 | 8.47 | [Thr+Tyr+Ahp+Phe+diMeTyr+Val]Asp+DA | | |
| CP-Tyr$^{2}$ | CP 1097 | 1097.5685 | 1080.5658 | 1082.57 | | | [Thr+Tyr+Ahp+Phe+diMeTyr+Val]Glu+DA | | |

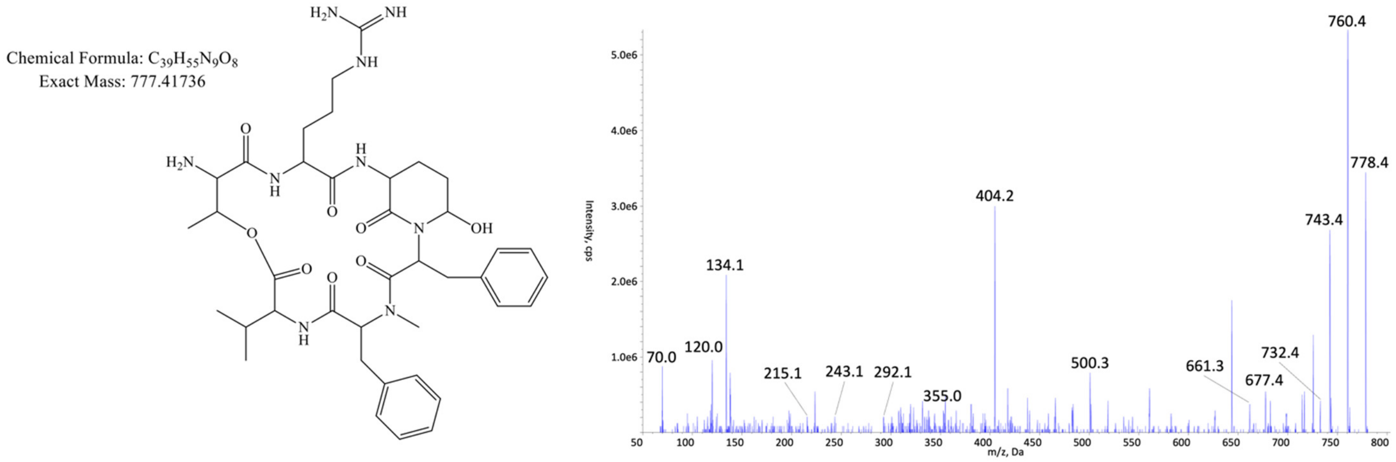


Figure S1. Structure and enhanced product ion mass spectrum of the cyanopeptolin CP 777.

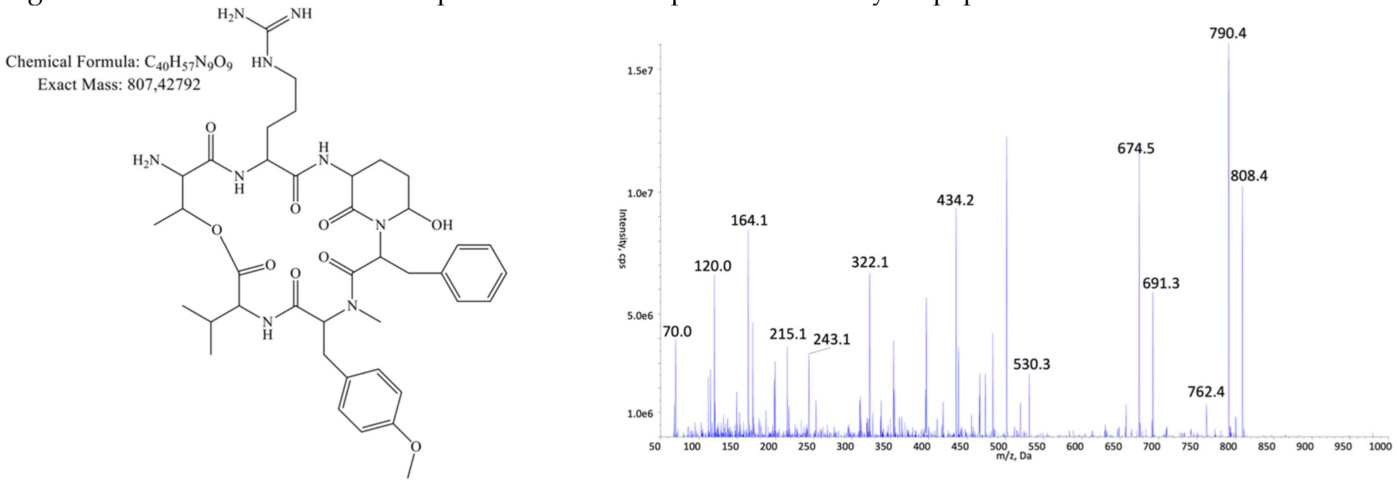


Figure S2. Structure and enhanced product ion mass spectrum of the cyanopeptolin CP 807.

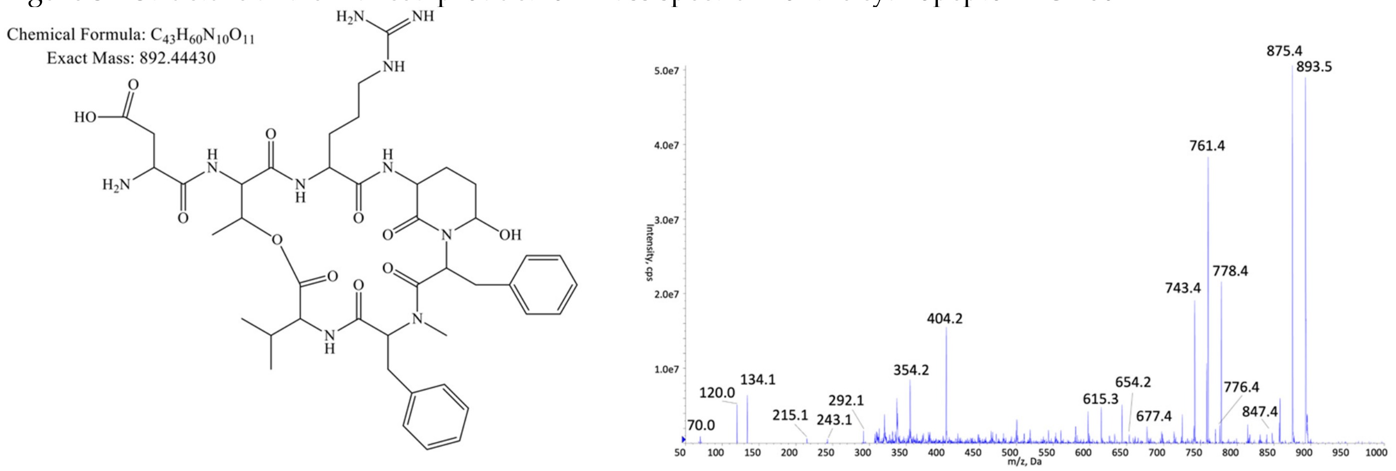


Figure S3. Structure and enhanced product ion mass spectrum of the cyanopeptolin CP 892.

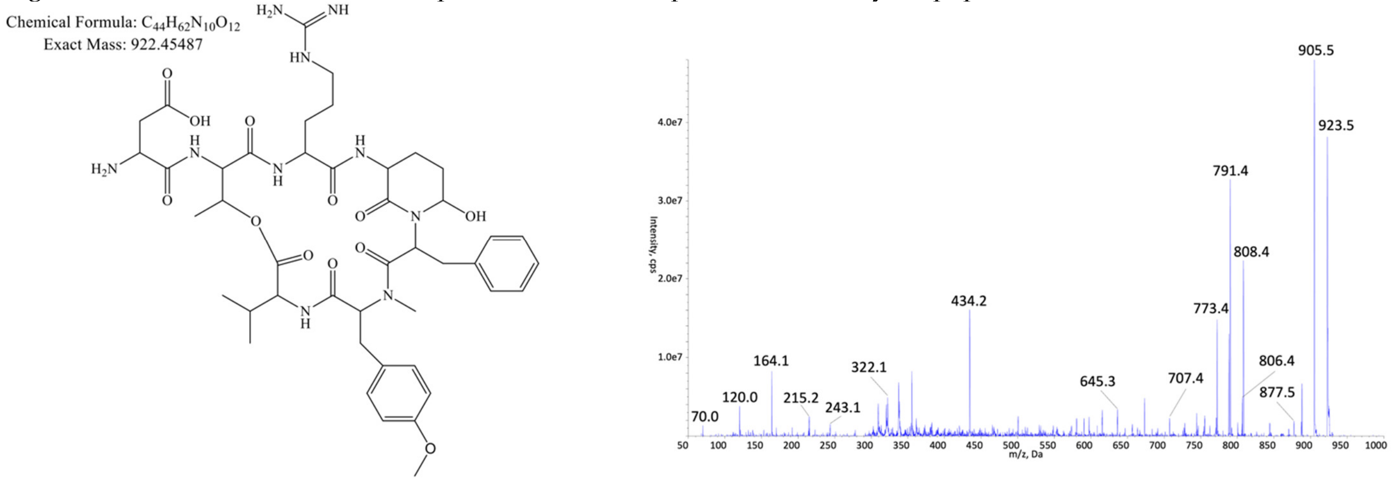


Figure S4. Structure and enhanced product ion mass spectrum of the cyanopeptolin CP 922.

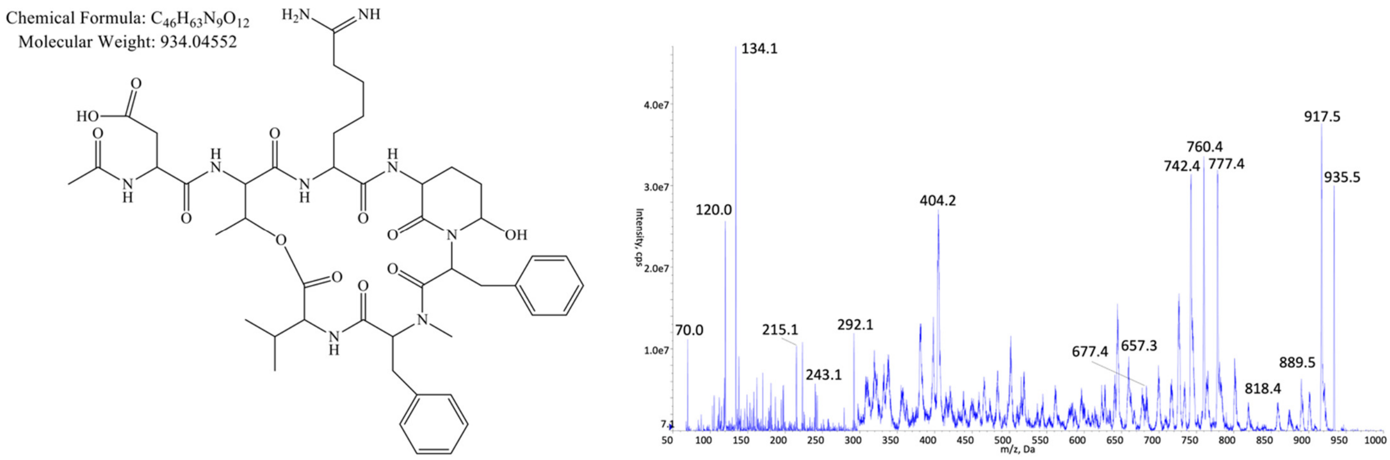


Figure S5. Structure and enhanced product ion mass spectrum of the cyanopeptolin CP 934.

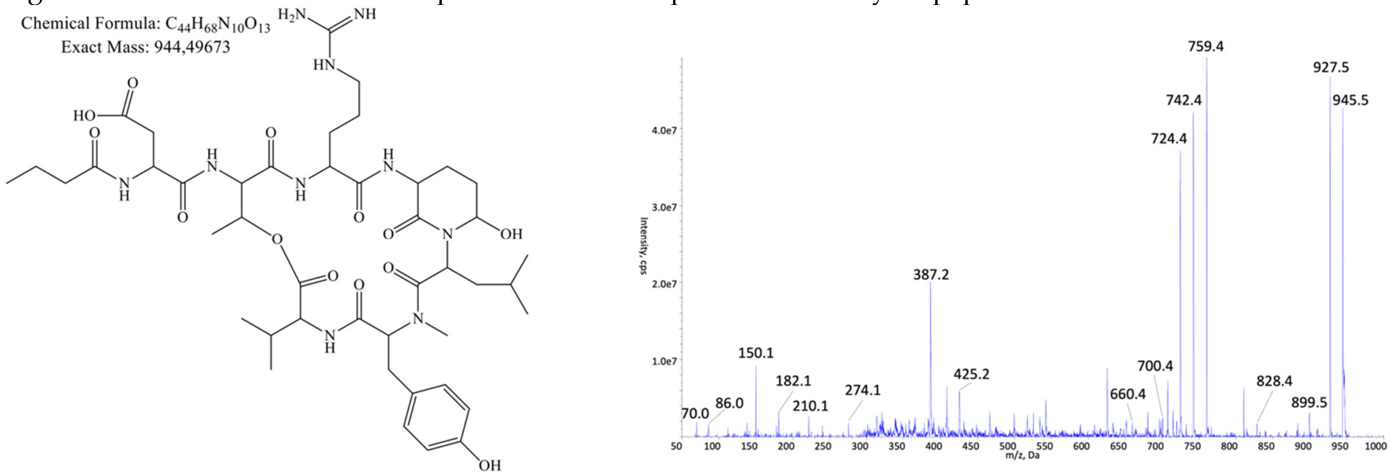


Figure S6. Structure and enhanced product ion mass spectrum of the cyanopeptolin CP 944.

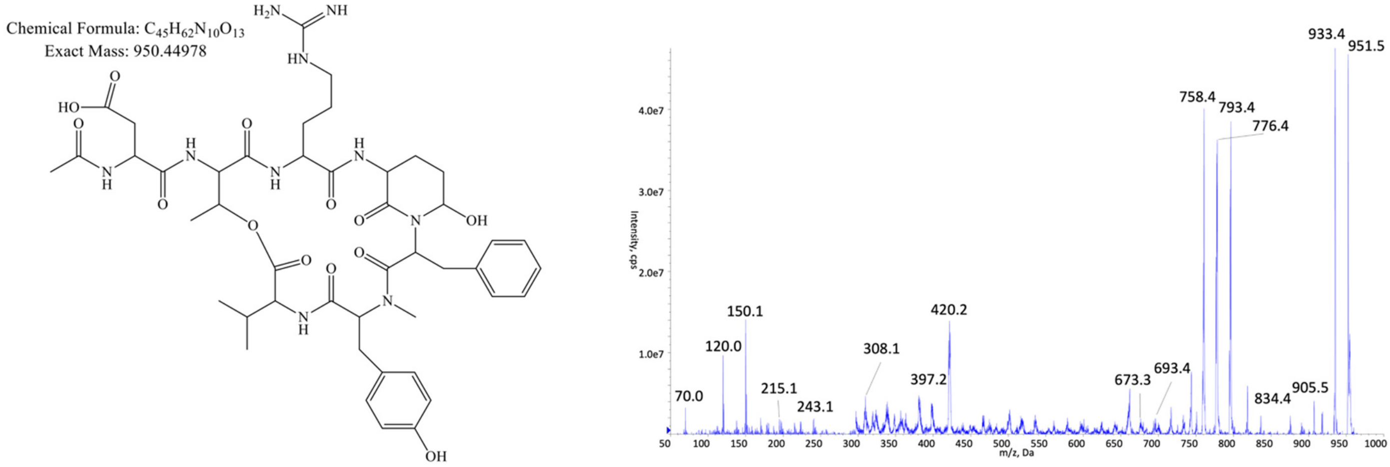


Figure S7. Structure and enhanced product ion mass spectrum of the cyanopeptolin CP 950.

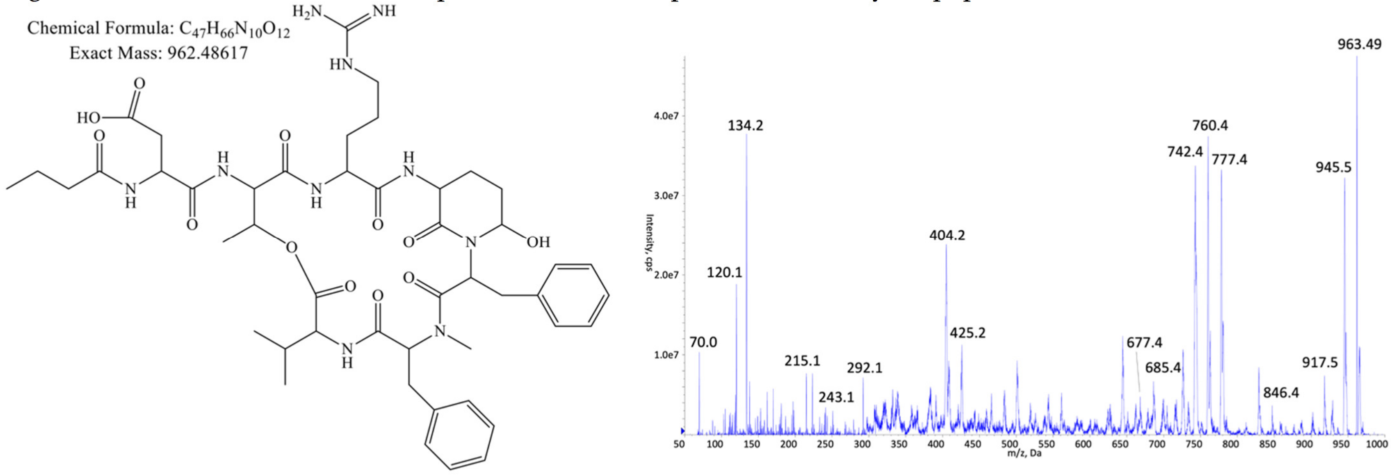


Figure S8. Structure and enhanced product ion mass spectrum of the cyanopeptolin CP 962 [8].

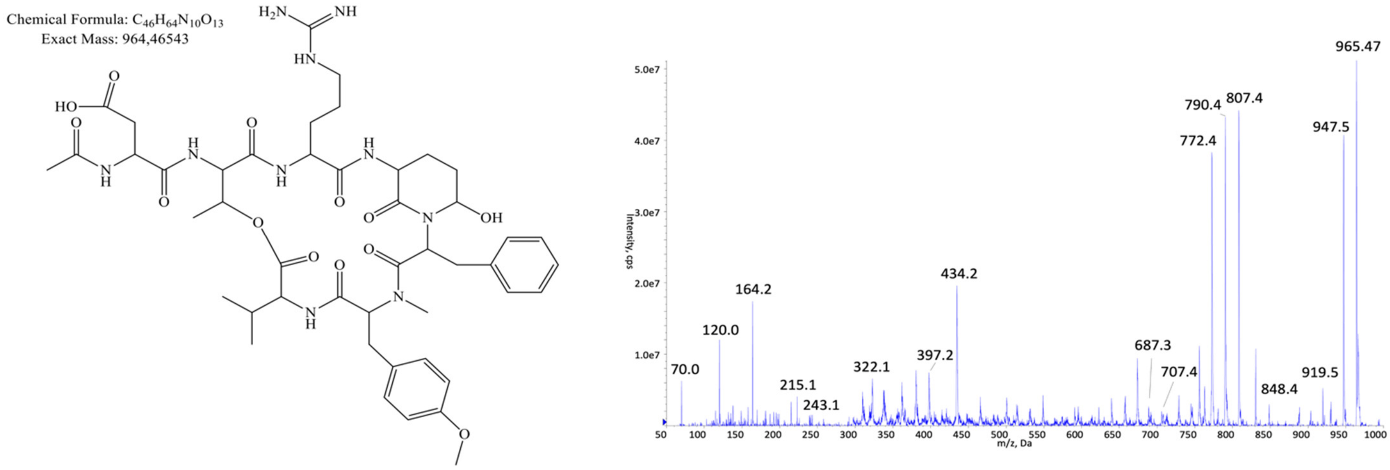


Figure S9. Structure and enhanced product ion mass spectrum of the cyanopeptolin CP 964.

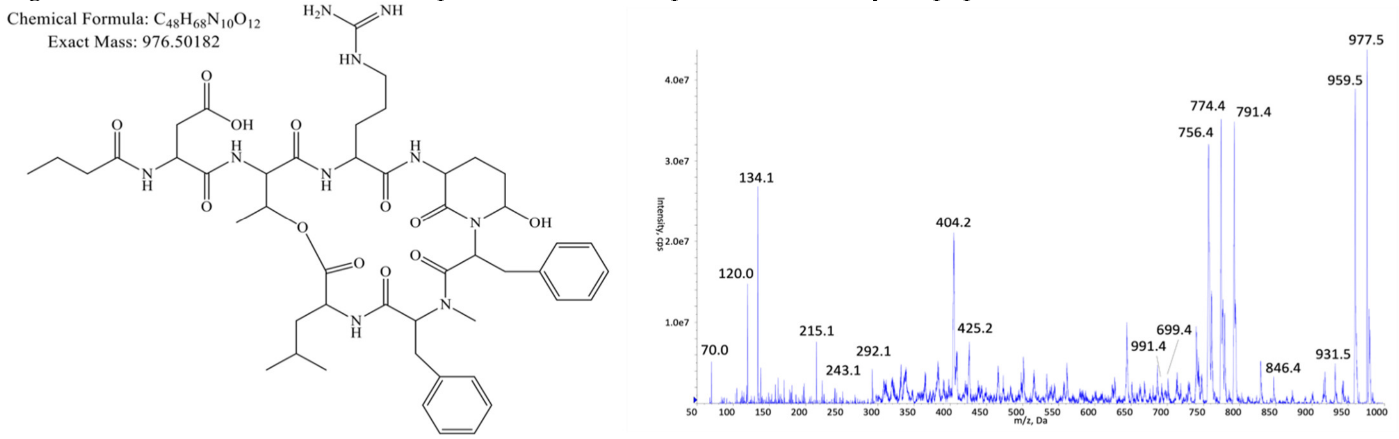


Figure S10. Structure and enhanced product ion mass spectrum of the cyanopeptolin CP 976.

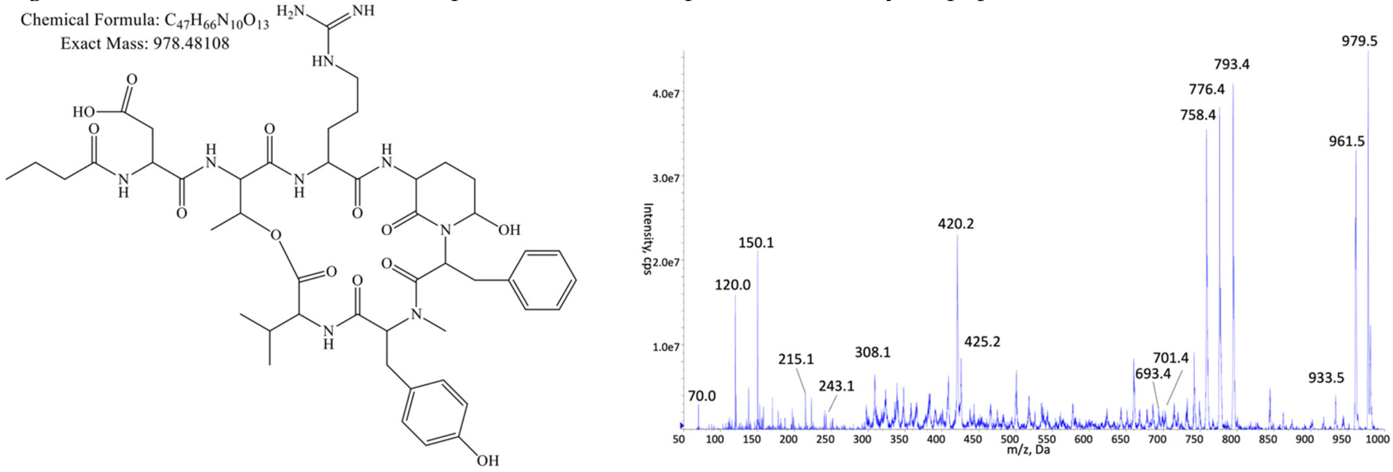


Figure S11. Structure and enhanced product ion mass spectrum of the cyanopeptolin CP 978 [8].

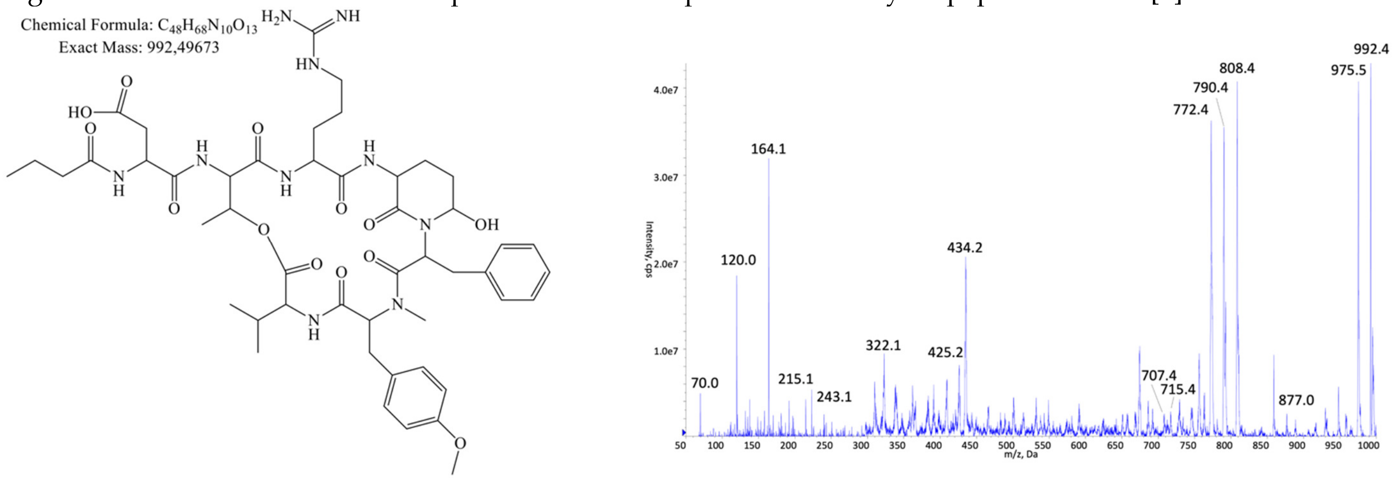


Figure S12. Structure and enhanced product ion mass spectrum of the cyanopeptolin CP 992 [8].

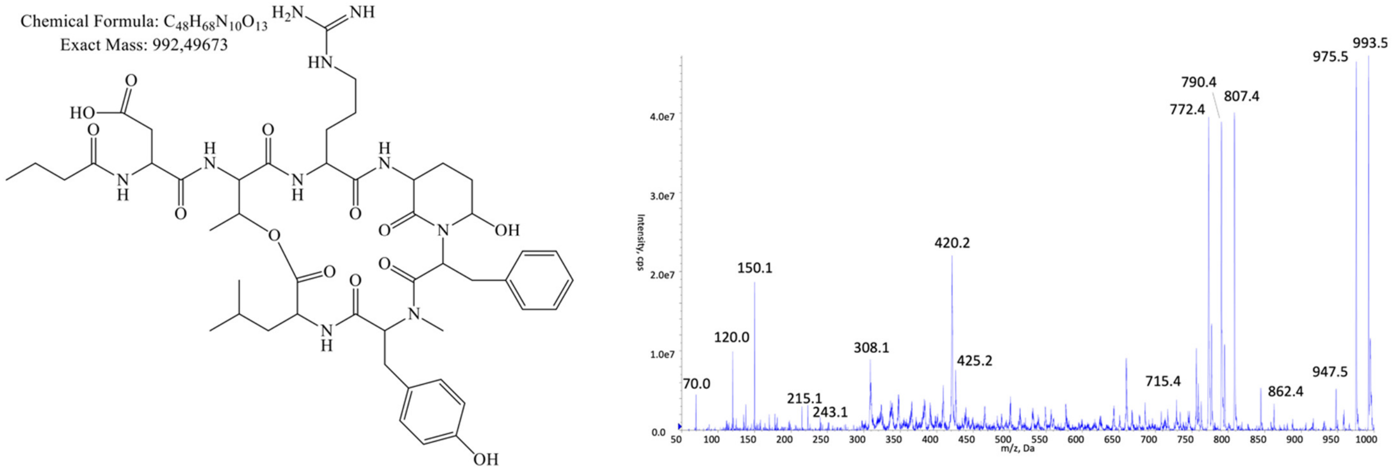


Figure S13. Structure and enhanced product ion mass spectrum of the cyanopeptolin CP 992b.

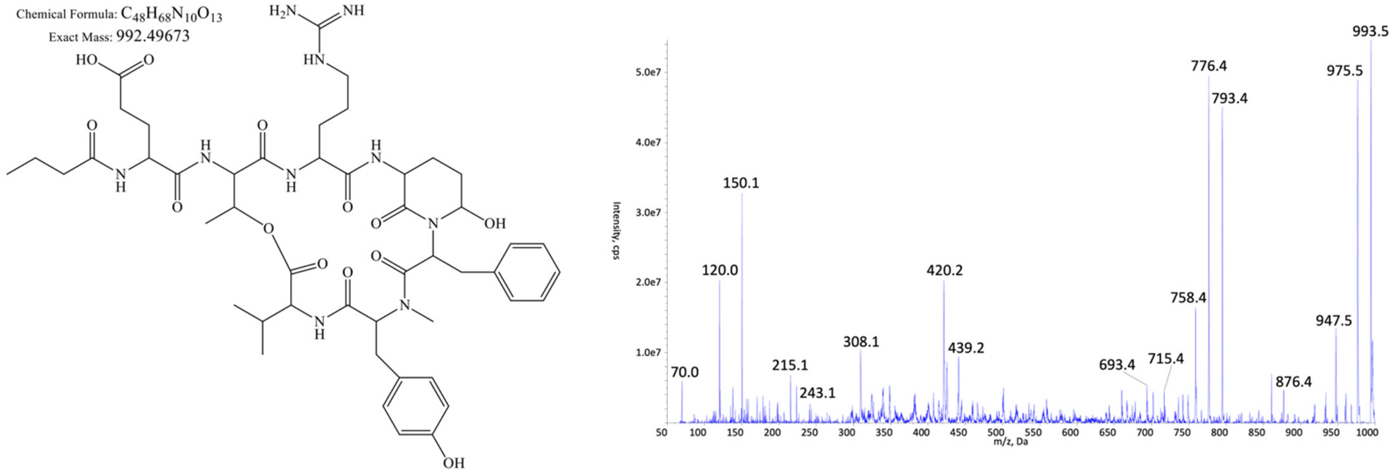


Figure S14. Structure and enhanced product ion mass spectrum of the cyanopeptolin CP 992c.

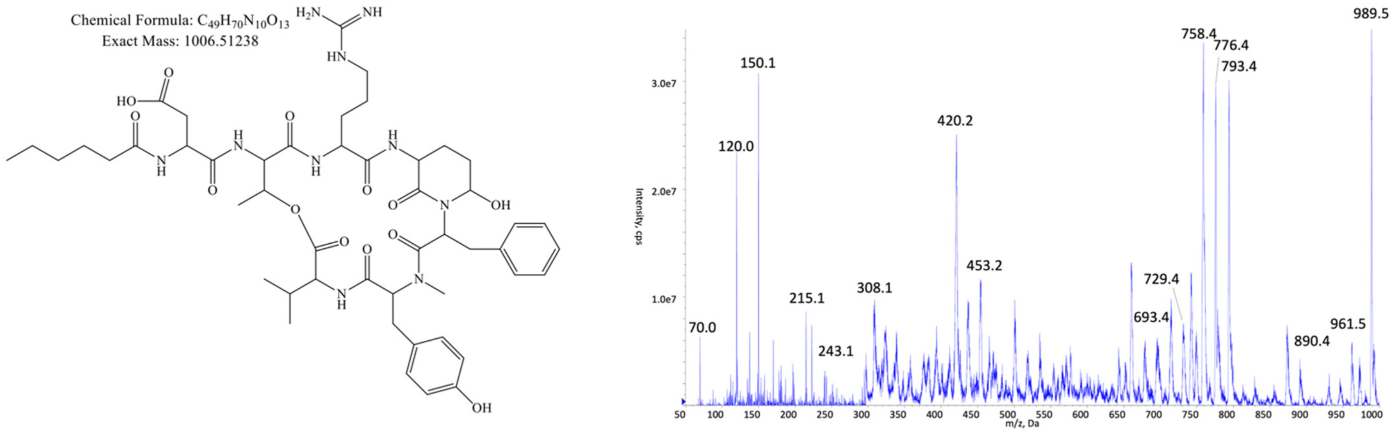


Figure S15. Structure and enhanced product ion mass spectrum of the cyanopeptolin CP 1006[8].

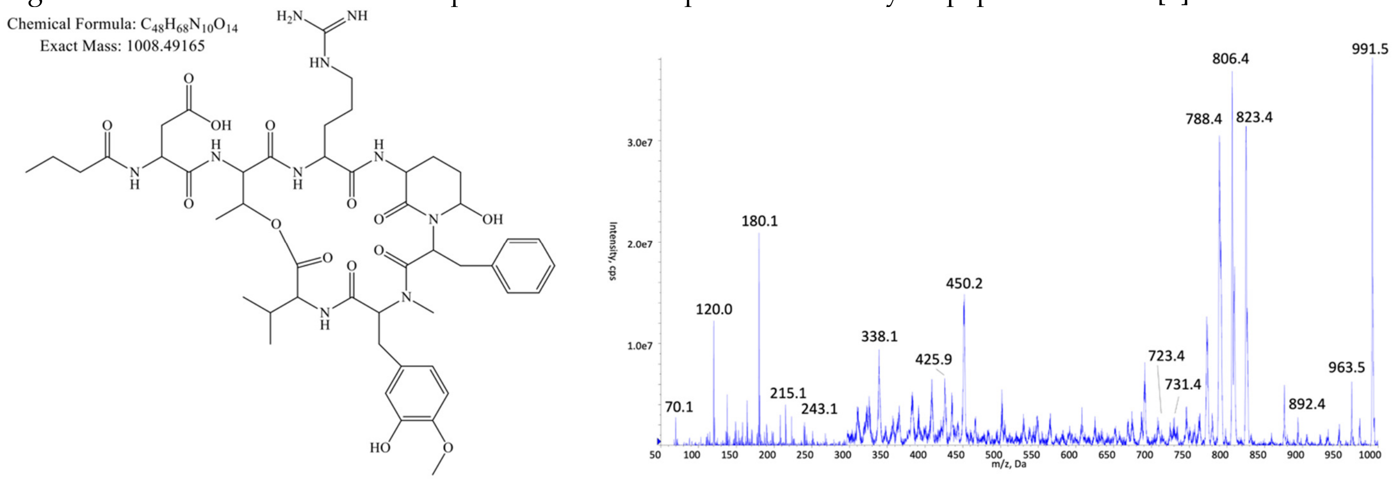


Figure S16. Structure and enhanced product ion mass spectrum of the cyanopeptolin CP 1008.

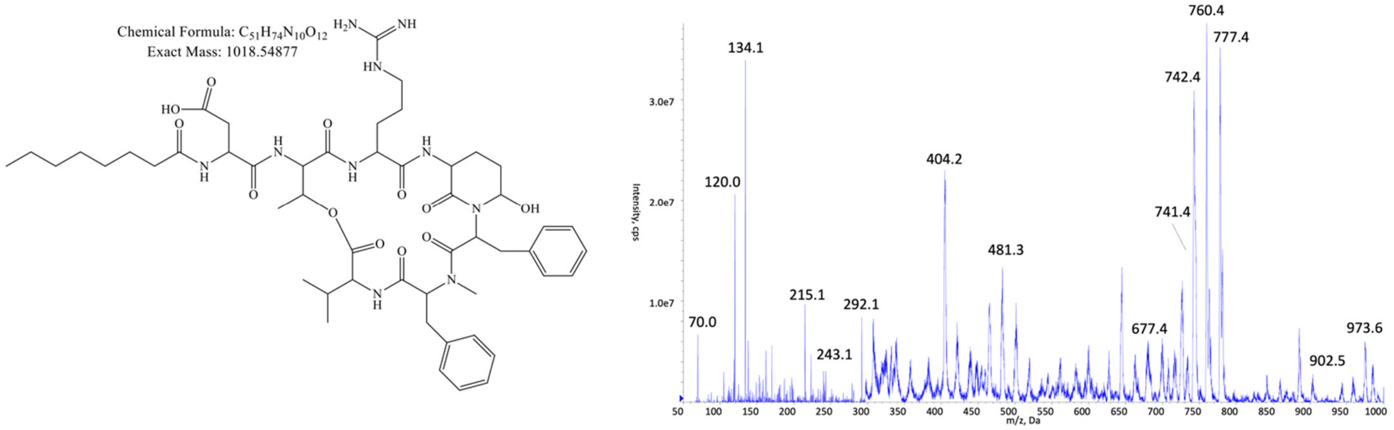


Figure S17. Structure and enhanced product ion mass spectrum of the cyanopeptolin CP 1018 [8].

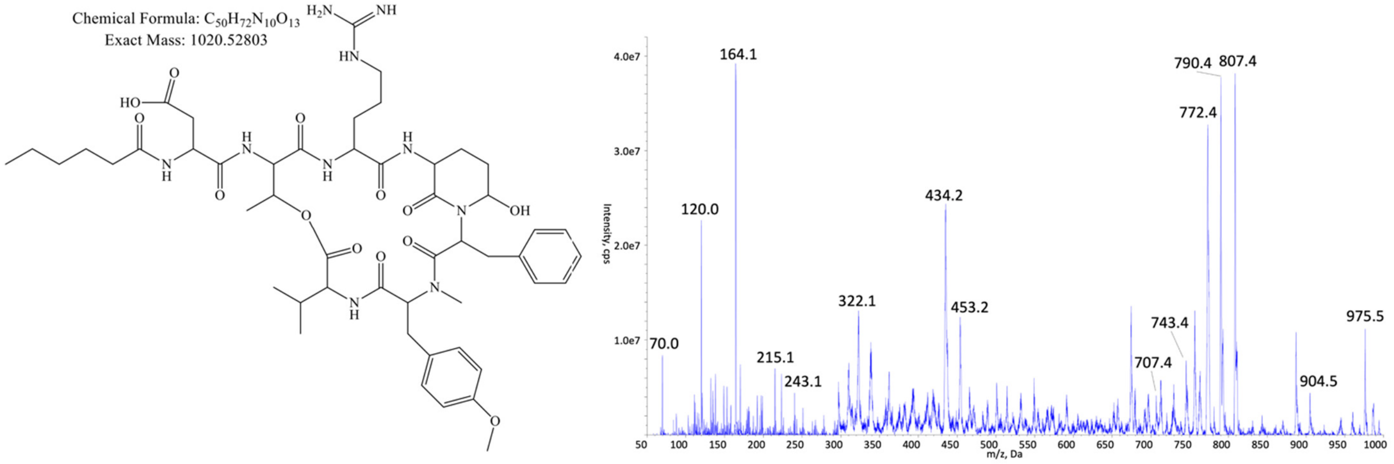


Figure S18. Structure and enhanced product ion mass spectrum of the cyanopeptolin CP 1020b [8].

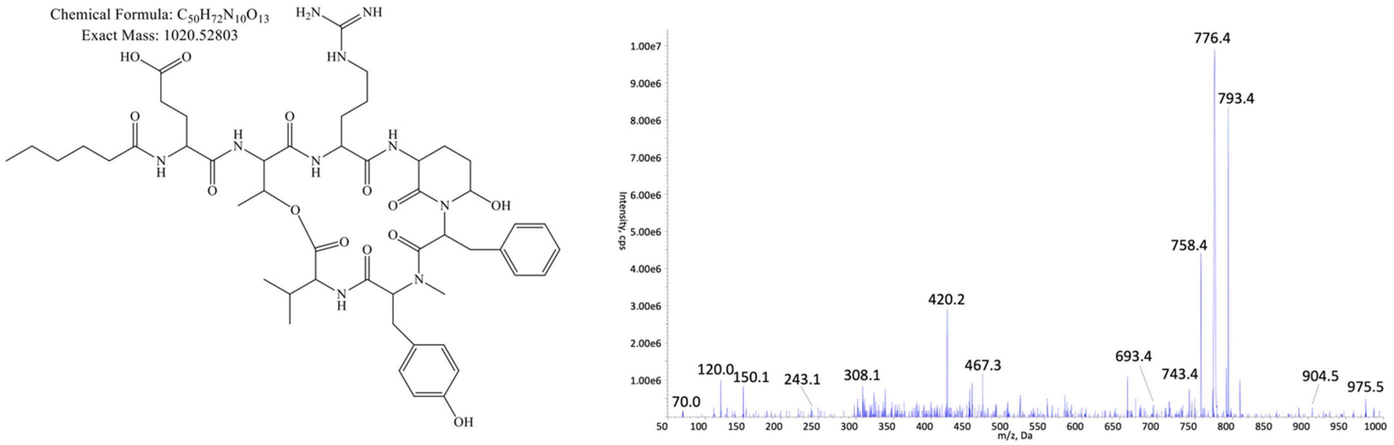


Figure S19. Structure and enhanced product ion mass spectrum of the cyanopeptolin CP 1020.

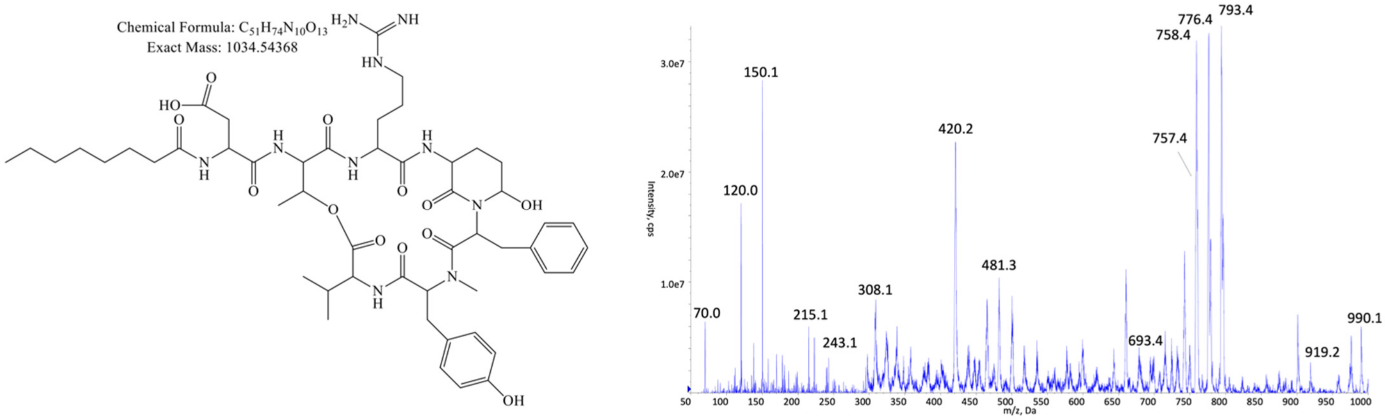


Figure S20. Structure and enhanced product ion mass spectrum of the cyanopeptolin CP 1034.

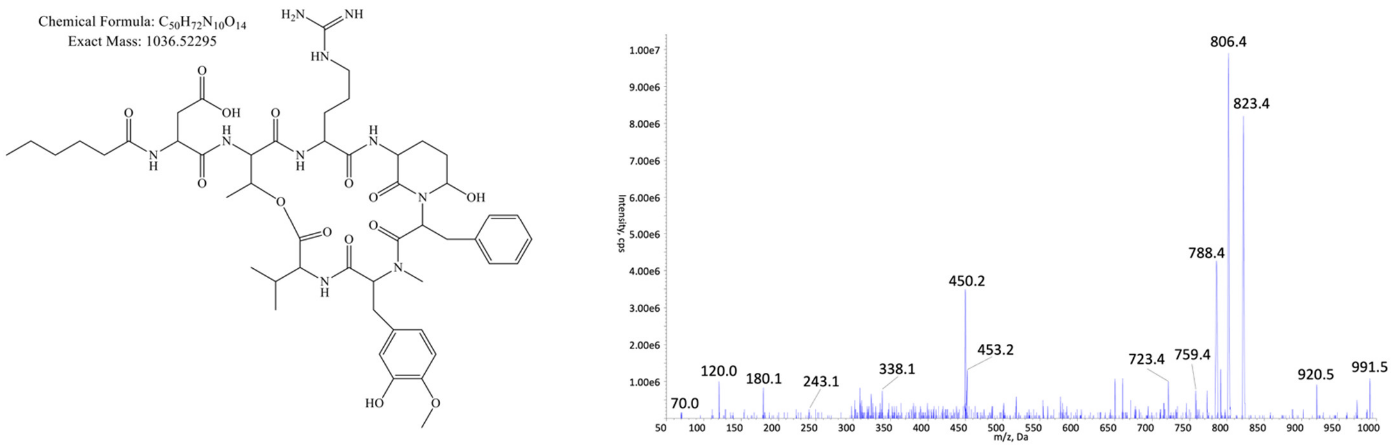


Figure S21. Structure and enhanced product ion mass spectrum of the cyanopeptolin CP 1036.

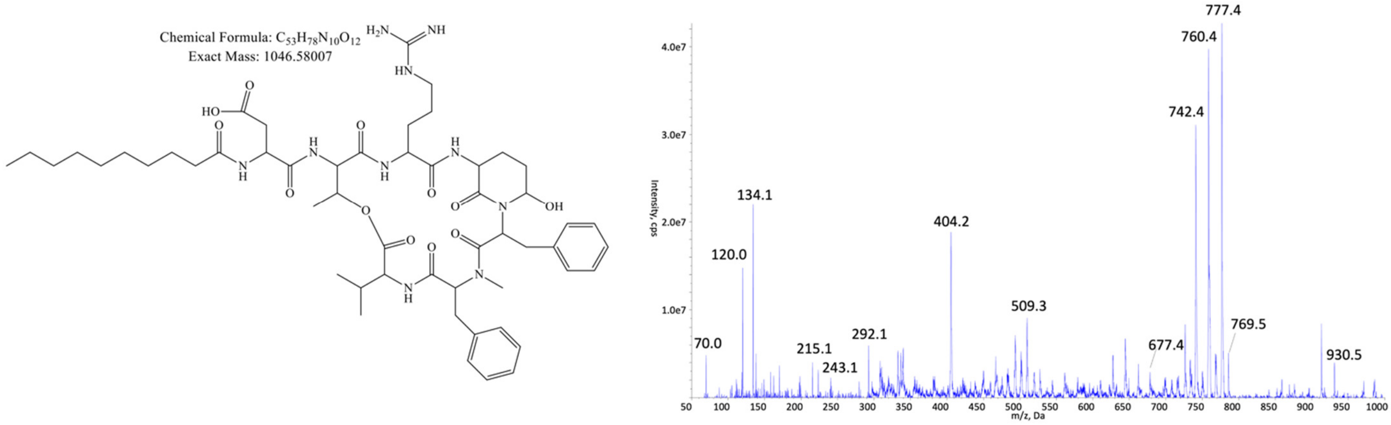


Figure S22. Structure and enhanced product ion mass spectrum of the cyanopeptolin CP 1046.

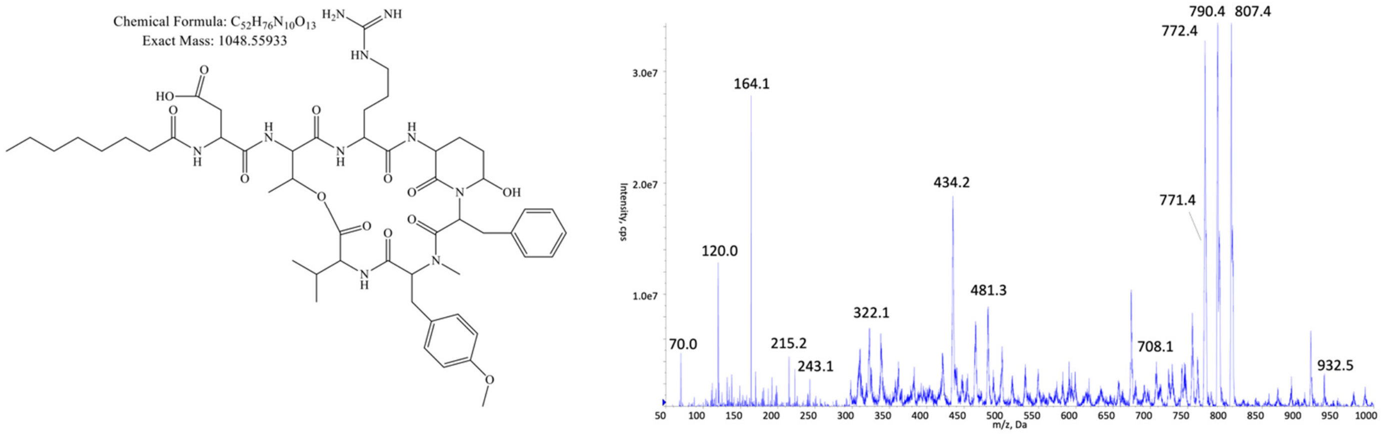


Figure S23. Structure and enhanced product ion mass spectrum of the cyanopeptolin CP 1048 [8].

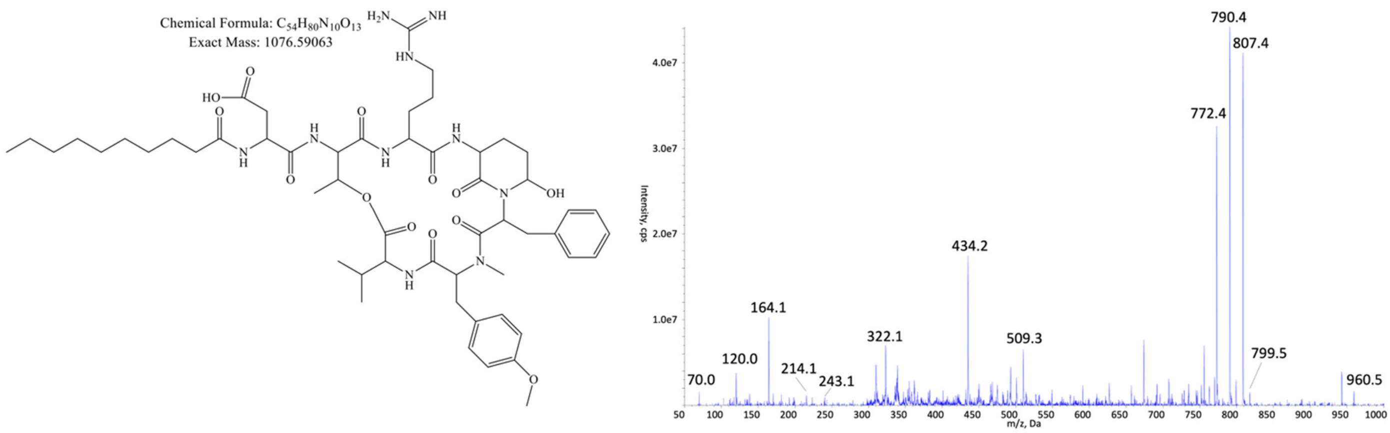


Figure S24. Structure and enhanced product ion mass spectrum of the cyanopeptolin CP 1076.

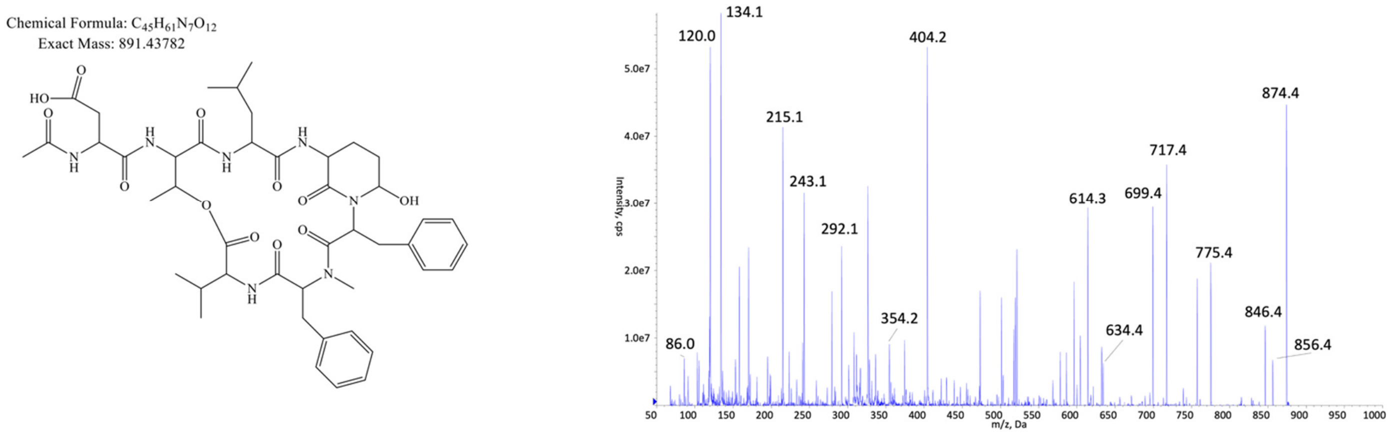


Figure S25. Structure and enhanced product ion mass spectrum of the cyanopeptolin CP 891.

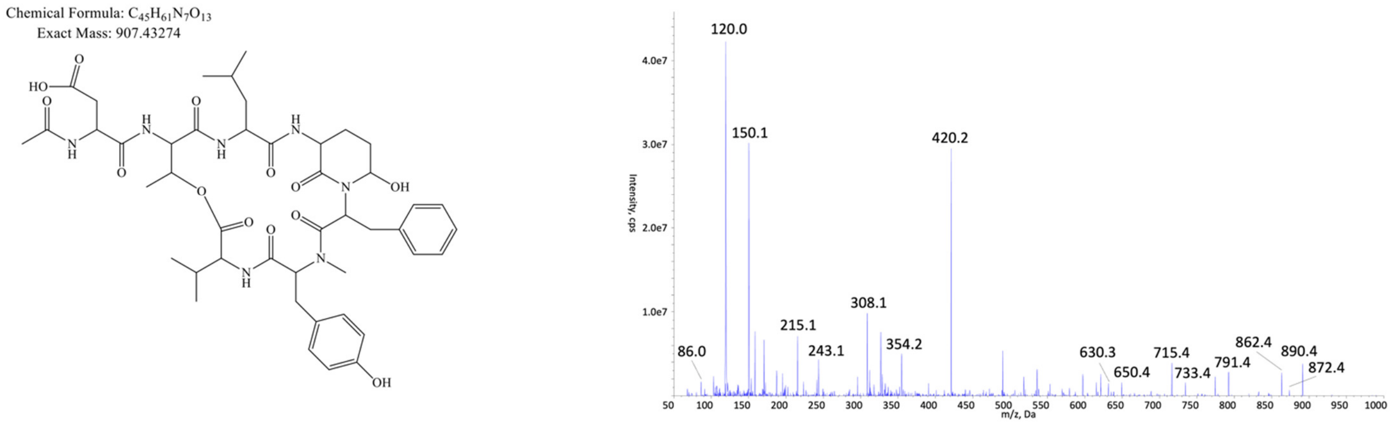


Figure S26. Structure and enhanced product ion mass spectrum of the cyanopeptolin CP 907.

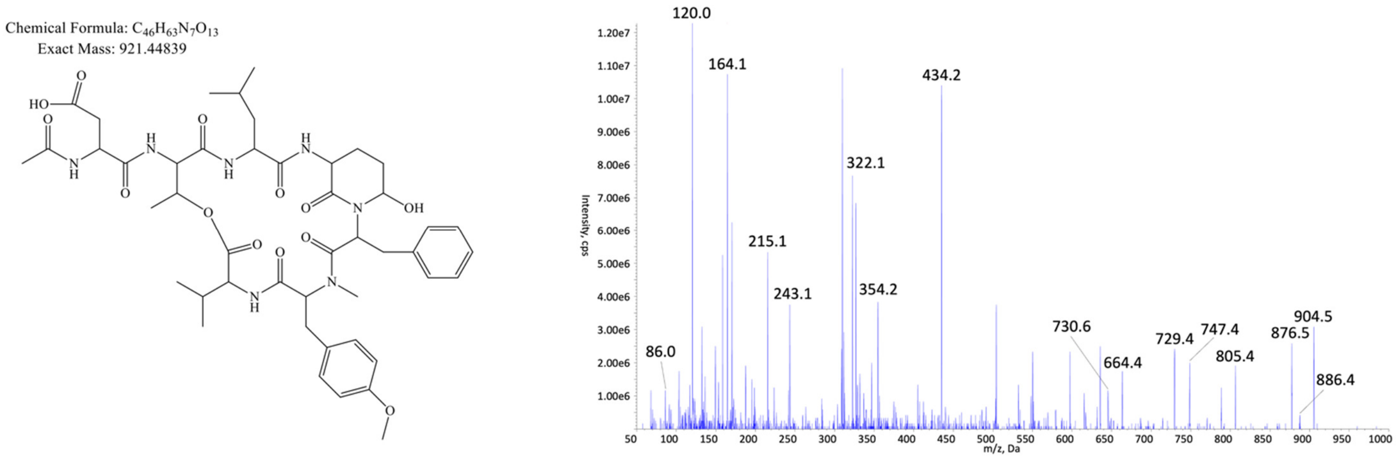


Figure S27. Structure and enhanced product ion mass spectrum of the cyanopeptolin CP 921.

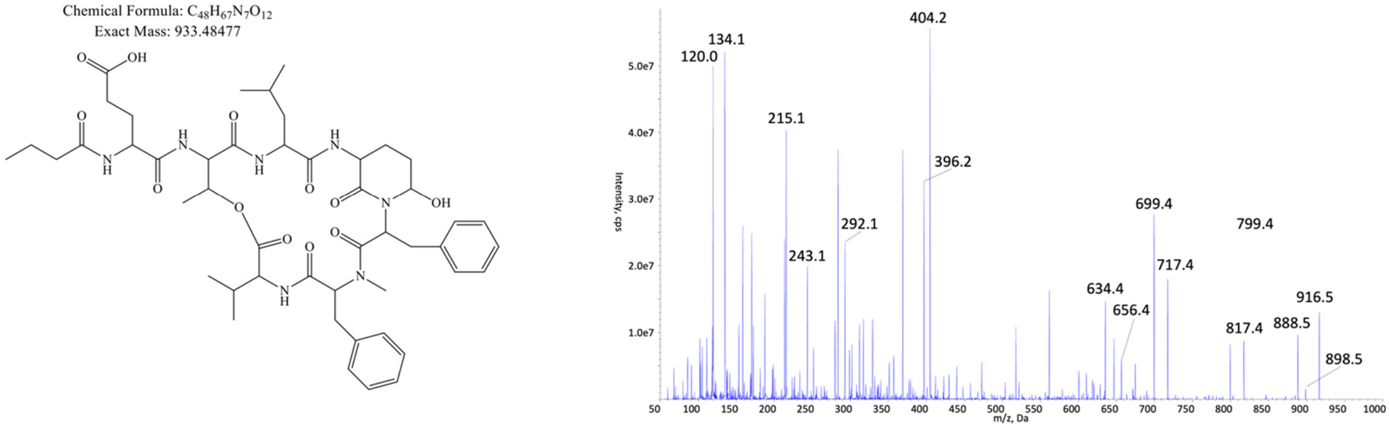


Figure S28. Structure and enhanced product ion mass spectrum of the cyanopeptolin CP 933.

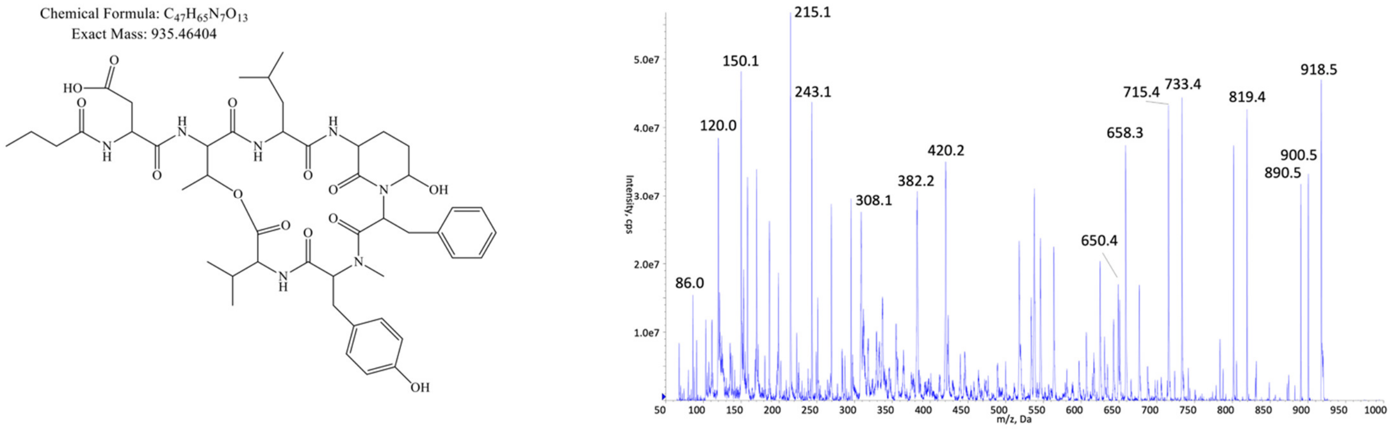


Figure S29. Structure and enhanced product ion mass spectrum of the cyanopeptolin CP 935.

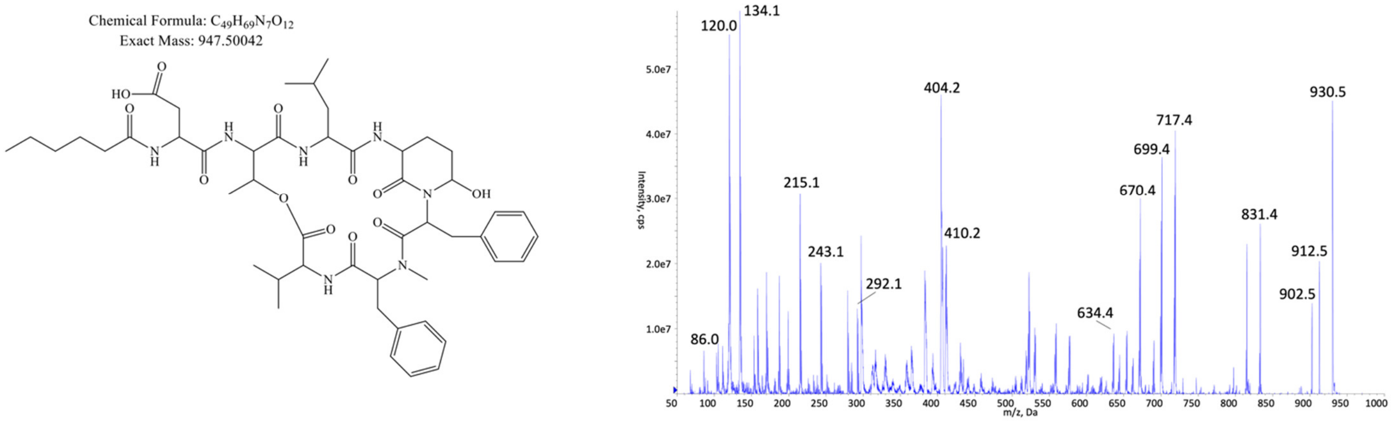


Figure S30. Structure and enhanced product ion mass spectrum of the cyanopeptolin CP 947.

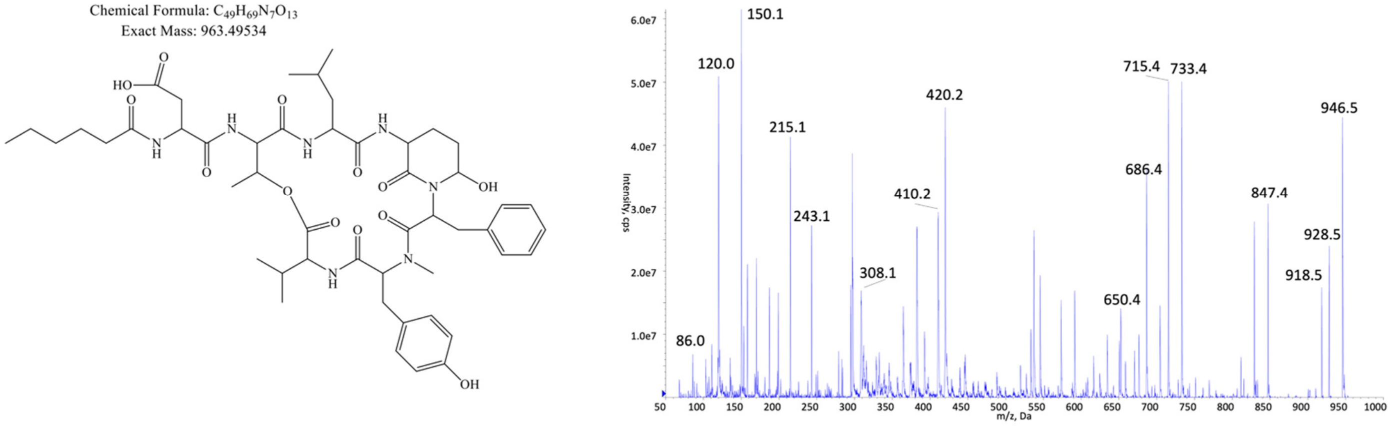


Figure S31. Structure and enhanced product ion mass spectrum of the cyanopeptolin CP 963.

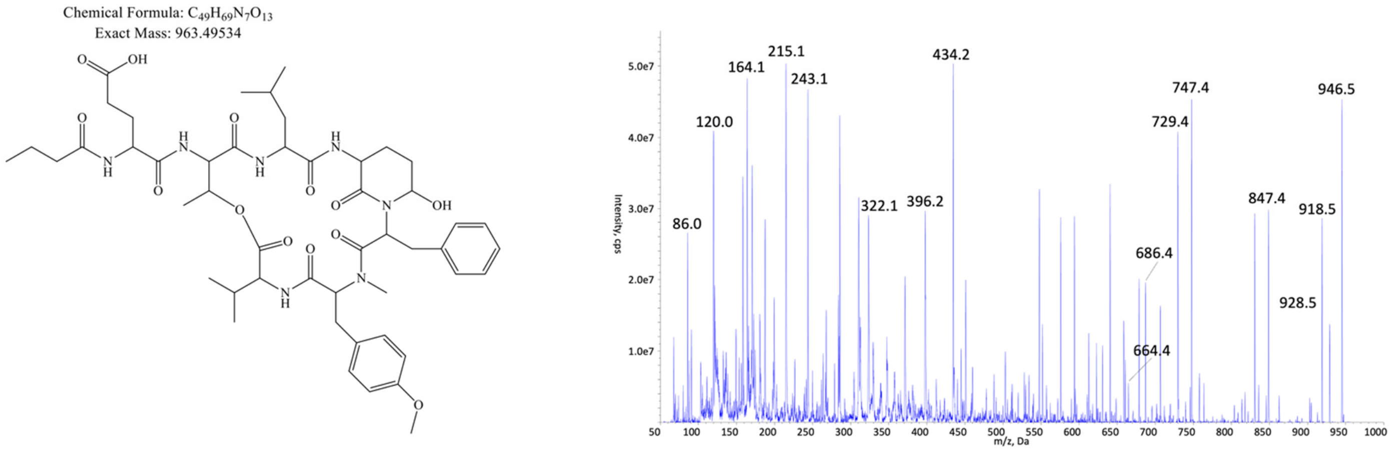


Figure S32. Structure and enhanced product ion mass spectrum of the cyanopeptolin CP 963b.

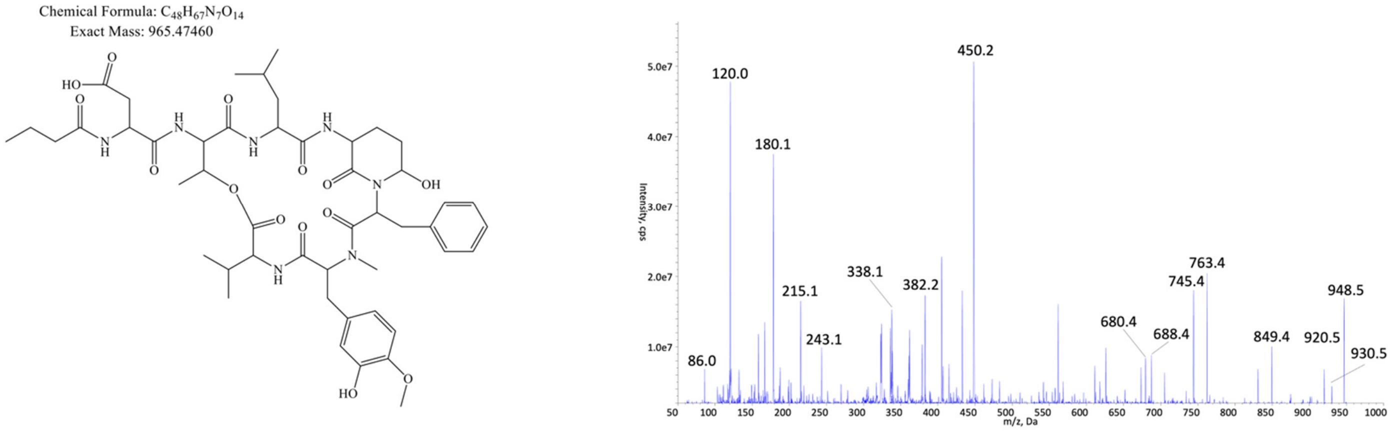


Figure S33. Structure and enhanced product ion mass spectrum of the cyanopeptolin CP 965b.

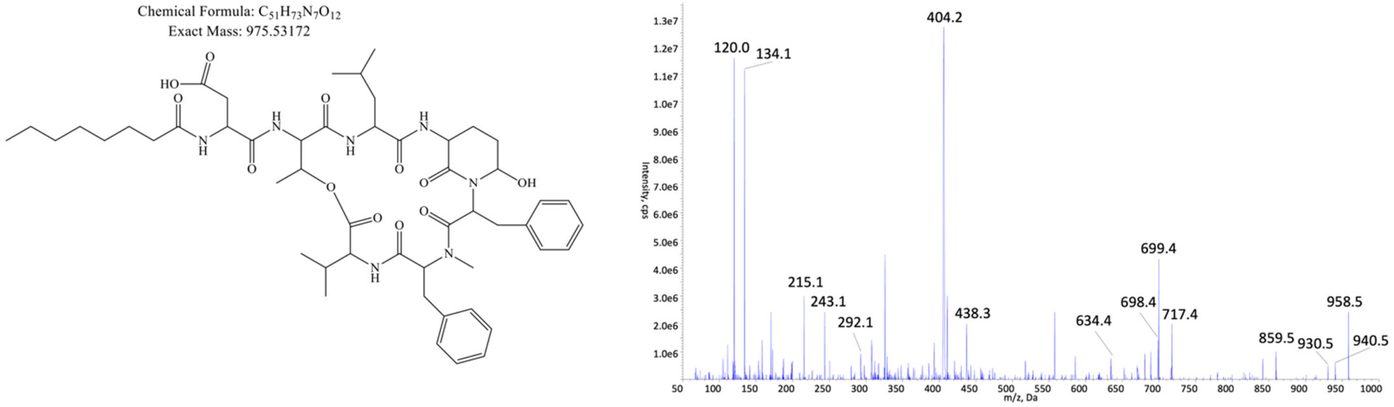


Figure S34. Structure and enhanced product ion mass spectrum of the cyanopeptolin CP 975.

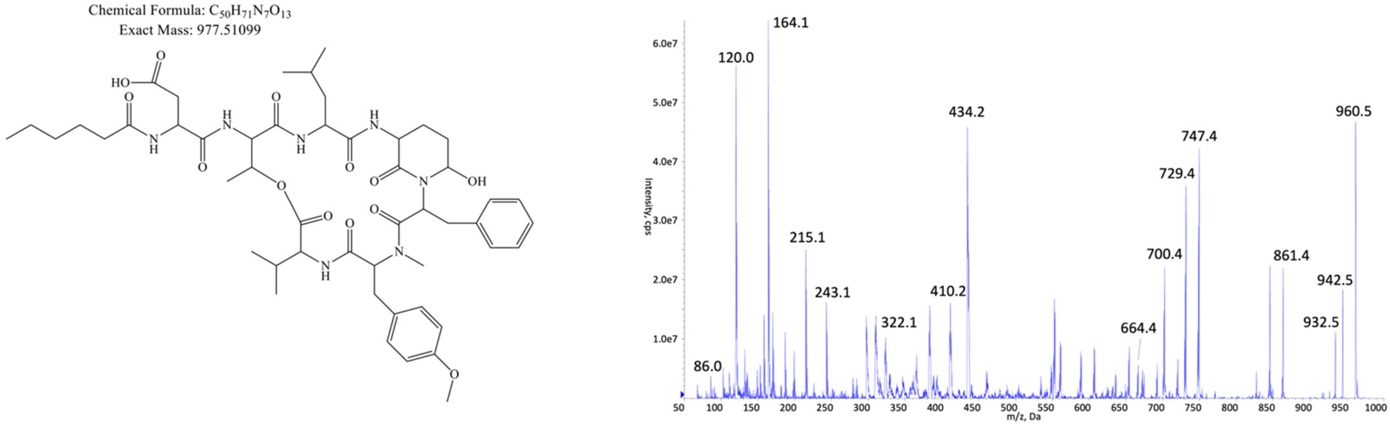


Figure S35. Structure and enhanced product ion mass spectrum of the cyanopeptolin CP 977.

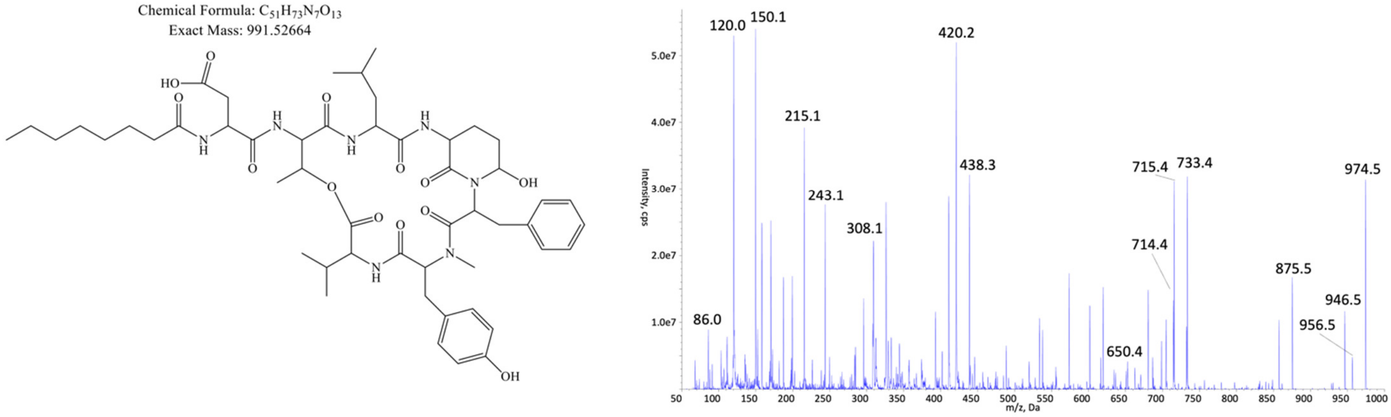


Figure S36. Structure and enhanced product ion mass spectrum of the cyanopeptolin CP 991.

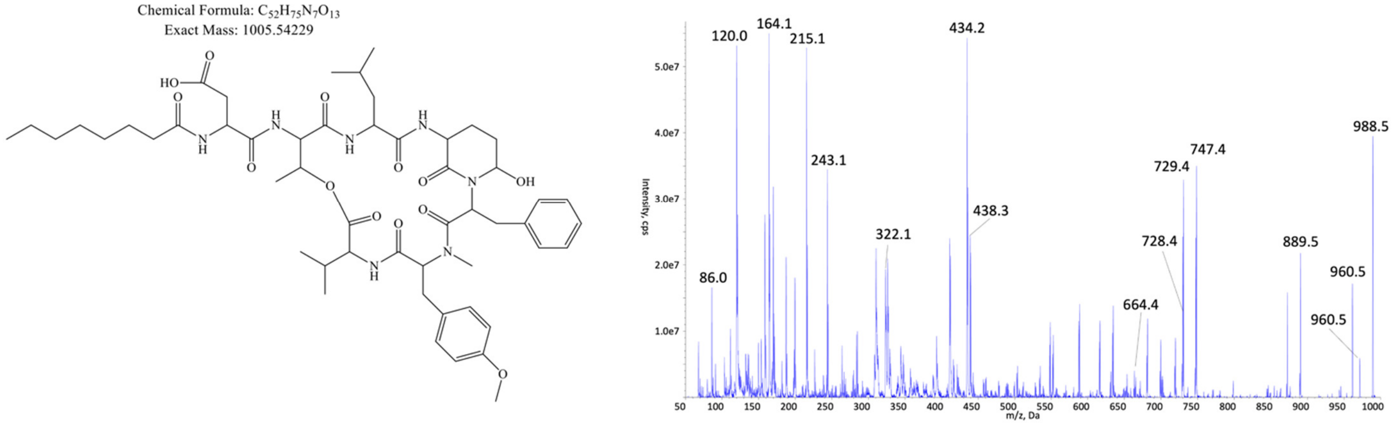


Figure S37. Structure and enhanced product ion mass spectrum of the cyanopeptolin CP 1005.

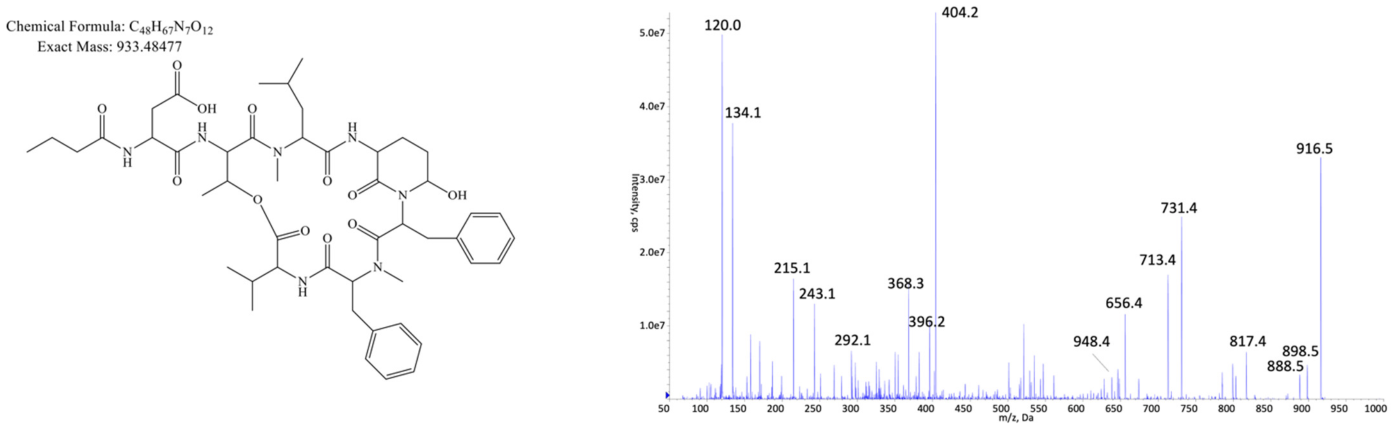


Figure S38. Structure and enhanced product ion mass spectrum of the cyanopeptolin CP 933b.

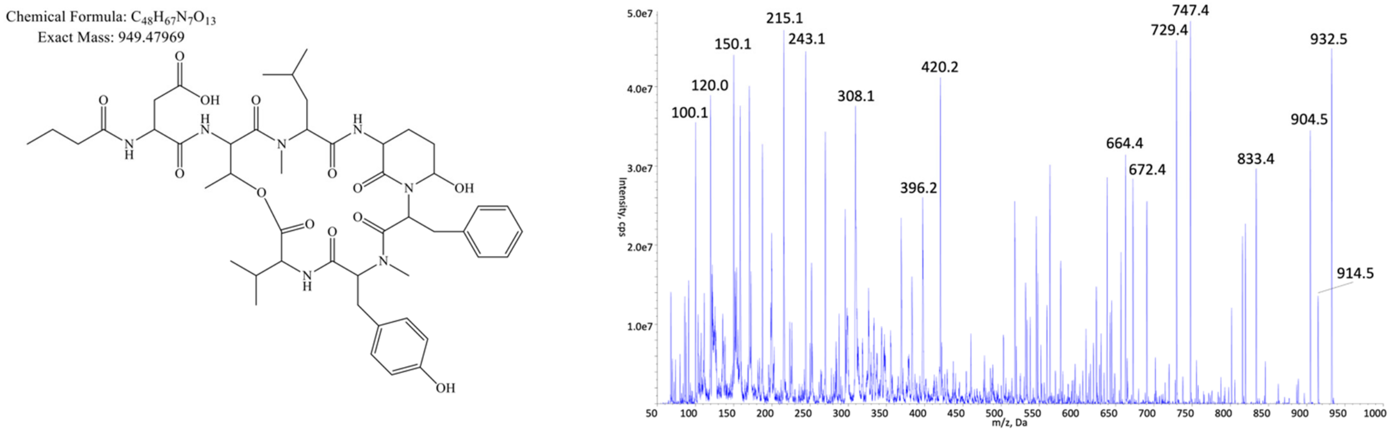


Figure S39. Structure and enhanced product ion mass spectrum of the cyanopeptolin CP 949b.

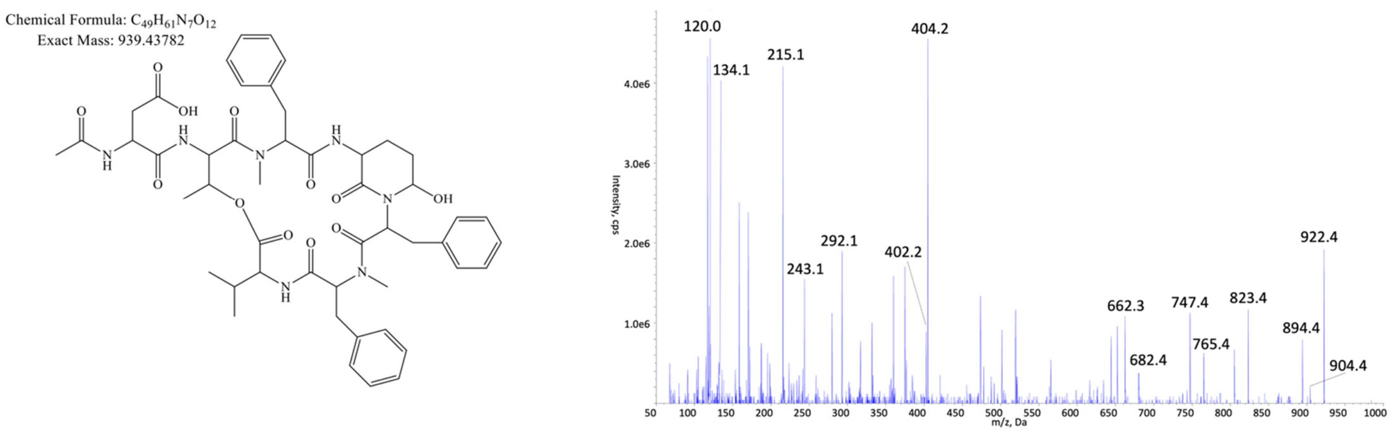


Figure S40. Structure and enhanced product ion mass spectrum of the cyanopeptolin CP 939b.

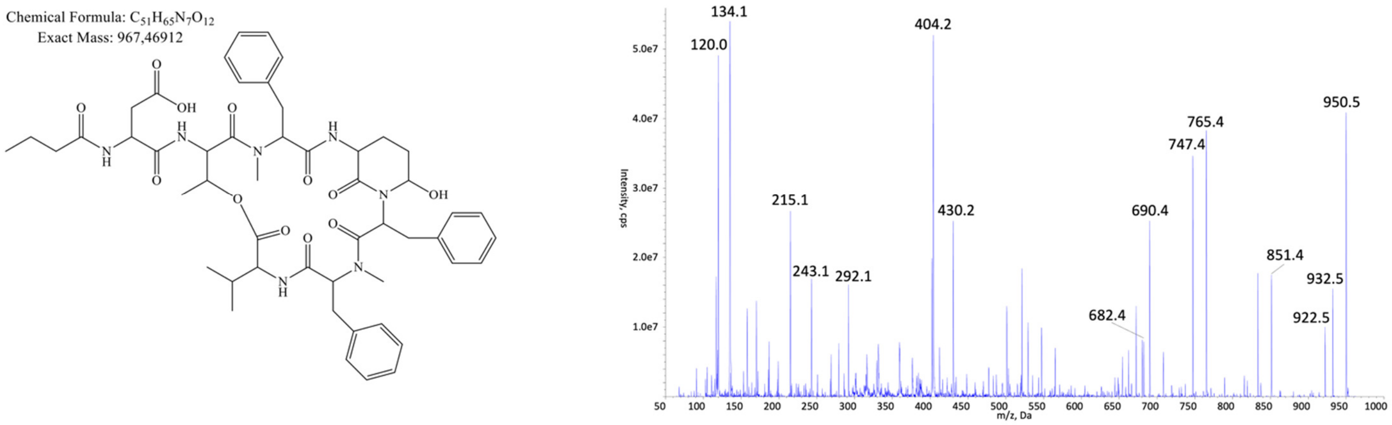


Figure S41. Structure and enhanced product ion mass spectrum of the cyanopeptolin CP 967b.

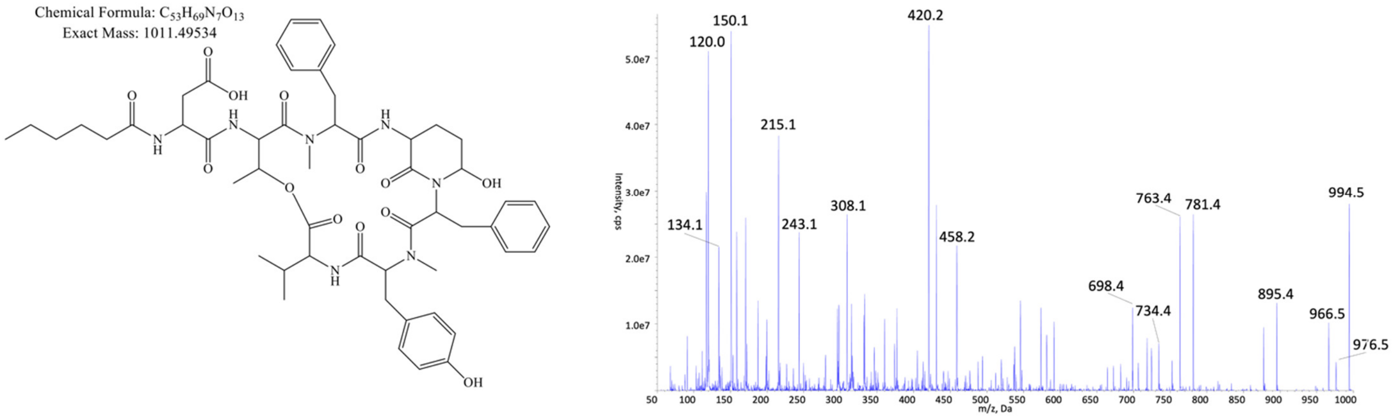


Figure S42. Structure and enhanced product ion mass spectrum of the cyanopeptolin CP 1011c.

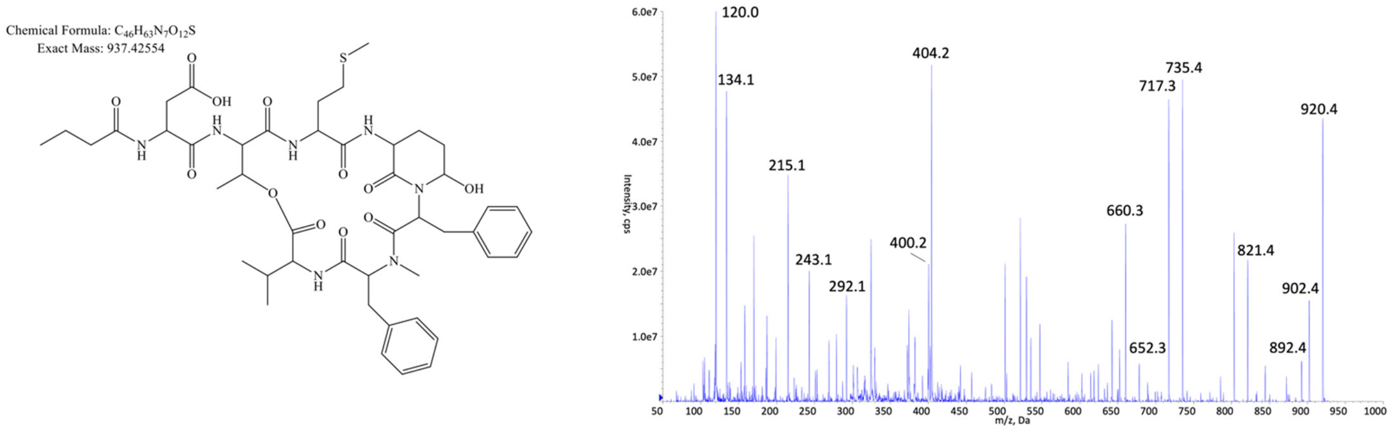


Figure S43. Structure and enhanced product ion mass spectrum of the cyanopeptolin CP 937.

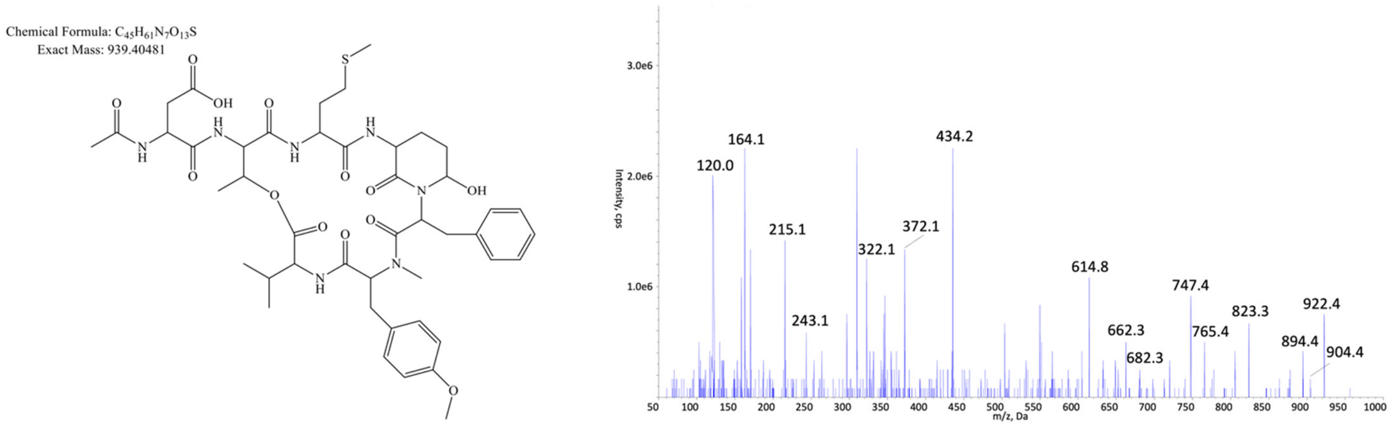


Figure S44. Structure and enhanced product ion mass spectrum of the cyanopeptolin CP 939.

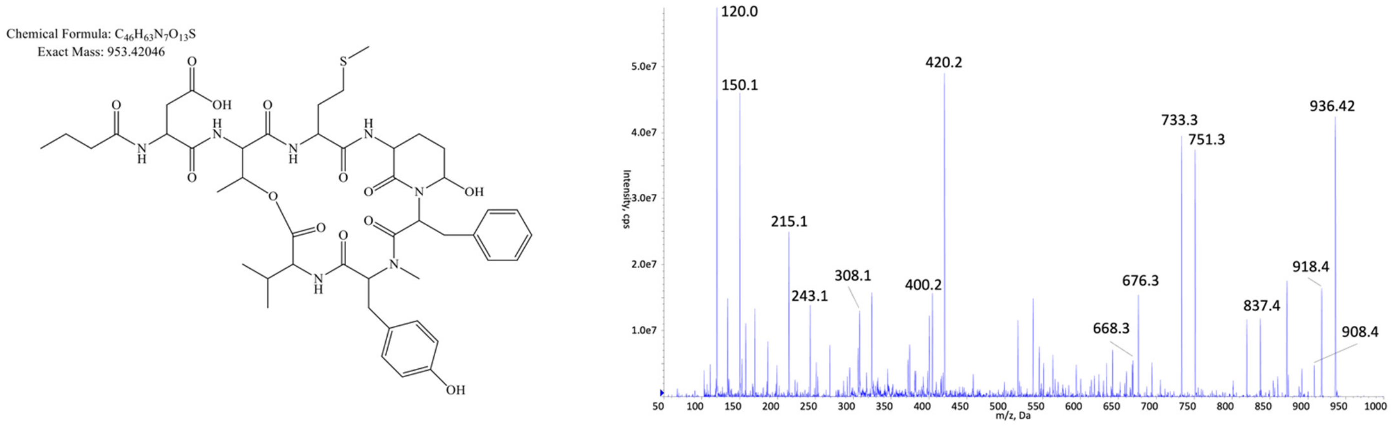


Figure S45. Structure and enhanced product ion mass spectrum of the cyanopeptolin CP 953b.

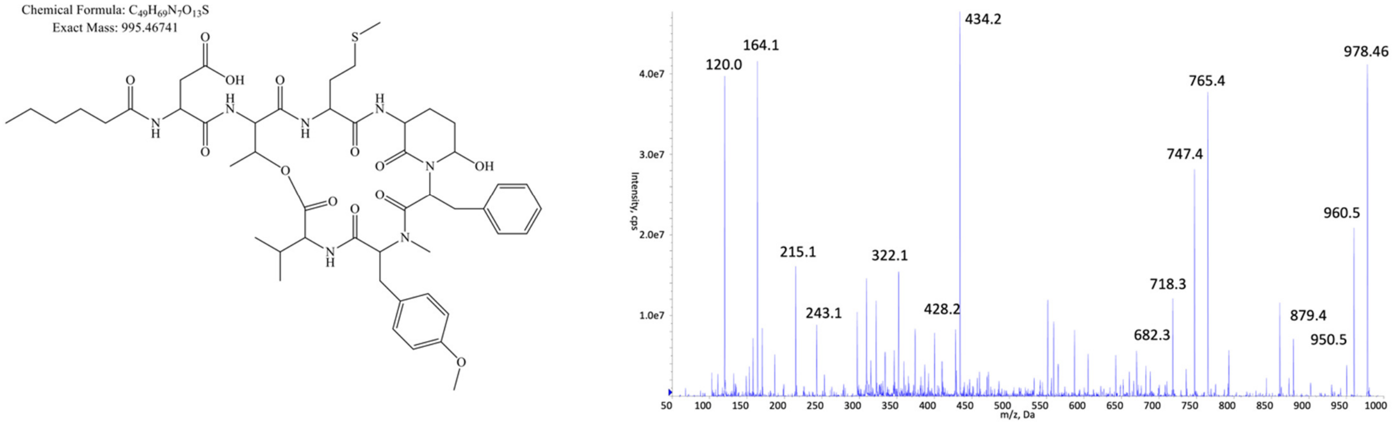


Figure S46. Structure and enhanced product ion mass spectrum of the cyanopeptolin CP 995.

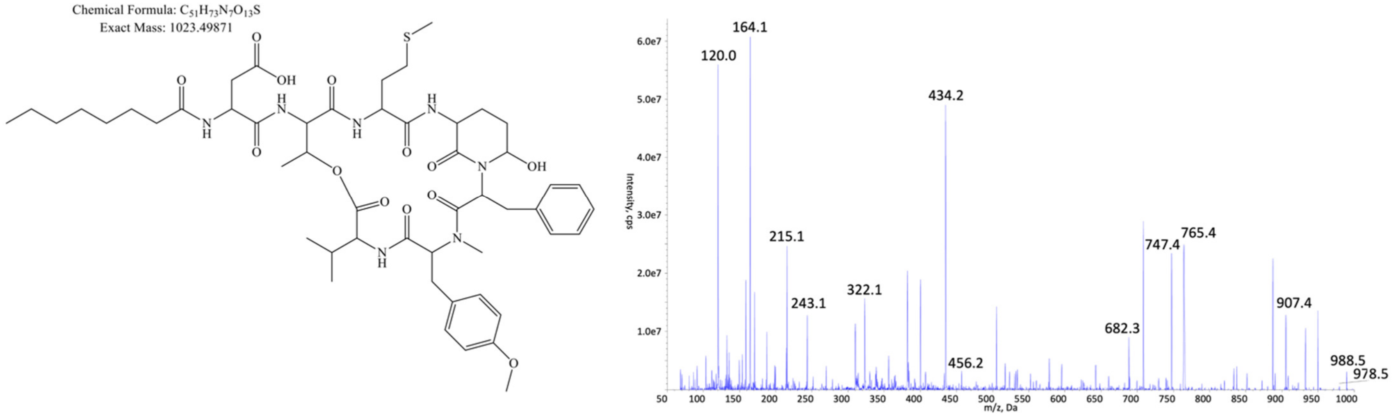


Figure S47. Structure and enhanced product ion mass spectrum of the cyanopeptolin CP 1023.

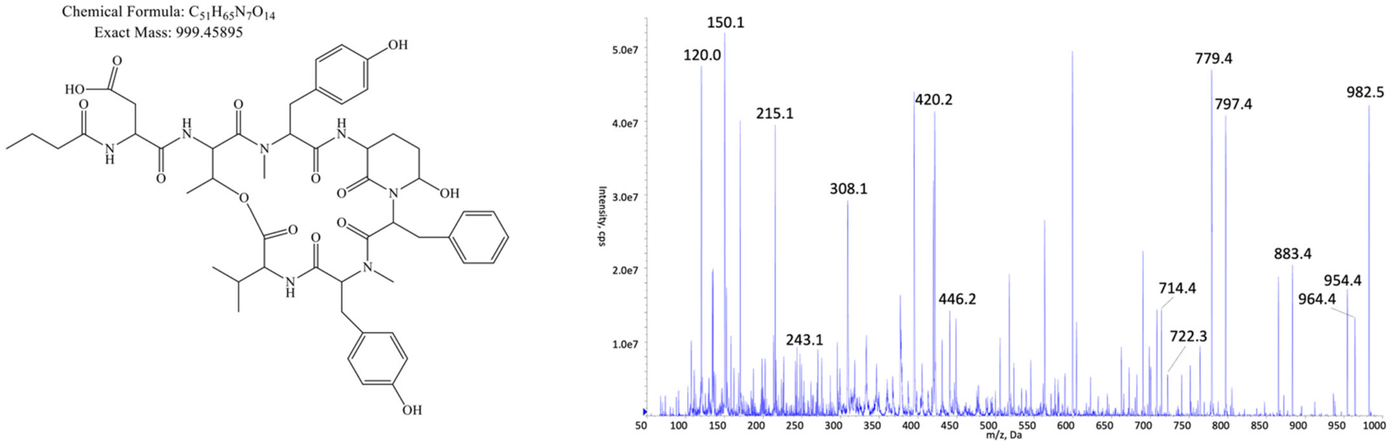


Figure S48. Structure and enhanced product ion mass spectrum of the cyanopeptolin CP 999d.

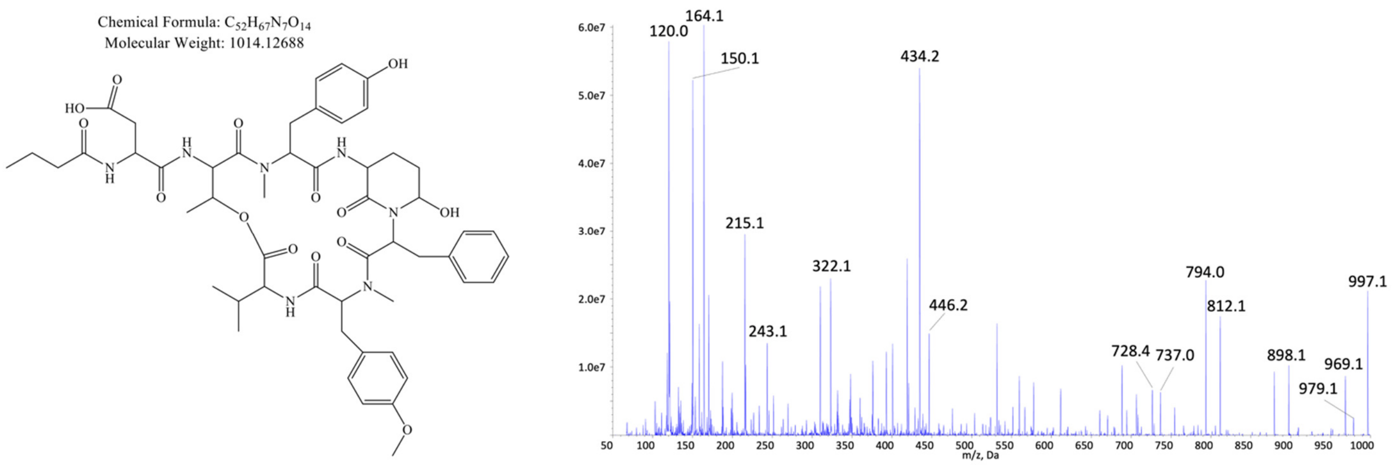


Figure S49. Structure and enhanced product ion mass spectrum of the cyanopeptolin CP 1014.

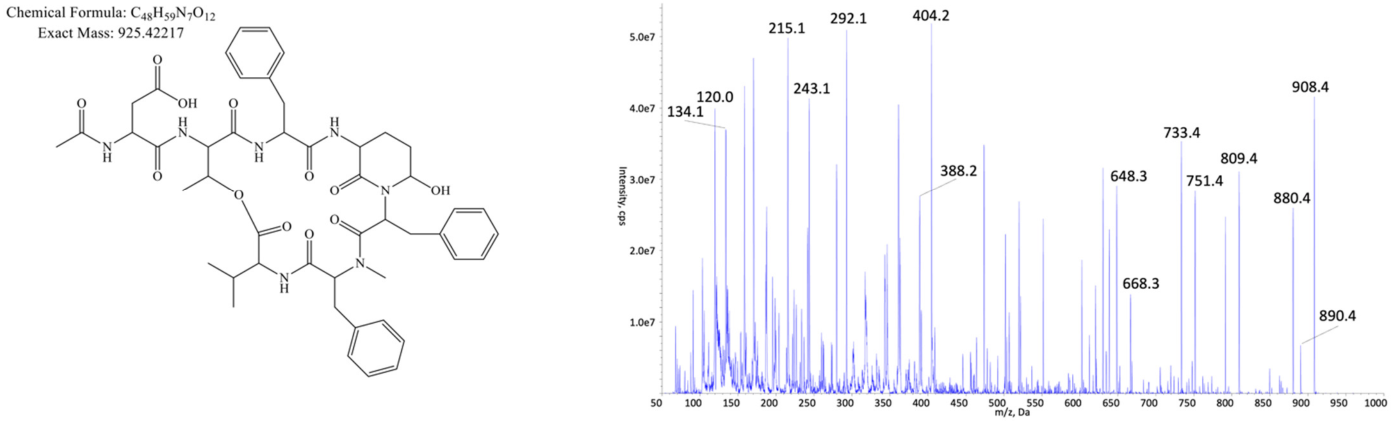


Figure S50. Structure and enhanced product ion mass spectrum of the cyanopeptolin CP 925.

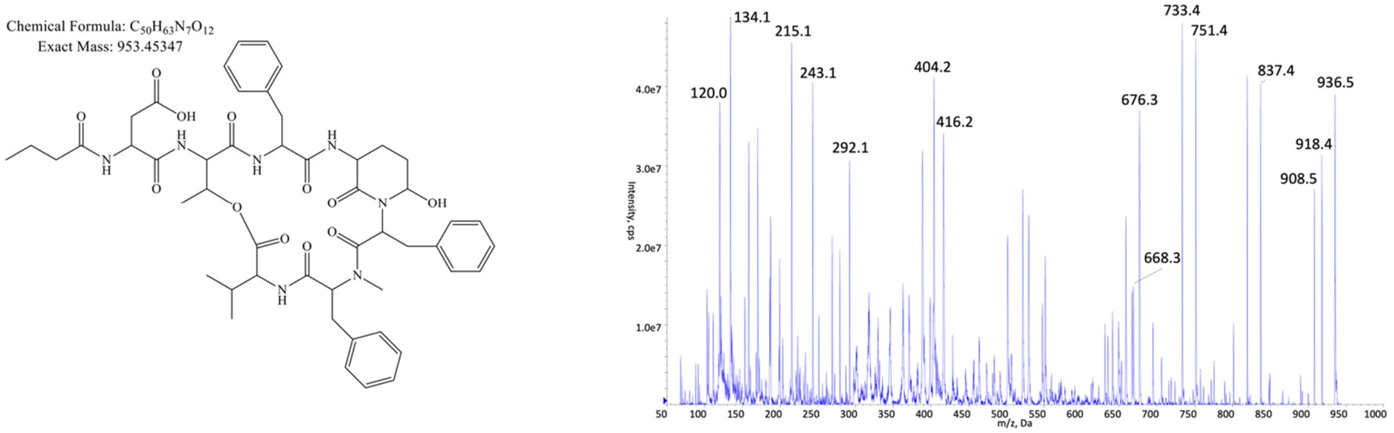


Figure S51. Structure and enhanced product ion mass spectrum of the cyanopeptolin CP 953.

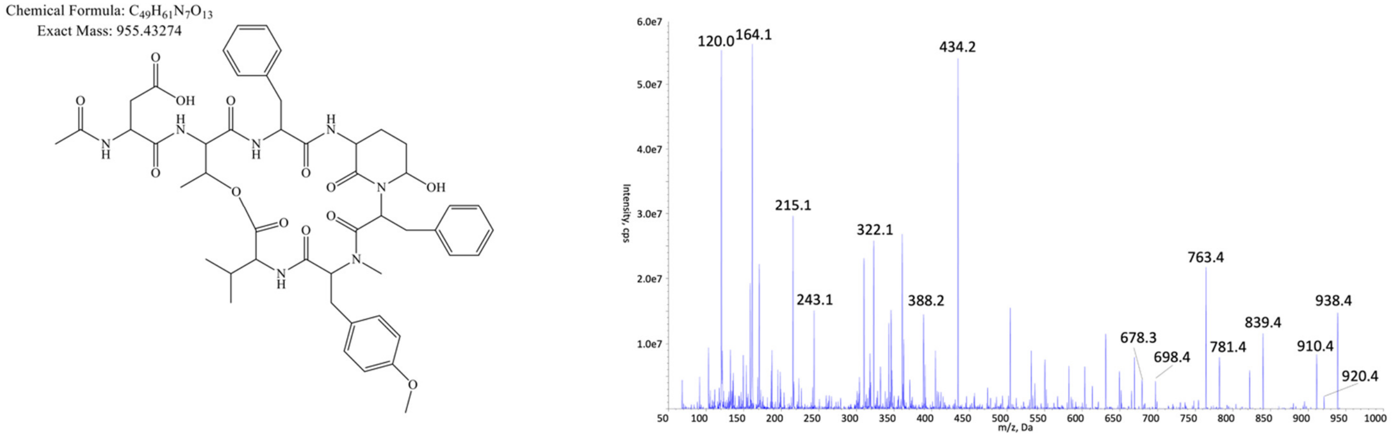


Figure S52. Structure and enhanced product ion mass spectrum of the cyanopeptolin CP 955.

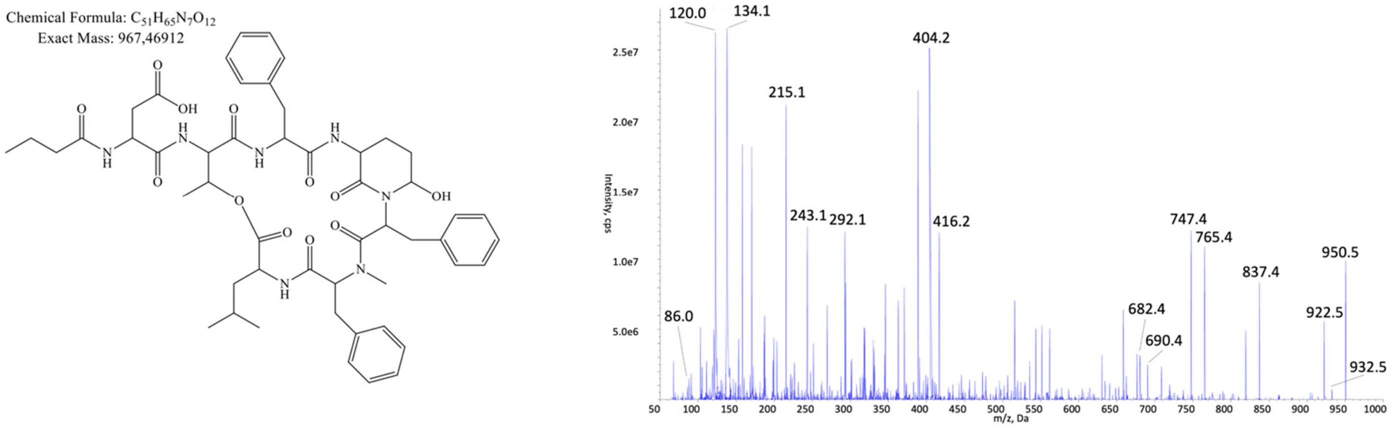


Figure S53. Structure and enhanced product ion mass spectrum of the cyanopeptolin CP 967.

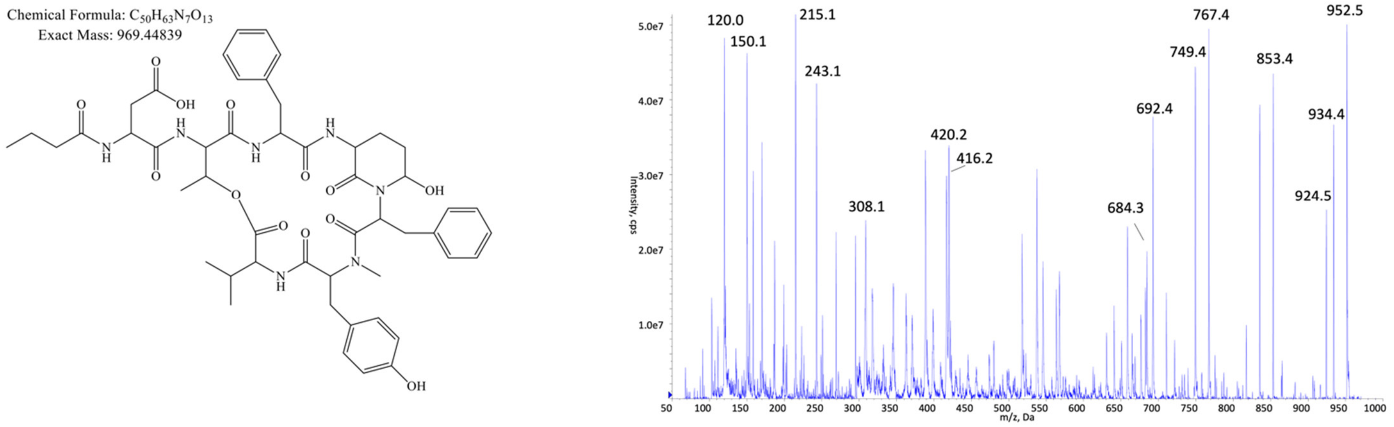


Figure S54. Structure and enhanced product ion mass spectrum of the cyanopeptolin CP 969b.

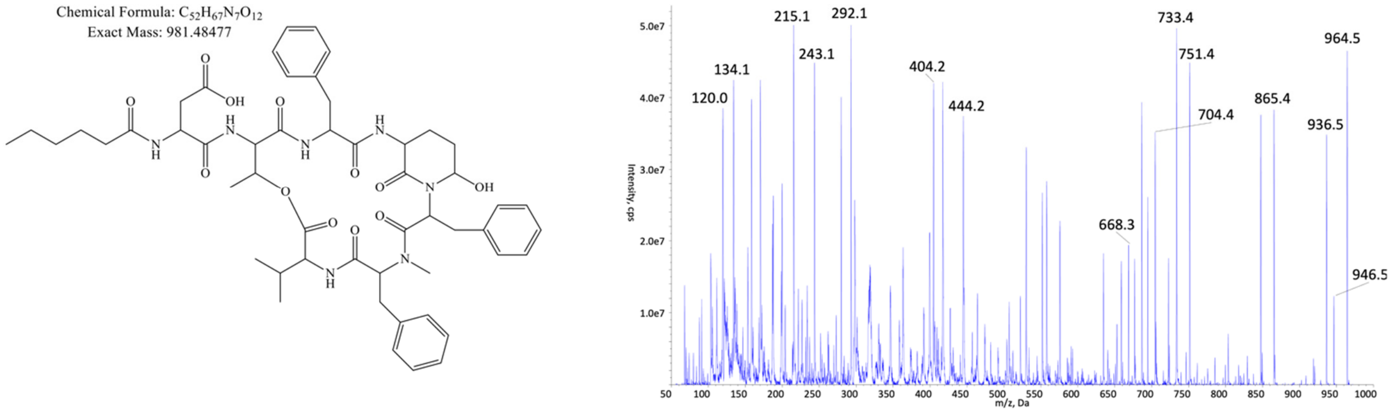


Figure S55. Structure and enhanced product ion mass spectrum of the cyanopeptolin CP 981.

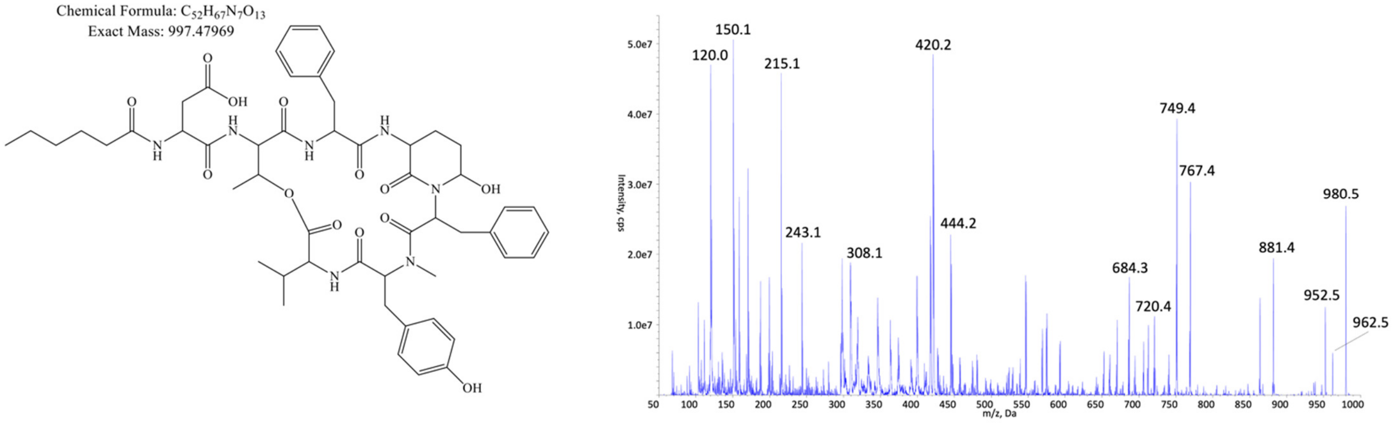


Figure S56. Structure and enhanced product ion mass spectrum of the cyanopeptolin CP 997.

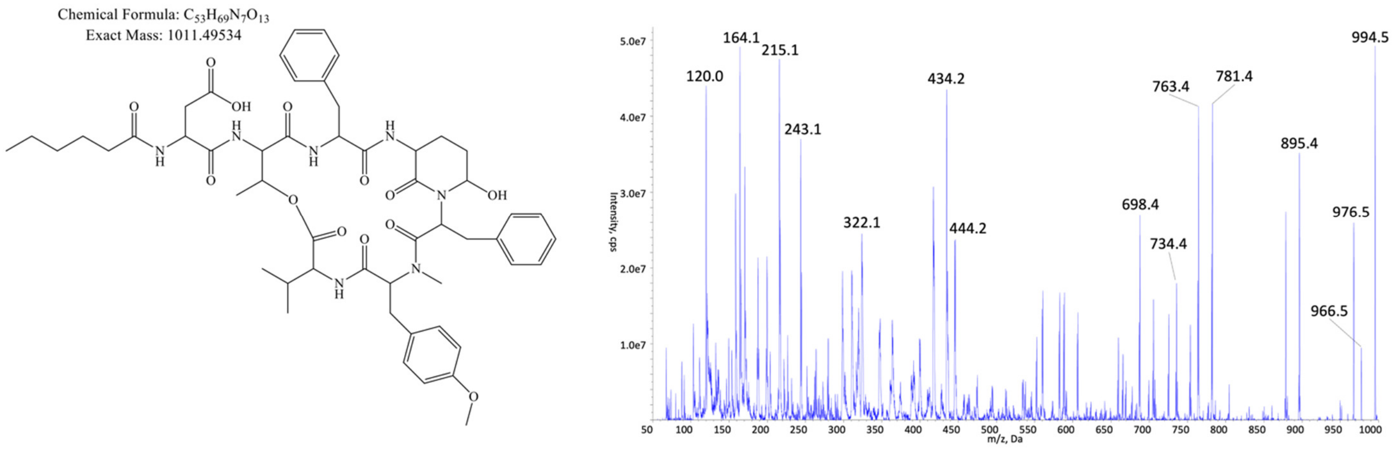


Figure S57. Structure and enhanced product ion mass spectrum of the cyanopeptolin CP 1011.

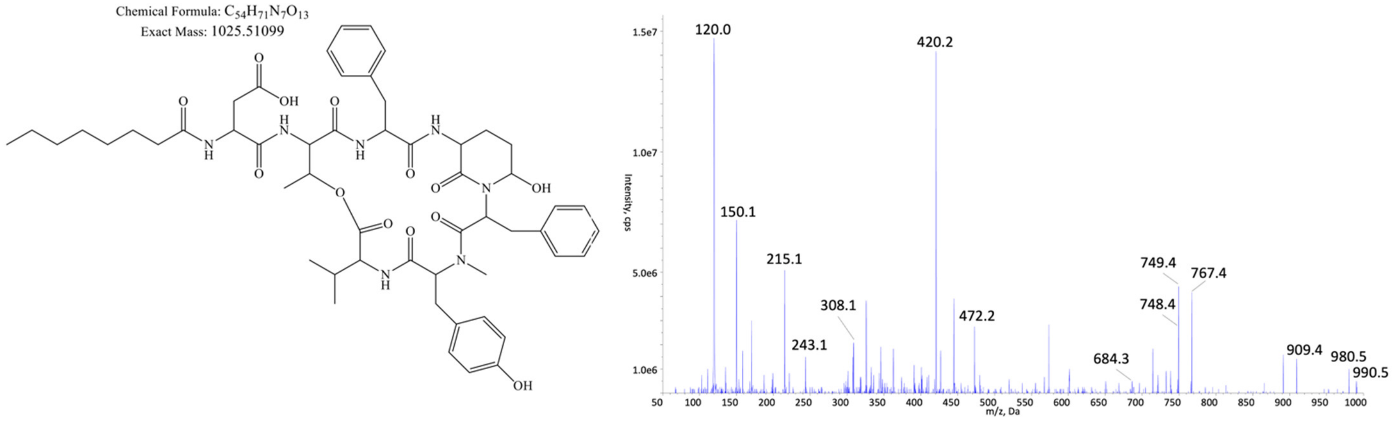


Figure S58. Structure and enhanced product ion mass spectrum of the cyanopeptolin CP 1025b.

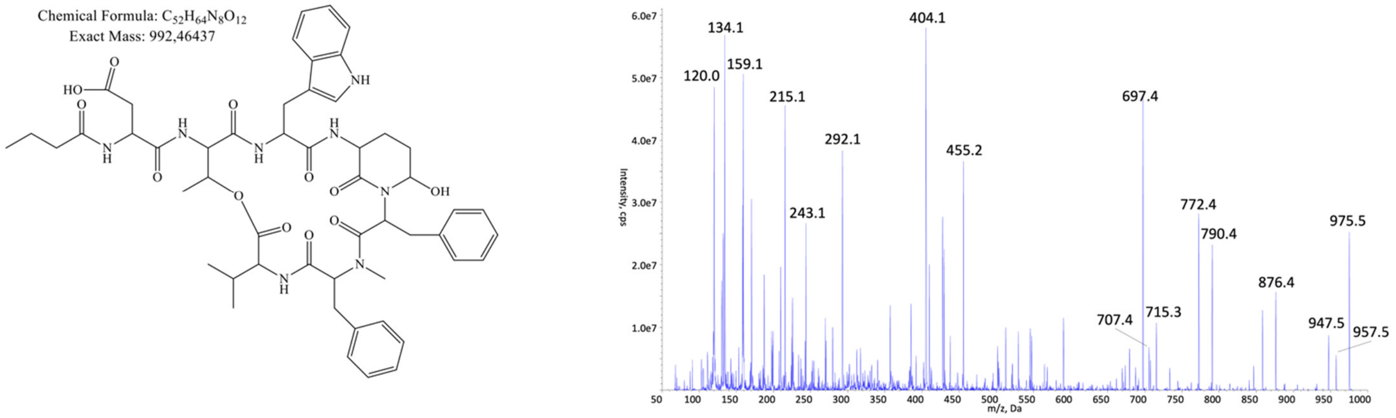


Figure S59. Structure and enhanced product ion mass spectrum of the cyanopeptolin CP 992d.

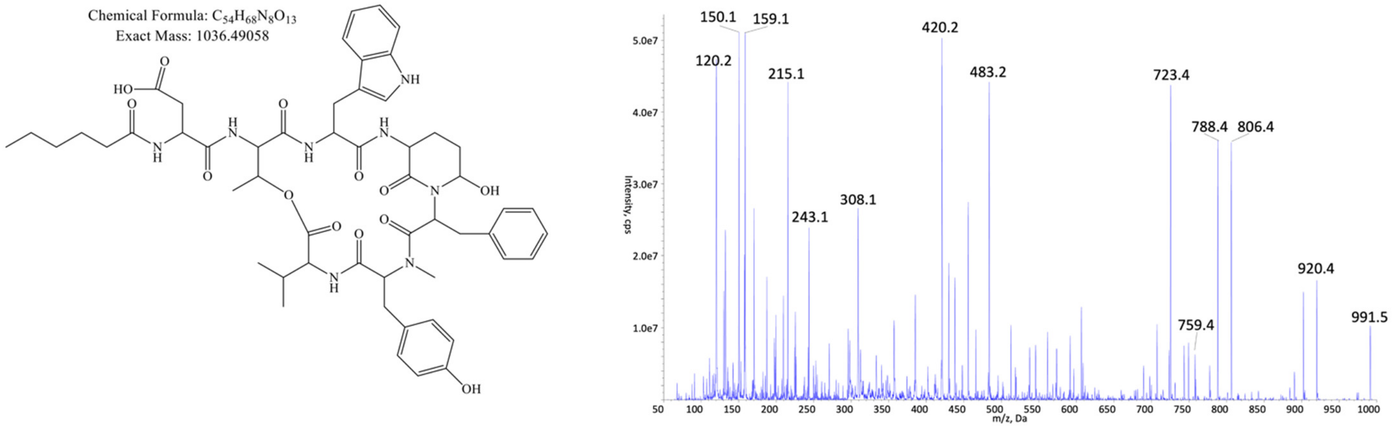


Figure S60. Structure and enhanced product ion mass spectrum of the cyanopeptolin CP 1036c.

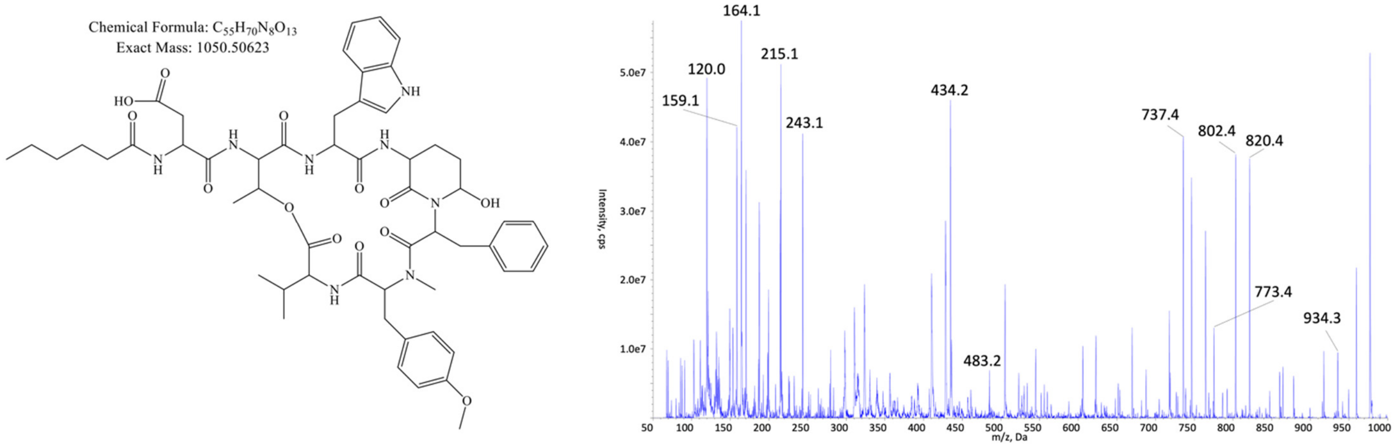


Figure S61. Structure and enhanced product ion mass spectrum of the cyanopeptolin CP 1050.

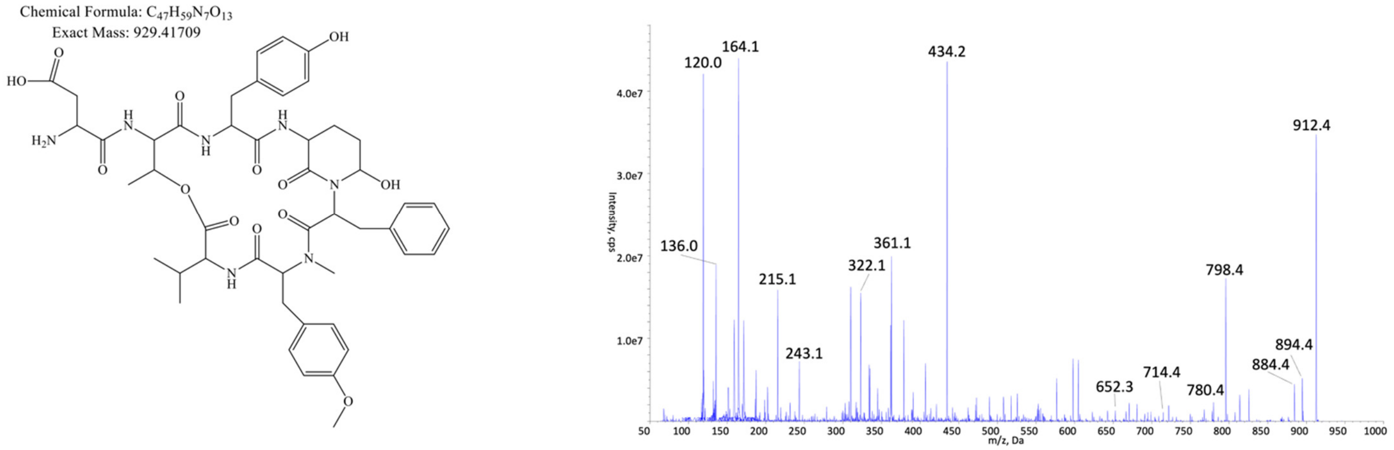


Figure S62. Structure and enhanced product ion mass spectrum of the cyanopeptolin CP 929.

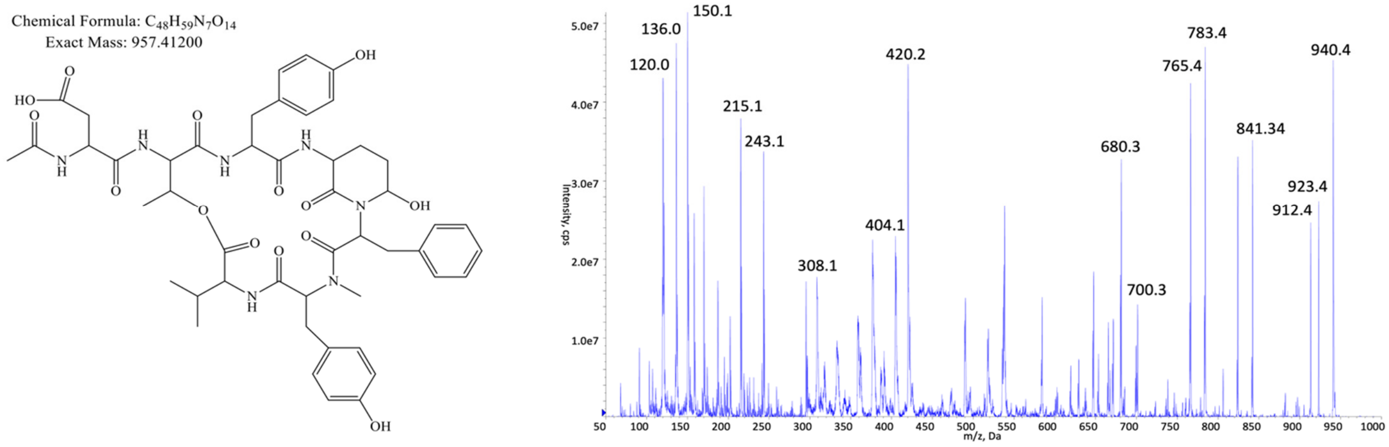


Figure S63. Structure and enhanced product ion mass spectrum of the cyanopeptolin CP 957.

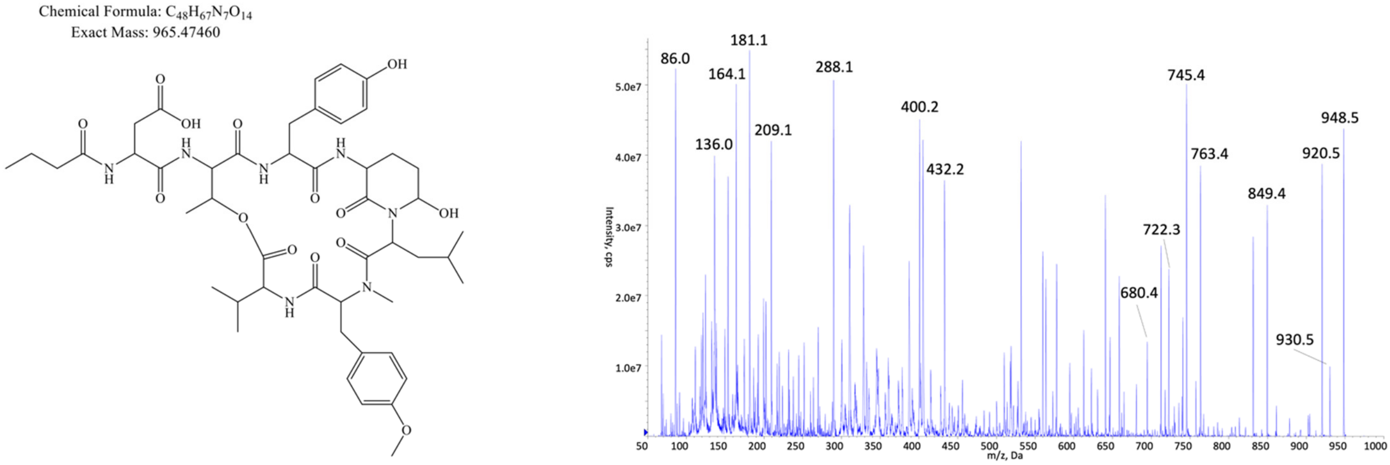


Figure S64. Structure and enhanced product ion mass spectrum of the cyanopeptolin CP 965.

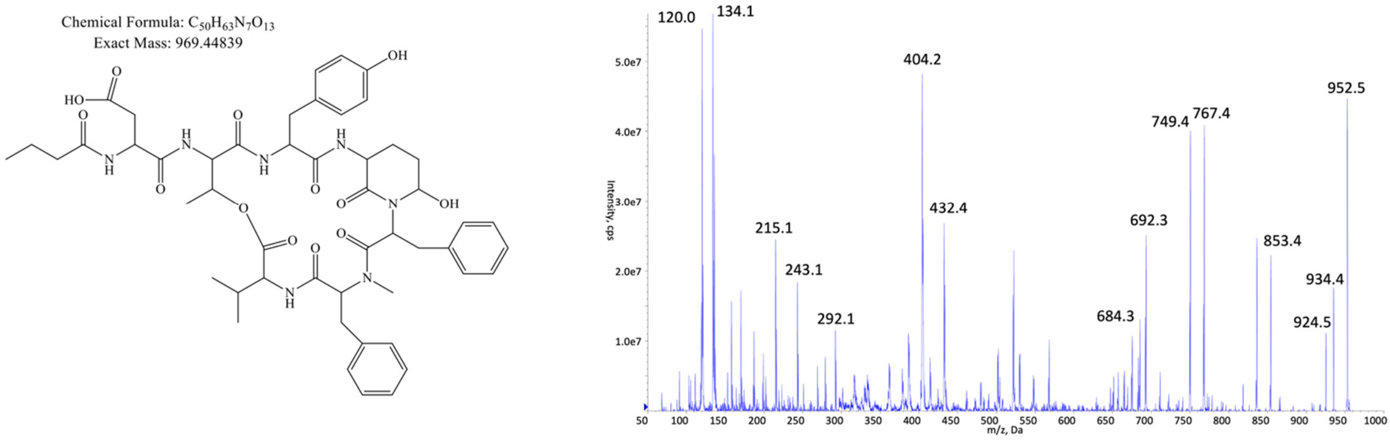


Figure S65. Structure and enhanced product ion mass spectrum of the cyanopeptolin CP 969 [8].

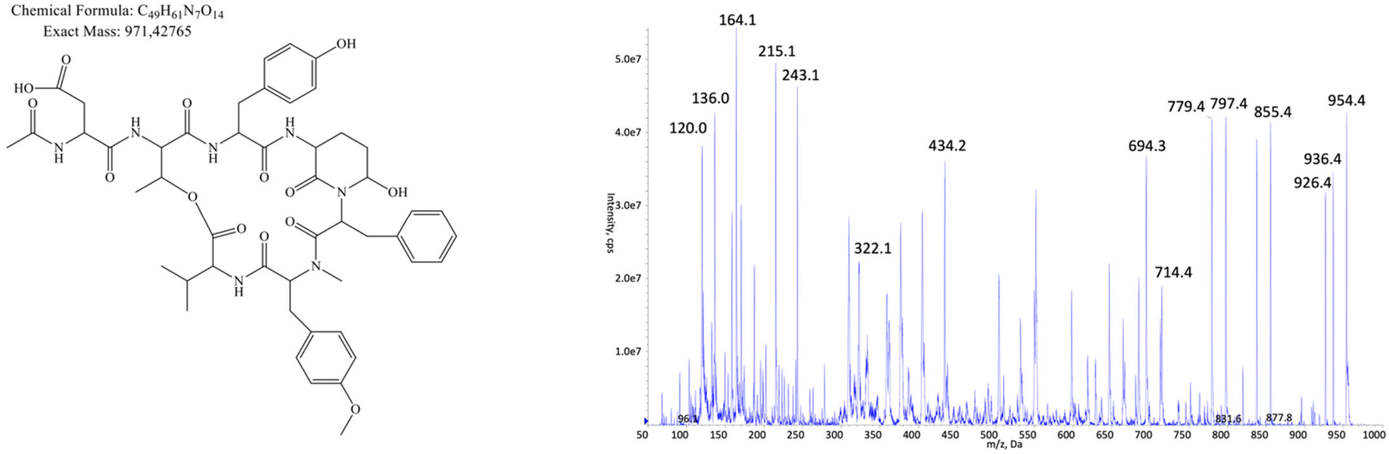


Figure S66. Structure and enhanced product ion mass spectrum of the cyanopeptolin CP 971.

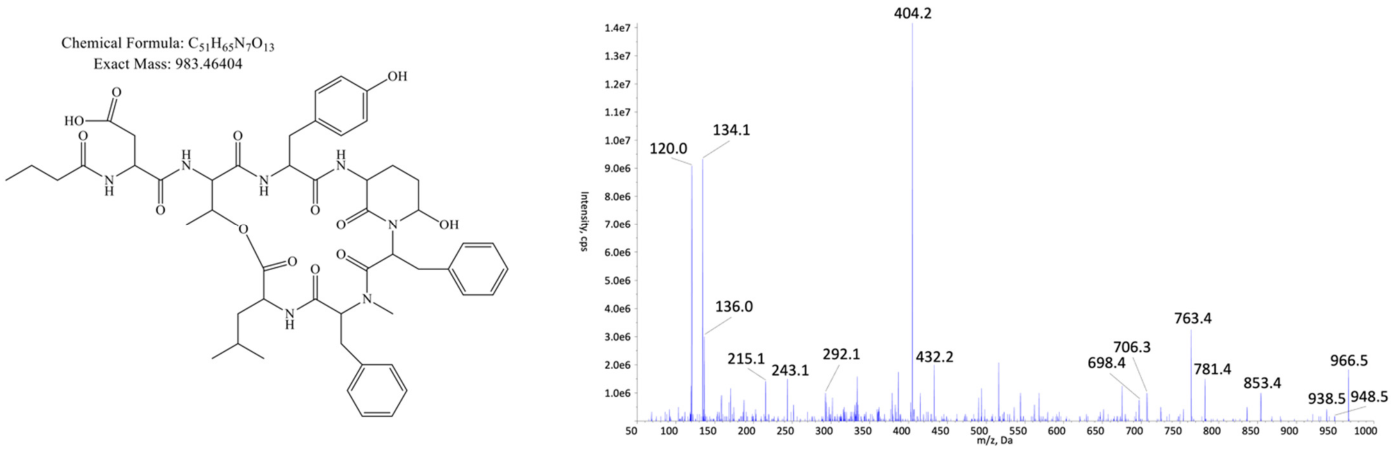


Figure S67. Structure and enhanced product ion mass spectrum of the cyanopeptolin CP 983b.

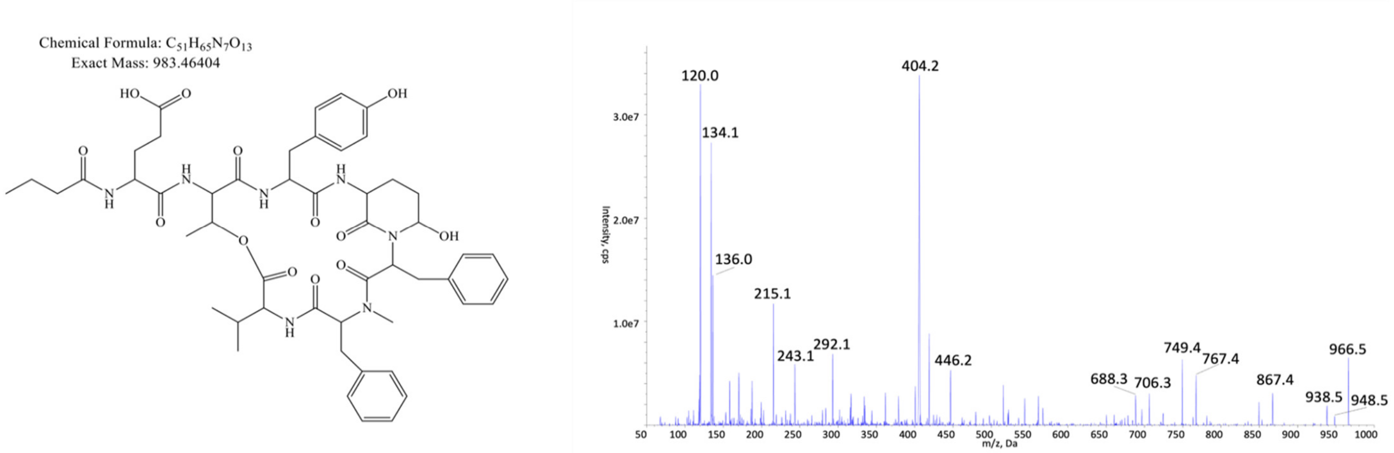


Figure S68. Structure and enhanced product ion mass spectrum of the cyanopeptolin CP 983c.

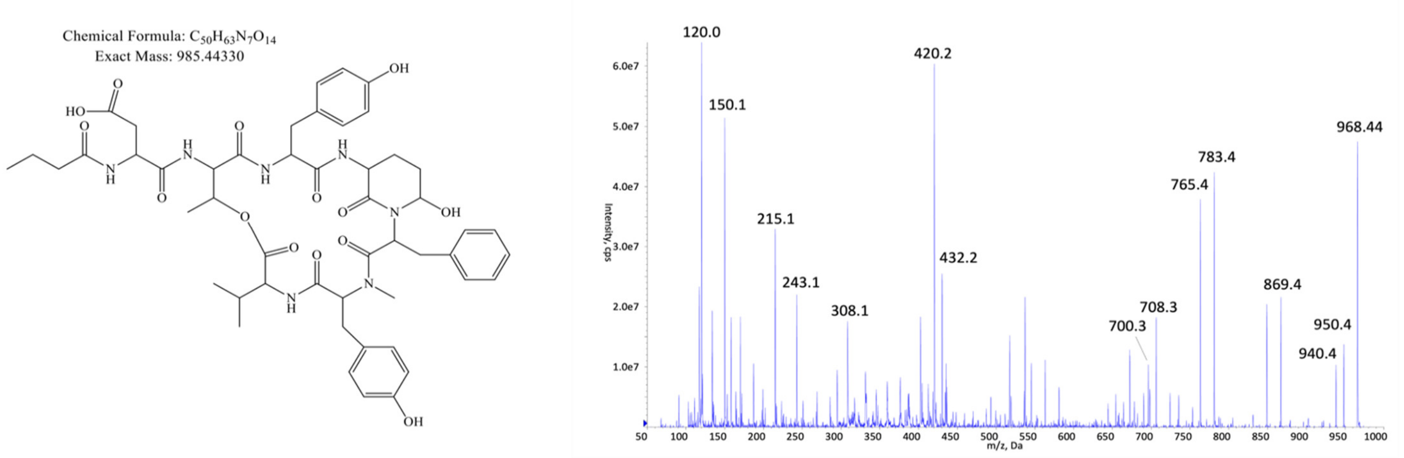


Figure S69. Structure and enhanced product ion mass spectrum of the cyanopeptolin CP 985 [8].

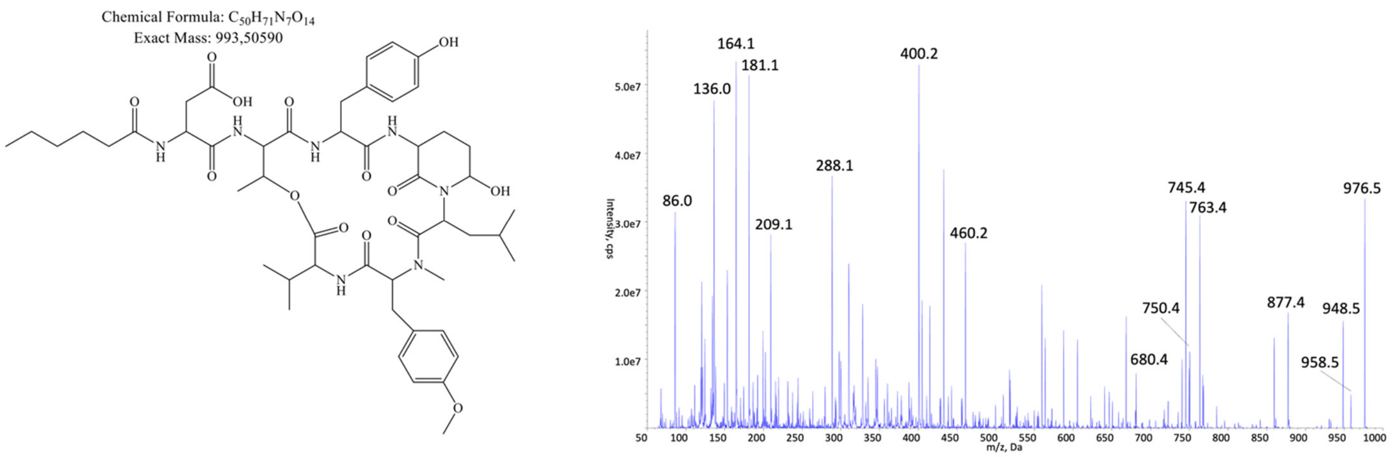


Figure S70. Structure and enhanced product ion mass spectrum of the cyanopeptolin CP 993.

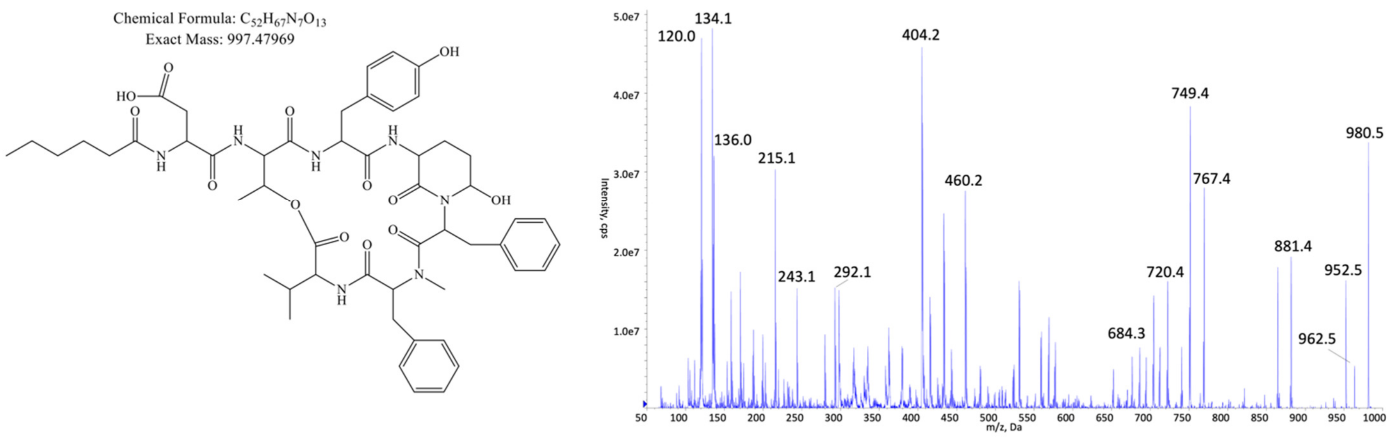


Figure S71. Structure and enhanced product ion mass spectrum of the cyanopeptolin CP 997b.

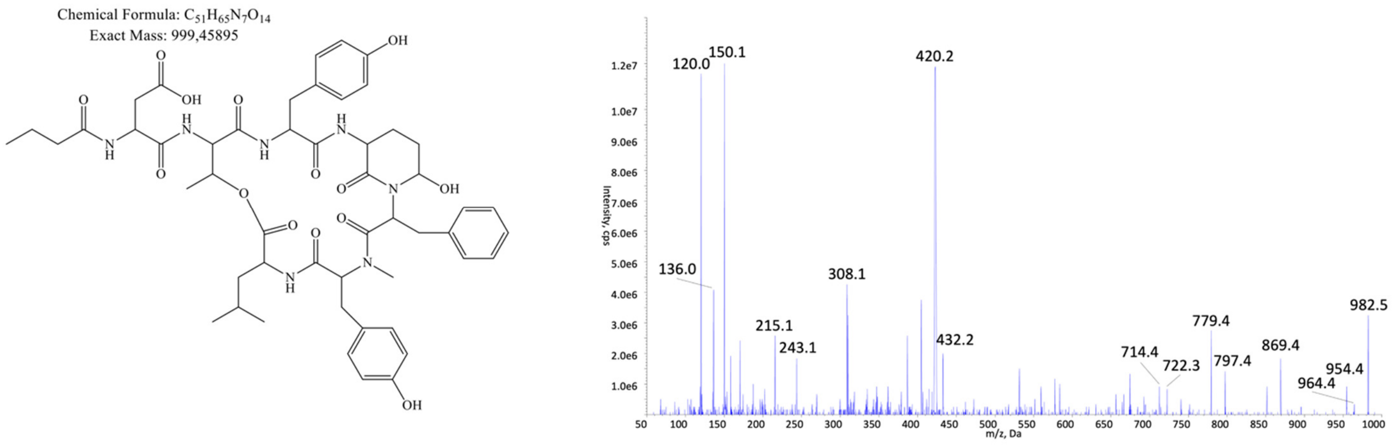


Figure S72. Structure and enhanced product ion mass spectrum of the cyanopeptolin CP 999b.

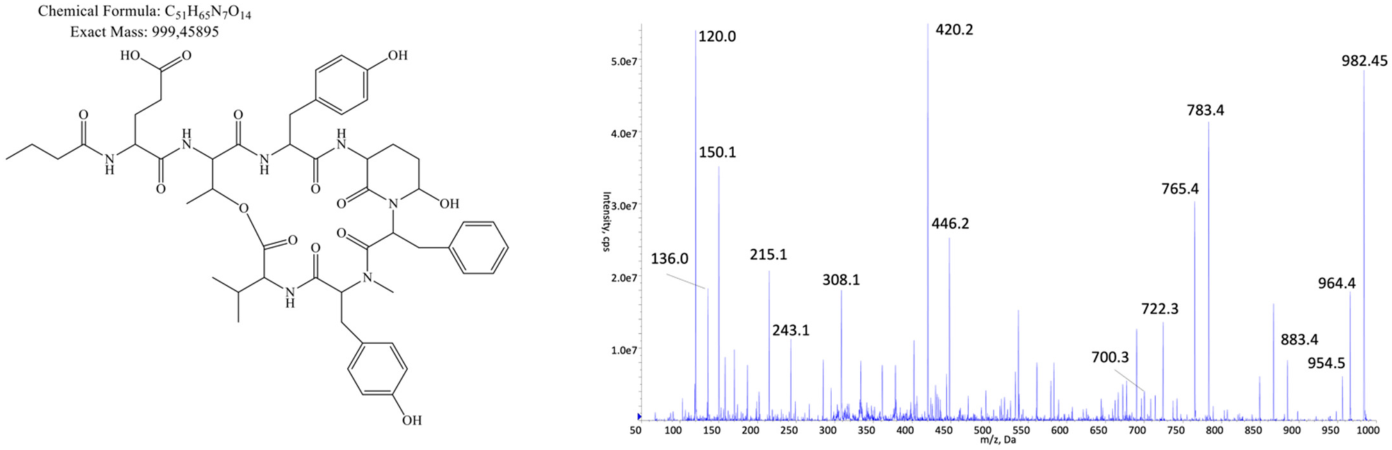


Figure S73. Structure and enhanced product ion mass spectrum of the cyanopeptolin CP 999c.

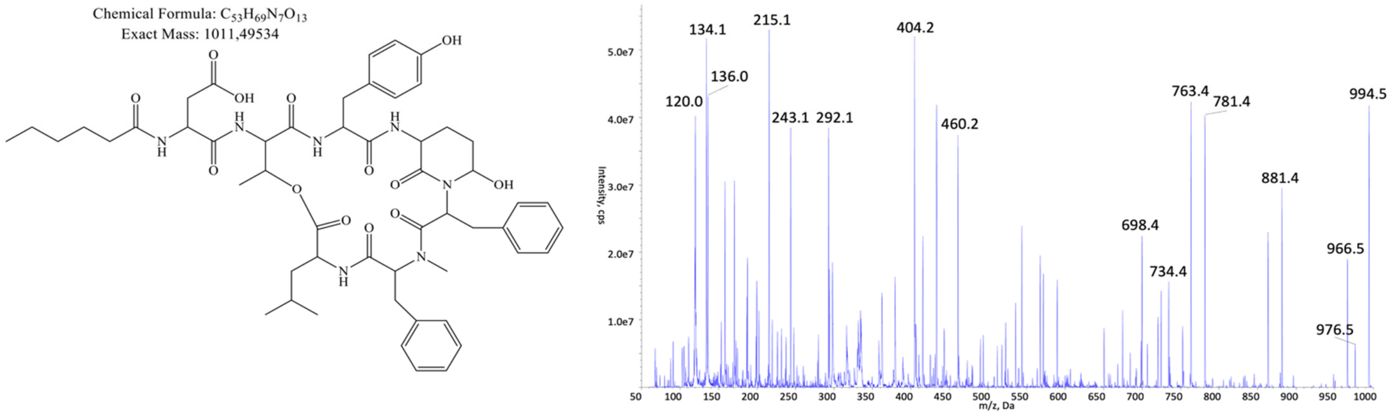


Figure S74. Structure and enhanced product ion mass spectrum of the cyanopeptolin CP 1011b.

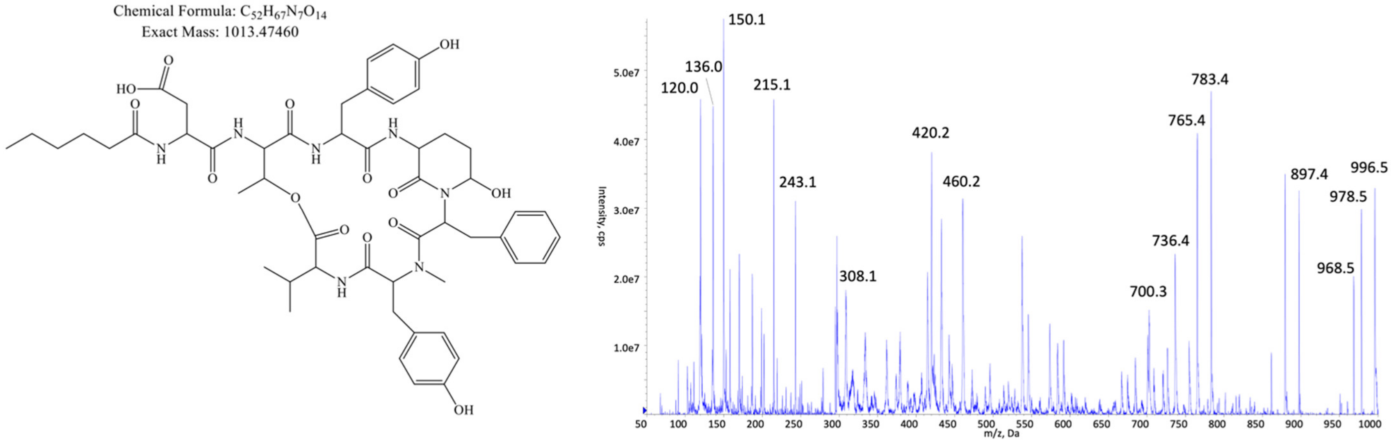


Figure S75. Structure and enhanced product ion mass spectrum of the cyanopeptolin CP 1013 [8].

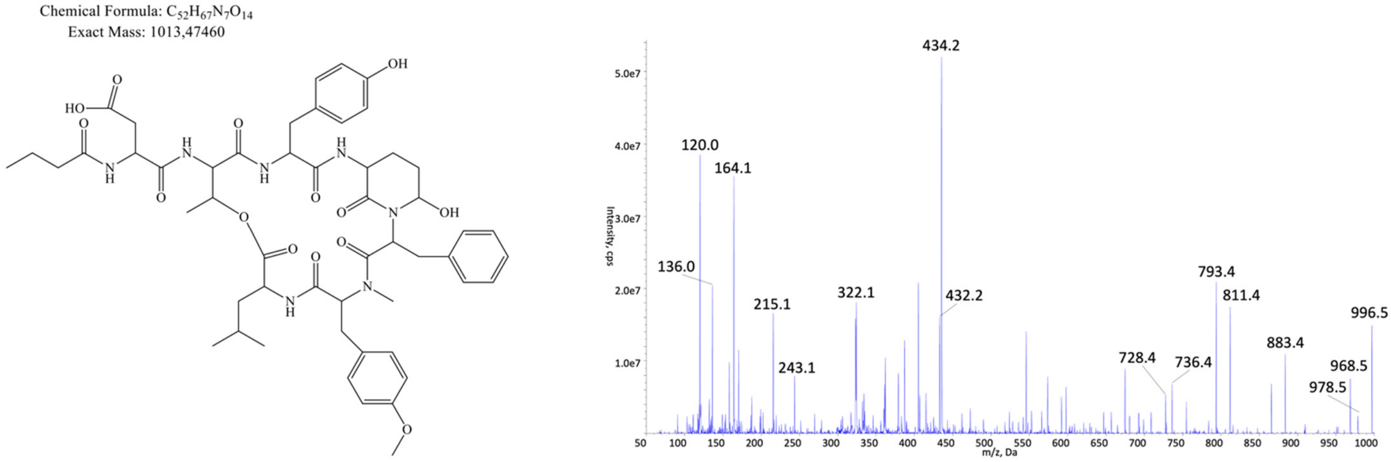


Figure S76. Structure and enhanced product ion mass spectrum of the cyanopeptolin CP 1013b.

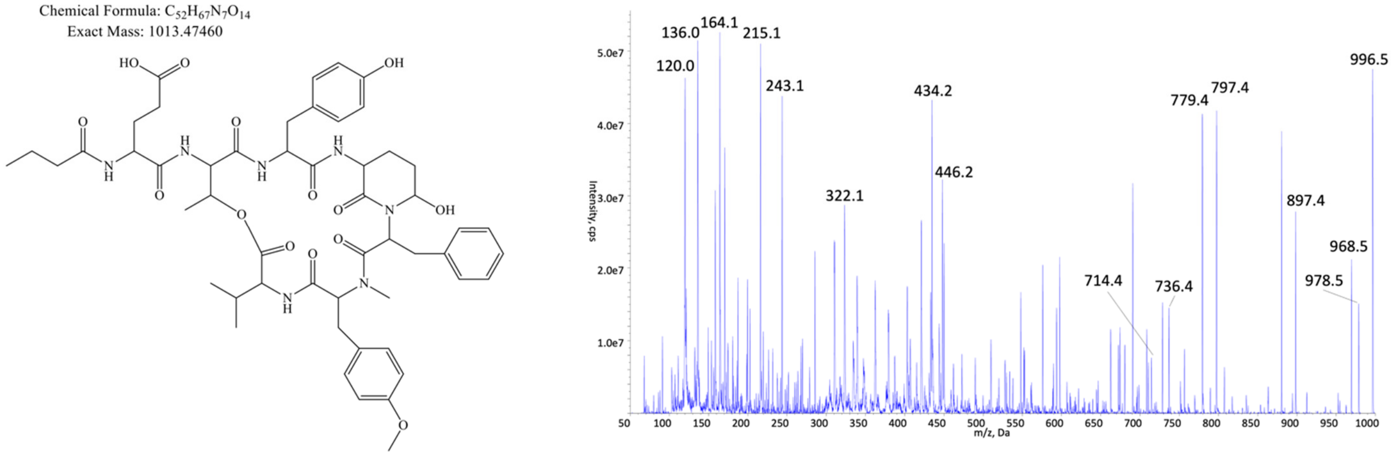


Figure S77. Structure and enhanced product ion mass spectrum of the cyanopeptolin CP 1013c.

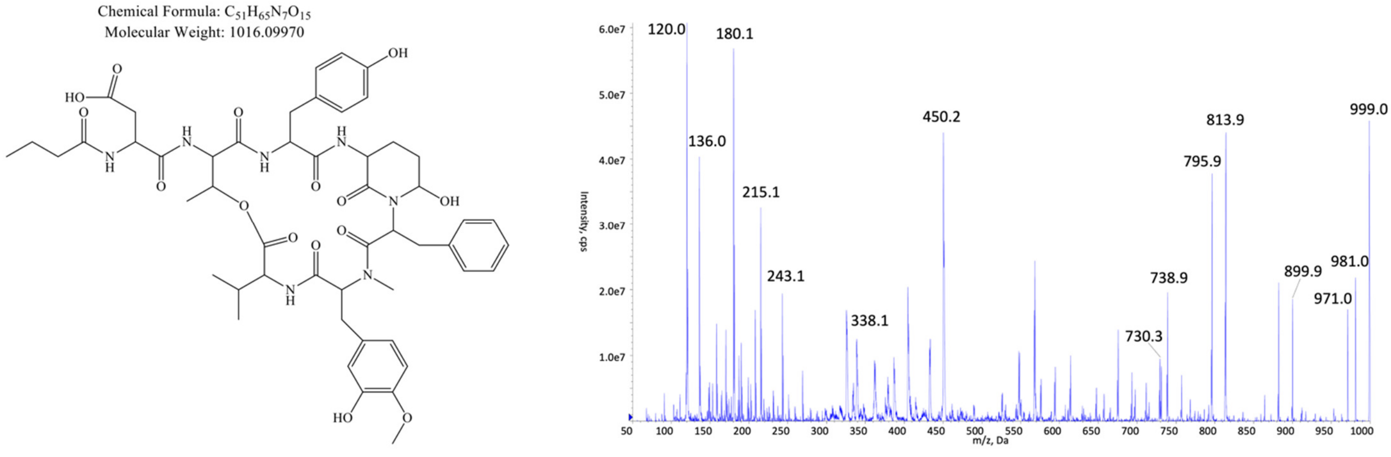


Figure S78. Structure and enhanced product ion mass spectrum of the cyanopeptolin CP 1016.

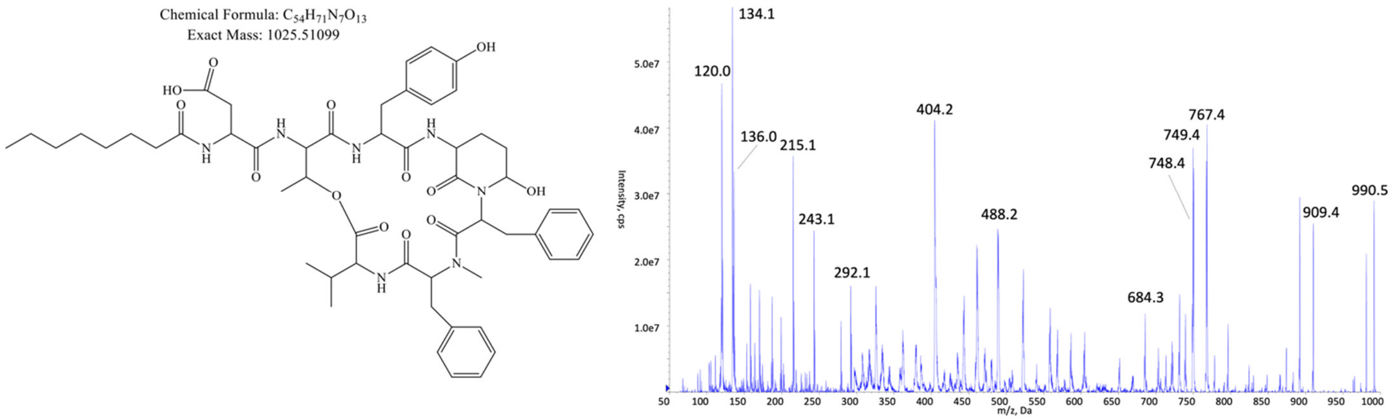


Figure S79. Structure and enhanced product ion mass spectrum of the cyanopeptolin CP 1025.

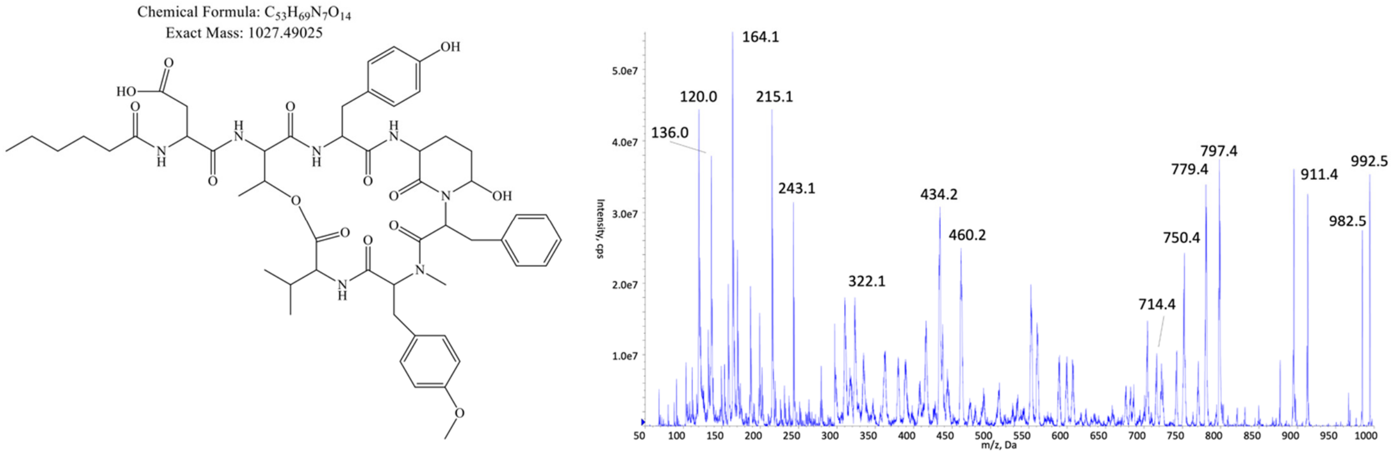


Figure S80. Structure and enhanced product ion mass spectrum of the cyanopeptolin CP 1027 [8].

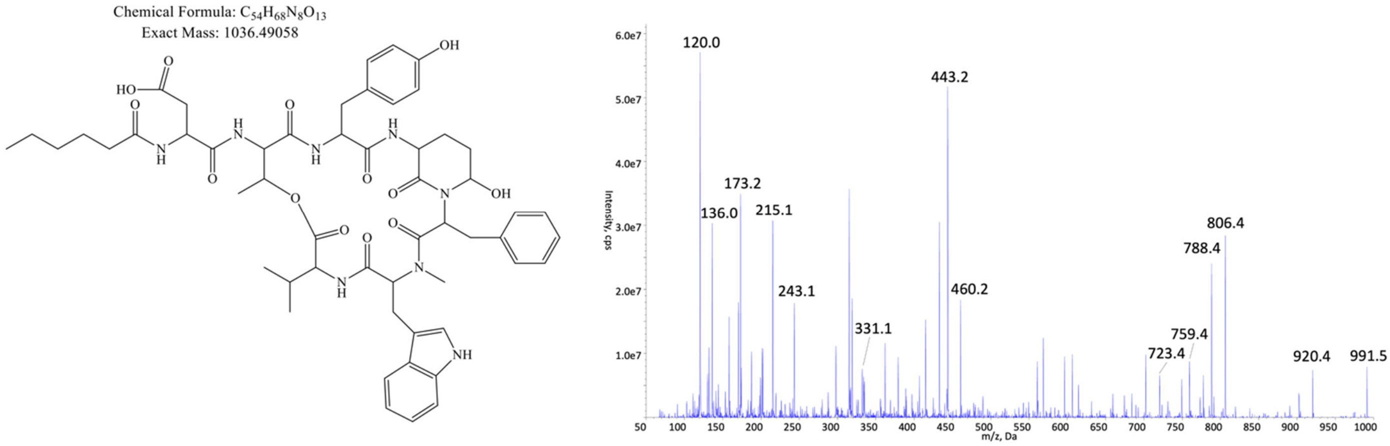


Figure S81. Structure and enhanced product ion mass spectrum of the cyanopeptolin CP 1036b.

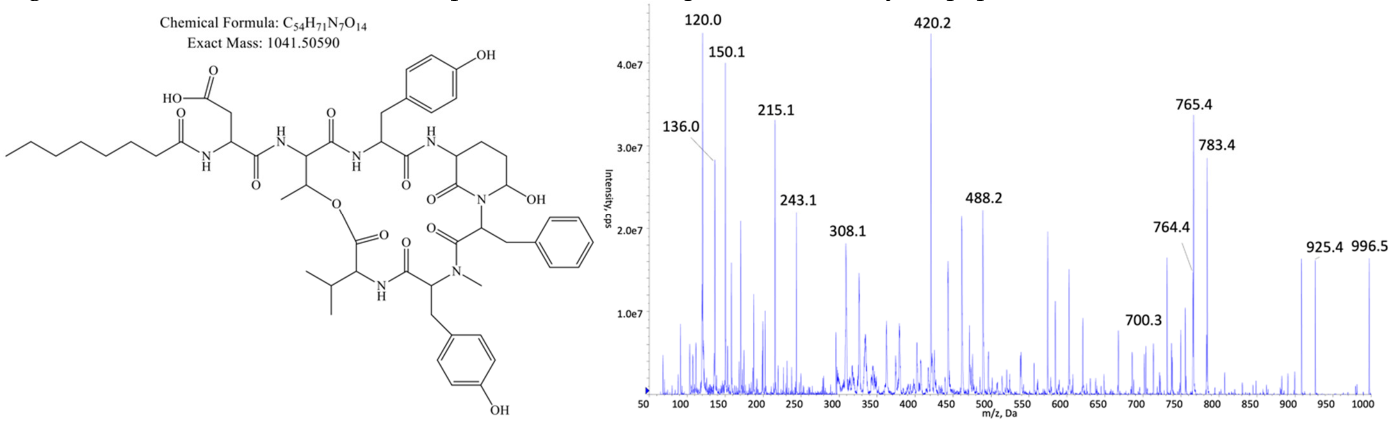


Figure S82. Structure and enhanced product ion mass spectrum of the cyanopeptolin CP 1041.

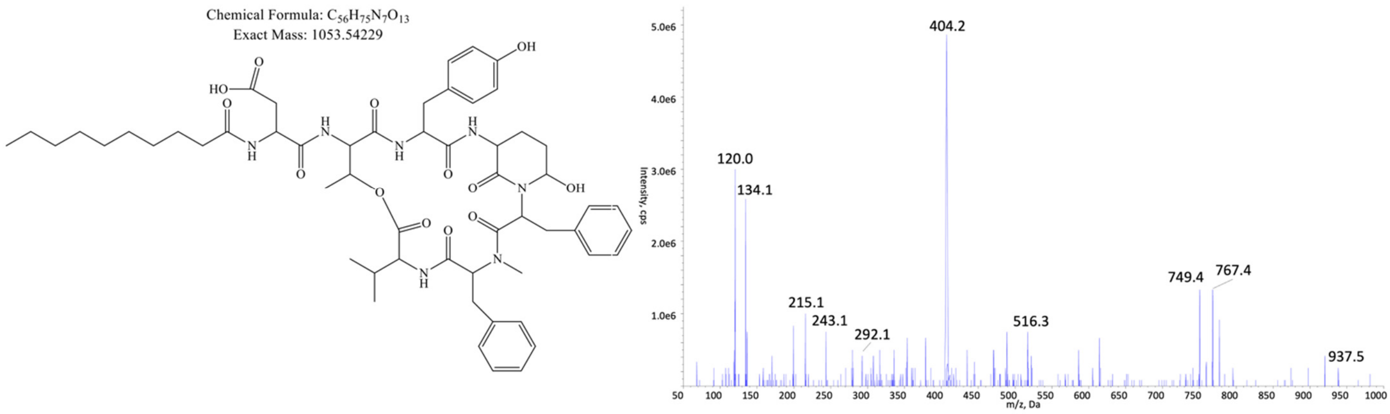


Figure S83. Structure and enhanced product ion mass spectrum of the cyanopeptolin CP 1053.

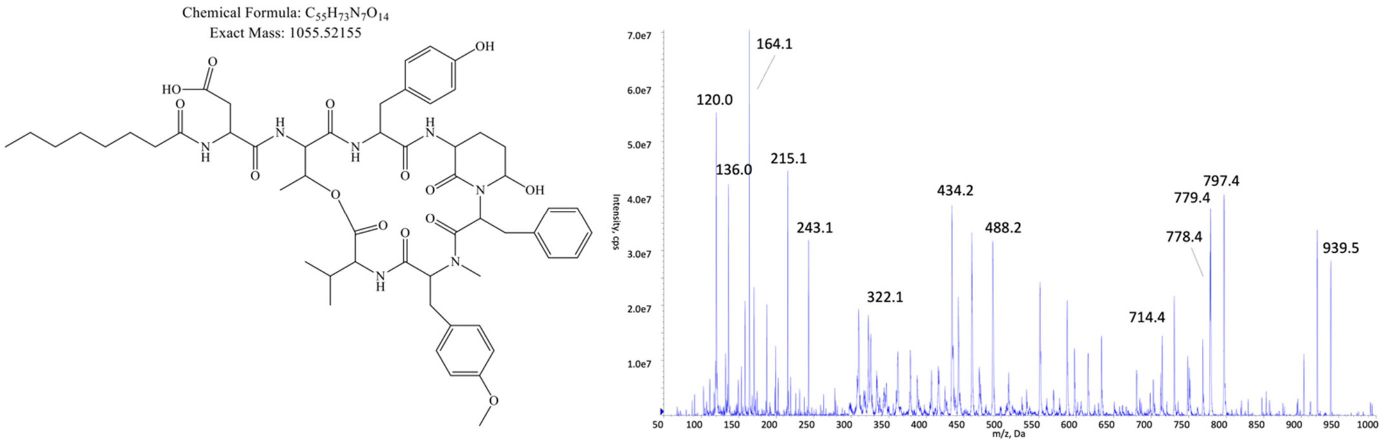


Figure S84. Structure and enhanced product ion mass spectrum of the cyanopeptolin CP 1055.

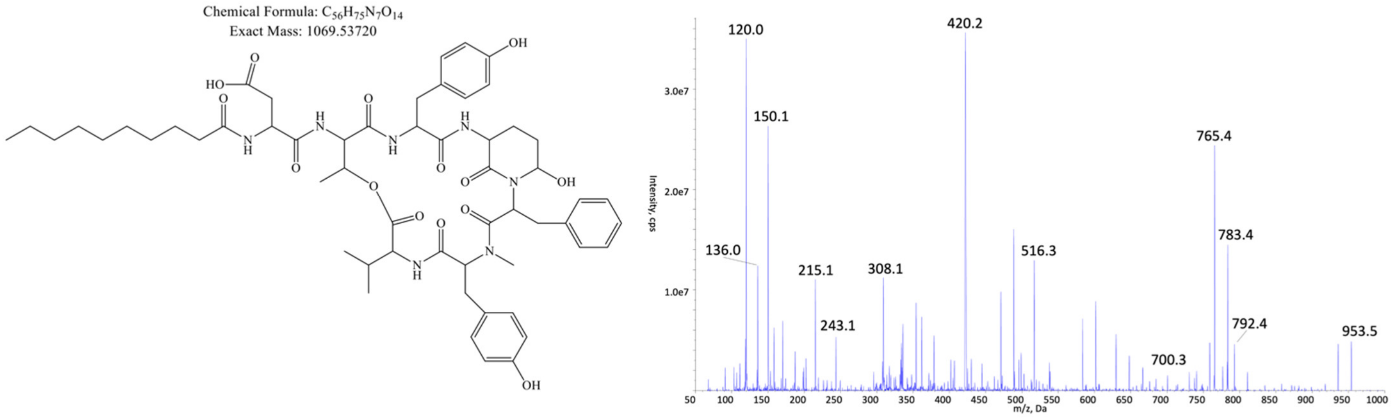


Figure S85. Structure and enhanced product ion mass spectrum of the cyanopeptolin CP 1069.

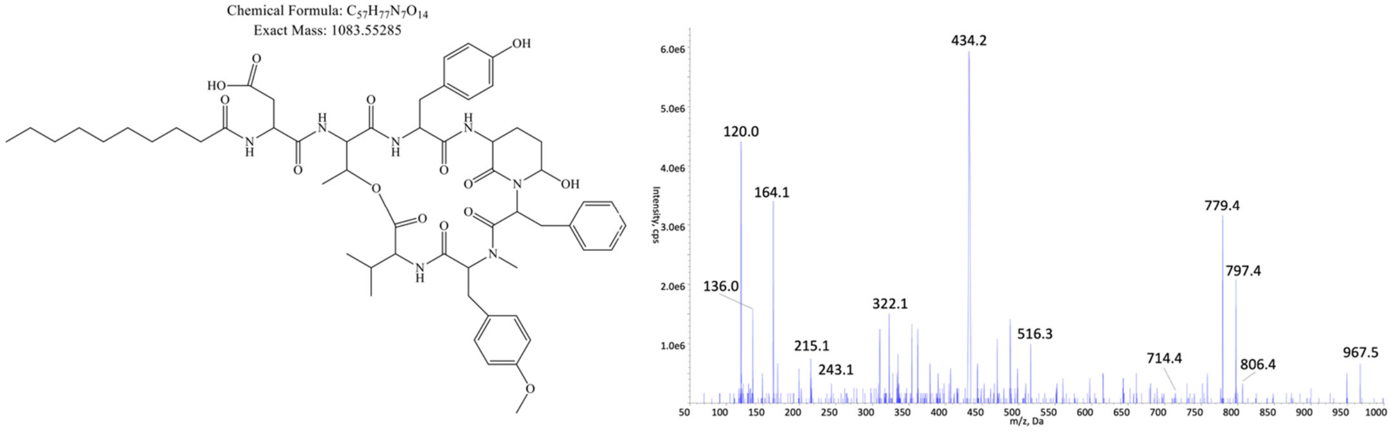


Figure S86. Structure and enhanced product ion mass spectrum of the cyanopeptolin CP 1083.

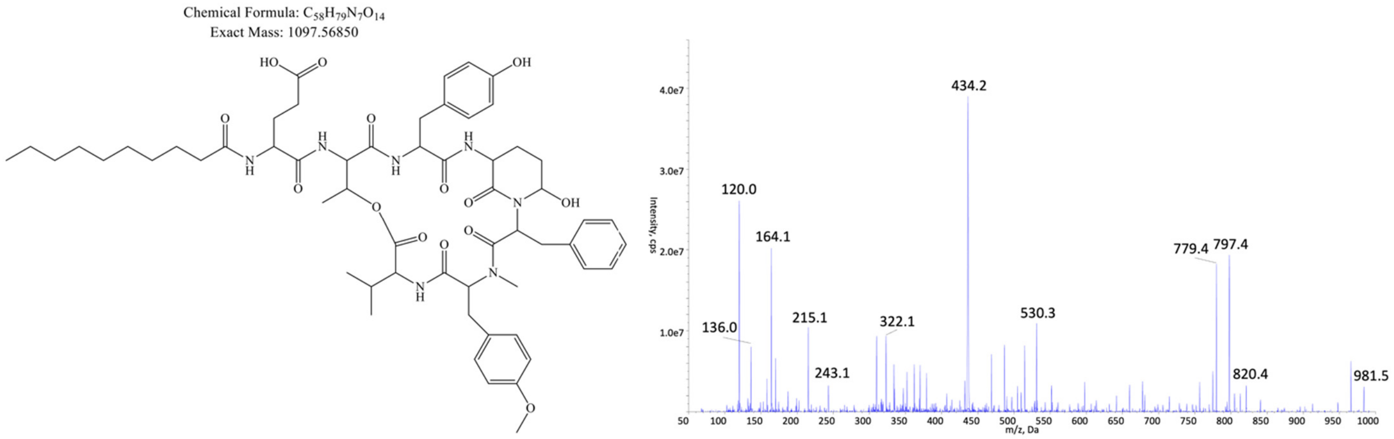


Figure S87. Structure and enhanced product ion mass spectrum of the cyanopeptolin CP 1097.

Table S3. Diagnostic ions for cyanopeptolins produced by *Nostoc edaphicum* CCNP1411.

| Group | Name | (*m/z*) $[M+H]^+$ | $[M+H-H_2O]^+$ | $[M+H-H_2O-CO]^+$ | $[M+H-X^6-H_2O]^+$ | $[M+H-SC]^+$ | $[M+2H-SC-H_2O]^+$ | $[M+2H-SC-2H_2O]^+$ | $[M+H-(Ahp+X^4)-H_2O]^+$ | $[X2+Ahp+X^4+X^5+X^6+H]^+$ | $[SC+Thr+X^2+H-H_2O]^+$ | $[Ahp+X^4+X^5+H-H_2O]^+$ | $[Ahp+X^4+H-H_2O]^+$ | $[X^4+X^5+2H-H_2O]^+$ | $[Ahp+X^4+H-CO-H_2O]^+$ |
|---|---|---|---|---|---|---|---|---|---|---|---|---|---|---|---|
| CP-Arg | CP 777 | 778.43 | 760.4 | 732.4 | 661.3 | | | | 500.3 | 677.4 | | 404.2 | 243.1 | 292.1 | 215.1 |
| CP-Arg | CP 807 | 808.44 | 790.4 | 762.4 | 691.4 | | | | 530.3 | 707.4 | | 434.2 | 243.1 | 322.1 | 215.1 |
| CP-Arg | CP 892 | 893.45 | 875.4 | 847.4 | 776.4 | 778.4 | 761.4 | 743.4 | 615.3 | 677.4 | 354.2 | 404.2 | 243.1 | 292.1 | 215.1 |
| CP-Arg | CP 922 | 923.46 | 905.5 | 877.5 | 806.4 | 808.4 | 791.4 | 773.4 | 645.3 | 707.4 | 354.2 | 434.2 | 243.1 | 322.1 | 215.1 |
| CP-Arg | CP 934 | 935.46 | 917.5 | 889.5 | 818.4 | 777.4 | 760.4 | 742.4 | 657.3 | 677.4 | 397.2 | 404.2 | 243.1 | 292.1 | 215.1 |
| CP-Arg | CP 944 | 945.51 | 927.5 | 899.5 | 828.4 | 759.4 | 742.4 | 724.4 | 700.4 | 660.4 | 425.2 | 387.2 | 210.1 | 274.1 | 182.1 |
| CP-Arg | CP 950 | 951.46 | 933.4 | 905.5 | 834.4 | 793.4 | 776.4 | 758.4 | 673.3 | 693.4 | 397.2 | 420.2 | 243.1 | 308.1 | 215.1 |
| CP-Arg | CP 962 | 963.49 | 945.5 | 917.5 | 846.4 | 777.4 | 760.4 | 742.4 | 685.4 | 677.4 | 425.2 | 404.2 | 243.1 | 292.1 | 215.1 |
| CP-Arg | CP 964 | 965.47 | 947.5 | 919.5 | 848.4 | 807.4 | 790.4 | 772.4 | 687.3 | 707.4 | 397.2 | 434.2 | 243.1 | 322.1 | 215.1 |
| CP-Arg | CP 976 | 977.51 | 959.5 | 931.5 | 846.4 | 791.4 | 774.4 | 756.4 | 699.4 | 691.4 | 425.2 | 404.2 | 243.1 | 292.1 | 215.1 |
| CP-Arg | CP 978 | 979.49 | 961.5 | 933.5 | 862.4 | 793.4 | 776.4 | 758.4 | 701.4 | 693.4 | 425.2 | 420.2 | 243.1 | 308.1 | 215.1 |
| CP-Arg | CP 990 | 991.52 | 973.5 | 945.5 | 874.4 | 777.4 | 760.4 | 742.4 | 713.4 | 677.4 | 453.2 | 404.2 | 243.1 | 292.1 | 215.1 |
| CP-Arg | CP 992 | 993.50 | 975.5 | 947.5 | 876.4 | 807.4 | 790.4 | 772.4 | 715.4 | 707.4 | 425.2 | 434.2 | 243.1 | 322.1 | 215.1 |
| CP-Arg | CP 992b | 993.50 | 975.5 | 947.5 | 862.4 | 807.4 | 790.4 | 772.4 | 715.4 | 707.4 | 425.2 | 420.2 | 243.1 | 308.1 | 215.1 |
| CP-Arg | CP 992c | 993.50 | 975.5 | 947.5 | 876.4 | 793.4 | 776.4 | 758.4 | 715.4 | 693.4 | 439.2 | 420.2 | 243.1 | 308.1 | 215.1 |
| CP-Arg | CP 1006 | 1007.52 | 989.5 | 961.5 | 890.4 | 793.4 | 776.4 | 758.4 | 729.4 | 693.4 | 453.2 | 420.2 | 243.1 | 308.1 | 215.1 |
| CP-Arg | CP 1008 | 1009.40 | 991.5 | 963.5 | 892.4 | 823.4 | 806.4 | 788.4 | 731.4 | 723.4 | 425.2 | 450.2 | 243.1 | 338.1 | 215.1 |
| CP-Arg | CP 1018 | 1019.56 | | 973.6 | 902.5 | 777.4 | 760.4 | 742.4 | 741.4 | 677.4 | 481.3 | 404.2 | 243.1 | 292.1 | 215.1 |
| CP-Arg | CP 1020b | 1021.54 | | 975.5 | 904.5 | 807.4 | 790.4 | 772.4 | 743.4 | 707.4 | 453.2 | 434.2 | 243.1 | 322.1 | 215.1 |
| CP-Arg | CP 1020 | 1021.54 | | 975.5 | 904.5 | 793.4 | 776.4 | 758.4 | 743.4 | 693.4 | 467.3 | 420.2 | 243.1 | 308.1 | 215.1 |
| CP-Arg | CP 1034 | 1035.55 | | 989.5 | 918.5 | 793.4 | 776.4 | 758.4 | 757.4 | 693.4 | 481.3 | 420.2 | 243.1 | 308.1 | 215.1 |
| CP-Arg | CP 1036 | 1037.53 | | 991.5 | 920.5 | 823.4 | 806.4 | 788.4 | 759.4 | 723.4 | 453.2 | 450.2 | 243.1 | 338.1 | 215.1 |
| CP-Arg | CP 1046 | 1047.59 | | | 930.5 | 777.4 | 760.4 | 742.4 | 769.5 | 677.4 | 509.3 | 404.2 | 243.1 | 292.1 | 215.1 |
| CP-Arg | CP 1048 | 1049.57 | | | 932.5 | 807.4 | 790.4 | 772.4 | 771.4 | 707.4 | 481.3 | 434.2 | 243.1 | 322.1 | 215.1 |
| CP-Arg | CP 1076 | 1077.60 | | | 960.5 | 807.4 | 790.4 | 772.4 | 799.5 | 707.4 | 509.3 | 434.2 | 243.1 | 322.1 | 215.1 |

| Group | Name | ($m/z$) $[M+H]^+$ | $[M+H-H_2O]^+$ | $[M+H-H_2O-CO]^+$ | $[M+H-X^6-H_2O]^+$ | $[M+H-SC]^+$ | $[M+2H-SC-H_2O]^+$ | $[M+2H-SC-2H_2O]^+$ | $[M+H-(Ahp+X^4)-H_2O]^+$ | $[X2+Ahp+X^4+X^5+X^6+H]^+$ | $[SC+Thr+X^2+H-H_2O]^+$ | $[Ahp+X^4+X^5+H-H_2O]^+$ | $[Ahp+X^4+H-H_2O]^+$ | $[X^4+X^5+2H-H_2O]^+$ | $[Ahp+X^4+H-CO-H_2O]^+$ |
|---|---|---|---|---|---|---|---|---|---|---|---|---|---|---|---|
| CP-Arg | CP 777 | 778.43 | 760.4 | 732.4 | 661.3 | | | | 500.3 | 677.4 | | 404.2 | 243.1 | 292.1 | 215.1 |
| CP-Arg | CP 807 | 808.44 | 790.4 | 762.4 | 691.4 | | | | 530.3 | 707.4 | | 434.2 | 243.1 | 322.1 | 215.1 |
| CP-Arg | CP 892 | 893.45 | 875.4 | 847.4 | 776.4 | 778.4 | 761.4 | 743.4 | 615.3 | 677.4 | 354.2 | 404.2 | 243.1 | 292.1 | 215.1 |
| CP-Arg | CP 922 | 923.46 | 905.5 | 877.5 | 806.4 | 808.4 | 791.4 | 773.4 | 645.3 | 707.4 | 354.2 | 434.2 | 243.1 | 322.1 | 215.1 |
| CP-Arg | CP 934 | 935.46 | 917.5 | 889.5 | 818.4 | 777.4 | 760.4 | 742.4 | 657.3 | 677.4 | 397.2 | 404.2 | 243.1 | 292.1 | 215.1 |
| CP-Arg | CP 944 | 945.51 | 927.5 | 899.5 | 828.4 | 759.4 | 742.4 | 724.4 | 700.4 | 660.4 | 425.2 | 387.2 | 210.1 | 274.1 | 182.1 |
| CP-Arg | CP 950 | 951.46 | 933.4 | 905.5 | 834.4 | 793.4 | 776.4 | 758.4 | 673.3 | 693.4 | 397.2 | 420.2 | 243.1 | 308.1 | 215.1 |
| CP-Arg | CP 962 | 963.49 | 945.5 | 917.5 | 846.4 | 777.4 | 760.4 | 742.4 | 685.4 | 677.4 | 425.2 | 404.2 | 243.1 | 292.1 | 215.1 |
| CP-Arg | CP 964 | 965.47 | 947.5 | 919.5 | 848.4 | 807.4 | 790.4 | 772.4 | 687.3 | 707.4 | 397.2 | 434.2 | 243.1 | 322.1 | 215.1 |
| CP-Arg | CP 976 | 977.51 | 959.5 | 931.5 | 846.4 | 791.4 | 774.4 | 756.4 | 699.4 | 691.4 | 425.2 | 404.2 | 243.1 | 292.1 | 215.1 |
| CP-Arg | CP 978 | 979.49 | 961.5 | 933.5 | 862.4 | 793.4 | 776.4 | 758.4 | 701.4 | 693.4 | 425.2 | 420.2 | 243.1 | 308.1 | 215.1 |
| CP-Arg | CP 990 | 991.52 | 973.5 | 945.5 | 874.4 | 777.4 | 760.4 | 742.4 | 713.4 | 677.4 | 453.2 | 404.2 | 243.1 | 292.1 | 215.1 |
| CP-Arg | CP 992 | 993.50 | 975.5 | 947.5 | 876.4 | 807.4 | 790.4 | 772.4 | 715.4 | 707.4 | 425.2 | 434.2 | 243.1 | 322.1 | 215.1 |
| CP-Arg | CP 992b | 993.50 | 975.5 | 947.5 | 862.4 | 807.4 | 790.4 | 772.4 | 715.4 | 707.4 | 425.2 | 420.2 | 243.1 | 308.1 | 215.1 |
| CP-Arg | CP 992c | 993.50 | 975.5 | 947.5 | 876.4 | 793.4 | 776.4 | 758.4 | 715.4 | 693.4 | 439.2 | 420.2 | 243.1 | 308.1 | 215.1 |
| CP-Arg | CP 1006 | 1007.52 | 989.5 | 961.5 | 890.4 | 793.4 | 776.4 | 758.4 | 729.4 | 693.4 | 453.2 | 420.2 | 243.1 | 308.1 | 215.1 |
| CP-Arg | CP 1008 | 1009.40 | 991.5 | 963.5 | 892.4 | 823.4 | 806.4 | 788.4 | 731.4 | 723.4 | 425.2 | 450.2 | 243.1 | 338.1 | 215.1 |
| CP-Arg | CP 1018 | 1019.56 | | 973.6 | 902.5 | 777.4 | 760.4 | 742.4 | 741.4 | 677.4 | 481.3 | 404.2 | 243.1 | 292.1 | 215.1 |
| CP-Arg | CP 1020b | 1021.54 | | 975.5 | 904.5 | 807.4 | 790.4 | 772.4 | 743.4 | 707.4 | 453.2 | 434.2 | 243.1 | 322.1 | 215.1 |
| CP-Arg | CP 1020 | 1021.54 | | 975.5 | 904.5 | 793.4 | 776.4 | 758.4 | 743.4 | 693.4 | 467.3 | 420.2 | 243.1 | 308.1 | 215.1 |
| CP-Arg | CP 1034 | 1035.55 | | 989.5 | 918.5 | 793.4 | 776.4 | 758.4 | 757.4 | 693.4 | 481.3 | 420.2 | 243.1 | 308.1 | 215.1 |
| CP-Arg | CP 1036 | 1037.53 | | 991.5 | 920.5 | 823.4 | 806.4 | 788.4 | 759.4 | 723.4 | 453.2 | 450.2 | 243.1 | 338.1 | 215.1 |
| CP-Arg | CP 1046 | 1047.59 | | | 930.5 | 777.4 | 760.4 | 742.4 | 769.5 | 677.4 | 509.3 | 404.2 | 243.1 | 292.1 | 215.1 |
| CP-Arg | CP 1048 | 1049.57 | | | 932.5 | 807.4 | 790.4 | 772.4 | 771.4 | 707.4 | 481.3 | 434.2 | 243.1 | 322.1 | 215.1 |
| CP-Arg | CP 1076 | 1077.60 | | | 960.5 | 807.4 | 790.4 | 772.4 | 799.5 | 707.4 | 509.3 | 434.2 | 243.1 | 322.1 | 215.1 |

| Group | Name | (*m/z*) $[M+H]^+$ | $[M+H-H_2O]^+$ | $[M+H-H_2O-CO]^+$ | $[M+H-X^6-H_2O]^+$ | $[M+2H-SC-H_2O]^+$ | $[M+2H-SC-2H_2O]^+$ | $[M+H-(Ahp+X^4)-H_2O]^+$ | $[X^2+Ahp+X^4+X^5+X^6+H]^+$ | $[SC+Thr+X^2+H-H2O]^+$ | $[Ahp+X^4+X^5+H-H_2O]^+$ | $[Ahp+X^4+H-H_2O]^+$ | $[X^4+X^5+2H-H_2O]^+$ | $[Ahp+X^4+H-CO-H_2O]^+$ |
|---|---|---|---|---|---|---|---|---|---|---|---|---|---|---|
| CP-Leu | CP 891 | 874.44 | 856.4 | 846.4 | 775.4 | 717.4 | 699.4 | 614.3 | 634.4 | 354.2 | 404.2 | 243.1 | 292.1 | 215.1 |
| CP-Leu | CP 907 | 890.43 | 872.4 | 862.4 | 791.4 | 733.4 | 715.4 | 630.3 | 650.4 | 354.2 | 420.2 | 243.1 | 308.1 | 215.1 |
| CP-Leu | CP 919 | 902.47 | 884.5 | 874.5 | 803.4 | 717.4 | 699.4 | 642.4 | 634.4 | 382.2 | 404.2 | 243.1 | 292.1 | 215.1 |
| CP-Leu | CP 921 | 904.45 | 886.4 | 876.5 | 805.4 | 747.4 | 729.4 | 644.3 | 664.4 | 354.2 | 434.2 | 243.1 | 322.1 | 215.1 |
| CP-Leu | CP 933 | 916.48 | 898.5 | 888.5 | 817.4 | 717.4 | 699.4 | 656.4 | 634.4 | 396.2 | 404.2 | 243.1 | 292.1 | 215.1 |
| CP-Leu | CP 935 | 918.46 | 900.5 | 890.5 | 819.4 | 733.4 | 715.4 | 658.3 | 650.4 | 382.2 | 420.2 | 243.1 | 308.1 | 215.1 |
| CP-Leu | CP 947 | 930.50 | 912.5 | 902.5 | 831.4 | 717.4 | 699.4 | 670.4 | 634.4 | 410.2 | 404.2 | 243.1 | 292.1 | 215.1 |
| CP-Leu | CP 949 | 932.48 | 914.5 | 904.5 | 833.4 | 747.4 | 729.4 | 672.4 | 664.4 | 382.2 | 434.2 | 243.1 | 322.1 | 215.1 |
| CP-Leu | CP 963b | 946.49 | 928.5 | 918.5 | 847.4 | 747.4 | 729.4 | 686.4 | 664.4 | 396.2 | 434.2 | 243.1 | 322.1 | 215.1 |
| CP-Leu | CP 963 | 946.49 | 928.5 | 918.5 | 847.4 | 733.4 | 715.4 | 686.4 | 650.4 | 410.2 | 420.2 | 243.1 | 308.1 | 215.1 |
| CP-Leu | CP 965b | 948.47 | 930.5 | 920.5 | 849.4 | 763.4 | 745.4 | 688.4 | 680.4 | 382.2 | 450.2 | 243.1 | 338.1 | 215.1 |
| CP-Leu | CP 975 | 958.53 | 940.5 | 930.5 | 859.5 | 717.4 | 699.4 | 698.4 | 634.4 | 438.3 | 404.2 | 243.1 | 292.1 | 215.1 |
| CP-Leu | CP 977 | 960.51 | 942.5 | 932.5 | 861.4 | 747.4 | 729.4 | 700.4 | 664.4 | 410.2 | 434.2 | 243.1 | 322.1 | 215.1 |
| CP-Leu | CP 991 | 974.52 | 956.5 | 946.5 | 875.5 | 733.4 | 715.4 | 714.4 | 650.4 | 438.3 | 420.2 | 243.1 | 308.1 | 215.1 |
| CP-Leu | CP 1005 | 988.54 | 970.5 | 960.5 | 889.5 | 747.4 | 729.4 | 728.4 | 664.4 | 438.3 | 434.2 | 243.1 | 322.1 | 215.1 |
| CP-MeLeu | CP 933b | 916.48 | 898.5 | 888.5 | 817.4 | 731.4 | 713.4 | 656.4 | 648.4 | 396.2 | 404.2 | 243.1 | 292.1 | 215.1 |
| CP-MeLeu | CP 949b | 932.48 | 914.5 | 904.5 | 833.4 | 747.4 | 729.4 | 672.4 | 664.4 | 396.2 | 420.2 | 243.1 | 308.1 | 215.1 |
| CP-MePhe | CP 939b | 922.44 | 904.4 | 894.4 | 823.4 | 765.4 | 747.4 | 662.3 | 682.4 | 402.2 | 404.2 | 243.1 | 292.1 | 215.1 |
| CP-MePhe | CP 967b | 950.47 | 932.5 | 922.5 | 851.4 | 765.4 | 747.4 | 690.4 | 682.4 | 430.2 | 404.2 | 243.1 | 292.1 | 215.1 |
| CP-MePhe | CP 1011c | 994.49 | 976.5 | 966.5 | 895.4 | 781.4 | 763.4 | 734.4 | 698.4 | 458.2 | 420.2 | 243.1 | 308.1 | 215.1 |
| CP-Met | CP 937 | 920.42 | 902.4 | 892.4 | 821.4 | 735.4 | 717.3 | 660.3 | 652.3 | 400.2 | 404.2 | 243.1 | 292.1 | 215.1 |
| CP-Met | CP 939 | 922.40 | 904.4 | 894.4 | 823.3 | 765.4 | 747.4 | 662.3 | 682.3 | 372.1 | 434.2 | 243.1 | 322.1 | 215.1 |
| CP-Met | CP 953b | 936.42 | 918.4 | 908.4 | 837.3 | 751.3 | 733.3 | 676.3 | 668.3 | 400.2 | 420.2 | 243.1 | 308.1 | 215.1 |
| CP-Met | CP 995 | 978.46 | 960.5 | 950.5 | 879.4 | 765.4 | 747.4 | 718.3 | 682.3 | 428.2 | 434.2 | 243.1 | 322.1 | 215.1 |
| CP-Met | CP 1023 | 1006.50 | 988.5 | 978.5 | 907.4 | 765.4 | 747.4 | 746.4 | 682.3 | 456.2 | 434.2 | 243.1 | 322.1 | 215.1 |

| | | | | | | | | | | | | | | |
|---|---|---|---|---|---|---|---|---|---|---|---|---|---|---|
| CP-MeTyr | CP 999d | 982.46 | 964.4 | 954.5 | 883.4 | 797.4 | 779.4 | 722.3 | 714.4 | 446.2 | 420.2 | 243.1 | 308.1 | 215.1 |
| CP-MeTyr | CP 1014 | 997.12 | 979.1 | 969.1 | 898.1 | 812.1 | 794.0 | 737.0 | 728.4 | 446.2 | 434.2 | 243.1 | 322.1 | 215.1 |
| CP-Phe | CP 925 | 908.42 | 890.4 | 880.4 | 809.4 | 751.4 | 733.4 | 648.3 | 668.3 | 388.2 | 404.2 | 243.1 | 292.1 | 215.1 |
| CP-Phe | CP 953 | 936.45 | 918.4 | 908.5 | 837.4 | 751.4 | 733.4 | 676.3 | 668.3 | 416.2 | 404.2 | 243.1 | 292.1 | 215.1 |
| CP-Phe | CP 955 | 938.43 | 920.4 | 910.4 | 839.4 | 781.4 | 763.4 | 678.3 | 698.4 | 388.2 | 434.2 | 243.1 | 322.1 | 215.1 |
| CP-Phe | CP 967 | 950.47 | 932.5 | 922.5 | 837.4 | 765.4 | 747.4 | 690.4 | 682.4 | 416.2 | 404.2 | 243.1 | 292.1 | 215.1 |
| CP-Phe | CP 969b | 952.45 | 934.4 | 924.5 | 853.4 | 767.4 | 749.4 | 692.3 | 684.3 | 416.2 | 420.2 | 243.1 | 308.1 | 215.1 |
| CP-Phe | CP 981 | 964.48 | 946.5 | 936.5 | 865.4 | 751.4 | 733.4 | 704.4 | 668.3 | 444.2 | 404.2 | 243.1 | 292.1 | 215.1 |
| CP-Phe | CP 983 | 966.46 | 948.5 | 938.5 | 867.4 | 781.4 | 763.4 | 706.3 | 698.4 | 416.2 | 434.2 | 243.1 | 322.1 | 215.1 |
| CP-Phe | CP 997 | 980.48 | 962.5 | 952.5 | 881.4 | 767.4 | 749.4 | 720.4 | 684.3 | 444.2 | 420.2 | 243.1 | 308.1 | 215.1 |
| CP-Phe | CP 1011 | 994.49 | 976.5 | 966.5 | 895.4 | 781.4 | 763.4 | 734.4 | 698.4 | 444.2 | 434.2 | 243.1 | 322.1 | 215.1 |
| CP-Phe | CP 1025b | 1008.51 | 990.5 | 980.5 | 909.4 | 767.4 | 749.4 | 748.4 | 684.3 | 472.2 | 420.2 | 243.1 | 308.1 | 215.1 |
| CP-Trp | CP 992d | 975.46 | 957.5 | 947.5 | 876.4 | 790.4 | 772.4 | 715.3 | 707.4 | 455.2 | 404.2 | 243.1 | 292.1 | 215.1 |
| CP-Trp | CP 1036c | 1019.49 | | 991.5 | 920.4 | 806.4 | 788.4 | 759.4 | 723.4 | 483.2 | 420.2 | 243.1 | 308.1 | 215.1 |
| CP-Trp | CP 1050 | 1033.50 | | | 934.4 | 820.4 | 802.4 | 773.4 | 737.4 | 483.2 | 434.2 | 243.1 | 322.1 | 215.1 |
| CP-Tyr | CP 929 | 912.41 | 894.4 | 884.4 | 813.3 | 798.4 | 780.4 | 652.3 | 714.4 | 361.1 | 434.2 | 243.1 | 322.1 | 215.1 |
| CP-Tyr | CP 941 | 924.41 | 906.4 | 896.4 | 825.3 | 767.4 | 749.4 | 664.3 | 684.3 | 404.1 | 404.2 | 243.1 | 292.1 | 215.1 |
| CP-Tyr | CP 957 | 940.41 | 922.4 | 912.4 | 841.3 | 783.4 | 765.4 | 680.3 | 700.3 | 404.1 | 420.2 | 243.1 | 308.1 | 215.1 |
| CP-Tyr | CP 965 | 948.47 | 930.5 | 920.5 | 849.4 | 763.4 | 745.4 | 722.3 | 680.4 | 432.2 | 400.2 | 209.1 | 288.1 | 181.1 |
| CP-Tyr | CP 969 | 952.45 | 934.4 | 924.5 | 853.4 | 767.4 | 749.4 | 692.3 | 684.3 | 432.2 | 404.2 | 243.1 | 292.1 | 215.1 |
| CP-Tyr | CP 971 | 954.42 | 936.4 | 926.4 | 855.4 | 797.4 | 779.4 | 694.3 | 714.4 | 404.1 | 434.2 | 243.1 | 322.1 | 215.1 |
| CP-Tyr | CP 983b | 966.46 | 948.5 | 938.5 | 853.4 | 781.4 | 763.4 | 706.3 | 698.4 | 432.2 | 404.2 | 243.1 | 292.1 | 215.1 |
| CP-Tyr | CP 983c | 966.46 | 948.5 | 938.5 | 867.4 | 767.4 | 749.4 | 706.3 | 684.3 | 446.2 | 404.2 | 243.1 | 292.1 | 215.1 |
| CP-Tyr | CP 985 | 968.44 | 950.4 | 940.4 | 869.4 | 783.4 | 765.4 | 708.3 | 700.3 | 432.2 | 420.2 | 243.1 | 308.1 | 215.1 |
| CP-Tyr | CP 993 | 976.50 | 958.5 | 948.5 | 877.4 | 763.4 | 745.4 | 750.4 | 680.4 | 460.2 | 400.2 | 209.1 | 288.1 | 181.1 |
| CP-Tyr | CP 997b | 980.48 | 962.5 | 952.5 | 881.4 | 767.4 | 749.4 | 720.4 | 684.3 | 460.2 | 404.2 | 243.1 | 292.1 | 215.1 |
| CP-Tyr | CP 999 | 982.46 | 964.4 | 954.5 | 883.4 | 797.4 | 779.4 | 722.3 | 714.4 | 432.2 | 434.2 | 243.1 | 322.1 | 215.1 |
| CP-Tyr | CP 999b | 982.46 | 964.4 | 954.5 | 869.4 | 797.4 | 779.4 | 722.3 | 714.4 | 432.2 | 420.2 | 243.1 | 308.1 | 215.1 |
| CP-Tyr | CP 999c | 982.46 | 964.4 | 954.5 | 883.4 | 783.4 | 765.4 | 722.3 | 700.3 | 446.2 | 420.2 | 243.1 | 308.1 | 215.1 |
| CP-Tyr | CP 1011b | 994.49 | 976.5 | 966.5 | 881.4 | 781.4 | 763.4 | 734.4 | 698.4 | 460.2 | 404.2 | 243.1 | 292.1 | 215.1 |
| CP-Tyr | CP 1013b | 996.37 | 978.5 | 968.5 | 883.4 | 811.4 | 793.4 | 736.4 | 728.4 | 432.2 | 434.2 | 243.1 | 322.1 | 215.1 |
| CP-Tyr | CP 1013 | 996.47 | 978.5 | 968.5 | 897.4 | 783.4 | 765.4 | 736.4 | 700.3 | 460.2 | 420.2 | 243.1 | 308.1 | 215.1 |
| CP-Tyr | CP 1013c | 996.47 | 978.5 | 968.5 | 897.4 | 797.4 | 779.4 | 736.4 | 714.4 | 446.2 | 434.2 | 243.1 | 322.1 | 215.1 |

| | | | | | | | | | | | | | | |
|---|---|---|---|---|---|---|---|---|---|---|---|---|---|---|
| CP-Tyr | CP 1016 | 999.01 | 981.0 | 971.0 | 899.9 | 813.9 | 795.9 | 738.9 | 730.3 | 432.2 | 450.2 | 243.1 | 338.1 | 215.1 |
| CP-Tyr | CP 1025 | 1008.51 | 990.5 | 980.5 | 909.4 | 767.4 | 749.4 | 748.4 | 684.3 | 488.2 | 404.2 | 243.1 | 292.1 | 215.1 |
| CP-Tyr | CP 1027 | 1010.49 | 992.5 | 982.5 | 911.4 | 797.4 | 779.4 | 750.4 | 714.4 | 460.2 | 434.2 | 243.1 | 322.1 | 215.1 |
| CP-Tyr | CP 1036b | 1019.49 | | 991.5 | 920.4 | 806.4 | 788.4 | 759.4 | 723.4 | 460.2 | 443.2 | 243.1 | 331.1 | 215.1 |
| CP-Tyr | CP 1041 | 1024.50 | | 996.5 | 925.4 | 783.4 | 765.4 | 764.4 | 700.3 | 488.2 | 420.2 | 243.1 | 308.1 | 215.1 |
| CP-Tyr | CP 1053 | 1036.54 | | | 937.5 | 767.4 | 749.4 | 776.4 | 684.3 | 516.3 | 404.2 | 243.1 | 292.1 | 215.1 |
| CP-Tyr | CP 1055 | 1038.52 | | | 939.5 | 797.4 | 779.4 | 778.4 | 714.4 | 488.2 | 434.2 | 243.1 | 322.1 | 215.1 |
| CP-Tyr | CP 1069 | 1052.53 | | | 953.5 | 783.4 | 765.4 | 792.4 | 700.3 | 516.3 | 420.2 | 243.1 | 308.1 | 215.1 |
| CP-Tyr | CP 1083 | 1066.55 | | | 967.5 | 797.4 | 779.4 | 806.4 | 714.4 | 516.3 | 434.2 | 243.1 | 322.1 | 215.1 |
| CP-Tyr | CP 1097 | 1080.57 | | | 981.5 | 797.4 | 779.4 | 820.4 | 700.3 | 530.3 | 420.2 | 243.1 | 308.1 | 215.1 |

Table S4. NMR Spectroscopic Data for cyanopeptolin CP 941 – AC-Asp-[Thr-Tyr-Ahp-Phe-MePhe-Val].

| Residue | Position | δC | δH (*J* in Hz) | ROESY | HMBC[a] |
|---|---|---|---|---|---|
| Ac | 1 | 169.4 | | | |
| | 2 | 23.1 | 1.84, s | | Ac-1 |
| Asp | 1 | *nd* | | | |
| | 2 | 50.5 | 4.48, m | Thr-NH | |
| | 3a | 37.7 | 2.35, m | | |
| | 3b | | 2.22, m | | |
| | 4 | *nd* | | | |
| | NH | | 8.63, m | | |
| Thr | 1 | 169.3 | | | |
| | 2 | 54.8 | 4.51, d (9.9) | Tyr-NH | Thr-1 |
| | 3 | 72.8 | 5.35, q (6.8) | Tyr-NH | |
| | 4 | 17.8 | 1.11, d (6.6) | | Thr-2, Thr-3 |
| | NH | | 7.68, d (9.6) | Asp-2 | |
| Tyr | 1 | *nd* | | | |
| | 2 | 54.5 | 4.26, m | Asp-NH | |
| | 3a | 35.4 | 3.13, dd (10.2, 3.7) | Tyr-2′/6′ | |
| | 3b | | 2.43[b], m | Tyr-2′/6′ | |
| | 1′ | 128.2 | | | |
| | 2′/6′ | 130.0 | 6.86, d (8.2) | Tyr-3a, Tyr-3b | Tyr-4′ |
| | 3′/5′ | 115.8 | 6.54, d (8.2) | | Tyr-1′ |
| | 4′ | 156.7 | | | |
| | NH | | 8.41, d (8.9) | Thr-2, Thr-3 | |
| Ahp | 1 | *nd* | | | |
| | 2 | 49.2 | 3.63, m | | |
| | 3a | 21.9 | 2.41[b], m | | |
| | 3b | | 1.60, m | | |
| | 4a | 29.7 | 1.69[c], m | Phe-2′/6′ | |
| | 4b | | 1.50, m | Phe-2′/6′ | |
| | 5 | 74.2 | 5.04, brs | | |
| | NH | | 7.05, d (9.2) | Tyr-2 | |
| | OH | | 5.98 | | |
| Phe | 1 | 170.7 | | | |
| | 2 | 50.5 | 4.74, dd (6.9, 4.4) | Phe-2′/6′ | |
| | 3a | 35.7 | 2.84[d], m | Phe-2′/6′ | |
| | 3b | | 1.69[c], m | | |
| | 1′ | 137.2 | | | |
| | 2′/6′ | 129.8 | 6.78, d (7.3) | Phe-2, Phe-3a, | Phe-3, Phe-4′ |
| | 3′/5′ | 128.2 | 7.19, t (7.4) | Ahp-4a, Ahp-4b | Phe-1′ |
| | 4′ | 126.7 | 7.14, t (7.3) | | Phe-2′/6′ |
| MePhe | 1 | *nd* | | | |
| | 2 | 61.0 | 5.00, dd (9.1, 2.2) | MePhe-2′/6′, | |
| | 3a | 34.3 | 3.23, m | Val-NH | |
| | 3b | | 2.83[d], m | MePhe-2′/6′ | |
| | 1′ | 138.3 | | MePhe-2′/6′ | |
| | 2′/6′ | 130.0 | 7.25, d (7.3) | | MePhe-4′, MePhe-3 |
| | 3′/5′ | 129.1 | 7.41, t (7.7) | MePhe-2, | MePhe-1′ |
| | 4′ | 127.1 | 7.31, t (7.4) | MePhe-3a, | MePhe-2′/6′ |
| | $NCH_3$ | 30.9 | 2.79, s | MePhe-3b | MePhe-2, Phe-1 |
| Val | 1 | *nd* | | | |
| | 2 | 56.4 | 4.63, dd (4.9, 4.3) | | |
| | 3 | 31.4 | 2.03, m | | |
| | 4 | 19.7 | 0.86, d (6.8) | | Val-2, Val-3, Val-5 |
| | 5 | 17.8 | 0.73, d (6.8) | | Val-2, Val-3, Val-4 |
| | NH | | 7.39, d (9.6) | MePhe-2 | |

[a] HMBC correlations are given from proton(s) stated to the indicated carbon atom; [b-d] assignments with the same superscript are overlapping; *nd* – resonances not detected

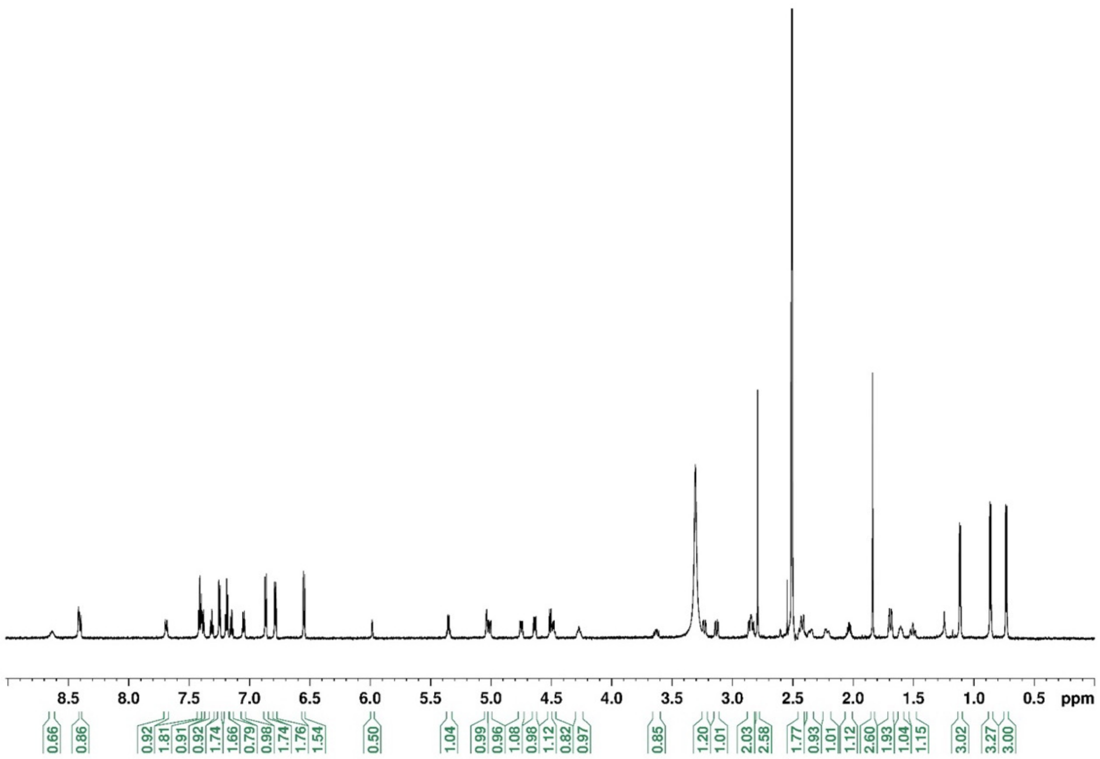


Figure S88. $^1$H NMR spectrum of cyanopeptolin CP 941 in DMSO-$d_6$.

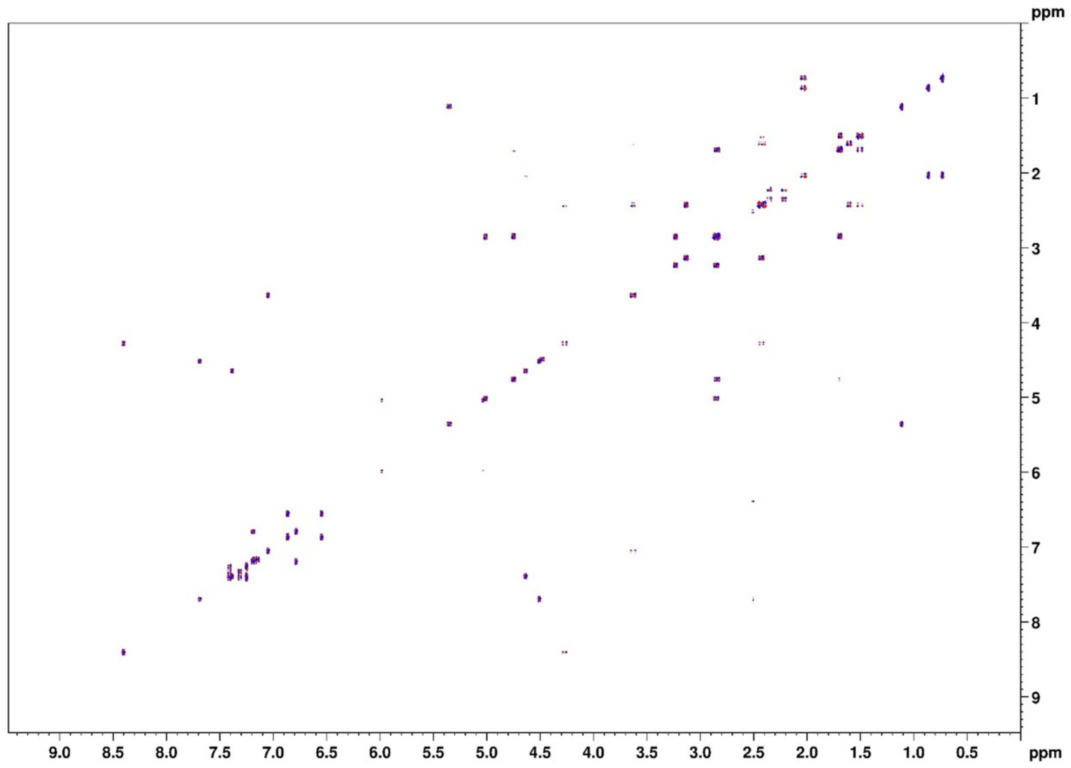


Figure S89. DQF-COSY spectrum of cyanopeptolin CP 941 in DMSO-$d_6$.

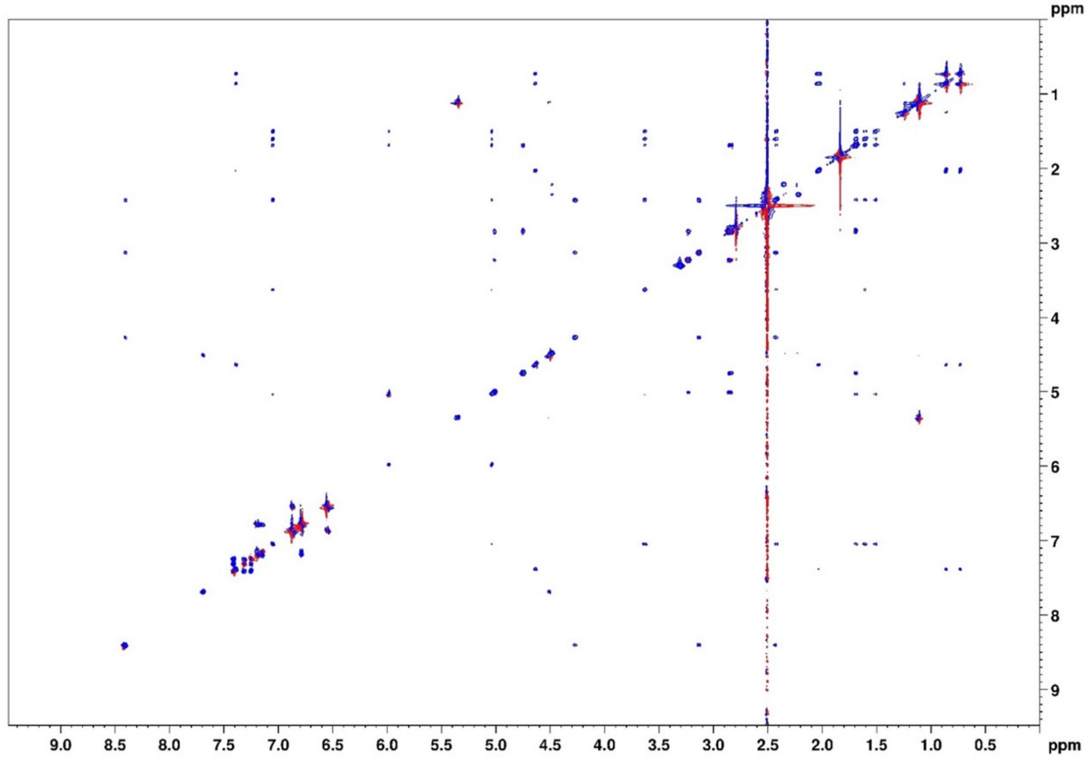


Figure S90. TOCSY spectrum of cyanopeptolin CP 941 in DMSO-d6.

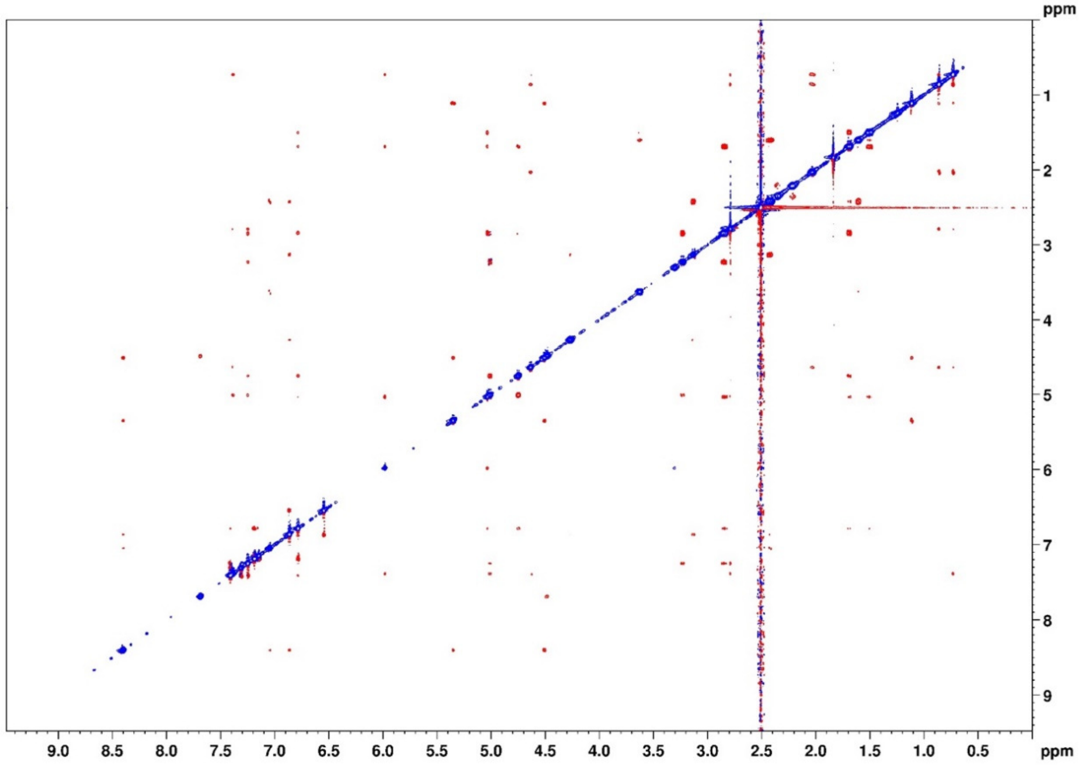


Figure S91. ROESY spectrum of cyanopeptolin CP 941 in DMSO-d6.

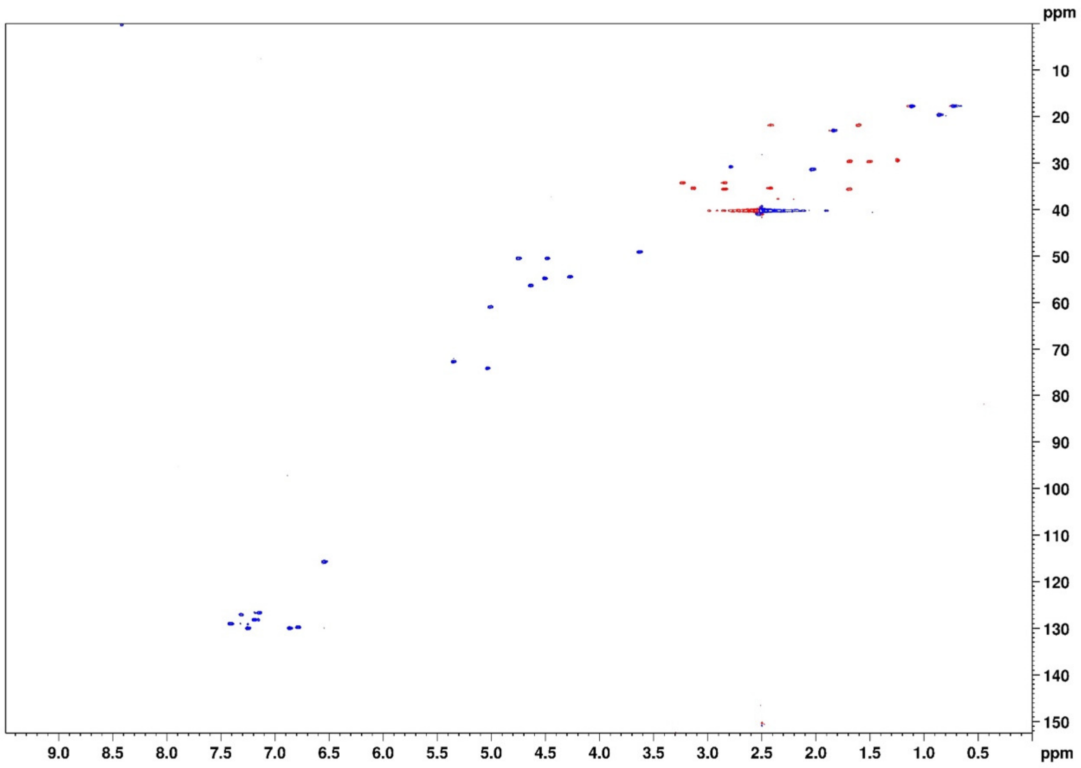


Figure S92. HSQC spectrum of cyanopeptolin CP 941 in DMSO-d6.

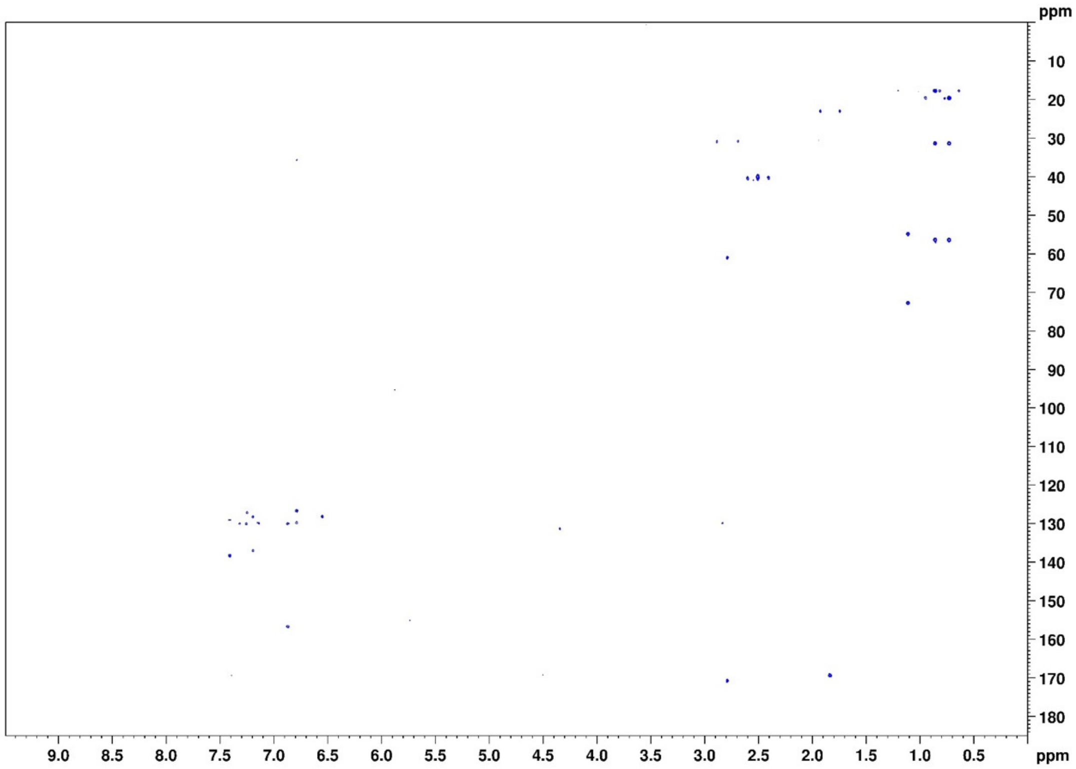


Figure S93. HMBC spectrum of cyanopeptolin CP 941 in DMSO-d6.

Table S5. NMR Spectroscopic Data for cyanopeptolin CP 999 – BA-Asp-[Thr-Tyr-Ahp-Phe-MeTyr(OMe)-Val].

| Residue | Position | $\delta_C$ | $\delta_H$ (*J* in Hz) | ROESY | HMBC[a] |
|---|---|---|---|---|---|
| BA | 1 | 172 | | | |
| | 2 | 37.9 | 2.05, t (7.0) | | BA-1, BA-3, BA-4 |
| | 3 | 19.2 | 1.53, q (7.3) | | BA-1, BA-2, BA-4 |
| | 4 | 14.1 | 0.88, t (7.3) | | BA-2, BA-3 |
| Asp | 1 | *nd* | | | |
| | 2 | 50.6 | 4.47, m | Thr-NH | |
| | 3a | 38.7 | 2.30, m | | |
| | 3b | | 2.12, m | | |
| | 4 | *nd* | | | |
| | NH | | 8.77, m | | |
| Thr | 1 | *nd* | | | |
| | 2 | 54.7 | 4.54, d (9.9) | Tyr-NH | |
| | 3 | 72.8 | 5.35, m | Tyr-NH | |
| | 4 | 17.6 | 1.11, d (6.5) | | Thr-2, Thr-3 |
| | NH | | 7.68, d (8.5) | Asp-2 | |
| Tyr | 1 | *nd* | | | |
| | 2 | 54.7 | 4.22, m | Ahp-NH | |
| | 3a | 35.6 | 3.13, dd (10.4, 3.6) | Tyr-2′/6′ | |
| | 3b | | 2.38, m | Tyr-2′/6′ | |
| | 1′ | 128.0 | | | |
| | 2′/6′ | 129.9 | 6.84, d (8.5) | Tyr-3a, Tyr-3b | Tyr-3, Tyr-4′ |
| | 3′/5′ | 115.9 | 6.53, d (8.0) | | Tyr-1′ |
| | 4′ | 156.9 | | | |
| | NH | | 8.42, d (9.3) | Thr-2, Thr-3 | |
| Ahp | 1 | *nd* | | | |
| | 2 | 49.1 | 3.67, m | | |
| | 3a | 21.9 | 2.44, m | | |
| | 3b | | 1.61, m | | |
| | 4a | 29.7 | 1.71, m | | |
| | 4b | | 1.54, m | Phe-2′/6′ | |
| | 5 | 74.2 | 5.06, brs | Phe-3a | |
| | NH | | 7.03, d (9.3) | Tyr-2 | |
| | OH | | 6.01 | | |
| Phe | 1 | 170.9 | | | |
| | 2 | 50.7 | 4.70, dd (6.8, 4.5) | MeTyr(OMe)-2 | |
| | 3a | 35.8 | 2.87, dd (2.3, 11.8) | Ahp-5, Phe-2′/6′ | Phe-2′/6′ |
| | 3b | | 1.79, dd (10.5, 3.8) | Phe-2′/6′ | |
| | 1′ | 137.2 | | | |
| | 2′/6′ | 129.7 | 6.79, d (7.2) | Phe-3a, Phe-3b, Ahp-4b | Phe-3, Phe-4′ |
| | 3′/5′ | 128.2 | 7.19, t (7.5) | | Phe-1′, Phe-2′/6′ |
| | 4′ | 126.7 | 7.15[b] | | Phe-2′/6′ |
| *N,O*-MeTyr | 1 | 169.5 | | | |
| | 2 | 61.3 | 4.91, dd (8.9, 2.4) | Phe-2, Val-NH | |
| | 3a | 33.3 | 3.18, m | MeTyr(OMe)-2′/6′ | |
| | 3b | | 2.76, m | MeTyr(OMe)-2′/6′ | |
| | 1′ | 129.9 | | | |
| | 2′/6′ | 131.0 | 7.14[b] | MeTyr(OMe)-3a, | MeTyr(OMe)-3, MeTyr(OMe)-4′ |
| | 3′/5′ | 114.5 | 6.97, d (8.8) | MeTyr(OMe)-3b | MeTyr(OMe)-1′ |
| | 4′ | 158.6 | | | |
| | $OCH_3$ | 55.5 | 3.71, s | | MeTyr(OMe)-4′ |
| | $NCH_3$ | 30.8 | 2.77, s | | MeTyr(OMe)-2, Phe-1 |
| Val | 1 | *nd* | | | |
| | 2 | 56.2 | 4.61, dd (5.0, 4.5) | | |
| | 3 | 31.3 | 2.02, m | | |
| | 4 | 19.8 | 0.86, d (6.8) | | Val-2, Val-3, Val-5 |
| | 5 | 17.7 | 0.73, d (6.8) | | Val-2, Val-3, Val-4 |
| | NH | | 7.37, d (9.5) | | MeTyr(OMe)-1 |

[a] HMBC correlations are given from proton(s) stated to the indicated carbon atom; [b] assignments with the same superscript are overlapping; *nd* – resonances not detected

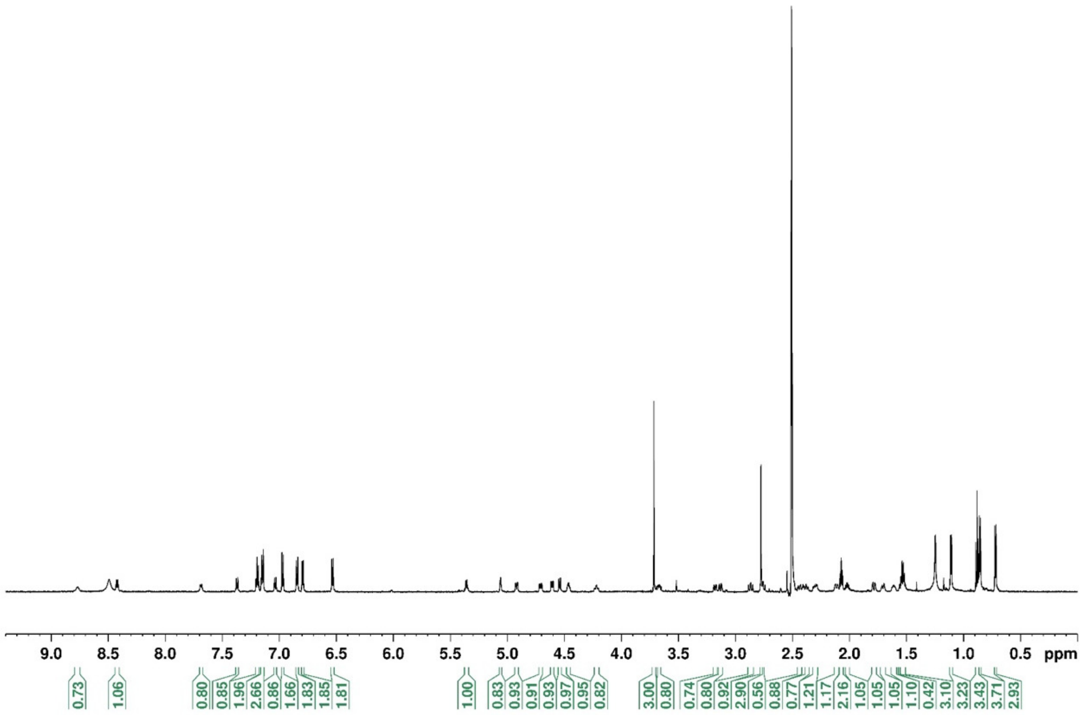


Figure S94. $^{1}$H NMR spectrum of cyanopeptolin CP 999 in DMSO-d6.

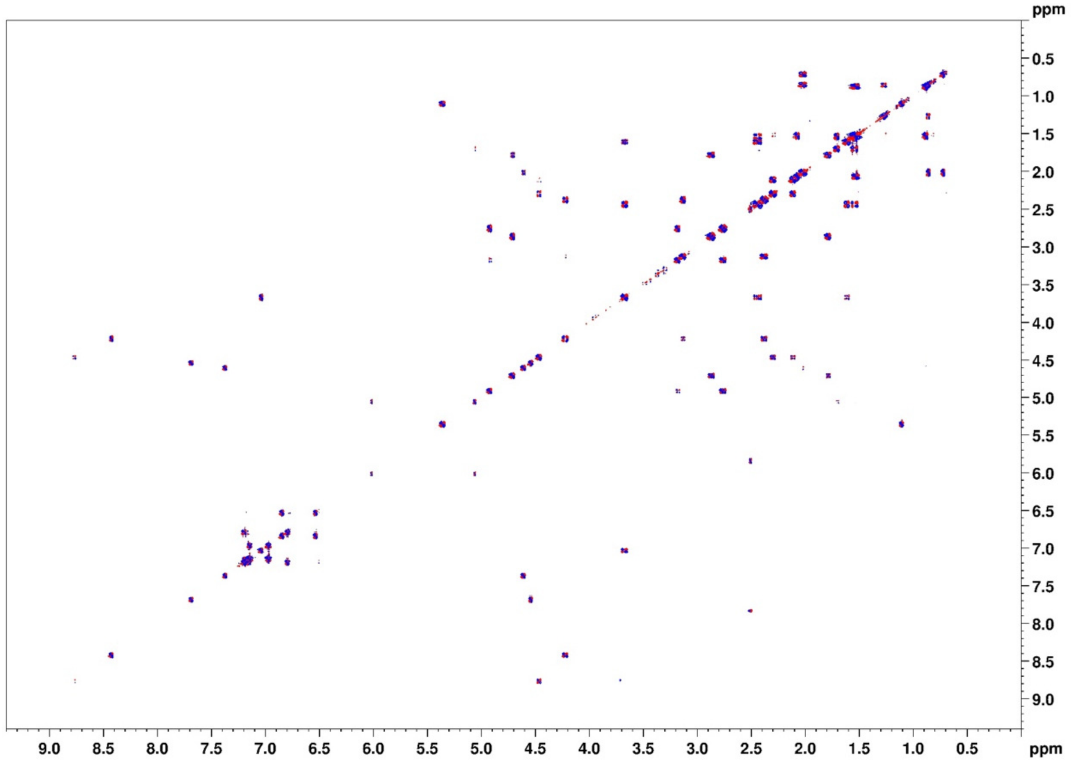


Figure S95. DQF-COSY spectrum of cyanopeptolin CP 999 in DMSO-d6.

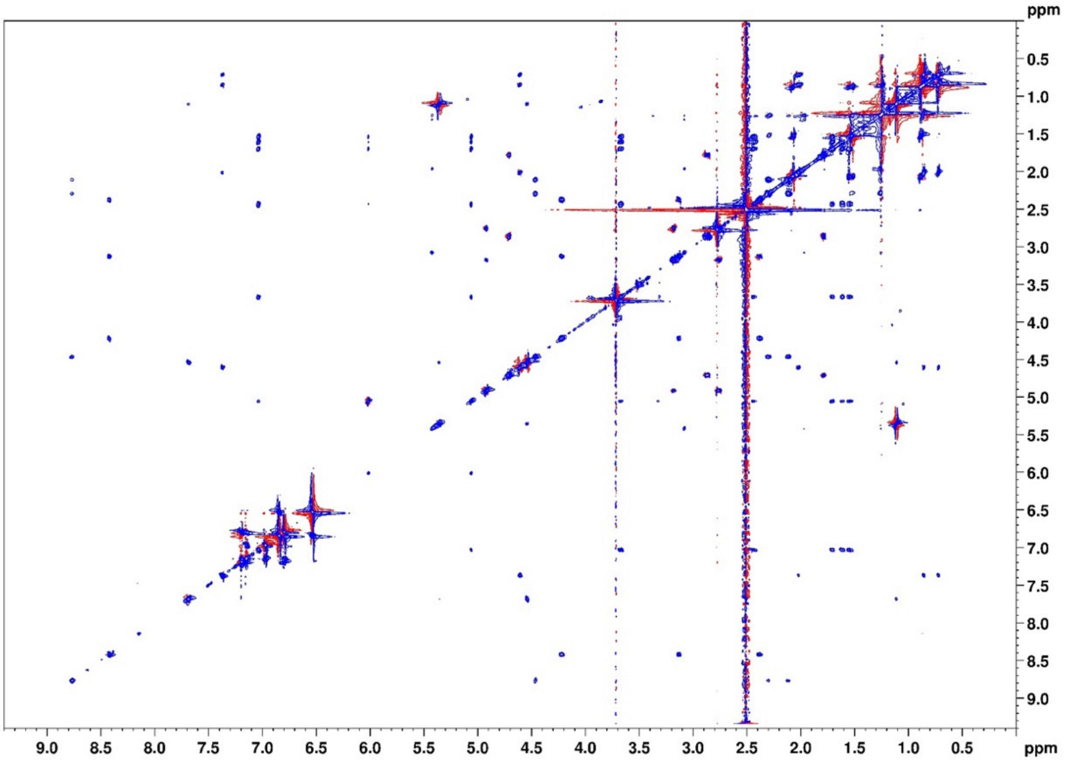


Figure S96. TOCSY spectrum of cyanopeptolin CP 999 in DMSO-d6.

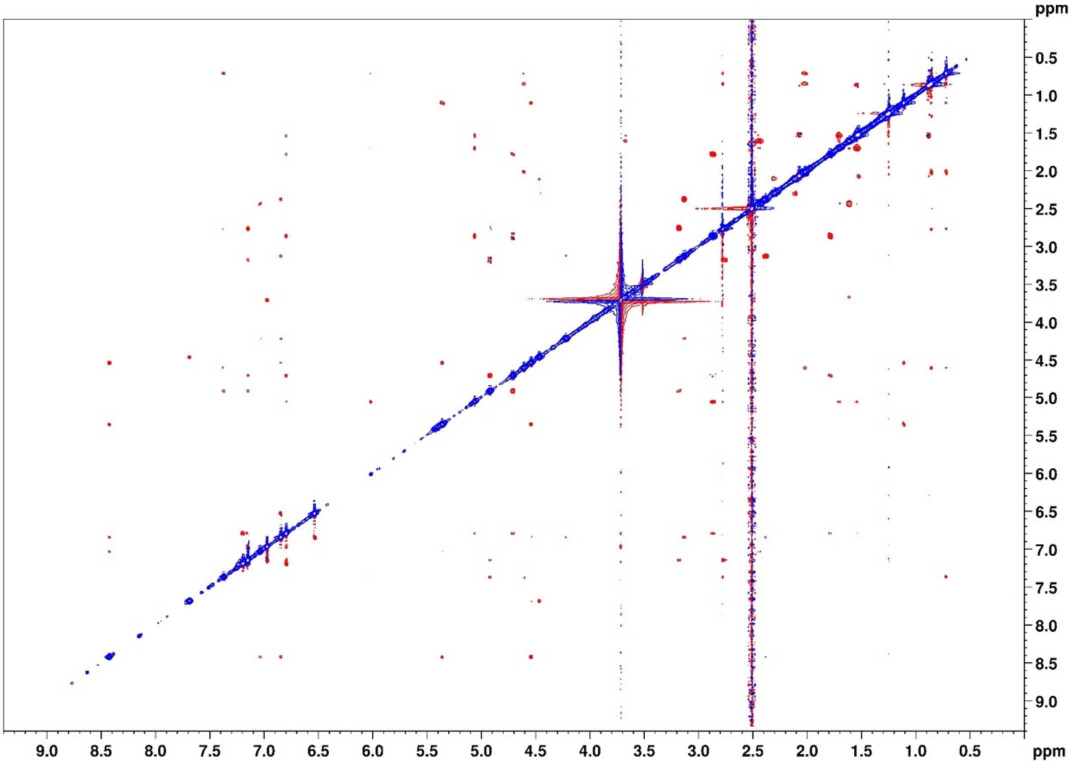


Figure S97. ROESY spectrum of cyanopeptolin CP 999 in DMSO-d6.

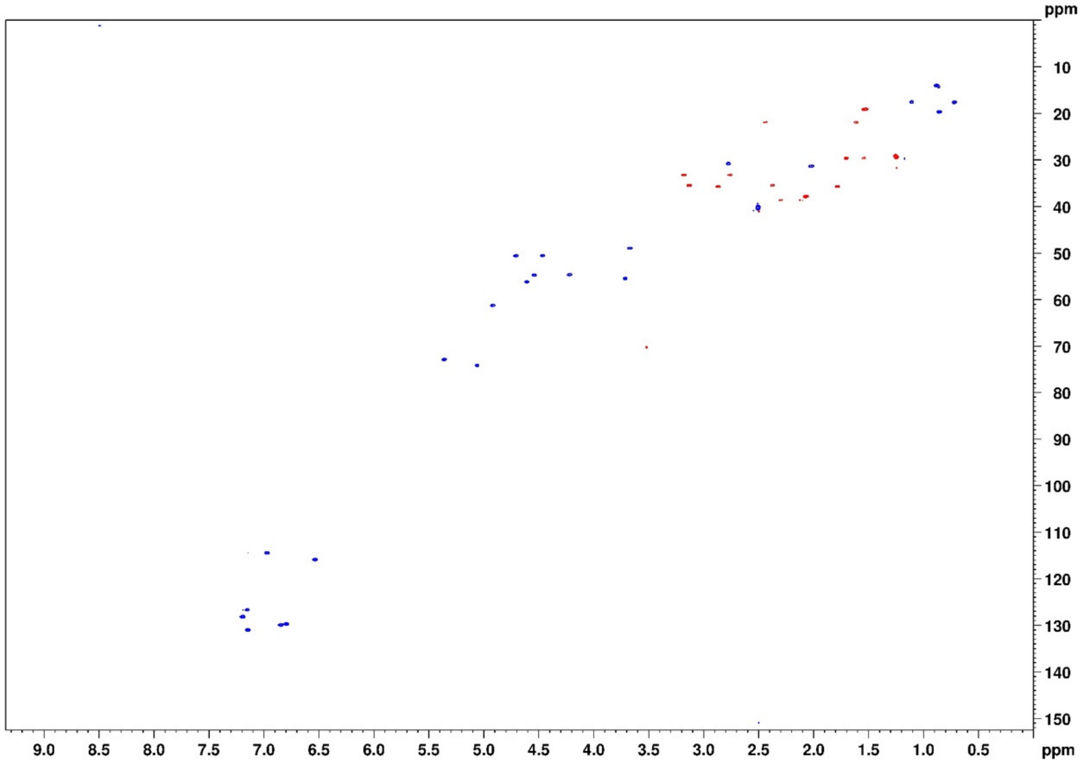


Figure S98. HSQC spectrum of cyanopeptolin CP 999 in DMSO-d6.

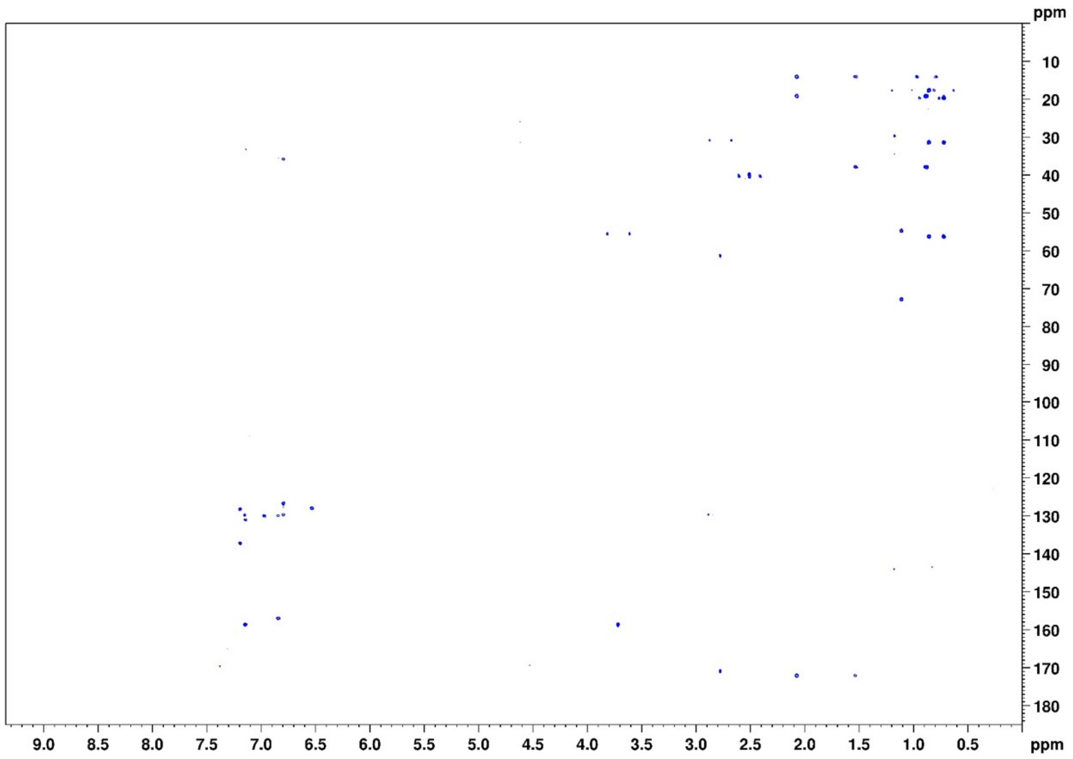


Figure S99. HMBC spectrum of cyanopeptolin CP 999 in DMSO-d6.

Table S6. NMR Spectroscopic Data for cyanopeptolin CP 990 – HA-Asp-[Thr-Arg-Ahp-Phe-MePhe-Val]

| Residue | Position | $\delta_C$ | $\delta_H$ (*J* in Hz) | ROESY | HMBC[a] |
|---|---|---|---|---|---|
| HA | 1 | 172.3 | | | |
| | 2 | 35.5 | 2.09, t (7.3) | | HA-1, HA-3, HA-4 |
| | 3 | 25.5 | 1.50, m | | HA-1, HA-4, HA-5 |
| | 4 | 31.2 | 1.23, m | | |
| | 5 | 22.4 | 1.27, m | | HA-4 |
| | 6 | 14.3 | 0.88[b], t | | HA-4, HA-5 |
| Asp | 1 | *nd* | | | |
| | 2 | 50.8 | 4.52, m | | |
| | 3a | 39.9 | 2.53, m | | Asp-2 |
| | 3b | | 2.11, m | | |
| | 4 | *nd* | | | |
| | NH | | 8.00, d (8.1) | | HA-1 |
| Thr | 1 | 169.9 | | | |
| | 2 | 55.0 | 4.58, d (9.0) | Arg-NH | Thr-1 |
| | 3 | 72.2 | 5.31, q (6.6) | Arg-NH | Val-1 |
| | 4 | 17.9 | 1.14, d (6.6) | | Thr-2, Thr-3 |
| | NH | | 7.23[c], d | | |
| Arg | 1 | 170.3 | | | |
| | 2 | 50.9 | 4.15, m | | |
| | 3a | 26.4 | 1.88, m | | |
| | 3b | | 1.45, m | | |
| | 4a | 24.6 | 1.47, m | | |
| | 4b | | 1.17, m | | |
| | 5a | 39.7 | 2.95, m | | |
| | 5b | | 2.89, m | | |
| | 6 | *nd* | | Thr-2, Thr-3 | Thr-1 |
| | NH | | 8.51, d (9.0) | | |
| Ahp | 1 | *nd* | | | |
| | 2 | 48.9 | 3.65, m | | |
| | 3a | 21.9 | 2.42, m | | |
| | 3b | | 1.55, m | | |
| | 4a | 29.6 | 1.69 | | |
| | 4b | | 1.49, m | Phe-2′/6′ | |
| | 5 | 74.1 | 5.04 | Phe-2′/6′ | |
| | NH | | 6.96, d (9.5) | | Arg-1 |
| | OH | | 6.01 | | |
| Phe | 1 | 170.7 | | | |
| | 2 | 50.6 | 4.74, dd (7.3, 4.4) | Phe-2′/6′, MePhe-2 | Phe-1 |
| | 3a | 35.7 | 2.85, m | Phe-2′/6′ | Phe-2′/6′ |
| | 3b | | 1.69, m | Phe-2′/6′ | Phe-2′/6′ |
| | 1′ | 137.0 | | | |
| | 2′/6′ | 129.8 | 6.78, d (7.3) | Ahp-4b, Ahp-5, Phe-2, Phe-3a, Phe-3b | Phe-3, Phe-4′ |
| | 3′/5′ | 128.2 | 7.18, t (7.4) | | Phe-1′ |
| | 4′ | 126.7 | 7.13, t (7.3) | | Phe-2′/6′ |
| MePhe | 1 | 169.3 | | | |
| | 2 | 60.9 | 5.02 | Phe-2, MePhe-2′/6′, Val-NH | |
| | 3a | 34.4 | 3.23, dd (11.7, 2.7) | MePhe-2′/6′ | |
| | 3b | | 2.88, m | MePhe-2′/6′ | |
| | 1′ | 138.3 | | | |
| | 2′/6′ | 130.1 | 7.24[c], d | MePhe-2, MePhe-3a, MePhe-3b | MePhe-3, MePhe-4′ |
| | 3′/5′ | 129.1 | 7.42[d], t | | MePhe-1′ |
| | 4′ | 127.1 | 7.32, t (7.4) | | MePhe-2′/6′ |
| | $NCH_3$ | 30.9 | 2.80, s | | MePhe-2, Phe-1 |
| Val | 1 | 172.6 | | | |
| | 2 | 56.3 | 4.71, dd (5.0, 4.3) | | Val-1 |
| | 3 | 31.6 | 2.05, m | | |
| | 4 | 19.7 | 0.87[b], d | | Val-2, Val-3, Val-5, |
| | 5 | 17.9 | 0.74, d (6.8) | | Thr-4 |
| | NH | | 7.41[d], d | MePhe-2 | Val-2, Val-3, Val-4 MePhe-1 |

[a] HMBC correlations are given from proton(s) stated to the indicated carbon atom; [b-d] assignments with the same superscript are overlapping; *nd* – resonances not detected

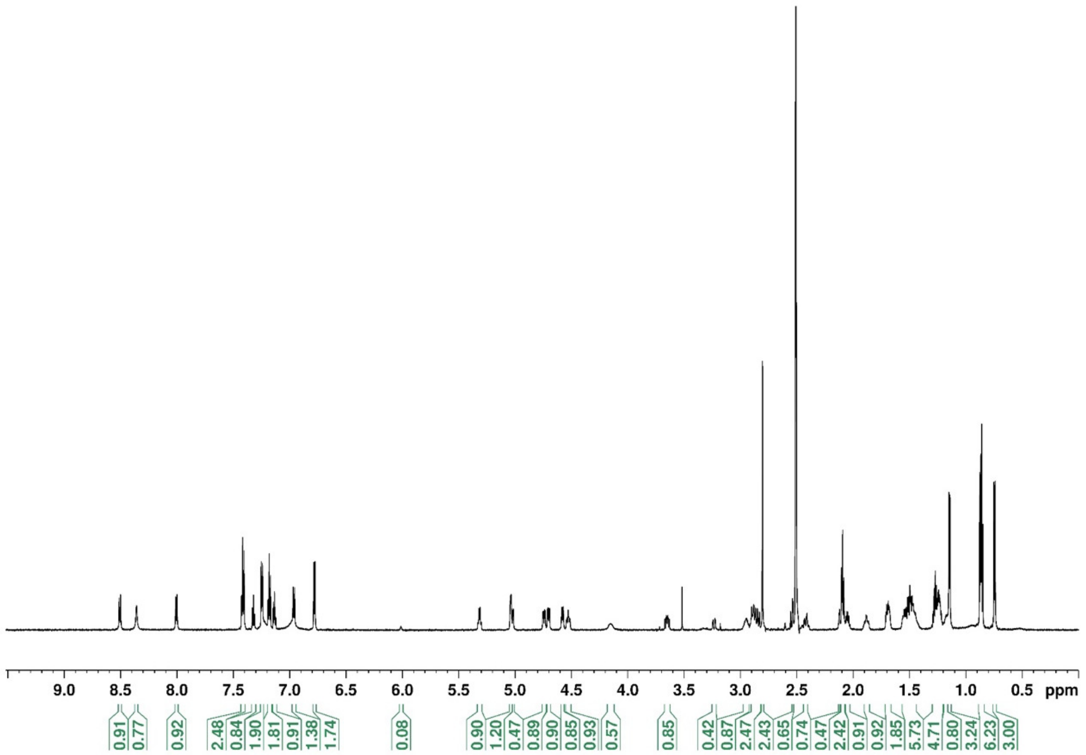


Figure S100. $^{1}$H NMR spectrum of cyanopeptolin CP 990 in DMSO-d6.

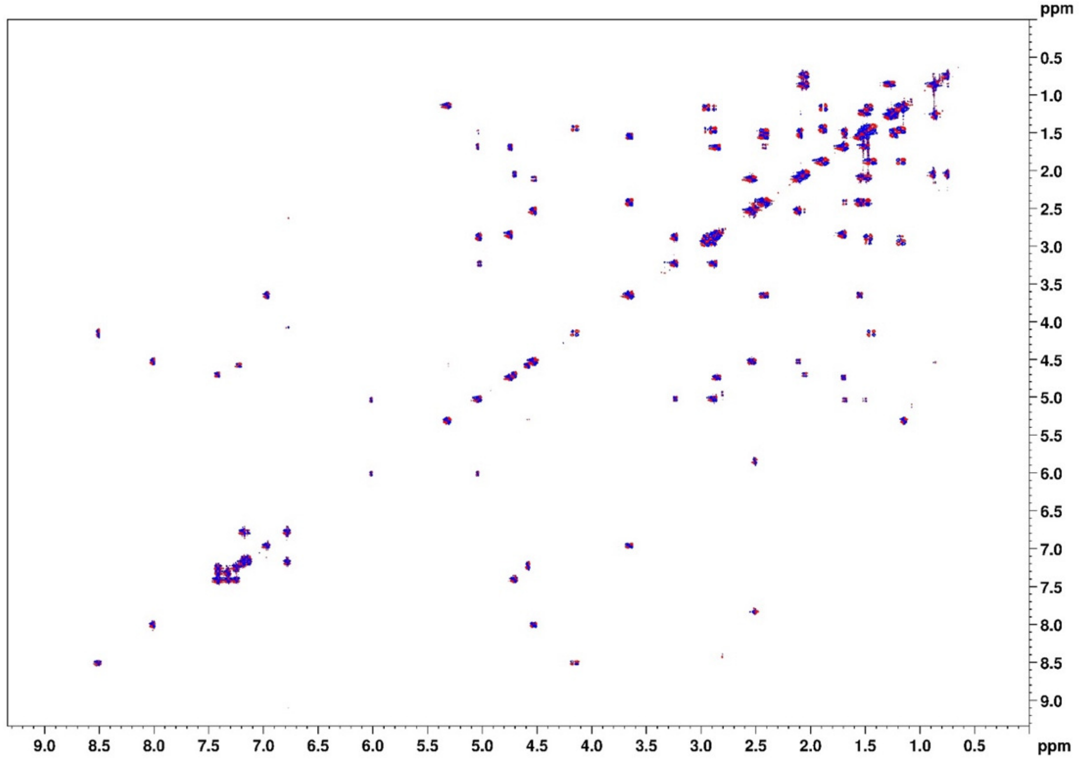


Figure S101. DQF-COSY spectrum of cyanopeptolin CP 990 in DMSO-d6.

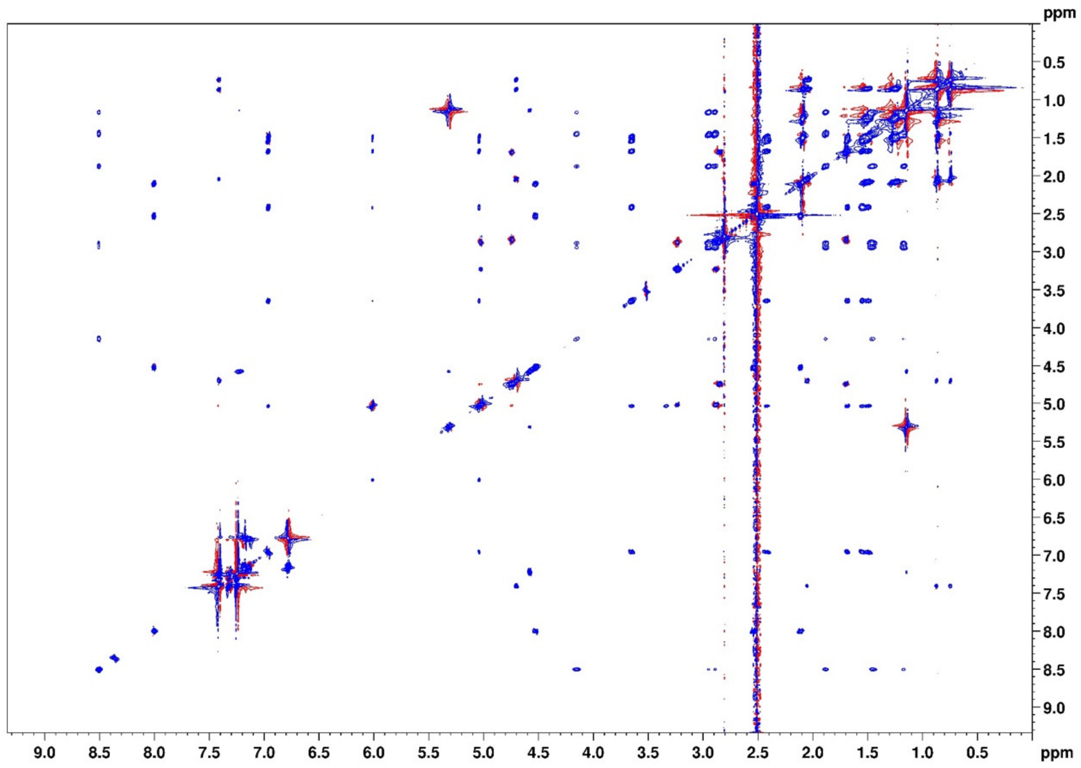


Figure S102. TOCSY spectrum of cyanopeptolin CP 990 in DMSO-d6.

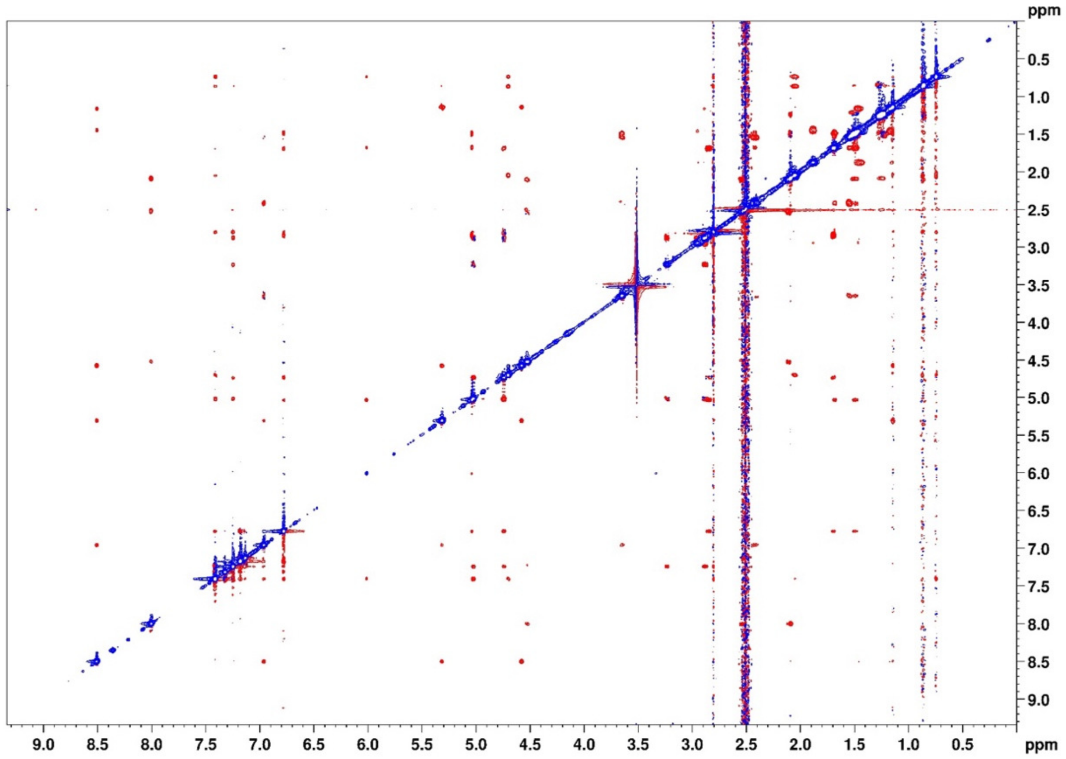


Figure S103. ROESY spectrum of cyanopeptolin CP 990 in DMSO-d6.

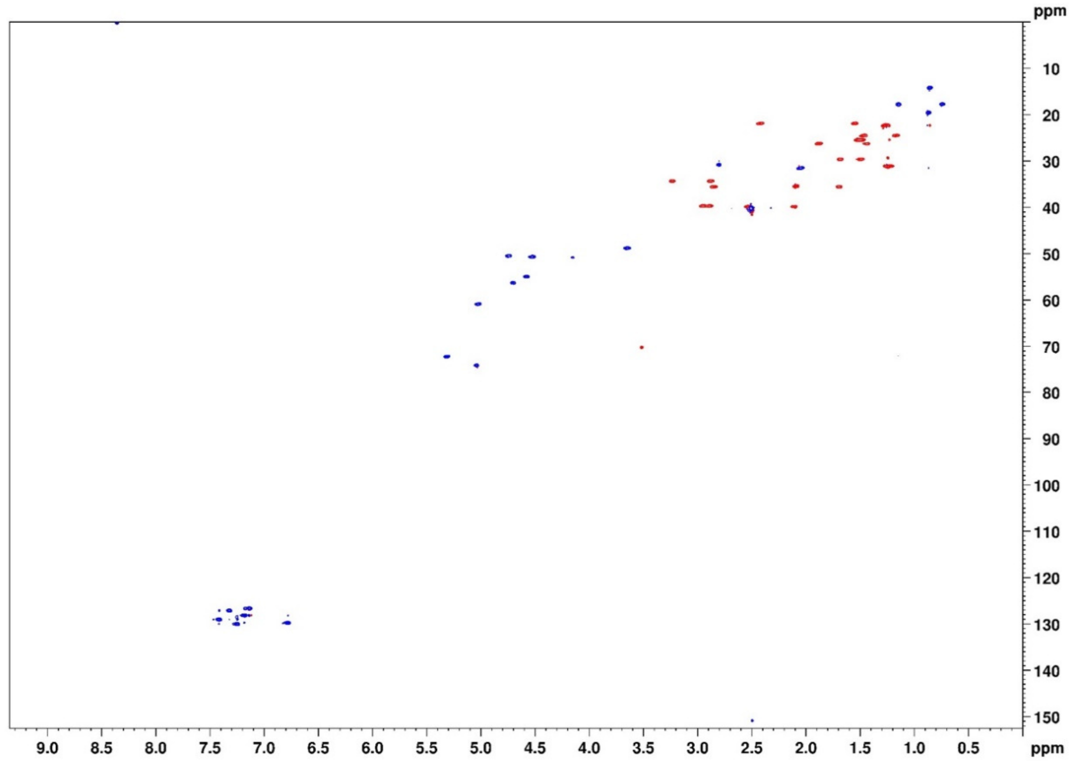


Figure S104. HSQC spectrum of cyanopeptolin CP 990 in DMSO-d6.

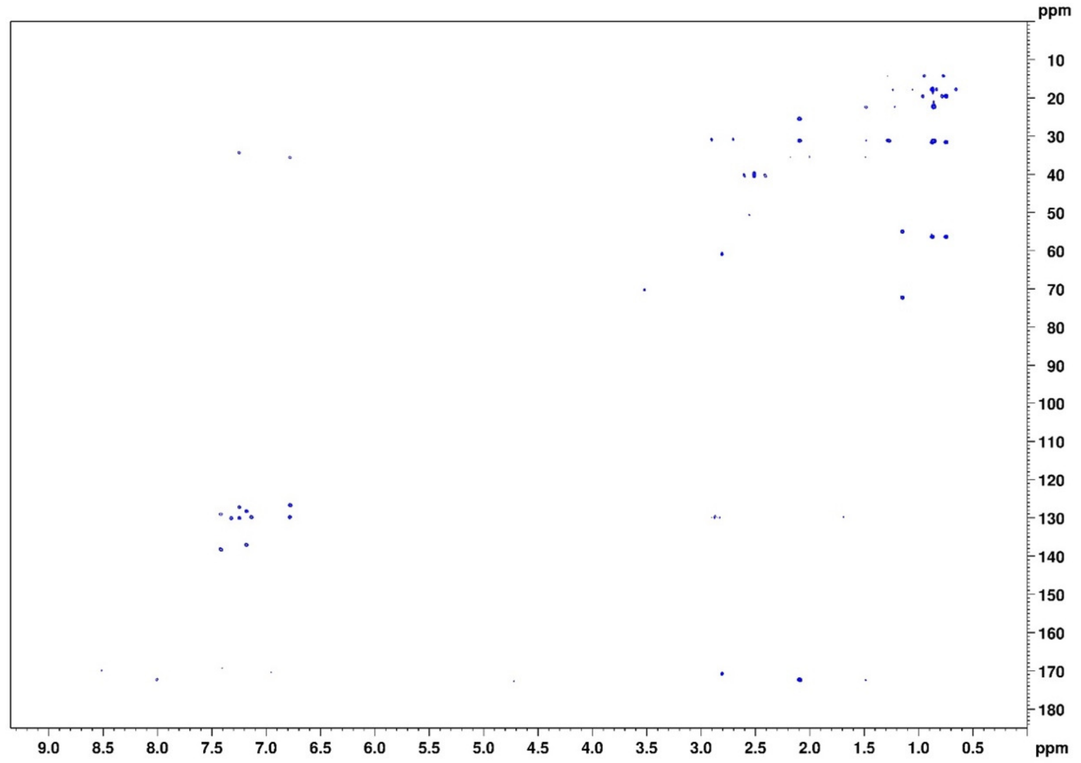


Figure S105. HMBC spectrum of cyanopeptolin CP 990 in DMSO-d6.

Table S7. NMR Spectroscopic Data for cyanopeptolin CP 983 – BA-Asp-[Thr-Phe-Ahp-Phe-MeTyr(OMe)-Val].

| Residue | Position | $\delta_C$ | $\delta_H$ (*J* in Hz) | ROESY | HMBC[a] |
|---|---|---|---|---|---|
| BA | 1 | 172.7 | | | |
| | 2 | 37.8 | 2.11, t (6.9) | Asp-NH | BA-1, BA-3, BA-4 |
| | 3 | 19.2 | 1.54[e], m | | BA-2, BA-4 |
| | 4 | 14.1 | 0.89, t (7.5) | | BA-2, BA-3 |
| Asp | 1 | *nd* | | | |
| | 2 | 50.2 | 4.52, m | | |
| | 3a | *nd* | 2.46, m | | |
| | 3b | | 2.36, m | | |
| | 4 | *nd* | | | |
| | NH | | 8.23, d (7.6) | BA-2 | |
| Thr | 1 | *nd* | | | |
| | 2 | 54.8 | 4.48, m | Phe4-NH | |
| | 3 | 72.6 | 5.35, m | Phe4-NH | |
| | 4 | 18.0 | 1.13, d (6.7) | | Thr-2, Thr-3 |
| | NH | | 7.70, m | | |
| Phe4 | 1 | *nd* | | | |
| | 2 | 53.9 | 4.39, m | | |
| | 3a | 36.3 | 3.25, m | Phe4-2′/6′ | |
| | 3b | | 2.62, m | Phe4-2′/6′ | |
| | 1′ | 137.7/138.6[b] | | | |
| | 2′/6′ | 126.6-131.0[c] | 7.11-7.14[c] | Phe4-3a, Phe4-3b | |
| | 3′/5′ | 126.5/128.3[d] | 7.17-7.19[d] | | |
| | 4′ | 126.6-131.0[c] | 7.11-7.14[c] | | |
| | NH | | 8.55, d (8.4) | Thr-2, Thr-3 | |
| Ahp | 1 | *nd* | | | |
| | 2 | 49.4 | 3.64, m | | |
| | 3a | 21.9 | 2.41, m | | |
| | 3b | | 1.64, m | | |
| | 4a | 29.7 | 1.71, m | | |
| | 4b | | 1.56[e], m | Phe6-2′/6′ | |
| | 5 | 74.2 | 5.06, brs | Phe6-3a, Phe6-2′/6′ | |
| | NH | | 7.08, d (9.2) | | |
| | OH | | 6.02 | | |
| Phe6 | 1 | 170.8 | | | |
| | 2 | 50.7 | 4.70, m | Phe6-2′/6′ | |
| | 3a | 35.8 | 2.87, m | Ahp-5, Phe6-2′/6′ | |
| | 3b | | 1.79, m | Phe6-2′/6′ | |
| | 1′ | 137.7/138.6[b] | | | |
| | 2′/6′ | 129.8 | 6.79, d (7.1) | Phe6-2, Phe6-3a, Phe6-3b, Ahp-4b, Ahp-5 | |
| | 3′/5′ | 126.5/128.3[d] | 7.17-7.19[d] | | |
| | 4′ | 126.6-131.0[c] | 7.11-7.14[c] | | |
| MeTyr(OMe) | 1 | *nd* | | | |
| | 2 | 61.3 | 4.92, m | Val-NH | |
| | 3a | 33.3 | 3.17, m | | |
| | 3b | | 2.77, m | | MeTyr(OMe)-2 |
| | 1′ | *nd* | | | |
| | 2′/6′ | 126.6-131.0[c] | 7.11-7.14[c] | MeTyr(OMe)-3a, MeTyr(OMe)-3b | MeTyr(OMe)-3 |
| | 3′/5′ | 114.5 | 6.96, d (8.6) | MeTyr(OMe)-$OCH_3$ | |
| | 4′ | 158.6 | | | |
| | $OCH_3$ | 55.5 | 3.71, s | | MeTyr(OMe)-4′ |
| | $NCH_3$ | 30.8 | 2.78, s | MeTyr(OMe)-3′/5′ | Phe6-1 |
| Val | 1 | *nd* | | | |
| | 2 | 56.3 | 4.63, m | | |
| | 3 | 31.4 | 2.03, m | | |
| | 4 | 19.7 | 0.86, d (6.9) | | Val-2, Val-3, Val-5 |
| | 5 | 17.8 | 0.72, d (6.9) | | Val-2, Val-3, Val-4 |
| | NH | | 7.42, d (9.4) | MeTyr(OMe)-2 | |

[a] HMBC correlations are given from proton(s) stated to the indicated carbon atom; [c,d] low amount of the sample made the unambiguous assignments of the aromatic signals impossible; [e] assignments are overlapping; *nd* – resonances not detected

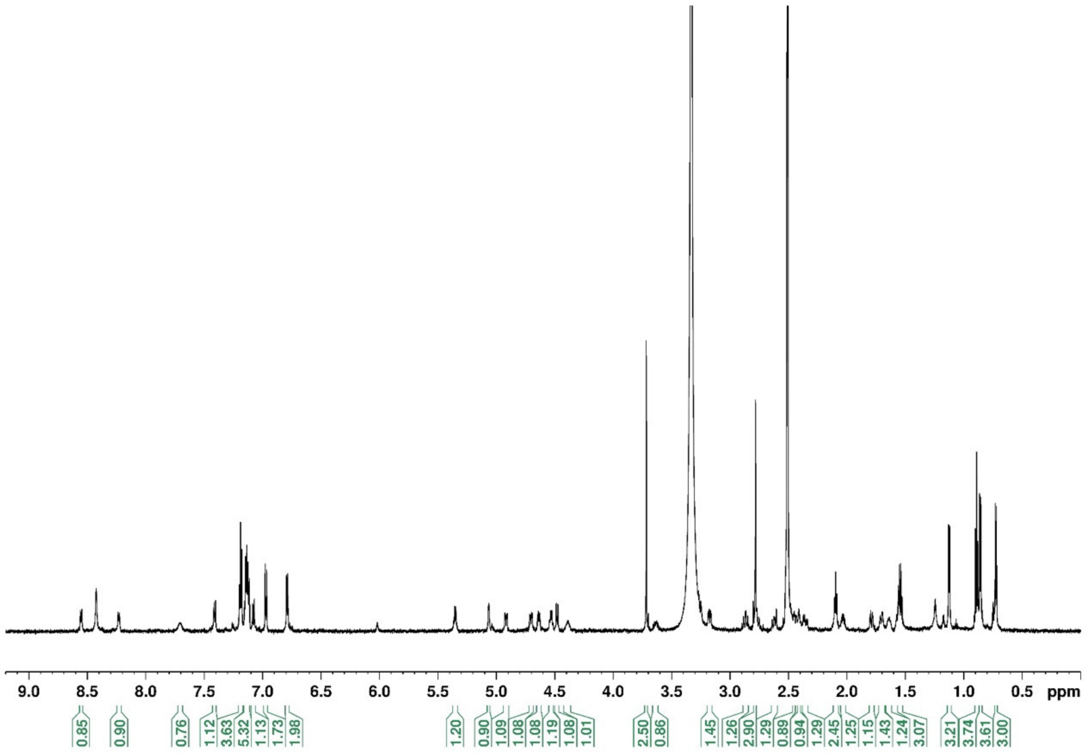

Figure S106. $^{1}$H NMR spectrum of cyanopeptolin CP 983 in DMSO-d6.

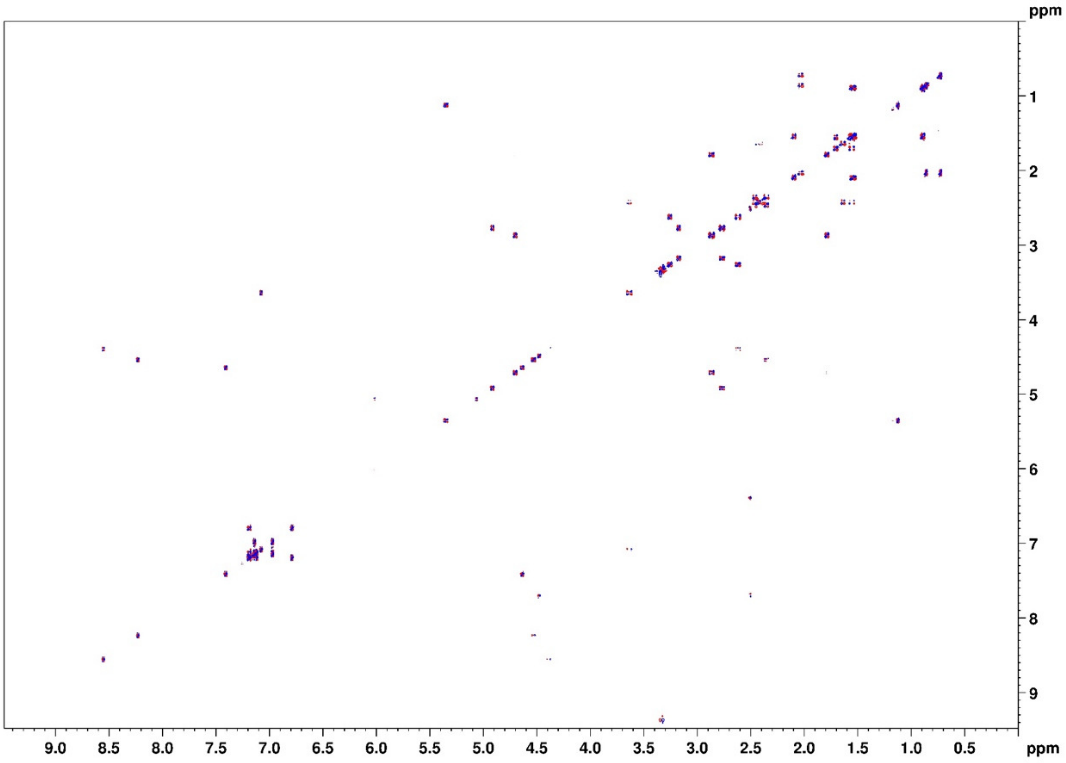

Figure S107. DQF-COSY spectrum of cyanopeptolin CP 983 in DMSO-d6.

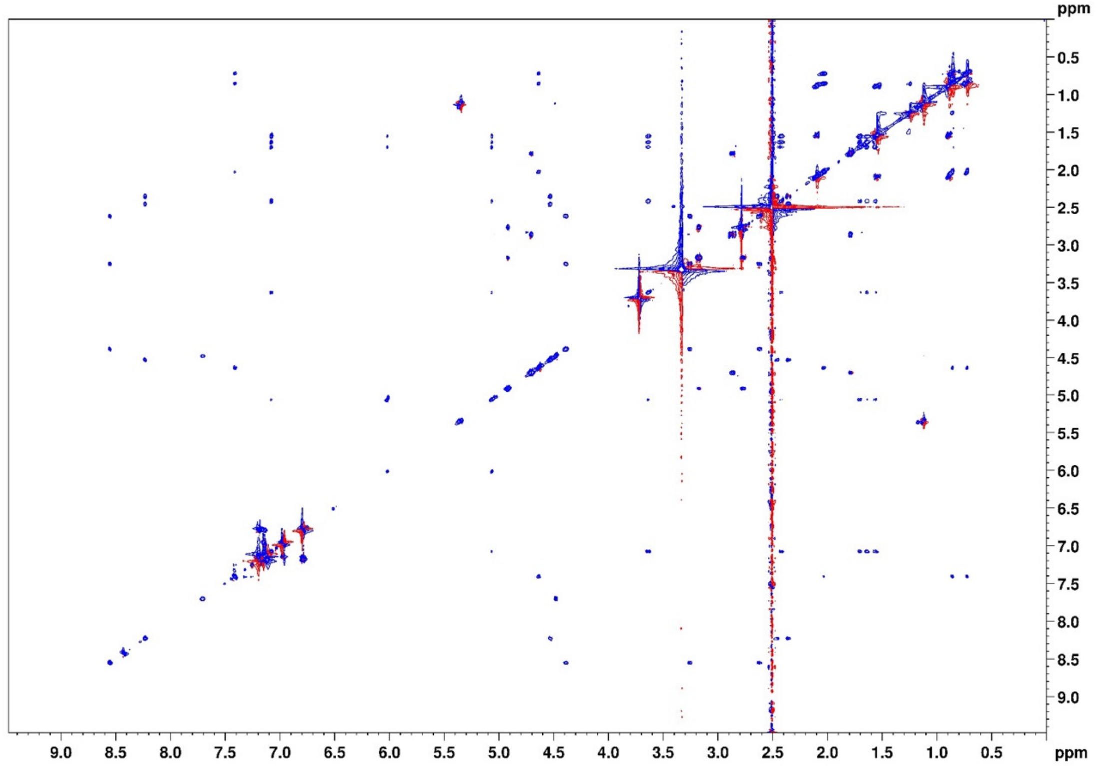


Figure S108. TOCSY spectrum of cyanopeptolin CP 983 in DMSO-d$_6$.

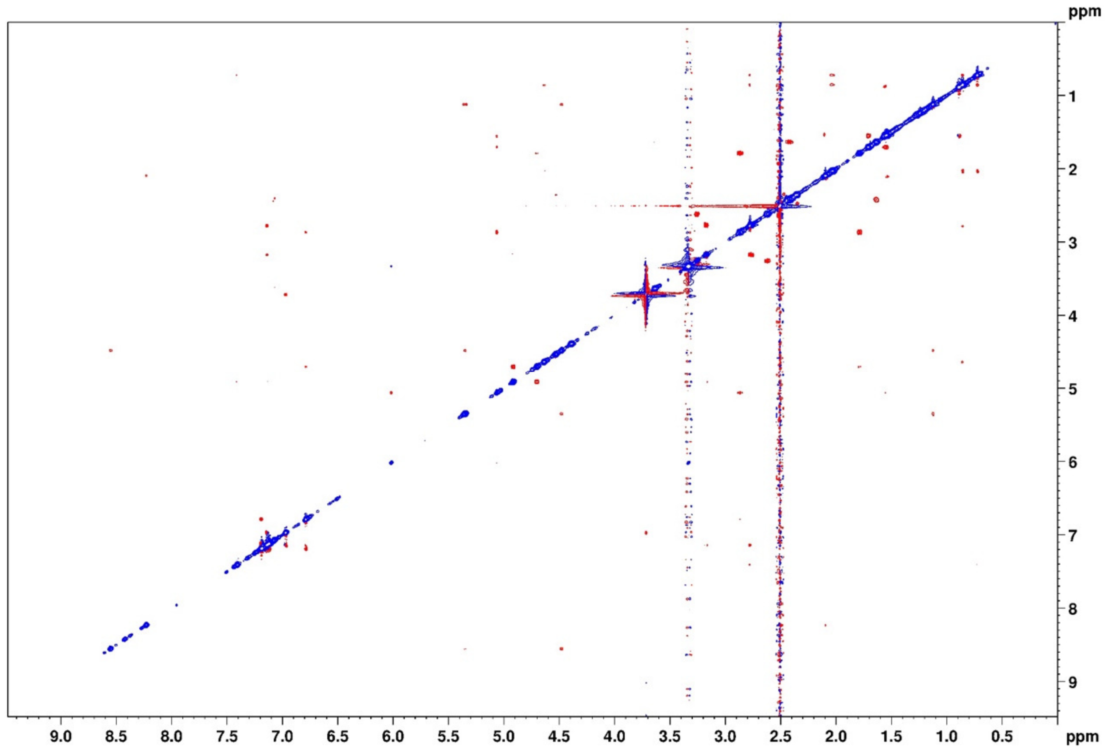


Figure S109. ROESY spectrum of cyanopeptolin CP 983 in DMSO-d$_6$.

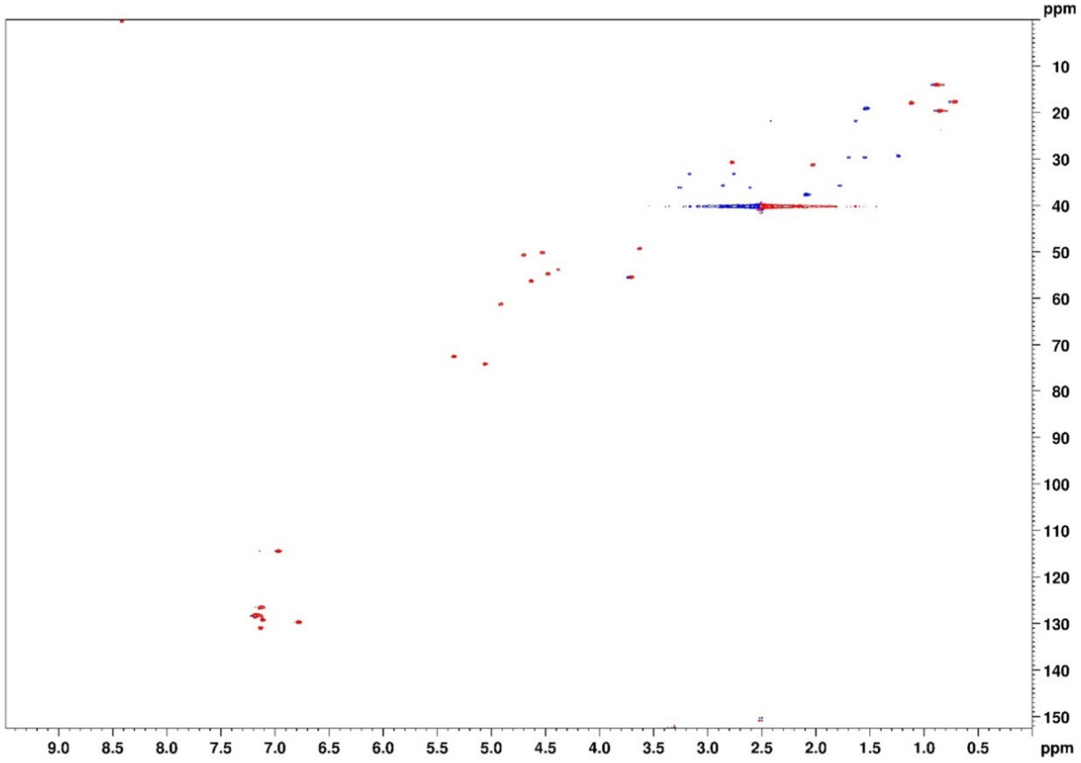


Figure S110. HSQC spectrum of cyanopeptolin CP 983 in DMSO-d6.

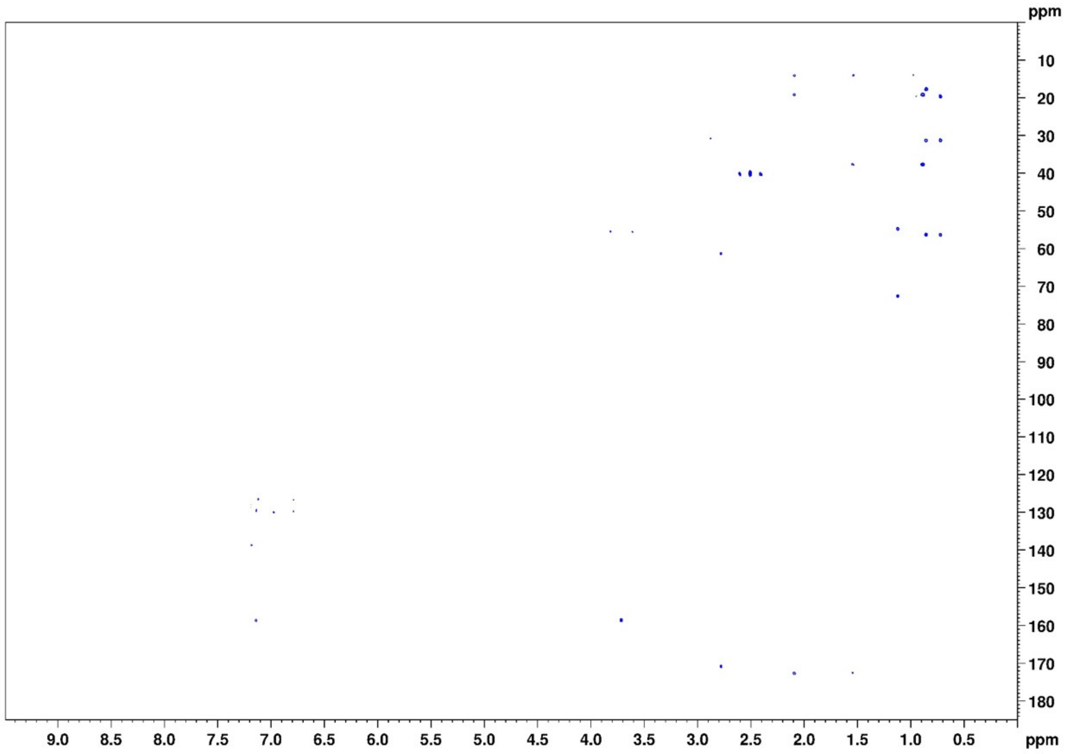


Figure S111. HMBC spectrum of cyanopeptolin CP 983 in DMSO-d6.

Table S8. NMR Spectroscopic Data for cyanopeptolin CP 949 – BA-Asp-[Thr-Leu-Ahp-Phe-MeTyr(OMe)-Val]

| Residue | Position | $\delta_C$ | $\delta_H$ | ROESY | HMBC[a] |
|---|---|---|---|---|---|
| BA | 1 | 172.8 | | | |
| | 2 | 37.7 | 2.11 | | BA-1 |
| | 3 | 19.2 | 1.55 | | BA-1, BA-2 |
| | 4 | 14.1 | 0.90 | | BA-2, BA-3 |
| Asp | 1 | *nd* | | | |
| | 2 | 50.1 | 4.64 | Thr-NH | |
| | 3a | 36.9 | 2.66 | | |
| | 3b | | 2.45 | | |
| | 4 | *nd* | | | |
| | NH | | 8.25 | | |
| Thr | 1 | *nd* | | | |
| | 2 | 55.0 | 4.55 | Leu-NH | |
| | 3 | 72.5 | 5.37 | Leu-NH | |
| | 4 | 18.2 | 1.17 | | |
| | NH | | 7.71 | Asp-2 | |
| Leu | 1 | *nd* | | | |
| | 2 | 50.9 | 4.18 | | |
| | 3a | 39.5 | 1.71 | | |
| | 3b | | 1.31 | | |
| | 4 | 24.6 | 1.45 | | |
| | 5 | 23.7 | 0.84 | | Leu-4 |
| | 6 | 21.5 | 0.75 | | |
| | NH | | 8.39 | Thr-2, Thr-3 | |
| Ahp | 1 | *nd* | | | |
| | 2 | 49.1 | 3.63 | | |
| | 3a | 22.0 | 2.41 | | |
| | 3b | | 1.58 | | |
| | 4a | 29.8 | 1.70 | | |
| | 4b | | 1.52 | | |
| | 5 | 74.2 | 5.07 | Phe-3a | |
| | NH | | 7.07 | | |
| | OH | | | | |
| Phe | 1 | 170.8 | | | |
| | 2 | 50.7 | 4.71 | Phe-2′/6′ | |
| | 3a | 35.8 | 2.86 | Ahp-5, Phe-2′/6′ | |
| | 3b | | 1.78 | Phe-2′/6′ | |
| | 1′ | *nd* | | | |
| | 2′/6′ | 129.7 | 6.78 | Phe-2, Phe-3a, Phe-3b | |
| | 3′/5′ | 128.2 | 7.17 | | |
| | 4′ | 126.7 | 7.13 | | |
| MeTyr(OMe) | 1 | | | | |
| | 2 | 61.3 | 4.94 | Val-NH | |
| | 3a | 33.2 | 3.20 | MeTyr(OMe)-2′/6′ | |
| | 3b | | 2.78 | MeTyr(OMe)-2′/6′ | |
| | 1′ | *nd* | | | |
| | 2′/6′ | 131.0 | 7.15 | MeTyr(OMe)-3a, MeTyr(OMe)-3b | MeTyr(OMe)-4′ |
| | 3′/5′ | 114.5 | 6.98 | MeTyr(OMe)-$OCH_3$ | |
| | 4′ | 158.6 | | | |
| | $OCH_3$ | 55.6 | 3.72 | MeTyr(OMe)-3′/5′ | MeTyr(OMe)-4′ |
| | $NCH_3$ | 30.8 | 2.78 | | Phe-1 |
| Val | 1 | *nd* | | | |
| | 2 | 56.3 | 4.67 | | |
| | 3 | 31.3 | 2.05 | | |
| | 4 | 19.7 | 0.88 | | Val-2, Val-3, Val-5 |
| | 5 | 17.8 | 0.74 | | Val-2, Val-3, Val-4 |
| | NH | | 7.43 | MeTyr(OMe)-2 | |

[a] HMBC correlations are given from proton(s) stated to the indicated carbon atom; *nd* – resonances not detected

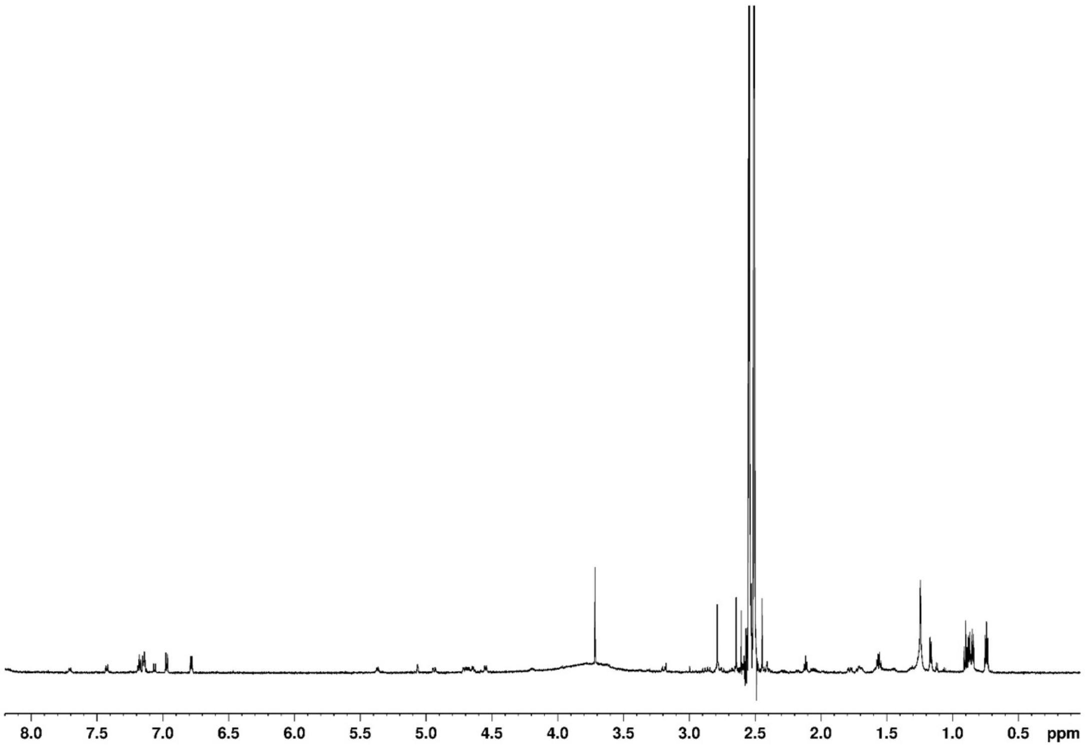


Figure S112. $^1$H NMR spectrum of cyanopeptolin CP 949 in DMSO-d$_6$.

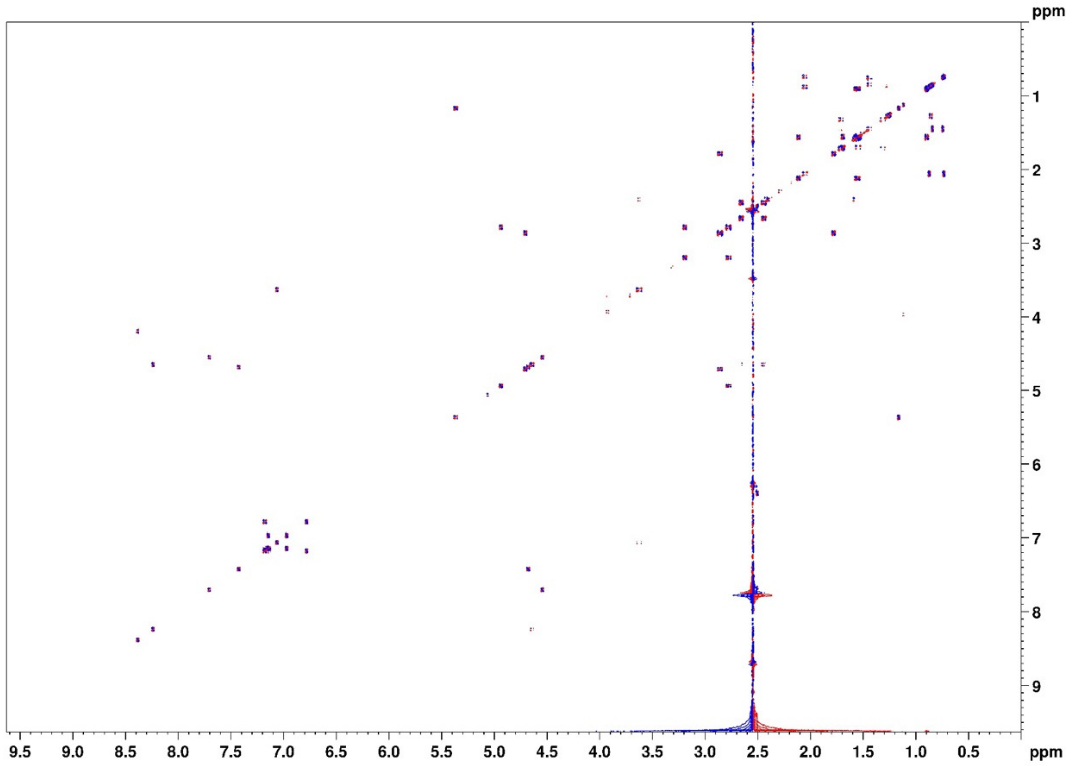


Figure S113. DQF-COSY spectrum of cyanopeptolin CP 949 in DMSO-d$_6$.

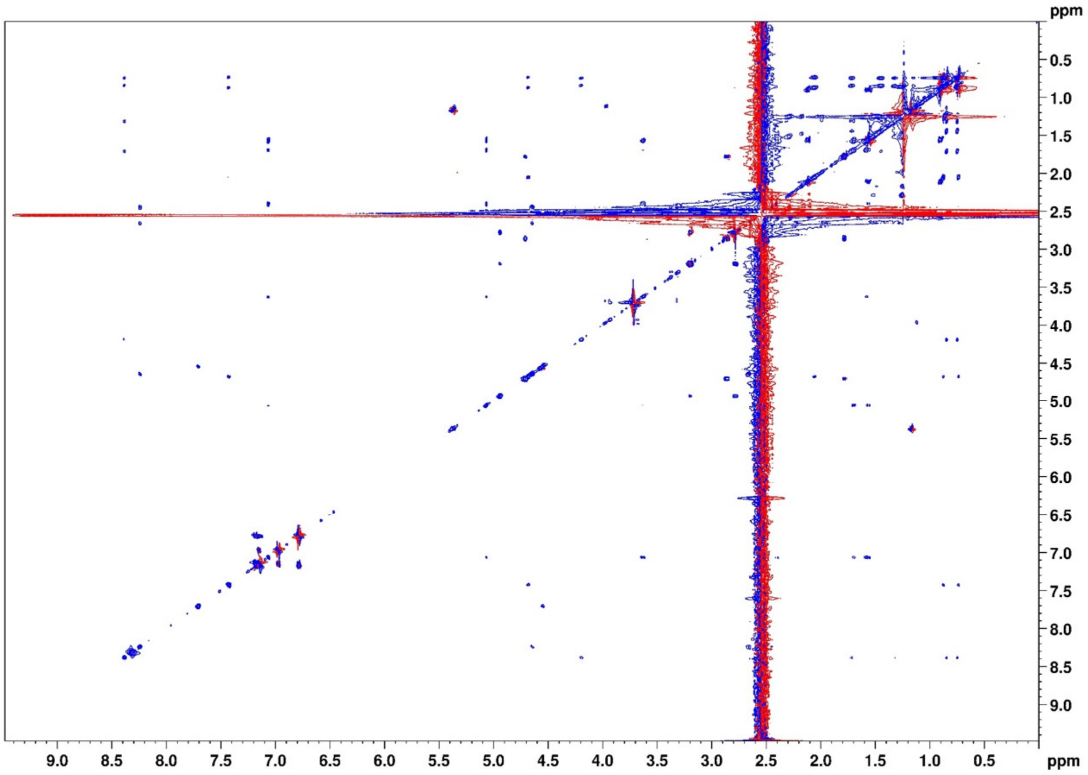


Figure S114. TOCSY spectrum of cyanopeptolin CP 949 in DMSO-d6.

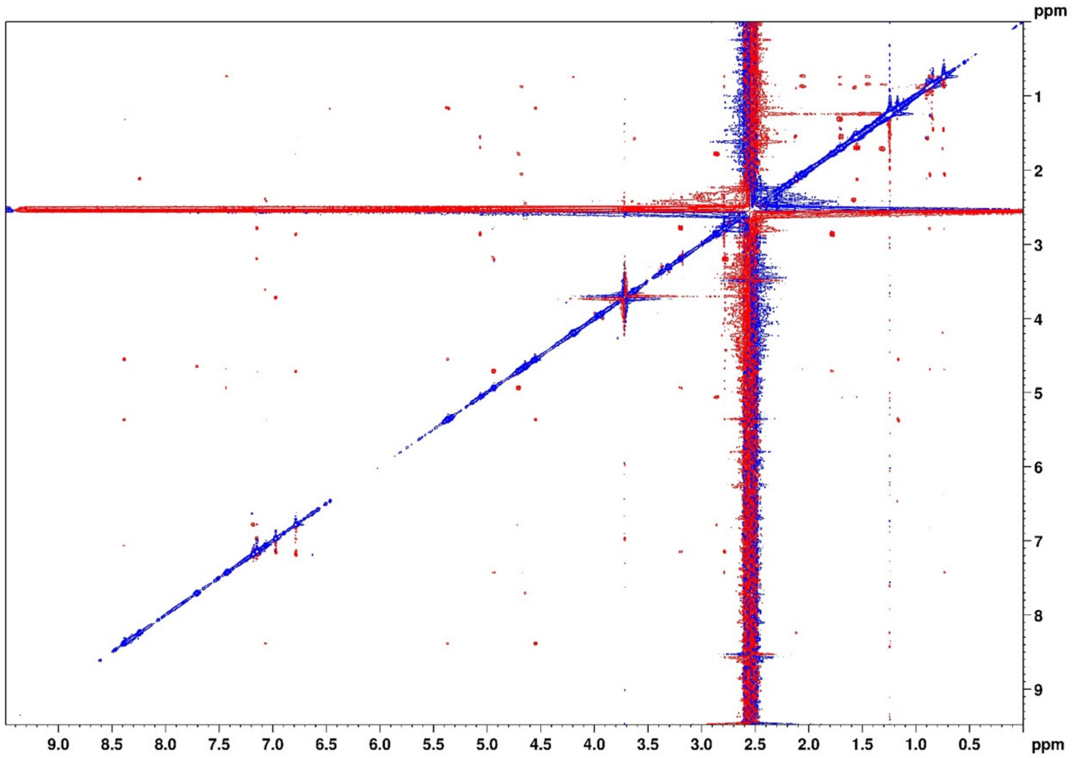


Figure S115. ROESY spectrum of cyanopeptolin CP 949 in DMSO-d6.

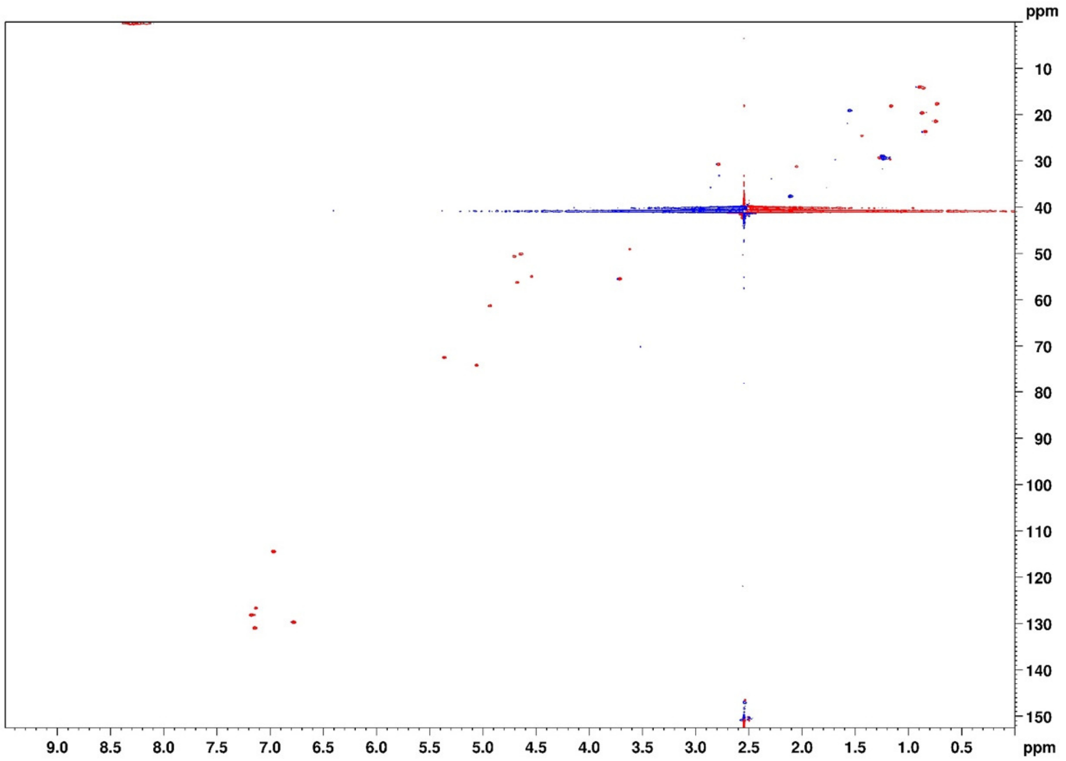


Figure S116. HSQC spectrum of cyanopeptolin CP 949 in DMSO-d6.

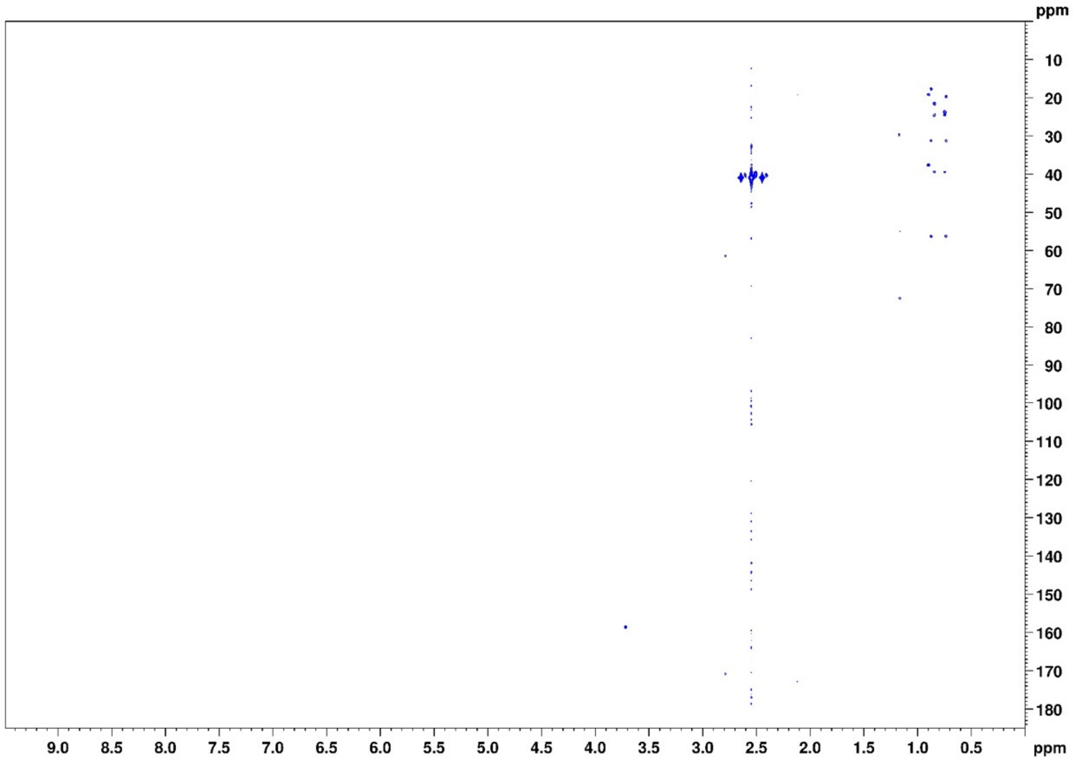


Figure S117. HMBC spectrum of cyanopeptolin CP 949 in DMSO-d6.

Table S9. NMR Spectroscopic Data for cyanopeptolin CP 919 – BA-Asp-[Thr-Leu-Ahp-Phe-MePhe-Val].

| Residue | Position | $\delta_C$ | $\delta_H$ (*J* in Hz) | ROESY | HMBC[a] |
|---|---|---|---|---|---|
| BA | 1 | 172.2 | | | |
| | 2 | 37.9 | 2.11, t (7.2) | Asp-NH | BA-1, BA-3, BA-4 |
| | 3 | 19.4 | 1.54, q (7.3) | | BA-1, BA-2, BA-4 |
| | 4 | 14.1 | 0.89[b], t | | BA-2, BA-3 |
| Asp | 1 | *nd* | | | |
| | 2 | 50.6 | 4.58, m | | |
| | 3a | 40.9 | 2.35, m | | |
| | 3b | | 2.27, m | | |
| | 4 | *nd* | | | |
| | NH | | 8.19, m | BA-2 | |
| Thr | 1 | *nd* | | | |
| | 2 | 55.1 | 4.53, d (8.7) | Leu-NH | |
| | 3 | 72.7 | 5.36, m | Leu-NH | |
| | 4 | 18.4 | 1.19, d (6.5) | | Thr-2, Thr-3 |
| | NH | | *nd* | | |
| Leu | 1 | *nd* | | | |
| | 2 | 51.1 | 4.16, m | | |
| | 3a | 39.3 | 1.71[c], m | | |
| | 3b | | 1.35, m | | |
| | 4 | 24.6 | 1.45, m | | |
| | 5 | 23.8 | 0.84, d (6.7) | | Leu-6 |
| | 6 | 21.5 | 0.75[d],d | | Leu-5 |
| | NH | | 8.40, m | Thr-2, Thr-3 | |
| Ahp | 1 | *nd* | | | |
| | 2 | 49.1 | 3.62, m | | |
| | 3a | 22.3 | 2.39, m | | |
| | 3b | | 1.56, m | | |
| | 4a | 29.5 | 1.69, m | | |
| | 4b | | 1.51, m | | |
| | 5 | 74.1 | 5.04[e], m | | |
| | NH | | 7.07, d (9.4) | | |
| | OH | | 6.09 | | |
| Phe | 1 | 170.9 | | | |
| | 2 | 50.3 | 4.75, m | MePhe-2 | |
| | 3a | 35.8 | 2.84[f], m | Phe-2′/6′ | |
| | 3b | | 1.71[c], m | Phe-2′/6′ | |
| | 1′ | 137.2 | | | |
| | 2′/6′ | 129.8 | 6.78, d (6.9) | Phe-3a, Phe-3b | Phe-3, Phe-4′ |
| | 3′/5′ | 128.2 | 7.18, t (7.3) | | Phe-1′ |
| | 4′ | 126.8 | 7.14, t (7.2) | | Phe-2′/6′ |
| MePhe | 1 | *nd* | | | |
| | 2 | 61.1 | 5.03[e], m | Phe-2, MePhe-2′/6′, NH-Val | |
| | 3a | 34.0 | 3.35, m | | |
| | 3b | | 2.84[f], m | | |
| | 1′ | 138.4 | | | |
| | 2′/6′ | 130.0 | 7.26, d (7.5) | MePhe-2 | MePhe-3, MePhe-4′ |
| | 3′/5′ | 129.1 | 7.41, t (7.5) | | MePhe-1′, MePhe-2′/6′ |
| | 4′ | 127.2 | 7.31, t (7.3) | | MePhe-2′/6′ |
| | $NCH_3$ | 30.8 | 2.81, s | | MePhe-2, Phe-1 |
| Val | 1 | *nd* | | | |
| | 2 | 56.5 | 4.66, m | | |
| | 3 | 31.2 | 2.06, m | | |
| | 4 | 19.9 | 0.89[b], d | | Val-2, Val-3, Val-5 |
| | 5 | 18.0 | 0.76[d], d | | Val-2, Val-3, Val-4 |
| | NH | | 7.52, d (9.4) | MePhe-2 | |

[a] HMBC correlations are given from proton(s) stated to the indicated carbon atom; [b-g] assignments with the same superscript are overlapping; *nd* – resonances not detected

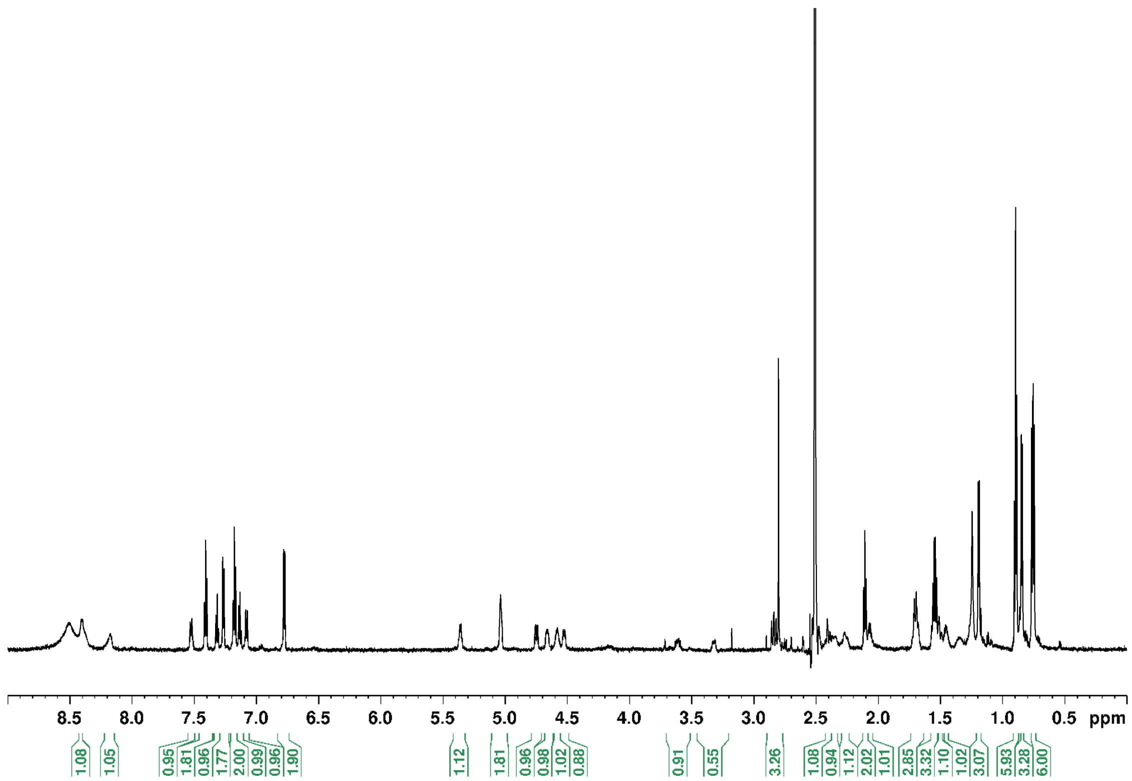


Figure S118. $^{1}$H NMR spectrum of cyanopeptolin CP 919 in DMSO-d6.

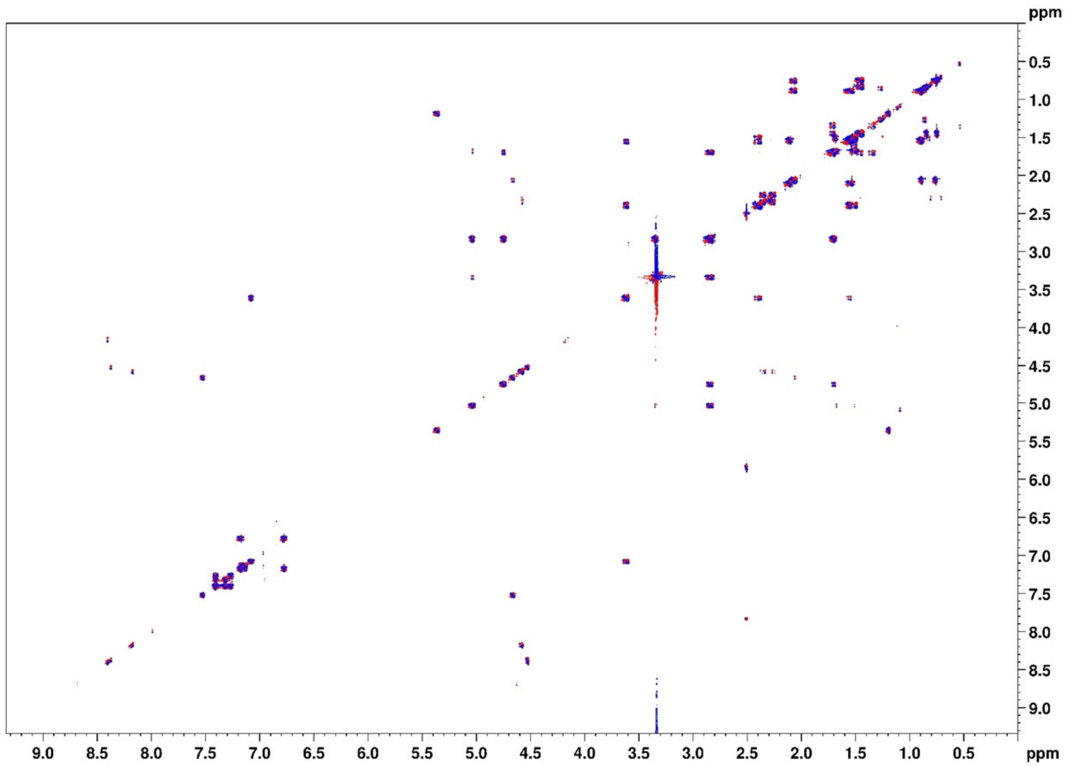


Figure S119. DQF-COSY spectrum of cyanopeptolin CP 919 in DMSO-d6.

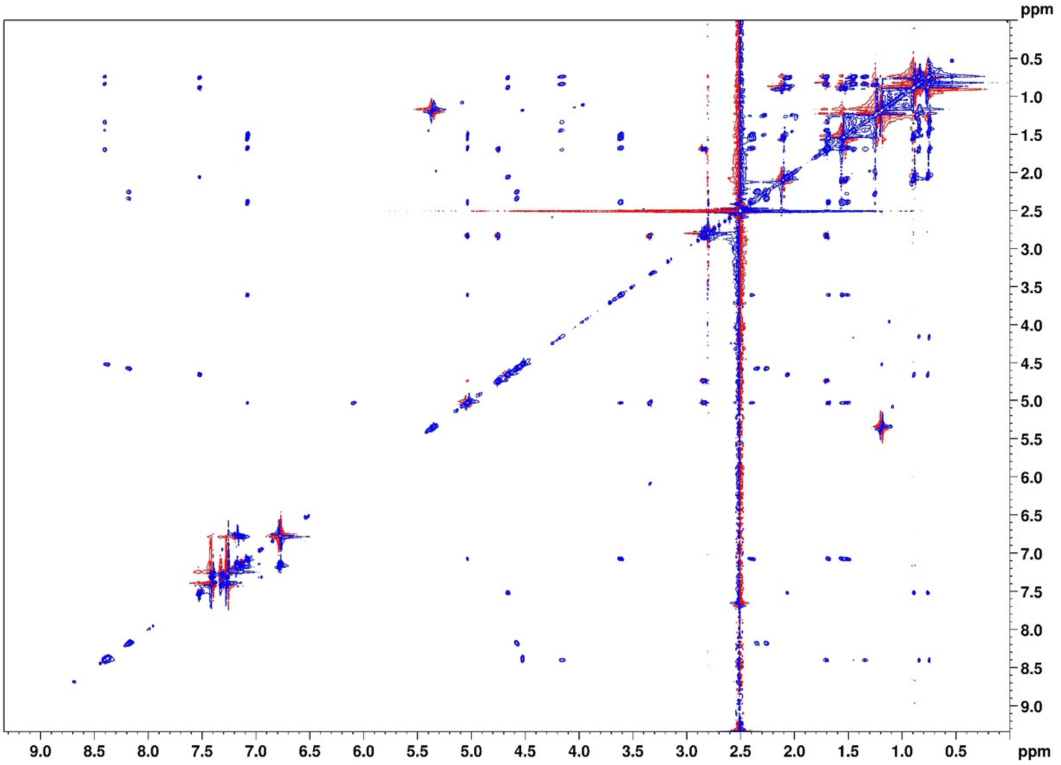


Figure S120. TOCSY spectrum of cyanopeptolin CP 919 in DMSO-d6.

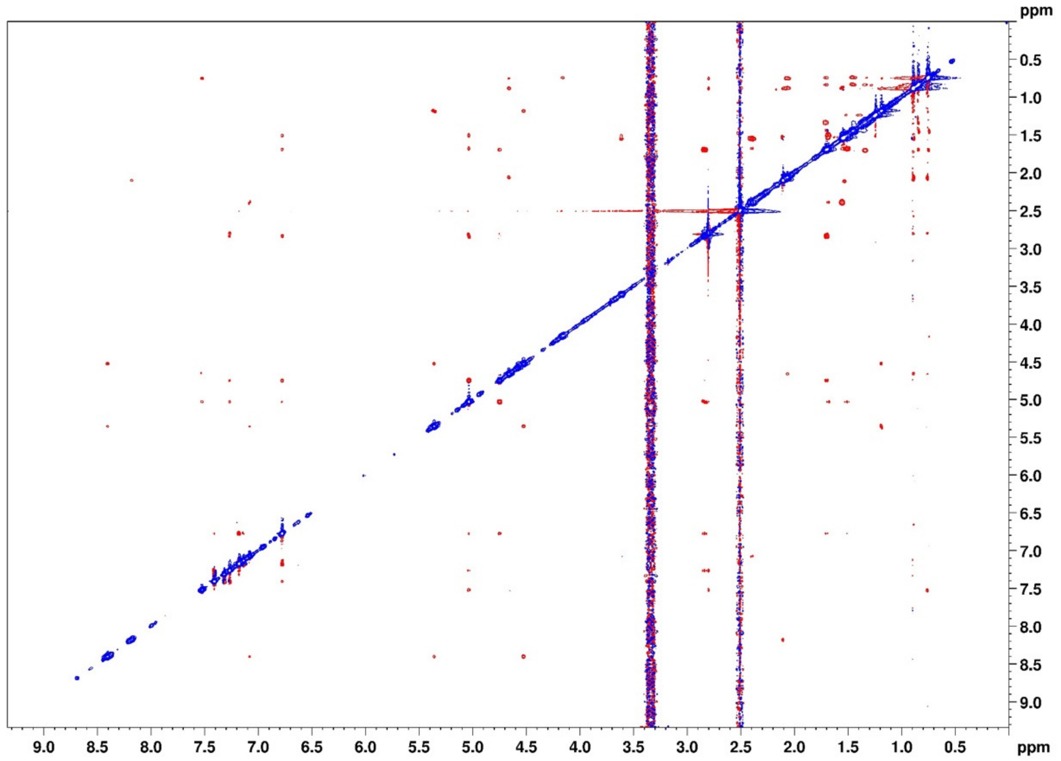


Figure S121. ROESY spectrum of cyanopeptolin CP 919 in DMSO-d6.

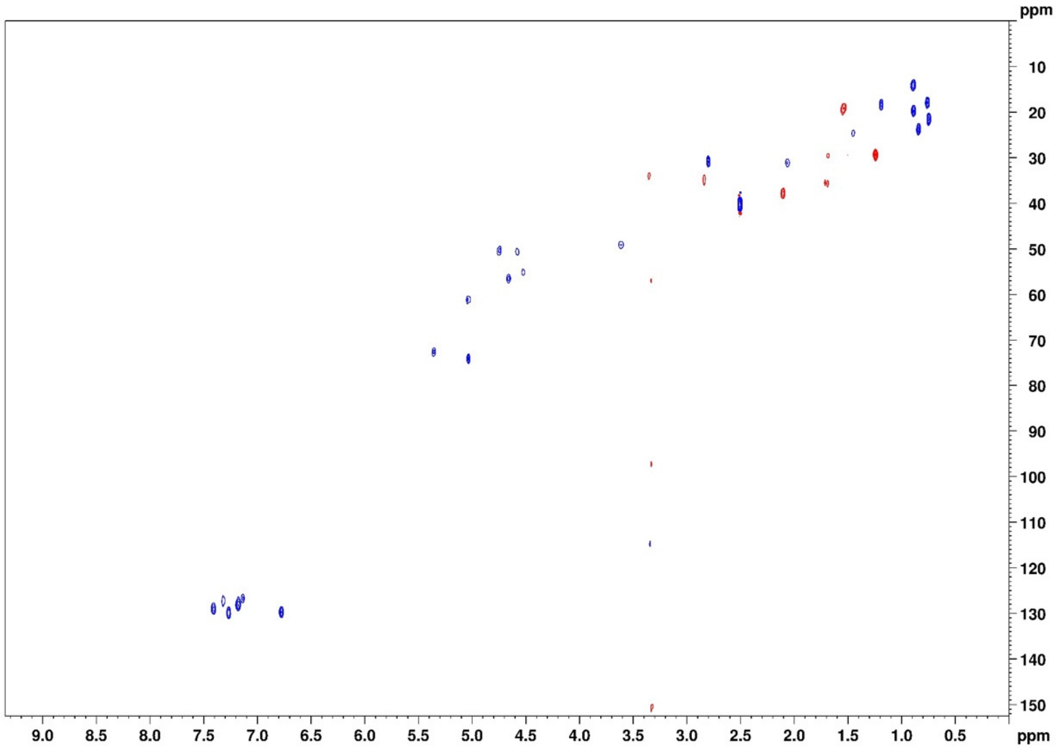


Figure S122. HSQC spectrum of cyanopeptolin CP 919 in DMSO-$d_6$.

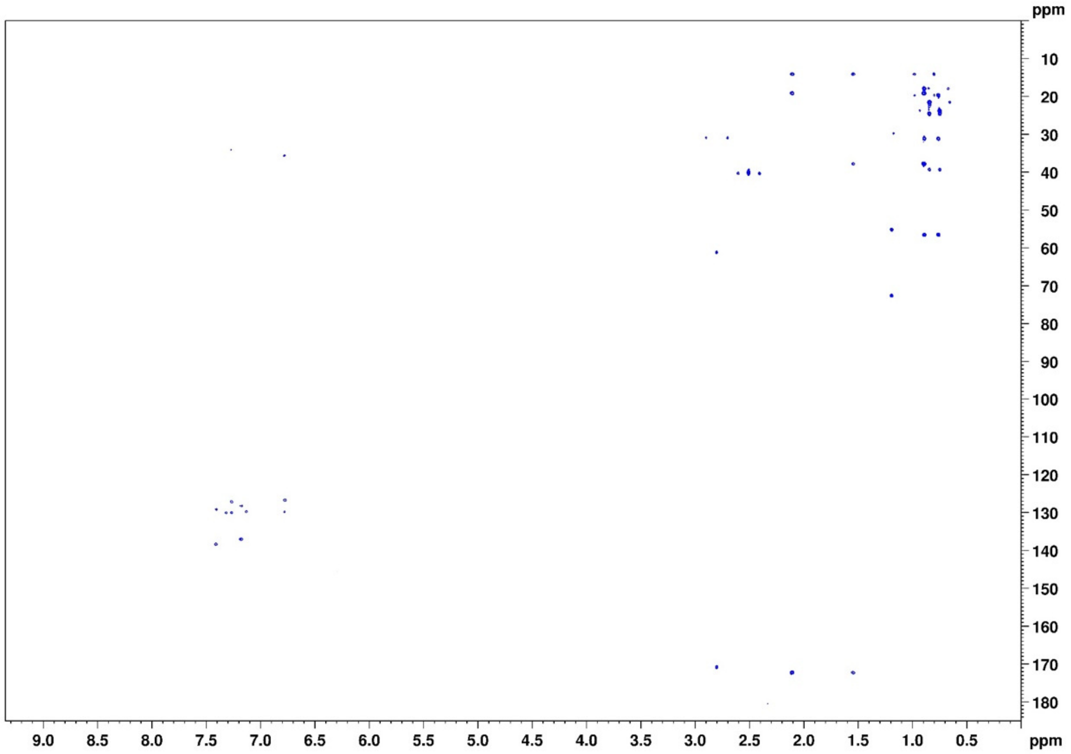


Figure S123. HMBC spectrum of cyanopeptolin CP 919 in DMSO-$d_6$.

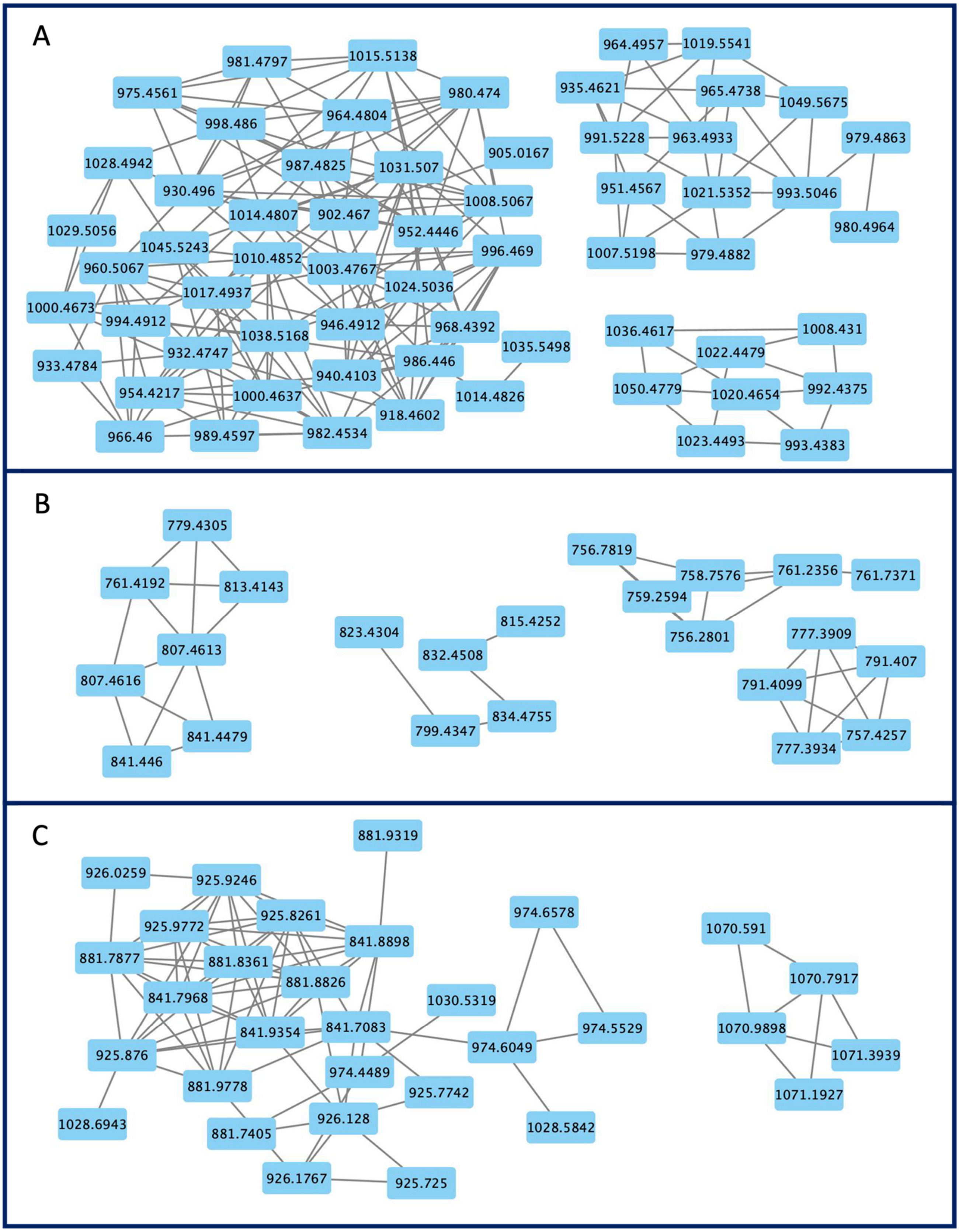


Figure S124. A CCNP1411 clusters formed by the GNPS analysis based on the HRMS/MS fragmentation spectra obtain from Nostoc edaphicum CCNP1411 extract. Clusters are separate as: A – nodes containing CPs features; B – nodes containing Nostocyclopeptides features C – nodes containing unknow features.
